# Predicting Drug-Target Interaction Networks Based on Functional Groups and Biological Features

**DOI:** 10.1371/journal.pone.0009603

**Published:** 2010-03-11

**Authors:** Zhisong He, Jian Zhang, Xiao-He Shi, Le-Le Hu, Xiangyin Kong, Yu-Dong Cai, Kuo-Chen Chou

**Affiliations:** 1 Institute of System Biology, Shanghai University, Shanghai, China; 2 CAS-MPG Partner Institute of Computational Biology, Shanghai Institutes for Biological Sciences (SIBS), Chinese Academy of Sciences (CAS), Shanghai, China; 3 Department of Ophthalmology, Yangpu District Central Hospital, Shanghai, China; 4 Institute of Health Sciences, Shanghai Institutes for Biological Sciences (SIBS), Chinese Academy of Sciences (CAS) and Shanghai Jiao Tong University School of Medicine (SJTUSM), Shanghai, China; 5 Centre for Computational Systems Biology, Fudan University, Shanghai, China; 6 State Key Laboratory of Medical Genomics, Ruijin Hospital, Shanghai Jiaotong University, Shanghai, China; 7 Gordon Life Science Institute, San Diego, California, United States of America; Cairo University, Egypt

## Abstract

**Background:**

Study of drug-target interaction networks is an important topic for drug development. It is both time-consuming and costly to determine compound-protein interactions or potential drug-target interactions by experiments alone. As a complement, the in silico prediction methods can provide us with very useful information in a timely manner.

**Methods/Principal Findings:**

To realize this, drug compounds are encoded with functional groups and proteins encoded by biological features including biochemical and physicochemical properties. The optimal feature selection procedures are adopted by means of the mRMR (Maximum Relevance Minimum Redundancy) method. Instead of classifying the proteins as a whole family, target proteins are divided into four groups: enzymes, ion channels, G-protein- coupled receptors and nuclear receptors. Thus, four independent predictors are established using the Nearest Neighbor algorithm as their operation engine, with each to predict the interactions between drugs and one of the four protein groups. As a result, the overall success rates by the jackknife cross-validation tests achieved with the four predictors are 85.48%, 80.78%, 78.49%, and 85.66%, respectively.

**Conclusion/Significance:**

Our results indicate that the network prediction system thus established is quite promising and encouraging.

## Introduction

Identification of drug-target interaction networks is an essential step in the drug discovery pipeline [1]. The emergence of molecular medicine and the completion of the human genome project provide more opportunity to discover unknown target proteins of drugs. Many efforts have been made to discover new drugs in the past few years. However, the number of new drug approvals remains quite low (around only 30 per year). This is partially because many compounds or drug candidates have to be withdrawn owing to unacceptable toxicity. Such failures have wasted a lot of money. It would be beneficial to develop computational methods for predicting the sensitivity and toxicity before a drug candidate was synthesized [2], [3], [4]. However, a number of problems need to be overcome in order to find out the exact effects of a drug. Firstly, drugs could have numerous effects including positive and negative effects, and it is hard to find out and elucidate the possible effects; secondly, different people would have completely different responses to a drug even though the same gene products are only slightly different [5], [6], [7], [8]; thirdly, it is very hard to trace the drug effects since the biological interaction pathways are extremely complicated in human beings. Therefore, it would be very helpful for drug development if the interactions between drugs and target proteins could be predicted more accurately and the underlying mechanisms could be better understood.

Several computational approaches have been developed for analyzing and predicting drug-protein interactions. The most commonly used are docking simulations [9], [10], [11], [12], literature text mining [13], and combining chemical structure, genomic sequence, and 3D structure information [14], among others (see, e.g., [15], [16], [17]).

Machine learning and data mining methods have been widely used in the computational biology and bioinformatics area. Many researchers have made lots of efforts to develop useful algorithms and softwares to investigate various drug-related biological problems, such as HIV protease cleavage site prediction [18], [19], identification of GPCR (G protein-coupled receptors) type [20], [21], protein signal peptide prediction [22], protein subcellular location prediction [23], [24], [25], analysis of specificity of GalNAc-transferase protein [26], identification of protease type [27], [28], membrane protein type prediction [29], [30], [31], [32], and a series of relevant web-server predictors as summarized in a recent review [33].

Here we propose a predictor for drug-target interactions based on the Nearest Neighbor algorithm [34]. Since biochemical and physicochemical features [35] are important for characterizing proteins, in this study they are used to represent proteins as done by many previous investigators (see, e.g., [36], [37], [38]. To improve the predictor's performance, minimum Redundancy Maximum Relevance (mRMR) algorithm [39] is used to rank the features. Meanwhile, the Incremental Feature Selection and Forward Feature Selection are applied for feature selection. The protein targets for drugs are divided into enzymes, ion channels [40], [41], [42], [43], GPCRs [44], [45], and nuclear receptors [14] in this study. Finally, four predictors for predicting the interactions of drugs with each of the four protein families are developed in hopes that they can help provide useful information for drug design.

## Materials and Methods

### Benchmark Datasets

In addition to the dataset used by Yamanishi et al. [14], information about drug compounds and genes can be obtained from KEGG [46], [47] by the FTP operations: ftp://ftp.genome.jp/pub/kegg/ligand/drug/drug for the drugs, and ftp://ftp.genome.jp/pub/kegg/genes/fasta/gene.pep for the genes. After excluding the drug-target pairs that lack experimental information, we finally obtained a total of 4,797 drug-target pairs, of which 2,719 for enzymes, 1,372 for ion channels, 630 for GPCRs, and 82 for nuclear receptors. All these datasets were used as the positive datasets in the current study.

The corresponding negative datasets were derived from the above positive datasets via the following steps: (1) separate the pairs in the above positive dataset into single drugs and proteins; (2) re-couple these singles into pairs in a way that none of them occurs in the corresponding positive dataset; (3) randomly picked the negative pairs thus formed until they reached the number two times as many as the positive pairs.

The drug-target benchmark datasets thus obtained for enzymes, ion-channels, GPCRs, and nuclear receptors are given in Online Supporting Information S1, S2, S3, and S4, respectively.

### Feature Vector Construction

#### Representing drugs with chemical functional groups composition

The number of drugs is extremely large. However, most of them are small organic molecules and are composed of some fixed small structures, called functional groups. Since functional groups usually represent the characteristics of a compound as well as its reaction mechanism with other molecules, features derived from its functional groups could be very effective in characterizing a drug. Moreover, the number of common functional groups is quite small, and hence it is possible to use the functional group composition to uniquely represent a drug [48]. A number of functional groups are available in nature, and we selected the following 28 common groups for the current study: (1) alcohol, (2) aldehyde, (3) amide, (4) amine, (5) hydroxamic acid, (6) phosphorus, (7) carboxylate, (8) methyl, (9) ester, (10) ether, (11) imine, (12) ketone, (13) nitro, (14) halogen, (15) thiol, (16) sulfonic acid, (17) sulfone, (18) sulfonamide, (19) sulfoxide, (20) sulfide, (21) a_5c_ring, (22) ar_6c_ring, (23) non_ar_5c_ring, (24) non_ar_6c_ring, (25) hetero ar_6_ring, (26) hetero non_ar_5_ring, (27) hetero non_ar_6_ring, and (28) hetero ar_5_ring. Thus, following the same treatment as in [23], a drug compound can now be formulated as a 28-D (dimensional) vector given below:
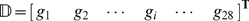
(1)where 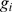 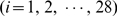 is the occurrence frequency of the 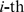 functional group in the drug 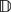, and 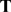 the matrix transpose operator.

#### Representing target proteins with pseudo amino acid composition by incorporating biochemical and physicochemical features

Now the problem is how to effectively represent a target protein. Two kinds of representations are generally used in this regard: the sequential representation and the non-sequential representation. The most typical sequential representation for a protein sample is its entire amino acid sequence, which can contain the most complete information of a protein. To deal with this model, the sequence-similarity-search-based tools, such as BLAST [49], are usually used to find the desired results. Unfortunately, this kind of approach failed to work when the query protein did not have significant homology to the proteins in the training dataset. Thus, various non-sequential representations or discrete models were proposed. The simplest discrete model was based on the amino acid composition (AAC) (see, e.g., [50]). However, if using the AAC model to represent a protein, all its sequence-order information will be lost. To avoid completely losing the sequence-order information, the pseudo amino acid composition (Pse-AAC) was proposed [36] to represent the sample of a protein. The PseAAC can be used to represent a protein sequence with a discrete model yet without completely losing its sequence-order information. For further information about PseAAC, see the web-page by clicking the link http://en.wikipedia.org/wiki/Pseudo_amino_acid_composition. Ever since the concept of PseAAC was introduced, it has been widely used to study various problems in proteins and protein-related systems (see, e.g., [37], [51], [52], [53], [54], [55], [56], [57], [58], [59], [60], [61], [62], [63], [64], [65], [66]). Meanwhile, many different forms of discrete models were also proposed (see, e.g., [20], [30], [32], [51], [67], [68], [69], [70], [71], [72], [73], [74], [75], [76], [77], [78], [79], [80], [81], [82]). However, regardless of how much different these models are, they just belong to different forms of PseAAC, as elucidated in a recent comprehensive review [83]. Here, we are to propose a different PseAAC to represent drug-targeted proteins in terms of their biochemical and physicochemical features [84]. Six different types of features were considered: (1) hydrophobicity, (2) polarizability, (3) polarity, (4) secondary structure, (5) normalized van der Waals volume, and (6) solvent accessibility.

Each amino acid residue in a protein sequence can be represented by a set of different states according to its features. For instance, its hydrophobicity feature can be marked by one of the following three states: “polar”, “neutral”, or “hydrophobic” [85]; its solvent accessibility feature by one of the two: “buried” or “exposed to solvent”, as predicted by PredAcc [35]; its secondary structure feature by one of the three: “helix”, “sheet”, or “coil”, as predicted by the method in [86]; and so forth.

Thus, a protein sequence can be translated to a series of codes according to the biochemical and physicochemical properties of its constituent amino acid residues. For example, if using “P”, “N” and “H” to represent the three states of hydrophobicity: “polar”, “neutral”, and “hydrophobic”, the protein sequence “DMAEIMSDKPQAGML” can be translated to “PHNPHHNPPNPNNHH” according to the codes of the hydrophobic property feature. The encoded sequences thus obtained would have different length for proteins of different sizes, which will make the prediction engine difficult to handle.

To make the feature-encoded sequence to be a vector with a fixed number of dimensions, three properties of a sequence was used: composition (C), transition (T), and distribution (D). C represents the global composition of each letter in the sequence; T, the frequency of a code letter changing from one to another; D, the distribution pattern of the code letters along the sequence, measuring the percentage of the sequence length within which the first, 25%, 50%, 75%, and 100% of the amino acids of each code letter is located. Take the above hydrophobic property sequence as an example: its C feature is 5/15 = 33.3% for all of P, H, and N, while the T feature is 2/10 = 20%, 3/10 = 30% and 5/10 = 50% for the changes between H and P, N and H, N and P, respectively. The measurement of feature D is a little more complicated. For the letter H, the first, 25%, 50%, 75% and 100% of Hs in the sequence is located at the position of 2, 5, 6, 14, and 15. Thus its D feature is (2/15 = 13.3%, 5/15 = 33.3%, 6/15 = 40%, 14/15 = 93.3%, 15/15 = 100%). In the same way, the distributions of letters P and N are (6.7%, 26.7%, 53.3%, 60%, 73.3%) and (20%, 46.7%, 66.7%, 80%, 86.7%), respectively. Accordingly, the three features of the code letter sequence are: C = (33.3%, 33.3%, 33.3%), T = (20%, 30%, 50%), and D = (13.3%, 33.3%, 40%, 93.3%, 100%, 6.7%, 26.7%, 53.3%, 60%, 73.3%, 20%, 46.7%, 66.7%, 80%, 86.7%), with a total of 21 components. Likewise, for the sequences encoded by the other four biochemical properties, each is also corresponding to 21 components. But for the sequence encoded by the solvent accessibility with only two states (“buried” or “exposed to solvent”), the encoded sequence is corresponding to only 14 components. Finally, by adding the 20 components of AAC [87] into the vector concerned, the total number of components thus obtained for a given protein is 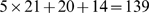; i.e., the protein can be formulated as a 139-D vector given by
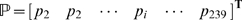
(2)where 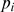 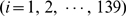 is the 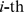 component of the protein 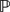. Of the 139 components, 119 are derived according to the codes of the above six biochemical and physicochemical features, and 20 are the AAC components of 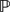.

### Nearest Neighbor Algorithm

With all samples represented by a feature vector, now it is possible for us to construct our predictor using the machine learning approach. The NN (Nearest Neighbor) algorithm is quite popular in pattern recognition community owing to its good performance and simple-to-use feature. According to the NN rule [88], the query sample should be assigned to the subset represented by its nearest neighbor. In this study, if the drug-target pair with the shortest distance is a positive sample, meaning that they can interact with each other, the sample for test is seen as a positive drug-target pair. Otherwise, the test sample is seen as a negative one.

There are many different definitions to measure the “nearness” for the NN algorithm, such as Euclidean distance, Hamming distance [89], and Mahalanobis distance [50], [90], [91]. In the current study, the following equation was adopted to measure the nearness between samples 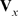 and 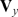


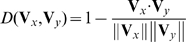
(3)where 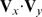 is the dot product of the two vectors, and 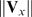 and 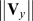 their modulus, respectively. When 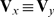 we have 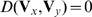, indicating the “distance” between these two sample vectors is zero and hence they have perfect or 100% similarity.

### Jackknife Cross-Validation Test

After constructing the drug-target interaction predictor, we have to evaluate its performance. In statistical prediction, the following three cross-validation methods are often used to examine a predictor for its effectiveness in practical application: independent dataset test, subsampling (K-fold cross-validation) test, and jackknife test [92]. However, as elucidated by [24] and demonstrated by Eq.50 in [93], among the three cross-validation methods, the jackknife test is deemed the most objective that can always yield a unique result for a given benchmark dataset, and hence has been increasingly used and widely recognized by investigators to examine the accuracy of various predictors (see, e.g. [51], [53], [54], [55], [56], [57], [59], [62], [63], [64], [94], [95], [96]).” Accordingly, in this study the jackknife cross-validation was adopted to calculate the success prediction rates as well.

### Maximum Relevance Minimum Redundancy (mRMR)

Although we've constructed the drug-target predictor based on the original feature set described above, it is possible to improve its performance with a better feature set. Apparently, not every feature in the feature set is equally relevant to the drug-target interaction. What's more, features may not be independent with each other. The “bad” will have negative impact on the accuracy and efficiency of the predictor, so it is possible to do the feature selection process to construct a more compact and effective feature set. The first step is using Maximum Relevance Minimum Redundancy (mRMR) [36] to do feature evaluation. Maximum Relevance Minimum Redundancy (mRMR) [39] was firstly developed for analysis of microarray data. It ranks each feature according to its relevance to the target and redundancy to other features. The better a feature is deemed to be, the higher the rank it will be assigned to. Mutual information (MI), denoted by *I* to indicate the dependence of two features used to quantify the relevance and redundancy. MI is defined as following:
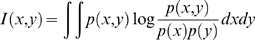
(4)


Based on MI, we can quantify relevance (*D*) and redundancy (*R*) as: 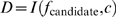
(5)

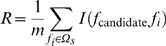
(6)where *f*_candidate_ is the feature to be calculated, and *c* is the target variable. By combining the above two equations to maximize relevance and minimize redundancy, the following mRMR function is constructed:
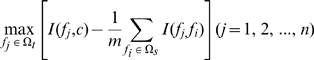
(7)where 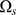 and 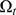 are the already-selected feature set and to-be-selected feature set, respectively, and *m* and *n* are the sizes of these two feature sets, respectively. The earlier a feature is selected, the better it would be though of. Finally, we can get an ordered feature list with a rank for every feature to indicate its importance in the feature set. In our study, the mRMR program is obtained from: http://research.janelia.org/peng/proj/mRMR/index.htm.

To calculate MI, the joint probabilistic density and the marginal probabilistic densities of the two vectors were used. A parameter *t* is introduced here to deal with these variables. Suppose mean to be the average value of one feature in all samples, and std to be the standard deviation, the feature of each sample would be classified into one of the three groups according to the boundaries: 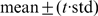. In our study, *t* was assigned to be 1.

### Incremental Feature Selection

As mentioned above, the importance of each feature is rated according to its rank in the mRMR analysis. The next step is to determine which features should be selected as the optimal feature set for our drug-target predictor. Here the IFS (Incremental Feature Selection) procedure is used to solve the problem. Each feature in the mRMR feature list was added one by one, and *N* different feature sets are obtained if the total feature number is *N*, while the *i*-th feature set is:
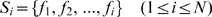
(8)


Based on each of the *N* feature sets, an NN algorithm predictor was constructed and tested with the jackknife cross-validation test. With all the *N* overall accurate rates calculated, we could draw an IFS curve with the index *i* to be the x-axis and the corresponding overall accurate rate to be the y-axis. Thus, 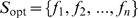 is regarded as the optimal feature set if the curve reach its peak where the value of its x-axis is 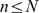.

Because four independent predictors are needed for the four different classes of drug-target pairs, the IFS analysis procedure will be processed four times with each for a specific predictor.

### Forward Feature Selection

To refine feature selection, the FFS (Forward Feature Selection) procedure based on the result of IFS was used. FFS is a feature selection method based on IFS results which tries every feature in the candidate feature set and adds the feature that achieves the highest prediction accuracy into the already-selected feature set in each goes. Suppose the IFS curve reaches its peak with apex as its x-axis, the initial FFS-selected feature set was constructed as:
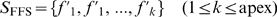
(9)


More features in FFS-to-be-selected feature set would be added into the FFS-selected feature set one by one. The FFS-to-be-selected feature set with *M* features covers the features with mRMR ranks between *k+1* and *k+1+M*, where *M* is a user-defined positive integer smaller than 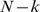 with *N* to be the size of the original feature set. In each round of FFS, each feature in FFS-to-be-selected feature set would be taken out and added to the FFS-selected feature set. Each predictor based on each new FFS-selected feature set would be tested, and the feature set obtained the highest overall accurate rate would be used as the new FFS-selected feature set. This process would be run for *M* times, until the FFS-to-be-selected feature set becomes a null set. An FFS curve similar to the IFS curve could be drawn with x-axis as the index and y-axis as the overall accurate rate.

In this study, FFS was run for each of the four benchmark datasets based on the corresponding IFS result. *M* for all these processes was set to 50, while *k* for each FFS was set to be the index of the point with the first maximum value (i.e. the maximum point with the smallest index) in the corresponding IFS curve.

## Results and Discussion

### Results of mRMR

To improve performance of the predictor of drug-target interaction, feature selection process was carried out. The first step of feature selection is feature evaluation. In this study, mRMR was used to evaluate every feature in original feature set. Listed in Online Supporting Information S5 are two kinds of outputs: the first one is the MaxRel list which shows ranks of features for their relevance to the target; the second is mRMR list showing the mRMR ranks according to the feature order satisfying Eq. 3. In this study, only the mRMR list was used as the results of feature evaluation. Since there are four groups of samples, mRMR was run four times with each for one of them.

### Results of IFS and FFS

**Figure 1:**
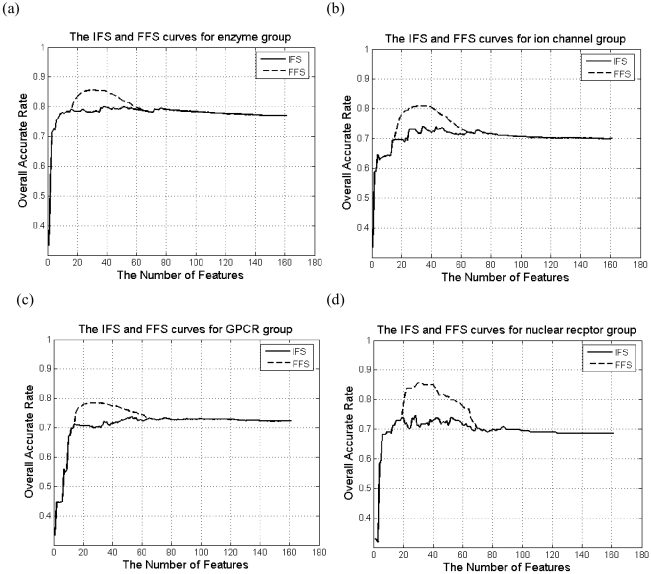
The IFS and FFS curves of the 4 groups. The detailed IFS curve with their corresponding FFS curve for (a) enzyme group, (b) ion channel group, (c) GPCR group, and (d) nuclear receptor group.

With the four mRMR lists, IFS was processed for each of the four sample groups, generating four IFS curves. Based on these results, we set *k* in FFS to be 16, 15, 14 and 19 for the data of enzymes, ion channels, GPCRs and nuclear receptors, respectively. Each of these figures is the index of the point of the first maximum value in the corresponding IFS curve. Shown in **Fig. 1** are the four IFS curves with their corresponding FFS curves. The peaks of the four FFS curves finally reach the overall success rates of 85.48% with 32 features, 80.78% with 37 features, 78.49% with 30 features, and 85.66% with 32 features for enzyme group, ion channel group, GPCR group and nuclear receptor groups, respectively.

Features selected by mRMR+FFS for the four different groups are quite different from each other, showing the intrinsic differences between them. Although there are more features for target than those for drug in the original feature set, more drug features were selected, showing the important role of drugs. Many of the selected target features are for protein secondary structure, especially for enzyme group (half of selected target features are for this). All types of features are selected in at least one group, showing that all biochemical and physicochemical features have their irreplaceable positions in drug-target interaction process.

For the details of the optimal feature-set outputs by FFS for the four benchmark datasets, see the Online Supporting Information S6.

### Discussions

For the specificity and promiscuity, we divided the drug-protein interactions into four groups according to the targets of drugs: enzymes, ion channels, GPCRs, and nuclear receptors. We used all the known drugs and target proteins in the gold standard data as training data to predict the potential interactions between all human proteins annotated as members of the four classes in KEGG genes and all compounds in KEGG ligands.

Enzyme recognition is the primary event involved in the interaction of proteins with other proteins and with small molecules such as metabolites and therapeutics. Predicting drug-enzyme interactions has direct application for completing genome annotations, finding enzymes for synthetic chemistry, and predicting drug specificity, promiscuity and pharmacology. It is suggested that the secondary structure information plays the major role in determining the drug-enzyme interactions activity. For example, cytochrome P450 (CYP) induction-mediated interaction is one of the major concerns in clinical practice and for the pharmaceutical industry [97]. Induction of CYP1A enzymes with a specific structure-stable state may activate some xenobiotics to their reactive metabolites, leading to toxicity [98], [99]. Amino acid composition and hydrophobicity also contribute considerably to these interactions. An insertion/deletion (I/D) polymorphism of the angiotensin I-converting enzyme (ACE) have an influence on the antihypertensive response, particularly when using ACE inhibitors (ACEI) [100], mirroring that the amino acid composition did contribute to the interactions. Hydrophobicity plays a role in determining the coefficients of drug-enzyme interaction energy with the application to drug screening as well as in silico target protein screening [101], [102].

The G-protein coupled receptor (GPCR) superfamily, which is comprised of estimated 600–1,000 members, is another largest known class of molecular targets with varieties of physiological activities and proven therapeutic value [103]. They are integral membrane proteins sharing a common global topology that consists of seven transmembrane alpha helices, intracellular C-terminal, an extracellular N-terminal, three intracellular loops and three extracellular loops [33], [44]. It is suggested that secondary structure and polarity would play a major role in determining the drug-GPCRs interactions activity. Small secondary structures such as helices and loops are identified as entities potentially involved in stabilizing interactions with ligands [33]. These motifs were situated mainly in the apical region of transmembrane segments and included a few extracellular residues [104]. Crystal structures of engineered human beta 2-adrenergic receptors (ARs) in complex with an inverse agonist ligand, carazolol, provide three-dimensional snapshots of an important G protein-coupled receptor (GPCR) with a beta-sheet structure and forms part of the chromophore-binding site [105]. GLIDA provides interaction data between GPCRs and their ligands, along with chemical information on the ligands, as well as biological information regarding GPCRs [106]. Some of the features reflect physical interactions that are responsible for the structural stability of the transmembrane, the formation of extensive networks of inter-helical H-bonds and sulfur-aromatic clusters that are spatially organized as “polarity”, the close packing of side-chains throughout the transmembrane domain. When more experimental 3D structures become available for GPCRs in the future, this will help building reliable models for a wider range of GPCRs that would be suitable for docking studies. Joint use of ligand-based chemogenomic and docking would certainly improve the prediction.

Ion channels are a large superfamily of membrane proteins that pass ions across membranes and are critical to diverse physiological functions in both excitable and nonexcitable cells and underlie many diseases. As a result, they are an important target class which is proven to be highly “druggable”. According to our analysis, secondary structure and polarity play the major role in determining the drug-ion channels interactions activity. Secondary structure controls the membrane potential and interrogates ion channels in different conformational states. The drug-ion channels interaction needs gated state where they can switch conformation between a closed and an open state [42], [43]. Simulations on model nanopores reveal that a narrow hydrophobic region can form a functionally closed gate in the channel and can be opened by either a small increase in pore radius or an increase in polarity [107], [108]. Nowadays, intense research is being conducted to develop new drugs acting selectively on ion channel subtypes and aimed at the understanding of the intimate drug–channel interaction [109].

Nuclear receptors (NR) are ligand-activated transcription factors that regulate the activation of a variety of important target genes, which are the most important drug targets in terms of potential therapeutic application. According to our results, secondary structure and polarizability play the major role in determining the drug-NRs interactions. The conservative motif of the NR is typically described as three stacked alpha-helical sheets. The helices that make up the “front” and “back” sheets are aligned parallel to one another. The helices in the middle sheet run across the two outer sheets and only occupy the space in the upper portion of the domain. The space in the lower part of the domain is relatively void of protein, and for most NRs, this creates an internal cavity for small-molecule ligands [110]. Hydrogen bonds with polarizability activity play a crucial role in protein-drug interactions (see, e.g., [11]). Our approaches and the results thus obtained could be used to demonstrate how nuclear hormone receptors form a network of direct interactions. And this network increases in complexity to describe the interactions with target genes as well as small molecules known to bind a receptor, enzyme, or transporter.

A comprehensive drug-target interaction network system has been established that contains four classifiers for predicting the drugable interaction of compounds with enzymes, ion-channels, GPCRs, and nuclear receptors, respectively. It is anticipated that the network predictor system may become a very useful tool for drug development. Particularly it may help us find new or potential drug-target interactions.

## Supporting Information

Online Supporting Information S1 The benchmark dataset for the drug-target enzyme interaction system. It contains 8,157 gene-drug pair samples, of which 2,719 are positive and 5,438 negative. The 1st column of the table indicates the nature of samples with 1 for positive and 2 for negative; the 2nd column shows the code of target gene; and the 3rd column shows the code of drug. All the detailed information for the genes and drugs listed here can be found in KEGG via their codes (Kanehisa, M., Goto, S., Hattori, M., Aoki-Kinoshita, K.F., Itoh, M., Kawashima, S., Katayama, T., Araki, M., Hirakawa, M. From genomics to chemical genomics: new developments in KEGG Nucleic Acids Research, 2006, 34: D354-357).
(6.30 MB DOC)

Online Supporting Information S2 The benchmark dataset for the drug-target ion channel interaction system. It contains 4,116 gene-drug pair samples, of which 1,372 are positive and 2,744 negative. The 1st column of the table indicates the nature of samples with 1 for positive and 2 for negative; the 2nd column shows the code of target gene; and the 3rd column shows the code of drug. All the detailed information for the genes and drugs listed here can be found in KEGG via their codes (see the caption of Online Supporting Information A for further explanation).
(3.35 MB DOC)

Online Supporting Information S3 The benchmark dataset for the drug-target GPCR interaction system. It contains 1,860 gene-drug pair samples, of which 620 are positive and 1,240 negative. The 1st column of the table indicates the nature of samples with 1 for positive and 2 for negative; the 2nd column shows the code of target gene; and the 3rd column shows the code of drug. All the detailed information for the genes and drugs listed here can be found in KEGG via their codes (see the caption of Online Supporting Information A for further explanation).
(1.53 MB DOC)

Online Supporting Information S4 The benchmark dataset for the drug-target nuclear receptor interaction system. It contains 258 gene-drug pair samples, of which 86 are positive and 172 negative. The 1st column of the table indicates the nature of samples with 1 for positive and 2 for negative; the 2nd column shows the code of target gene; and the 3rd column shows the code of drug. All the detailed information for the genes and drugs listed here can be found in KEGG via their codes (see the caption of Online Supporting Information A for further explanation).
(0.22 MB DOC)

Online Supporting Information S5 Output of Maximum Relevancy Minimum Redundancy (mRMR).
(1.02 MB DOC)

Online Supporting Information S6 The Results of Forward Feature Selection (FFS).
(0.12 MB DOC)
