# Supplementary material for: Predicting Drug-Target Interaction Networks Based on Functional Groups and Biological Features
Source: PLoS One. 2010 Mar 11;5(3):e9603. doi: 10.1371/journal.pone.0009603 (PMC2836373; doi:10.1371/journal.pone.0009603)
Supplement: Online Supporting Information S1 — The benchmark dataset for the drug-target enzyme interaction system. It contains 8,157 gene-drug pair samples, of which 2,719 are positive and 5,438 negative. The 1st column of the table indicates the nature of samples with 1 for positive and 2 for negative; the 2nd column shows the code of target gene; and the 3rd column shows the code of drug. All the detailed information for the genes and drugs listed here can be found in KEGG via their codes (Kanehisa, M., Goto, S., Hattori, M., Aoki-Kinoshita, K.F., Itoh, M., Kawashima, S., Katayama, T., Araki, M., Hirakawa, M. From genomics to chemical genomics: new developments in KEGG Nucleic Acids Research, 2006, 34: D354-357). (6.30 MB DOC) [file pone.0009603.s001.doc]

**Online Supporting Information S1**: The benchmark dataset for the drug-target enzyme interaction system. It contains 8,157 gene-drug pair samples, of which 2,719 are positive and 5,438 negative. The 1st column of the table indicates the nature of samples with 1 for positive and 2 for negative; the 2nd column shows the code of target gene; and the 3rd column shows the code of drug. All the detailed information for the genes and drugs listed here can be found in KEGG via their codes (Kanehisa, M., Goto, S., Hattori, M., Aoki-Kinoshita, K.F., Itoh, M., Kawashima, S., Katayama, T., Araki, M., Hirakawa, M. From genomics to chemical genomics: new developments in KEGG Nucleic Acids Research, 2006, 34: D354-357).

| **Group** | **Target Gene** | **Drug** |
| --- | --- | --- |
| 1 | hsa_10056 | D00021 |
| 1 | hsa_100 | D00037 |
| 1 | hsa_100 | D00155 |
| 1 | hsa_10188 | D01441 |
| 1 | hsa_10269 | D00279 |
| 1 | hsa_10279 | D00043 |
| 1 | hsa_10279 | D00160 |
| 1 | hsa_10295 | D00039 |
| 1 | hsa_10295 | D00065 |
| 1 | hsa_10327 | D00136 |
| 1 | hsa_10327 | D02323 |
| 1 | hsa_1033 | D00107 |
| 1 | hsa_1033 | D00184 |
| 1 | hsa_10461 | D01441 |
| 1 | hsa_10461 | D01977 |
| 1 | hsa_10461 | D03218 |
| 1 | hsa_10461 | D03350 |
| 1 | hsa_10461 | D04024 |
| 1 | hsa_10461 | D04025 |
| 1 | hsa_10549 | D00217 |
| 1 | hsa_10549 | D00577 |
| 1 | hsa_10549 | D03670 |
| 1 | hsa_1056 | D01223 |
| 1 | hsa_1056 | D04028 |
| 1 | hsa_10667 | D00021 |
| 1 | hsa_10720 | D01276 |
| 1 | hsa_10747 | D00043 |
| 1 | hsa_10747 | D00160 |
| 1 | hsa_107 | D00002 |
| 1 | hsa_107 | D00045 |
| 1 | hsa_107 | D02769 |
| 1 | hsa_10825 | D00900 |
| 1 | hsa_10825 | D00902 |
| 1 | hsa_10825 | D03829 |
| 1 | hsa_10846 | D00227 |
| 1 | hsa_10846 | D00231 |
| 1 | hsa_10846 | D00371 |
| 1 | hsa_10846 | D00417 |
| 1 | hsa_10846 | D00501 |
| 1 | hsa_10846 | D00528 |
| 1 | hsa_10846 | D00691 |
| 1 | hsa_10846 | D01133 |
| 1 | hsa_10846 | D01198 |
| 1 | hsa_10846 | D01690 |
| 1 | hsa_10846 | D01704 |
| 1 | hsa_10846 | D01712 |
| 1 | hsa_10846 | D02008 |
| 1 | hsa_10846 | D02017 |
| 1 | hsa_10846 | D02042 |
| 1 | hsa_10846 | D02229 |
| 1 | hsa_10846 | D02655 |
| 1 | hsa_10846 | D02731 |
| 1 | hsa_10858 | D00139 |
| 1 | hsa_10858 | D00225 |
| 1 | hsa_10858 | D00380 |
| 1 | hsa_10858 | D00394 |
| 1 | hsa_10858 | D00410 |
| 1 | hsa_10858 | D00437 |
| 1 | hsa_10858 | D00528 |
| 1 | hsa_10858 | D00542 |
| 1 | hsa_10858 | D00574 |
| 1 | hsa_10858 | D01071 |
| 1 | hsa_10858 | D03670 |
| 1 | hsa_108 | D00002 |
| 1 | hsa_10901 | D02335 |
| 1 | hsa_10924 | D00417 |
| 1 | hsa_10924 | D00501 |
| 1 | hsa_10924 | D00528 |
| 1 | hsa_10935 | D00217 |
| 1 | hsa_10935 | D00577 |
| 1 | hsa_10935 | D03670 |
| 1 | hsa_10941 | D01276 |
| 1 | hsa_109 | D00002 |
| 1 | hsa_10 | D00002 |
| 1 | hsa_10 | D00448 |
| 1 | hsa_11072 | D00107 |
| 1 | hsa_11072 | D00184 |
| 1 | hsa_111 | D00002 |
| 1 | hsa_11202 | D00043 |
| 1 | hsa_11202 | D00160 |
| 1 | hsa_11221 | D00107 |
| 1 | hsa_11221 | D00184 |
| 1 | hsa_11238 | D00218 |
| 1 | hsa_11238 | D00340 |
| 1 | hsa_11238 | D00518 |
| 1 | hsa_11238 | D00519 |
| 1 | hsa_11238 | D00538 |
| 1 | hsa_11238 | D00652 |
| 1 | hsa_11238 | D00653 |
| 1 | hsa_11238 | D00655 |
| 1 | hsa_11238 | D01196 |
| 1 | hsa_11266 | D00107 |
| 1 | hsa_11266 | D00184 |
| 1 | hsa_112 | D00002 |
| 1 | hsa_11330 | D00160 |
| 1 | hsa_11330 | D01346 |
| 1 | hsa_11343 | D01223 |
| 1 | hsa_113 | D00002 |
| 1 | hsa_1147 | D00448 |
| 1 | hsa_114 | D00002 |
| 1 | hsa_115 | D00002 |
| 1 | hsa_116447 | D01061 |
| 1 | hsa_116447 | D01432 |
| 1 | hsa_116447 | D01911 |
| 1 | hsa_116447 | D02168 |
| 1 | hsa_116447 | D02756 |
| 1 | hsa_116447 | D04031 |
| 1 | hsa_1178 | D01223 |
| 1 | hsa_1200 | D00043 |
| 1 | hsa_1215 | D00043 |
| 1 | hsa_1215 | D00160 |
| 1 | hsa_124 | D00002 |
| 1 | hsa_125 | D00002 |
| 1 | hsa_1267 | D00417 |
| 1 | hsa_1267 | D00501 |
| 1 | hsa_1267 | D00528 |
| 1 | hsa_126 | D00002 |
| 1 | hsa_128853 | D00107 |
| 1 | hsa_128853 | D00184 |
| 1 | hsa_128 | D00002 |
| 1 | hsa_129807 | D00900 |
| 1 | hsa_129807 | D00902 |
| 1 | hsa_129807 | D03829 |
| 1 | hsa_130399 | D01441 |
| 1 | hsa_1312 | D00781 |
| 1 | hsa_1312 | D00786 |
| 1 | hsa_131 | D00002 |
| 1 | hsa_132160 | D00107 |
| 1 | hsa_132160 | D00184 |
| 1 | hsa_132 | D00423 |
| 1 | hsa_132 | D02769 |
| 1 | hsa_133121 | D00417 |
| 1 | hsa_133121 | D00501 |
| 1 | hsa_133121 | D00528 |
| 1 | hsa_134510 | D00107 |
| 1 | hsa_134510 | D00184 |
| 1 | hsa_1360 | D00037 |
| 1 | hsa_1363 | D00052 |
| 1 | hsa_1374 | D02176 |
| 1 | hsa_1376 | D02176 |
| 1 | hsa_1384 | D02176 |
| 1 | hsa_13 | D01223 |
| 1 | hsa_13 | D02356 |
| 1 | hsa_142679 | D00107 |
| 1 | hsa_142679 | D00184 |
| 1 | hsa_1429 | D03798 |
| 1 | hsa_1431 | D00037 |
| 1 | hsa_1432 | D01840 |
| 1 | hsa_1432 | D03115 |
| 1 | hsa_1432 | D03736 |
| 1 | hsa_1436 | D01441 |
| 1 | hsa_1436 | D01977 |
| 1 | hsa_1436 | D03218 |
| 1 | hsa_1436 | D03350 |
| 1 | hsa_1436 | D04024 |
| 1 | hsa_1436 | D04025 |
| 1 | hsa_1445 | D01441 |
| 1 | hsa_150290 | D00107 |
| 1 | hsa_150290 | D00184 |
| 1 | hsa_1504 | D00160 |
| 1 | hsa_1504 | D01346 |
| 1 | hsa_151531 | D00584 |
| 1 | hsa_1537 | D03670 |
| 1 | hsa_1543 | D00217 |
| 1 | hsa_1543 | D00225 |
| 1 | hsa_1543 | D00380 |
| 1 | hsa_1543 | D00394 |
| 1 | hsa_1543 | D00437 |
| 1 | hsa_1543 | D00528 |
| 1 | hsa_1543 | D00542 |
| 1 | hsa_1543 | D00569 |
| 1 | hsa_1543 | D00574 |
| 1 | hsa_1543 | D00771 |
| 1 | hsa_1543 | D00960 |
| 1 | hsa_1543 | D00964 |
| 1 | hsa_1543 | D01071 |
| 1 | hsa_1543 | D01425 |
| 1 | hsa_1543 | D02451 |
| 1 | hsa_1543 | D03670 |
| 1 | hsa_1543 | D03778 |
| 1 | hsa_1543 | D03781 |
| 1 | hsa_1543 | D03784 |
| 1 | hsa_1544 | D00139 |
| 1 | hsa_1544 | D00217 |
| 1 | hsa_1544 | D00225 |
| 1 | hsa_1544 | D00283 |
| 1 | hsa_1544 | D00380 |
| 1 | hsa_1544 | D00394 |
| 1 | hsa_1544 | D00410 |
| 1 | hsa_1544 | D00437 |
| 1 | hsa_1544 | D00454 |
| 1 | hsa_1544 | D00528 |
| 1 | hsa_1544 | D00542 |
| 1 | hsa_1544 | D00563 |
| 1 | hsa_1544 | D00569 |
| 1 | hsa_1544 | D00574 |
| 1 | hsa_1544 | D00771 |
| 1 | hsa_1544 | D00960 |
| 1 | hsa_1544 | D00964 |
| 1 | hsa_1544 | D01071 |
| 1 | hsa_1544 | D01425 |
| 1 | hsa_1544 | D02451 |
| 1 | hsa_1544 | D03670 |
| 1 | hsa_1544 | D03778 |
| 1 | hsa_1544 | D03781 |
| 1 | hsa_1544 | D03784 |
| 1 | hsa_1545 | D00139 |
| 1 | hsa_1545 | D00225 |
| 1 | hsa_1545 | D00380 |
| 1 | hsa_1545 | D00394 |
| 1 | hsa_1545 | D00410 |
| 1 | hsa_1545 | D00437 |
| 1 | hsa_1545 | D00528 |
| 1 | hsa_1545 | D00542 |
| 1 | hsa_1545 | D00574 |
| 1 | hsa_1545 | D00960 |
| 1 | hsa_1545 | D00964 |
| 1 | hsa_1545 | D01071 |
| 1 | hsa_1545 | D01425 |
| 1 | hsa_1545 | D02451 |
| 1 | hsa_1545 | D03670 |
| 1 | hsa_1545 | D03778 |
| 1 | hsa_1545 | D03781 |
| 1 | hsa_1545 | D03784 |
| 1 | hsa_1548 | D00139 |
| 1 | hsa_1548 | D00225 |
| 1 | hsa_1548 | D00380 |
| 1 | hsa_1548 | D00394 |
| 1 | hsa_1548 | D00410 |
| 1 | hsa_1548 | D00437 |
| 1 | hsa_1548 | D00528 |
| 1 | hsa_1548 | D00542 |
| 1 | hsa_1548 | D00574 |
| 1 | hsa_1548 | D00960 |
| 1 | hsa_1548 | D00964 |
| 1 | hsa_1548 | D01071 |
| 1 | hsa_1548 | D01425 |
| 1 | hsa_1548 | D02451 |
| 1 | hsa_1548 | D03670 |
| 1 | hsa_1548 | D03778 |
| 1 | hsa_1548 | D03781 |
| 1 | hsa_1548 | D03784 |
| 1 | hsa_1549 | D00139 |
| 1 | hsa_1549 | D00225 |
| 1 | hsa_1549 | D00380 |
| 1 | hsa_1549 | D00394 |
| 1 | hsa_1549 | D00410 |
| 1 | hsa_1549 | D00437 |
| 1 | hsa_1549 | D00542 |
| 1 | hsa_1549 | D00574 |
| 1 | hsa_1549 | D00771 |
| 1 | hsa_1549 | D00960 |
| 1 | hsa_1549 | D00964 |
| 1 | hsa_1549 | D01071 |
| 1 | hsa_1549 | D01425 |
| 1 | hsa_1549 | D02451 |
| 1 | hsa_1549 | D03670 |
| 1 | hsa_1549 | D03778 |
| 1 | hsa_1549 | D03781 |
| 1 | hsa_1549 | D03784 |
| 1 | hsa_1551 | D00139 |
| 1 | hsa_1551 | D00225 |
| 1 | hsa_1551 | D00380 |
| 1 | hsa_1551 | D00394 |
| 1 | hsa_1551 | D00410 |
| 1 | hsa_1551 | D00437 |
| 1 | hsa_1551 | D00528 |
| 1 | hsa_1551 | D00542 |
| 1 | hsa_1551 | D00574 |
| 1 | hsa_1551 | D00960 |
| 1 | hsa_1551 | D00964 |
| 1 | hsa_1551 | D01071 |
| 1 | hsa_1551 | D01425 |
| 1 | hsa_1551 | D02451 |
| 1 | hsa_1551 | D03670 |
| 1 | hsa_1551 | D03778 |
| 1 | hsa_1551 | D03781 |
| 1 | hsa_1551 | D03784 |
| 1 | hsa_1553 | D00139 |
| 1 | hsa_1553 | D00225 |
| 1 | hsa_1553 | D00380 |
| 1 | hsa_1553 | D00394 |
| 1 | hsa_1553 | D00410 |
| 1 | hsa_1553 | D00437 |
| 1 | hsa_1553 | D00528 |
| 1 | hsa_1553 | D00542 |
| 1 | hsa_1553 | D00574 |
| 1 | hsa_1553 | D00960 |
| 1 | hsa_1553 | D00964 |
| 1 | hsa_1553 | D01071 |
| 1 | hsa_1553 | D01425 |
| 1 | hsa_1553 | D02451 |
| 1 | hsa_1553 | D03670 |
| 1 | hsa_1553 | D03778 |
| 1 | hsa_1553 | D03781 |
| 1 | hsa_1553 | D03784 |
| 1 | hsa_1555 | D00139 |
| 1 | hsa_1555 | D00225 |
| 1 | hsa_1555 | D00380 |
| 1 | hsa_1555 | D00394 |
| 1 | hsa_1555 | D00410 |
| 1 | hsa_1555 | D00437 |
| 1 | hsa_1555 | D00528 |
| 1 | hsa_1555 | D00542 |
| 1 | hsa_1555 | D00574 |
| 1 | hsa_1555 | D00960 |
| 1 | hsa_1555 | D00964 |
| 1 | hsa_1555 | D01071 |
| 1 | hsa_1555 | D01425 |
| 1 | hsa_1555 | D02451 |
| 1 | hsa_1555 | D03670 |
| 1 | hsa_1555 | D03778 |
| 1 | hsa_1555 | D03781 |
| 1 | hsa_1555 | D03784 |
| 1 | hsa_1557 | D00139 |
| 1 | hsa_1557 | D00225 |
| 1 | hsa_1557 | D00283 |
| 1 | hsa_1557 | D00293 |
| 1 | hsa_1557 | D00322 |
| 1 | hsa_1557 | D00380 |
| 1 | hsa_1557 | D00394 |
| 1 | hsa_1557 | D00410 |
| 1 | hsa_1557 | D00416 |
| 1 | hsa_1557 | D00437 |
| 1 | hsa_1557 | D00512 |
| 1 | hsa_1557 | D00528 |
| 1 | hsa_1557 | D00533 |
| 1 | hsa_1557 | D00536 |
| 1 | hsa_1557 | D00542 |
| 1 | hsa_1557 | D00574 |
| 1 | hsa_1557 | D00882 |
| 1 | hsa_1557 | D00960 |
| 1 | hsa_1557 | D00964 |
| 1 | hsa_1557 | D01071 |
| 1 | hsa_1557 | D01425 |
| 1 | hsa_1557 | D02451 |
| 1 | hsa_1557 | D03670 |
| 1 | hsa_1557 | D03778 |
| 1 | hsa_1557 | D03781 |
| 1 | hsa_1557 | D03784 |
| 1 | hsa_1558 | D00139 |
| 1 | hsa_1558 | D00225 |
| 1 | hsa_1558 | D00252 |
| 1 | hsa_1558 | D00380 |
| 1 | hsa_1558 | D00394 |
| 1 | hsa_1558 | D00410 |
| 1 | hsa_1558 | D00437 |
| 1 | hsa_1558 | D00528 |
| 1 | hsa_1558 | D00542 |
| 1 | hsa_1558 | D00574 |
| 1 | hsa_1558 | D00596 |
| 1 | hsa_1558 | D00960 |
| 1 | hsa_1558 | D00964 |
| 1 | hsa_1558 | D01071 |
| 1 | hsa_1558 | D01425 |
| 1 | hsa_1558 | D02356 |
| 1 | hsa_1558 | D02451 |
| 1 | hsa_1558 | D03670 |
| 1 | hsa_1558 | D03778 |
| 1 | hsa_1558 | D03781 |
| 1 | hsa_1558 | D03784 |
| 1 | hsa_1558 | D05341 |
| 1 | hsa_1559 | D00126 |
| 1 | hsa_1559 | D00139 |
| 1 | hsa_1559 | D00225 |
| 1 | hsa_1559 | D00322 |
| 1 | hsa_1559 | D00380 |
| 1 | hsa_1559 | D00394 |
| 1 | hsa_1559 | D00410 |
| 1 | hsa_1559 | D00416 |
| 1 | hsa_1559 | D00512 |
| 1 | hsa_1559 | D00528 |
| 1 | hsa_1559 | D00542 |
| 1 | hsa_1559 | D00567 |
| 1 | hsa_1559 | D00574 |
| 1 | hsa_1559 | D00593 |
| 1 | hsa_1559 | D00882 |
| 1 | hsa_1559 | D00960 |
| 1 | hsa_1559 | D00964 |
| 1 | hsa_1559 | D01071 |
| 1 | hsa_1559 | D01425 |
| 1 | hsa_1559 | D02451 |
| 1 | hsa_1559 | D03670 |
| 1 | hsa_1559 | D03778 |
| 1 | hsa_1559 | D03781 |
| 1 | hsa_1559 | D03784 |
| 1 | hsa_1562 | D00139 |
| 1 | hsa_1562 | D00225 |
| 1 | hsa_1562 | D00380 |
| 1 | hsa_1562 | D00394 |
| 1 | hsa_1562 | D00410 |
| 1 | hsa_1562 | D00437 |
| 1 | hsa_1562 | D00528 |
| 1 | hsa_1562 | D00542 |
| 1 | hsa_1562 | D00574 |
| 1 | hsa_1562 | D00960 |
| 1 | hsa_1562 | D00964 |
| 1 | hsa_1562 | D01071 |
| 1 | hsa_1562 | D01425 |
| 1 | hsa_1562 | D02451 |
| 1 | hsa_1562 | D03670 |
| 1 | hsa_1562 | D03778 |
| 1 | hsa_1562 | D03781 |
| 1 | hsa_1562 | D03784 |
| 1 | hsa_1565 | D00139 |
| 1 | hsa_1565 | D00217 |
| 1 | hsa_1565 | D00225 |
| 1 | hsa_1565 | D00234 |
| 1 | hsa_1565 | D00274 |
| 1 | hsa_1565 | D00283 |
| 1 | hsa_1565 | D00300 |
| 1 | hsa_1565 | D00380 |
| 1 | hsa_1565 | D00394 |
| 1 | hsa_1565 | D00410 |
| 1 | hsa_1565 | D00437 |
| 1 | hsa_1565 | D00454 |
| 1 | hsa_1565 | D00503 |
| 1 | hsa_1565 | D00521 |
| 1 | hsa_1565 | D00528 |
| 1 | hsa_1565 | D00542 |
| 1 | hsa_1565 | D00563 |
| 1 | hsa_1565 | D00574 |
| 1 | hsa_1565 | D00960 |
| 1 | hsa_1565 | D00964 |
| 1 | hsa_1565 | D01071 |
| 1 | hsa_1565 | D01164 |
| 1 | hsa_1565 | D01425 |
| 1 | hsa_1565 | D02342 |
| 1 | hsa_1565 | D02451 |
| 1 | hsa_1565 | D02671 |
| 1 | hsa_1565 | D03670 |
| 1 | hsa_1565 | D03778 |
| 1 | hsa_1565 | D03781 |
| 1 | hsa_1565 | D03784 |
| 1 | hsa_1571 | D00131 |
| 1 | hsa_1571 | D00139 |
| 1 | hsa_1571 | D00217 |
| 1 | hsa_1571 | D00225 |
| 1 | hsa_1571 | D00380 |
| 1 | hsa_1571 | D00394 |
| 1 | hsa_1571 | D00410 |
| 1 | hsa_1571 | D00437 |
| 1 | hsa_1571 | D00528 |
| 1 | hsa_1571 | D00543 |
| 1 | hsa_1571 | D00544 |
| 1 | hsa_1571 | D00547 |
| 1 | hsa_1571 | D00574 |
| 1 | hsa_1571 | D00771 |
| 1 | hsa_1571 | D00960 |
| 1 | hsa_1571 | D00964 |
| 1 | hsa_1571 | D01071 |
| 1 | hsa_1571 | D01425 |
| 1 | hsa_1571 | D02451 |
| 1 | hsa_1571 | D03670 |
| 1 | hsa_1571 | D03778 |
| 1 | hsa_1571 | D03781 |
| 1 | hsa_1571 | D03784 |
| 1 | hsa_1572 | D00139 |
| 1 | hsa_1572 | D00225 |
| 1 | hsa_1572 | D00380 |
| 1 | hsa_1572 | D00394 |
| 1 | hsa_1572 | D00410 |
| 1 | hsa_1572 | D00437 |
| 1 | hsa_1572 | D00528 |
| 1 | hsa_1572 | D00542 |
| 1 | hsa_1572 | D00574 |
| 1 | hsa_1572 | D00960 |
| 1 | hsa_1572 | D00964 |
| 1 | hsa_1572 | D01071 |
| 1 | hsa_1572 | D01425 |
| 1 | hsa_1572 | D02451 |
| 1 | hsa_1572 | D03670 |
| 1 | hsa_1572 | D03778 |
| 1 | hsa_1572 | D03781 |
| 1 | hsa_1572 | D03784 |
| 1 | hsa_1573 | D00139 |
| 1 | hsa_1573 | D00225 |
| 1 | hsa_1573 | D00380 |
| 1 | hsa_1573 | D00394 |
| 1 | hsa_1573 | D00410 |
| 1 | hsa_1573 | D00437 |
| 1 | hsa_1573 | D00528 |
| 1 | hsa_1573 | D00542 |
| 1 | hsa_1573 | D00574 |
| 1 | hsa_1573 | D00960 |
| 1 | hsa_1573 | D00964 |
| 1 | hsa_1573 | D01071 |
| 1 | hsa_1573 | D01425 |
| 1 | hsa_1573 | D02451 |
| 1 | hsa_1573 | D03670 |
| 1 | hsa_1573 | D03778 |
| 1 | hsa_1573 | D03781 |
| 1 | hsa_1573 | D03784 |
| 1 | hsa_1576 | D00136 |
| 1 | hsa_1576 | D00139 |
| 1 | hsa_1576 | D00225 |
| 1 | hsa_1576 | D00234 |
| 1 | hsa_1576 | D00252 |
| 1 | hsa_1576 | D00274 |
| 1 | hsa_1576 | D00283 |
| 1 | hsa_1576 | D00293 |
| 1 | hsa_1576 | D00364 |
| 1 | hsa_1576 | D00380 |
| 1 | hsa_1576 | D00387 |
| 1 | hsa_1576 | D00394 |
| 1 | hsa_1576 | D00410 |
| 1 | hsa_1576 | D00434 |
| 1 | hsa_1576 | D00437 |
| 1 | hsa_1576 | D00503 |
| 1 | hsa_1576 | D00521 |
| 1 | hsa_1576 | D00528 |
| 1 | hsa_1576 | D00542 |
| 1 | hsa_1576 | D00550 |
| 1 | hsa_1576 | D00563 |
| 1 | hsa_1576 | D00574 |
| 1 | hsa_1576 | D00960 |
| 1 | hsa_1576 | D00964 |
| 1 | hsa_1576 | D01071 |
| 1 | hsa_1576 | D01425 |
| 1 | hsa_1576 | D01973 |
| 1 | hsa_1576 | D02356 |
| 1 | hsa_1576 | D02451 |
| 1 | hsa_1576 | D03670 |
| 1 | hsa_1576 | D03778 |
| 1 | hsa_1576 | D03781 |
| 1 | hsa_1576 | D03784 |
| 1 | hsa_1577 | D00139 |
| 1 | hsa_1577 | D00225 |
| 1 | hsa_1577 | D00380 |
| 1 | hsa_1577 | D00394 |
| 1 | hsa_1577 | D00410 |
| 1 | hsa_1577 | D00437 |
| 1 | hsa_1577 | D00528 |
| 1 | hsa_1577 | D00542 |
| 1 | hsa_1577 | D00574 |
| 1 | hsa_1577 | D00960 |
| 1 | hsa_1577 | D00964 |
| 1 | hsa_1577 | D01071 |
| 1 | hsa_1577 | D01425 |
| 1 | hsa_1577 | D02451 |
| 1 | hsa_1577 | D03670 |
| 1 | hsa_1577 | D03778 |
| 1 | hsa_1577 | D03781 |
| 1 | hsa_1577 | D03784 |
| 1 | hsa_1579 | D00139 |
| 1 | hsa_1579 | D00225 |
| 1 | hsa_1579 | D00380 |
| 1 | hsa_1579 | D00394 |
| 1 | hsa_1579 | D00410 |
| 1 | hsa_1579 | D00437 |
| 1 | hsa_1579 | D00528 |
| 1 | hsa_1579 | D00542 |
| 1 | hsa_1579 | D00574 |
| 1 | hsa_1579 | D01071 |
| 1 | hsa_1580 | D00139 |
| 1 | hsa_1580 | D00225 |
| 1 | hsa_1580 | D00380 |
| 1 | hsa_1580 | D00394 |
| 1 | hsa_1580 | D00410 |
| 1 | hsa_1580 | D00437 |
| 1 | hsa_1580 | D00528 |
| 1 | hsa_1580 | D00542 |
| 1 | hsa_1580 | D00574 |
| 1 | hsa_1580 | D00960 |
| 1 | hsa_1580 | D00964 |
| 1 | hsa_1580 | D01071 |
| 1 | hsa_1580 | D01425 |
| 1 | hsa_1580 | D02451 |
| 1 | hsa_1580 | D03670 |
| 1 | hsa_1580 | D03778 |
| 1 | hsa_1580 | D03781 |
| 1 | hsa_1580 | D03784 |
| 1 | hsa_1581 | D00139 |
| 1 | hsa_1581 | D00225 |
| 1 | hsa_1581 | D00380 |
| 1 | hsa_1581 | D00394 |
| 1 | hsa_1581 | D00410 |
| 1 | hsa_1581 | D00437 |
| 1 | hsa_1581 | D00528 |
| 1 | hsa_1581 | D00542 |
| 1 | hsa_1581 | D00574 |
| 1 | hsa_1581 | D01071 |
| 1 | hsa_1581 | D03670 |
| 1 | hsa_1582 | D00139 |
| 1 | hsa_1582 | D00225 |
| 1 | hsa_1582 | D00380 |
| 1 | hsa_1582 | D00394 |
| 1 | hsa_1582 | D00410 |
| 1 | hsa_1582 | D00437 |
| 1 | hsa_1582 | D00528 |
| 1 | hsa_1582 | D00542 |
| 1 | hsa_1582 | D00574 |
| 1 | hsa_1582 | D01071 |
| 1 | hsa_1582 | D03670 |
| 1 | hsa_1583 | D00139 |
| 1 | hsa_1583 | D00225 |
| 1 | hsa_1583 | D00380 |
| 1 | hsa_1583 | D00394 |
| 1 | hsa_1583 | D00437 |
| 1 | hsa_1583 | D00528 |
| 1 | hsa_1583 | D00542 |
| 1 | hsa_1583 | D00574 |
| 1 | hsa_1583 | D01071 |
| 1 | hsa_1583 | D03670 |
| 1 | hsa_1584 | D00139 |
| 1 | hsa_1584 | D00225 |
| 1 | hsa_1584 | D00380 |
| 1 | hsa_1584 | D00394 |
| 1 | hsa_1584 | D00410 |
| 1 | hsa_1584 | D00437 |
| 1 | hsa_1584 | D00528 |
| 1 | hsa_1584 | D00542 |
| 1 | hsa_1584 | D00574 |
| 1 | hsa_1584 | D01071 |
| 1 | hsa_1584 | D03670 |
| 1 | hsa_1585 | D00139 |
| 1 | hsa_1585 | D00225 |
| 1 | hsa_1585 | D00380 |
| 1 | hsa_1585 | D00394 |
| 1 | hsa_1585 | D00528 |
| 1 | hsa_1585 | D00542 |
| 1 | hsa_1585 | D00574 |
| 1 | hsa_1585 | D01071 |
| 1 | hsa_1585 | D03670 |
| 1 | hsa_1586 | D00139 |
| 1 | hsa_1586 | D00225 |
| 1 | hsa_1586 | D00380 |
| 1 | hsa_1586 | D00394 |
| 1 | hsa_1586 | D00410 |
| 1 | hsa_1586 | D00437 |
| 1 | hsa_1586 | D00528 |
| 1 | hsa_1586 | D00542 |
| 1 | hsa_1586 | D00574 |
| 1 | hsa_1586 | D01071 |
| 1 | hsa_1586 | D03670 |
| 1 | hsa_1586 | D03781 |
| 1 | hsa_1586 | D03784 |
| 1 | hsa_1588 | D00139 |
| 1 | hsa_1588 | D00153 |
| 1 | hsa_1588 | D00225 |
| 1 | hsa_1588 | D00380 |
| 1 | hsa_1588 | D00394 |
| 1 | hsa_1588 | D00410 |
| 1 | hsa_1588 | D00437 |
| 1 | hsa_1588 | D00528 |
| 1 | hsa_1588 | D00542 |
| 1 | hsa_1588 | D00574 |
| 1 | hsa_1588 | D00960 |
| 1 | hsa_1588 | D00963 |
| 1 | hsa_1588 | D00964 |
| 1 | hsa_1588 | D01071 |
| 1 | hsa_1588 | D01425 |
| 1 | hsa_1588 | D02451 |
| 1 | hsa_1588 | D03670 |
| 1 | hsa_1588 | D03778 |
| 1 | hsa_1588 | D03781 |
| 1 | hsa_1588 | D03784 |
| 1 | hsa_1589 | D00139 |
| 1 | hsa_1589 | D00225 |
| 1 | hsa_1589 | D00380 |
| 1 | hsa_1589 | D00394 |
| 1 | hsa_1589 | D00410 |
| 1 | hsa_1589 | D00437 |
| 1 | hsa_1589 | D00528 |
| 1 | hsa_1589 | D00542 |
| 1 | hsa_1589 | D01071 |
| 1 | hsa_1589 | D03670 |
| 1 | hsa_1593 | D00139 |
| 1 | hsa_1593 | D00188 |
| 1 | hsa_1593 | D00225 |
| 1 | hsa_1593 | D00380 |
| 1 | hsa_1593 | D00394 |
| 1 | hsa_1593 | D00410 |
| 1 | hsa_1593 | D00437 |
| 1 | hsa_1593 | D00528 |
| 1 | hsa_1593 | D00542 |
| 1 | hsa_1593 | D00574 |
| 1 | hsa_1593 | D01071 |
| 1 | hsa_1593 | D03670 |
| 1 | hsa_1594 | D00139 |
| 1 | hsa_1594 | D00187 |
| 1 | hsa_1594 | D00225 |
| 1 | hsa_1594 | D00380 |
| 1 | hsa_1594 | D00394 |
| 1 | hsa_1594 | D00410 |
| 1 | hsa_1594 | D00437 |
| 1 | hsa_1594 | D00528 |
| 1 | hsa_1594 | D00542 |
| 1 | hsa_1594 | D00574 |
| 1 | hsa_1594 | D01071 |
| 1 | hsa_1594 | D03670 |
| 1 | hsa_1595 | D00139 |
| 1 | hsa_1595 | D00225 |
| 1 | hsa_1595 | D00380 |
| 1 | hsa_1595 | D00394 |
| 1 | hsa_1595 | D00410 |
| 1 | hsa_1595 | D00437 |
| 1 | hsa_1595 | D00528 |
| 1 | hsa_1595 | D00542 |
| 1 | hsa_1595 | D00574 |
| 1 | hsa_1595 | D01071 |
| 1 | hsa_1595 | D02556 |
| 1 | hsa_1595 | D03670 |
| 1 | hsa_1610 | D00005 |
| 1 | hsa_1610 | D00038 |
| 1 | hsa_1621 | D00131 |
| 1 | hsa_1621 | D03787 |
| 1 | hsa_1633 | D02368 |
| 1 | hsa_1636 | D00198 |
| 1 | hsa_1636 | D00251 |
| 1 | hsa_1636 | D00362 |
| 1 | hsa_1636 | D00383 |
| 1 | hsa_1636 | D00421 |
| 1 | hsa_1636 | D00459 |
| 1 | hsa_1636 | D00620 |
| 1 | hsa_1636 | D00621 |
| 1 | hsa_1636 | D00622 |
| 1 | hsa_1636 | D00623 |
| 1 | hsa_1636 | D00624 |
| 1 | hsa_1636 | D01069 |
| 1 | hsa_1636 | D01119 |
| 1 | hsa_1636 | D01549 |
| 1 | hsa_1636 | D01667 |
| 1 | hsa_1636 | D01900 |
| 1 | hsa_1636 | D03077 |
| 1 | hsa_1636 | D03440 |
| 1 | hsa_1636 | D03752 |
| 1 | hsa_1636 | D03753 |
| 1 | hsa_1636 | D03756 |
| 1 | hsa_1636 | D03758 |
| 1 | hsa_1636 | D03760 |
| 1 | hsa_1636 | D03763 |
| 1 | hsa_1636 | D03765 |
| 1 | hsa_1636 | D03767 |
| 1 | hsa_1636 | D03769 |
| 1 | hsa_1636 | D03772 |
| 1 | hsa_1636 | D03773 |
| 1 | hsa_1636 | D03775 |
| 1 | hsa_1636 | D03776 |
| 1 | hsa_1645 | D00136 |
| 1 | hsa_1675 | D00043 |
| 1 | hsa_1675 | D00160 |
| 1 | hsa_1719 | D00142 |
| 1 | hsa_1719 | D00145 |
| 1 | hsa_1719 | D00285 |
| 1 | hsa_1719 | D00488 |
| 1 | hsa_1719 | D02115 |
| 1 | hsa_1719 | D02487 |
| 1 | hsa_1719 | D06238 |
| 1 | hsa_1723 | D00055 |
| 1 | hsa_1725 | D00002 |
| 1 | hsa_1728 | D00005 |
| 1 | hsa_1728 | D00208 |
| 1 | hsa_1728 | D03798 |
| 1 | hsa_1800 | D02194 |
| 1 | hsa_1803 | D00043 |
| 1 | hsa_1806 | D00005 |
| 1 | hsa_1806 | D00027 |
| 1 | hsa_1806 | D00584 |
| 1 | hsa_1843 | D00107 |
| 1 | hsa_1843 | D00184 |
| 1 | hsa_1844 | D00107 |
| 1 | hsa_1844 | D00184 |
| 1 | hsa_1845 | D00107 |
| 1 | hsa_1845 | D00184 |
| 1 | hsa_1846 | D00107 |
| 1 | hsa_1846 | D00184 |
| 1 | hsa_1847 | D00107 |
| 1 | hsa_1847 | D00184 |
| 1 | hsa_1848 | D00107 |
| 1 | hsa_1848 | D00184 |
| 1 | hsa_1849 | D00107 |
| 1 | hsa_1849 | D00184 |
| 1 | hsa_1850 | D00107 |
| 1 | hsa_1850 | D00184 |
| 1 | hsa_1852 | D00107 |
| 1 | hsa_1852 | D00184 |
| 1 | hsa_1890 | D01223 |
| 1 | hsa_189 | D00369 |
| 1 | hsa_18 | D00332 |
| 1 | hsa_18 | D00535 |
| 1 | hsa_191 | D00002 |
| 1 | hsa_1956 | D01441 |
| 1 | hsa_1956 | D01977 |
| 1 | hsa_1956 | D03218 |
| 1 | hsa_1956 | D03350 |
| 1 | hsa_1956 | D04024 |
| 1 | hsa_1956 | D04025 |
| 1 | hsa_196883 | D00002 |
| 1 | hsa_1969 | D01441 |
| 1 | hsa_1969 | D01977 |
| 1 | hsa_1969 | D03218 |
| 1 | hsa_1969 | D03350 |
| 1 | hsa_1969 | D04024 |
| 1 | hsa_1969 | D04025 |
| 1 | hsa_1990 | D00160 |
| 1 | hsa_1991 | D00160 |
| 1 | hsa_1991 | D01918 |
| 1 | hsa_1991 | D03788 |
| 1 | hsa_199974 | D00139 |
| 1 | hsa_199974 | D00225 |
| 1 | hsa_199974 | D00380 |
| 1 | hsa_199974 | D00394 |
| 1 | hsa_199974 | D00410 |
| 1 | hsa_199974 | D00437 |
| 1 | hsa_199974 | D00528 |
| 1 | hsa_199974 | D00542 |
| 1 | hsa_199974 | D00574 |
| 1 | hsa_199974 | D00960 |
| 1 | hsa_199974 | D00964 |
| 1 | hsa_199974 | D01071 |
| 1 | hsa_199974 | D01425 |
| 1 | hsa_199974 | D02451 |
| 1 | hsa_199974 | D03670 |
| 1 | hsa_199974 | D03778 |
| 1 | hsa_199974 | D03781 |
| 1 | hsa_199974 | D03784 |
| 1 | hsa_2041 | D01441 |
| 1 | hsa_2041 | D01977 |
| 1 | hsa_2041 | D03218 |
| 1 | hsa_2041 | D03350 |
| 1 | hsa_2041 | D04024 |
| 1 | hsa_2041 | D04025 |
| 1 | hsa_2042 | D01441 |
| 1 | hsa_2042 | D01977 |
| 1 | hsa_2042 | D03218 |
| 1 | hsa_2042 | D03350 |
| 1 | hsa_2042 | D04024 |
| 1 | hsa_2042 | D04025 |
| 1 | hsa_2043 | D01441 |
| 1 | hsa_2043 | D01977 |
| 1 | hsa_2043 | D03218 |
| 1 | hsa_2043 | D03350 |
| 1 | hsa_2043 | D04024 |
| 1 | hsa_2043 | D04025 |
| 1 | hsa_2044 | D01441 |
| 1 | hsa_2044 | D01977 |
| 1 | hsa_2044 | D03218 |
| 1 | hsa_2044 | D03350 |
| 1 | hsa_2044 | D04024 |
| 1 | hsa_2044 | D04025 |
| 1 | hsa_2045 | D01441 |
| 1 | hsa_2045 | D01977 |
| 1 | hsa_2045 | D03218 |
| 1 | hsa_2045 | D03350 |
| 1 | hsa_2045 | D04024 |
| 1 | hsa_2045 | D04025 |
| 1 | hsa_2046 | D01441 |
| 1 | hsa_2046 | D01977 |
| 1 | hsa_2046 | D03218 |
| 1 | hsa_2046 | D03350 |
| 1 | hsa_2046 | D04024 |
| 1 | hsa_2046 | D04025 |
| 1 | hsa_2047 | D01441 |
| 1 | hsa_2047 | D01977 |
| 1 | hsa_2047 | D03218 |
| 1 | hsa_2047 | D03350 |
| 1 | hsa_2047 | D04024 |
| 1 | hsa_2047 | D04025 |
| 1 | hsa_2048 | D01441 |
| 1 | hsa_2048 | D01977 |
| 1 | hsa_2048 | D03218 |
| 1 | hsa_2048 | D03350 |
| 1 | hsa_2048 | D04024 |
| 1 | hsa_2048 | D04025 |
| 1 | hsa_2049 | D01441 |
| 1 | hsa_2049 | D01977 |
| 1 | hsa_2049 | D03218 |
| 1 | hsa_2049 | D03350 |
| 1 | hsa_2049 | D04024 |
| 1 | hsa_2049 | D04025 |
| 1 | hsa_2050 | D01441 |
| 1 | hsa_2050 | D01977 |
| 1 | hsa_2050 | D03218 |
| 1 | hsa_2050 | D03350 |
| 1 | hsa_2050 | D04024 |
| 1 | hsa_2050 | D04025 |
| 1 | hsa_2051 | D01441 |
| 1 | hsa_2051 | D01977 |
| 1 | hsa_2051 | D03218 |
| 1 | hsa_2051 | D03350 |
| 1 | hsa_2051 | D04024 |
| 1 | hsa_2051 | D04025 |
| 1 | hsa_2058 | D00035 |
| 1 | hsa_2064 | D01441 |
| 1 | hsa_2064 | D01977 |
| 1 | hsa_2064 | D03218 |
| 1 | hsa_2064 | D03350 |
| 1 | hsa_2064 | D04024 |
| 1 | hsa_2064 | D04025 |
| 1 | hsa_2065 | D01441 |
| 1 | hsa_2065 | D01977 |
| 1 | hsa_2065 | D03218 |
| 1 | hsa_2065 | D03350 |
| 1 | hsa_2065 | D04024 |
| 1 | hsa_2065 | D04025 |
| 1 | hsa_2066 | D01441 |
| 1 | hsa_2066 | D01977 |
| 1 | hsa_2066 | D03218 |
| 1 | hsa_2066 | D03350 |
| 1 | hsa_2066 | D04024 |
| 1 | hsa_2066 | D04025 |
| 1 | hsa_2098 | D00043 |
| 1 | hsa_2098 | D01223 |
| 1 | hsa_2135 | D01828 |
| 1 | hsa_2147 | D00270 |
| 1 | hsa_2147 | D00494 |
| 1 | hsa_2147 | D00560 |
| 1 | hsa_2147 | D00733 |
| 1 | hsa_2147 | D01981 |
| 1 | hsa_2147 | D02335 |
| 1 | hsa_2147 | D03722 |
| 1 | hsa_2147 | D03728 |
| 1 | hsa_2155 | D00043 |
| 1 | hsa_2155 | D00160 |
| 1 | hsa_2155 | D02335 |
| 1 | hsa_2158 | D00043 |
| 1 | hsa_2158 | D00160 |
| 1 | hsa_2158 | D02335 |
| 1 | hsa_2159 | D00043 |
| 1 | hsa_2159 | D00160 |
| 1 | hsa_2159 | D01844 |
| 1 | hsa_2159 | D02335 |
| 1 | hsa_2159 | D04029 |
| 1 | hsa_2160 | D00043 |
| 1 | hsa_2160 | D00160 |
| 1 | hsa_2161 | D00043 |
| 1 | hsa_2161 | D00160 |
| 1 | hsa_216 | D00002 |
| 1 | hsa_216 | D00094 |
| 1 | hsa_216 | D00131 |
| 1 | hsa_217 | D00002 |
| 1 | hsa_217 | D00131 |
| 1 | hsa_2180 | D02769 |
| 1 | hsa_2185 | D01441 |
| 1 | hsa_218 | D00131 |
| 1 | hsa_2193 | D00021 |
| 1 | hsa_219 | D00131 |
| 1 | hsa_2224 | D01968 |
| 1 | hsa_2232 | D00005 |
| 1 | hsa_2235 | D03670 |
| 1 | hsa_223 | D00131 |
| 1 | hsa_2241 | D01441 |
| 1 | hsa_2242 | D01441 |
| 1 | hsa_224 | D00131 |
| 1 | hsa_225689 | D01840 |
| 1 | hsa_225689 | D03115 |
| 1 | hsa_225689 | D03736 |
| 1 | hsa_2260 | D01441 |
| 1 | hsa_2260 | D01977 |
| 1 | hsa_2260 | D03218 |
| 1 | hsa_2260 | D03350 |
| 1 | hsa_2260 | D04024 |
| 1 | hsa_2260 | D04025 |
| 1 | hsa_2261 | D01441 |
| 1 | hsa_2261 | D01977 |
| 1 | hsa_2261 | D03218 |
| 1 | hsa_2261 | D03350 |
| 1 | hsa_2261 | D04024 |
| 1 | hsa_2261 | D04025 |
| 1 | hsa_2263 | D01441 |
| 1 | hsa_2263 | D01977 |
| 1 | hsa_2263 | D03218 |
| 1 | hsa_2263 | D03350 |
| 1 | hsa_2263 | D04024 |
| 1 | hsa_2263 | D04025 |
| 1 | hsa_2264 | D01441 |
| 1 | hsa_2264 | D01977 |
| 1 | hsa_2264 | D03218 |
| 1 | hsa_2264 | D03350 |
| 1 | hsa_2264 | D04024 |
| 1 | hsa_2264 | D04025 |
| 1 | hsa_2280 | D00753 |
| 1 | hsa_22843 | D00107 |
| 1 | hsa_22843 | D00184 |
| 1 | hsa_22954 | D00283 |
| 1 | hsa_22954 | D00451 |
| 1 | hsa_22954 | D00513 |
| 1 | hsa_22954 | D00726 |
| 1 | hsa_22978 | D00423 |
| 1 | hsa_22978 | D00501 |
| 1 | hsa_23035 | D00107 |
| 1 | hsa_23035 | D00184 |
| 1 | hsa_231 | D00037 |
| 1 | hsa_231 | D00136 |
| 1 | hsa_231 | D01688 |
| 1 | hsa_231 | D01715 |
| 1 | hsa_231 | D01842 |
| 1 | hsa_231 | D02323 |
| 1 | hsa_231 | D02328 |
| 1 | hsa_231 | D02835 |
| 1 | hsa_231 | D03803 |
| 1 | hsa_231 | D03805 |
| 1 | hsa_231 | D03806 |
| 1 | hsa_231 | D03807 |
| 1 | hsa_2321 | D01441 |
| 1 | hsa_2321 | D01977 |
| 1 | hsa_2321 | D03218 |
| 1 | hsa_2321 | D03350 |
| 1 | hsa_2321 | D04024 |
| 1 | hsa_2321 | D04025 |
| 1 | hsa_2322 | D01441 |
| 1 | hsa_2322 | D01977 |
| 1 | hsa_2322 | D03218 |
| 1 | hsa_2322 | D03350 |
| 1 | hsa_2322 | D04024 |
| 1 | hsa_2322 | D04025 |
| 1 | hsa_23236 | D00417 |
| 1 | hsa_23236 | D00501 |
| 1 | hsa_23236 | D00528 |
| 1 | hsa_23239 | D00107 |
| 1 | hsa_23239 | D00184 |
| 1 | hsa_2324 | D01441 |
| 1 | hsa_2324 | D01977 |
| 1 | hsa_2324 | D03218 |
| 1 | hsa_2324 | D03350 |
| 1 | hsa_2324 | D04024 |
| 1 | hsa_2324 | D04025 |
| 1 | hsa_2326 | D00005 |
| 1 | hsa_2326 | D00401 |
| 1 | hsa_2328 | D00120 |
| 1 | hsa_2328 | D00401 |
| 1 | hsa_2339 | D03720 |
| 1 | hsa_2342 | D03720 |
| 1 | hsa_23430 | D00043 |
| 1 | hsa_23430 | D00160 |
| 1 | hsa_23436 | D00043 |
| 1 | hsa_23436 | D00160 |
| 1 | hsa_23475 | D00049 |
| 1 | hsa_2356 | D00070 |
| 1 | hsa_23632 | D00218 |
| 1 | hsa_23632 | D00340 |
| 1 | hsa_23632 | D00518 |
| 1 | hsa_23632 | D00519 |
| 1 | hsa_23632 | D00538 |
| 1 | hsa_23632 | D00652 |
| 1 | hsa_23632 | D00653 |
| 1 | hsa_23632 | D00655 |
| 1 | hsa_23632 | D01196 |
| 1 | hsa_238 | D01441 |
| 1 | hsa_238 | D01977 |
| 1 | hsa_238 | D03218 |
| 1 | hsa_238 | D03350 |
| 1 | hsa_238 | D04024 |
| 1 | hsa_238 | D04025 |
| 1 | hsa_239 | D00097 |
| 1 | hsa_239 | D00118 |
| 1 | hsa_239 | D00126 |
| 1 | hsa_239 | D00132 |
| 1 | hsa_239 | D00141 |
| 1 | hsa_239 | D00448 |
| 1 | hsa_239 | D00510 |
| 1 | hsa_239 | D01332 |
| 1 | hsa_240 | D00097 |
| 1 | hsa_240 | D00118 |
| 1 | hsa_240 | D00126 |
| 1 | hsa_240 | D00132 |
| 1 | hsa_240 | D00141 |
| 1 | hsa_240 | D00377 |
| 1 | hsa_240 | D00414 |
| 1 | hsa_240 | D00448 |
| 1 | hsa_240 | D00510 |
| 1 | hsa_240 | D01332 |
| 1 | hsa_240 | D03882 |
| 1 | hsa_242 | D00097 |
| 1 | hsa_242 | D00118 |
| 1 | hsa_242 | D00126 |
| 1 | hsa_242 | D00132 |
| 1 | hsa_242 | D00141 |
| 1 | hsa_242 | D00448 |
| 1 | hsa_242 | D00510 |
| 1 | hsa_242 | D01332 |
| 1 | hsa_246 | D00097 |
| 1 | hsa_246 | D00118 |
| 1 | hsa_246 | D00132 |
| 1 | hsa_246 | D00141 |
| 1 | hsa_246 | D00448 |
| 1 | hsa_246 | D00510 |
| 1 | hsa_246 | D01332 |
| 1 | hsa_246 | D01364 |
| 1 | hsa_247 | D00097 |
| 1 | hsa_247 | D00118 |
| 1 | hsa_247 | D00132 |
| 1 | hsa_247 | D00141 |
| 1 | hsa_247 | D00448 |
| 1 | hsa_247 | D00510 |
| 1 | hsa_247 | D01332 |
| 1 | hsa_2534 | D01441 |
| 1 | hsa_2548 | D00216 |
| 1 | hsa_2548 | D00625 |
| 1 | hsa_2548 | D01665 |
| 1 | hsa_2548 | D03433 |
| 1 | hsa_25796 | D01223 |
| 1 | hsa_25824 | D00038 |
| 1 | hsa_25824 | D00217 |
| 1 | hsa_25824 | D00577 |
| 1 | hsa_25824 | D03670 |
| 1 | hsa_2582 | D00002 |
| 1 | hsa_2595 | D00216 |
| 1 | hsa_2595 | D00625 |
| 1 | hsa_2595 | D01665 |
| 1 | hsa_2595 | D03433 |
| 1 | hsa_2597 | D00002 |
| 1 | hsa_25 | D01441 |
| 1 | hsa_260293 | D00139 |
| 1 | hsa_260293 | D00225 |
| 1 | hsa_260293 | D00380 |
| 1 | hsa_260293 | D00394 |
| 1 | hsa_260293 | D00410 |
| 1 | hsa_260293 | D00437 |
| 1 | hsa_260293 | D00528 |
| 1 | hsa_260293 | D00542 |
| 1 | hsa_260293 | D00574 |
| 1 | hsa_260293 | D00960 |
| 1 | hsa_260293 | D00964 |
| 1 | hsa_260293 | D01071 |
| 1 | hsa_260293 | D01425 |
| 1 | hsa_260293 | D02451 |
| 1 | hsa_260293 | D03670 |
| 1 | hsa_260293 | D03778 |
| 1 | hsa_260293 | D03781 |
| 1 | hsa_260293 | D03784 |
| 1 | hsa_26279 | D01223 |
| 1 | hsa_2638 | D02315 |
| 1 | hsa_2639 | D00005 |
| 1 | hsa_2673 | D00332 |
| 1 | hsa_2677 | D00148 |
| 1 | hsa_2677 | D02335 |
| 1 | hsa_27032 | D00542 |
| 1 | hsa_27032 | D00543 |
| 1 | hsa_27032 | D00544 |
| 1 | hsa_27032 | D00545 |
| 1 | hsa_27032 | D00546 |
| 1 | hsa_27032 | D00547 |
| 1 | hsa_27034 | D00005 |
| 1 | hsa_27034 | D00039 |
| 1 | hsa_270 | D02769 |
| 1 | hsa_27115 | D00227 |
| 1 | hsa_27115 | D00231 |
| 1 | hsa_27115 | D00371 |
| 1 | hsa_27115 | D00417 |
| 1 | hsa_27115 | D00501 |
| 1 | hsa_27115 | D00528 |
| 1 | hsa_27115 | D00691 |
| 1 | hsa_27115 | D01133 |
| 1 | hsa_27115 | D01198 |
| 1 | hsa_27115 | D01332 |
| 1 | hsa_27115 | D01690 |
| 1 | hsa_27115 | D01704 |
| 1 | hsa_27115 | D01712 |
| 1 | hsa_27115 | D02008 |
| 1 | hsa_27115 | D02017 |
| 1 | hsa_27115 | D02042 |
| 1 | hsa_27115 | D02229 |
| 1 | hsa_27115 | D02655 |
| 1 | hsa_27115 | D02731 |
| 1 | hsa_2746 | D00002 |
| 1 | hsa_2746 | D00007 |
| 1 | hsa_2766 | D03798 |
| 1 | hsa_279 | D00216 |
| 1 | hsa_279 | D00625 |
| 1 | hsa_279 | D01346 |
| 1 | hsa_2806 | D00332 |
| 1 | hsa_28227 | D00107 |
| 1 | hsa_28227 | D00184 |
| 1 | hsa_2822 | D00417 |
| 1 | hsa_2822 | D00501 |
| 1 | hsa_2822 | D00528 |
| 1 | hsa_284541 | D00139 |
| 1 | hsa_284541 | D00225 |
| 1 | hsa_284541 | D00380 |
| 1 | hsa_284541 | D00394 |
| 1 | hsa_284541 | D00410 |
| 1 | hsa_284541 | D00437 |
| 1 | hsa_284541 | D00528 |
| 1 | hsa_284541 | D00542 |
| 1 | hsa_284541 | D00574 |
| 1 | hsa_284541 | D01071 |
| 1 | hsa_284541 | D03670 |
| 1 | hsa_285220 | D01441 |
| 1 | hsa_285220 | D01977 |
| 1 | hsa_285220 | D03218 |
| 1 | hsa_285220 | D03350 |
| 1 | hsa_285220 | D04024 |
| 1 | hsa_285220 | D04025 |
| 1 | hsa_2880 | D00577 |
| 1 | hsa_2882 | D00577 |
| 1 | hsa_28972 | D00043 |
| 1 | hsa_28972 | D00160 |
| 1 | hsa_28 | D00203 |
| 1 | hsa_28 | D00222 |
| 1 | hsa_28 | D00317 |
| 1 | hsa_28 | D00884 |
| 1 | hsa_28 | D00885 |
| 1 | hsa_2936 | D00002 |
| 1 | hsa_2936 | D00005 |
| 1 | hsa_2936 | D00014 |
| 1 | hsa_2950 | D00014 |
| 1 | hsa_29785 | D00139 |
| 1 | hsa_29785 | D00225 |
| 1 | hsa_29785 | D00380 |
| 1 | hsa_29785 | D00394 |
| 1 | hsa_29785 | D00410 |
| 1 | hsa_29785 | D00437 |
| 1 | hsa_29785 | D00528 |
| 1 | hsa_29785 | D00542 |
| 1 | hsa_29785 | D00574 |
| 1 | hsa_29785 | D00960 |
| 1 | hsa_29785 | D00964 |
| 1 | hsa_29785 | D01071 |
| 1 | hsa_29785 | D01425 |
| 1 | hsa_29785 | D02451 |
| 1 | hsa_29785 | D03670 |
| 1 | hsa_29785 | D03778 |
| 1 | hsa_29785 | D03781 |
| 1 | hsa_29785 | D03784 |
| 1 | hsa_29920 | D00035 |
| 1 | hsa_29941 | D01441 |
| 1 | hsa_29968 | D00332 |
| 1 | hsa_3001 | D00043 |
| 1 | hsa_3001 | D00160 |
| 1 | hsa_3002 | D00043 |
| 1 | hsa_3002 | D00160 |
| 1 | hsa_3028 | D00002 |
| 1 | hsa_3033 | D00002 |
| 1 | hsa_3034 | D00032 |
| 1 | hsa_3035 | D00032 |
| 1 | hsa_3055 | D01441 |
| 1 | hsa_3067 | D00032 |
| 1 | hsa_306 | D00417 |
| 1 | hsa_306 | D00501 |
| 1 | hsa_306 | D00528 |
| 1 | hsa_30814 | D01223 |
| 1 | hsa_30833 | D00501 |
| 1 | hsa_30 | D00222 |
| 1 | hsa_30 | D00317 |
| 1 | hsa_30 | D00333 |
| 1 | hsa_30 | D00342 |
| 1 | hsa_30 | D00391 |
| 1 | hsa_30 | D00398 |
| 1 | hsa_30 | D05407 |
| 1 | hsa_313 | D01223 |
| 1 | hsa_3141 | D00029 |
| 1 | hsa_3156 | D00359 |
| 1 | hsa_3156 | D00434 |
| 1 | hsa_3156 | D00887 |
| 1 | hsa_3156 | D00889 |
| 1 | hsa_3156 | D00892 |
| 1 | hsa_3156 | D00893 |
| 1 | hsa_3156 | D01862 |
| 1 | hsa_3156 | D01915 |
| 1 | hsa_3156 | D02258 |
| 1 | hsa_3156 | D03601 |
| 1 | hsa_3156 | D03643 |
| 1 | hsa_3156 | D03816 |
| 1 | hsa_31 | D00007 |
| 1 | hsa_31 | D00027 |
| 1 | hsa_31 | D00029 |
| 1 | hsa_326625 | D01027 |
| 1 | hsa_327 | D00043 |
| 1 | hsa_3283 | D01180 |
| 1 | hsa_3290 | D00410 |
| 1 | hsa_3291 | D00410 |
| 1 | hsa_3292 | D00002 |
| 1 | hsa_3295 | D00002 |
| 1 | hsa_32 | D00029 |
| 1 | hsa_3376 | D00065 |
| 1 | hsa_339221 | D00417 |
| 1 | hsa_339221 | D00501 |
| 1 | hsa_339221 | D00528 |
| 1 | hsa_3416 | D00052 |
| 1 | hsa_3480 | D01441 |
| 1 | hsa_3480 | D01977 |
| 1 | hsa_3480 | D03218 |
| 1 | hsa_3480 | D03350 |
| 1 | hsa_3480 | D04024 |
| 1 | hsa_3480 | D04025 |
| 1 | hsa_349565 | D00002 |
| 1 | hsa_34 | D00005 |
| 1 | hsa_353 | D02769 |
| 1 | hsa_354 | D00043 |
| 1 | hsa_354 | D00160 |
| 1 | hsa_3551 | D00097 |
| 1 | hsa_3551 | D00120 |
| 1 | hsa_3551 | D00448 |
| 1 | hsa_35 | D00005 |
| 1 | hsa_3612 | D00501 |
| 1 | hsa_3614 | D00423 |
| 1 | hsa_3614 | D00752 |
| 1 | hsa_3615 | D00002 |
| 1 | hsa_3615 | D00752 |
| 1 | hsa_3643 | D01441 |
| 1 | hsa_3643 | D01977 |
| 1 | hsa_3643 | D03218 |
| 1 | hsa_3643 | D03350 |
| 1 | hsa_3643 | D04024 |
| 1 | hsa_3643 | D04025 |
| 1 | hsa_3643 | D04966 |
| 1 | hsa_3645 | D01441 |
| 1 | hsa_3645 | D01977 |
| 1 | hsa_3645 | D03218 |
| 1 | hsa_3645 | D03350 |
| 1 | hsa_3645 | D04024 |
| 1 | hsa_3645 | D04025 |
| 1 | hsa_3645 | D04966 |
| 1 | hsa_36 | D00065 |
| 1 | hsa_3702 | D01441 |
| 1 | hsa_3712 | D00005 |
| 1 | hsa_3716 | D01441 |
| 1 | hsa_3717 | D01441 |
| 1 | hsa_3718 | D01441 |
| 1 | hsa_3735 | D02304 |
| 1 | hsa_377677 | D00218 |
| 1 | hsa_377677 | D00340 |
| 1 | hsa_377677 | D00518 |
| 1 | hsa_377677 | D00519 |
| 1 | hsa_377677 | D00538 |
| 1 | hsa_377677 | D00652 |
| 1 | hsa_377677 | D00653 |
| 1 | hsa_377677 | D00655 |
| 1 | hsa_377677 | D01196 |
| 1 | hsa_3791 | D01441 |
| 1 | hsa_3791 | D01977 |
| 1 | hsa_3791 | D03218 |
| 1 | hsa_3791 | D03350 |
| 1 | hsa_3791 | D04024 |
| 1 | hsa_3791 | D04025 |
| 1 | hsa_3815 | D01441 |
| 1 | hsa_3815 | D01977 |
| 1 | hsa_3815 | D03218 |
| 1 | hsa_3815 | D03350 |
| 1 | hsa_3815 | D04024 |
| 1 | hsa_3815 | D04025 |
| 1 | hsa_3816 | D00043 |
| 1 | hsa_3816 | D00160 |
| 1 | hsa_3817 | D00043 |
| 1 | hsa_3817 | D00160 |
| 1 | hsa_3818 | D00043 |
| 1 | hsa_3818 | D00160 |
| 1 | hsa_38 | D00065 |
| 1 | hsa_38 | D00448 |
| 1 | hsa_3906 | D00126 |
| 1 | hsa_3906 | D00132 |
| 1 | hsa_3906 | D00293 |
| 1 | hsa_3906 | D00510 |
| 1 | hsa_390956 | D00107 |
| 1 | hsa_3932 | D01441 |
| 1 | hsa_3939 | D00002 |
| 1 | hsa_3945 | D00002 |
| 1 | hsa_3988 | D04028 |
| 1 | hsa_3990 | D01223 |
| 1 | hsa_3990 | D04028 |
| 1 | hsa_3991 | D01223 |
| 1 | hsa_4017 | D00270 |
| 1 | hsa_4023 | D00279 |
| 1 | hsa_4023 | D01223 |
| 1 | hsa_4025 | D00097 |
| 1 | hsa_4025 | D00217 |
| 1 | hsa_4025 | D00562 |
| 1 | hsa_4025 | D00577 |
| 1 | hsa_4025 | D03670 |
| 1 | hsa_4048 | D00097 |
| 1 | hsa_4048 | D00118 |
| 1 | hsa_4048 | D00126 |
| 1 | hsa_4048 | D00132 |
| 1 | hsa_4048 | D00141 |
| 1 | hsa_4048 | D00448 |
| 1 | hsa_4048 | D00510 |
| 1 | hsa_4048 | D01332 |
| 1 | hsa_4051 | D00139 |
| 1 | hsa_4051 | D00225 |
| 1 | hsa_4051 | D00380 |
| 1 | hsa_4051 | D00394 |
| 1 | hsa_4051 | D00410 |
| 1 | hsa_4051 | D00437 |
| 1 | hsa_4051 | D00528 |
| 1 | hsa_4051 | D00542 |
| 1 | hsa_4051 | D00574 |
| 1 | hsa_4051 | D01071 |
| 1 | hsa_4051 | D03670 |
| 1 | hsa_4058 | D01441 |
| 1 | hsa_4058 | D01977 |
| 1 | hsa_4058 | D03218 |
| 1 | hsa_4058 | D03350 |
| 1 | hsa_4058 | D04024 |
| 1 | hsa_4058 | D04025 |
| 1 | hsa_4067 | D01441 |
| 1 | hsa_4128 | D00270 |
| 1 | hsa_4128 | D00505 |
| 1 | hsa_4128 | D00785 |
| 1 | hsa_4128 | D00826 |
| 1 | hsa_4128 | D00947 |
| 1 | hsa_4128 | D01097 |
| 1 | hsa_4128 | D01888 |
| 1 | hsa_4128 | D02559 |
| 1 | hsa_4128 | D02560 |
| 1 | hsa_4128 | D02561 |
| 1 | hsa_4128 | D02562 |
| 1 | hsa_4128 | D02563 |
| 1 | hsa_4128 | D02564 |
| 1 | hsa_4128 | D02579 |
| 1 | hsa_4128 | D02580 |
| 1 | hsa_4128 | D02581 |
| 1 | hsa_4128 | D03731 |
| 1 | hsa_4128 | D03733 |
| 1 | hsa_4129 | D00005 |
| 1 | hsa_4129 | D00270 |
| 1 | hsa_4129 | D00505 |
| 1 | hsa_4129 | D00785 |
| 1 | hsa_4129 | D00826 |
| 1 | hsa_4129 | D01097 |
| 1 | hsa_4129 | D01888 |
| 1 | hsa_4129 | D02559 |
| 1 | hsa_4129 | D02560 |
| 1 | hsa_4129 | D02561 |
| 1 | hsa_4129 | D02562 |
| 1 | hsa_4129 | D02563 |
| 1 | hsa_4129 | D02564 |
| 1 | hsa_4129 | D02579 |
| 1 | hsa_4129 | D02580 |
| 1 | hsa_4129 | D02581 |
| 1 | hsa_4129 | D03731 |
| 1 | hsa_4129 | D03733 |
| 1 | hsa_4129 | D05458 |
| 1 | hsa_4143 | D04983 |
| 1 | hsa_4145 | D01441 |
| 1 | hsa_4190 | D00002 |
| 1 | hsa_4191 | D00037 |
| 1 | hsa_4200 | D00002 |
| 1 | hsa_4200 | D02308 |
| 1 | hsa_4233 | D01441 |
| 1 | hsa_4233 | D01977 |
| 1 | hsa_4233 | D03218 |
| 1 | hsa_4233 | D03350 |
| 1 | hsa_4233 | D04024 |
| 1 | hsa_4233 | D04025 |
| 1 | hsa_4282 | D00037 |
| 1 | hsa_4311 | D00052 |
| 1 | hsa_4329 | D00039 |
| 1 | hsa_4353 | D00097 |
| 1 | hsa_4353 | D00217 |
| 1 | hsa_4353 | D00401 |
| 1 | hsa_4353 | D00562 |
| 1 | hsa_4353 | D00577 |
| 1 | hsa_43 | D00043 |
| 1 | hsa_43 | D00196 |
| 1 | hsa_43 | D00487 |
| 1 | hsa_43 | D00667 |
| 1 | hsa_43 | D00670 |
| 1 | hsa_43 | D00805 |
| 1 | hsa_43 | D00994 |
| 1 | hsa_43 | D00995 |
| 1 | hsa_43 | D00998 |
| 1 | hsa_43 | D01001 |
| 1 | hsa_43 | D01223 |
| 1 | hsa_43 | D01228 |
| 1 | hsa_43 | D02068 |
| 1 | hsa_43 | D02173 |
| 1 | hsa_43 | D02193 |
| 1 | hsa_43 | D02418 |
| 1 | hsa_43 | D02558 |
| 1 | hsa_43 | D02729 |
| 1 | hsa_43 | D03751 |
| 1 | hsa_43 | D03822 |
| 1 | hsa_43 | D03823 |
| 1 | hsa_43 | D03826 |
| 1 | hsa_43 | D04292 |
| 1 | hsa_444 | D00131 |
| 1 | hsa_4482 | D04983 |
| 1 | hsa_4486 | D01441 |
| 1 | hsa_4486 | D01977 |
| 1 | hsa_4486 | D03218 |
| 1 | hsa_4486 | D03350 |
| 1 | hsa_4486 | D04024 |
| 1 | hsa_4486 | D04025 |
| 1 | hsa_4548 | D01027 |
| 1 | hsa_4548 | D04983 |
| 1 | hsa_4552 | D01027 |
| 1 | hsa_4552 | D04983 |
| 1 | hsa_4593 | D00726 |
| 1 | hsa_4593 | D01441 |
| 1 | hsa_4593 | D01977 |
| 1 | hsa_4593 | D03218 |
| 1 | hsa_4593 | D03350 |
| 1 | hsa_4593 | D04024 |
| 1 | hsa_4593 | D04025 |
| 1 | hsa_4594 | D01027 |
| 1 | hsa_4758 | D00900 |
| 1 | hsa_4758 | D00902 |
| 1 | hsa_4758 | D03829 |
| 1 | hsa_4759 | D00900 |
| 1 | hsa_4759 | D00902 |
| 1 | hsa_4759 | D03829 |
| 1 | hsa_476 | D00298 |
| 1 | hsa_476 | D00654 |
| 1 | hsa_476 | D00656 |
| 1 | hsa_476 | D00658 |
| 1 | hsa_476 | D01240 |
| 1 | hsa_4835 | D00005 |
| 1 | hsa_4835 | D03798 |
| 1 | hsa_4837 | D00049 |
| 1 | hsa_4860 | D00054 |
| 1 | hsa_4860 | D01370 |
| 1 | hsa_4881 | D00515 |
| 1 | hsa_4881 | D00516 |
| 1 | hsa_4881 | D00630 |
| 1 | hsa_4907 | D00501 |
| 1 | hsa_4914 | D01441 |
| 1 | hsa_4914 | D01977 |
| 1 | hsa_4914 | D03218 |
| 1 | hsa_4914 | D03350 |
| 1 | hsa_4914 | D04024 |
| 1 | hsa_4914 | D04025 |
| 1 | hsa_4915 | D01441 |
| 1 | hsa_4915 | D01977 |
| 1 | hsa_4915 | D03218 |
| 1 | hsa_4915 | D03350 |
| 1 | hsa_4915 | D04024 |
| 1 | hsa_4915 | D04025 |
| 1 | hsa_4916 | D01441 |
| 1 | hsa_4916 | D01977 |
| 1 | hsa_4916 | D03218 |
| 1 | hsa_4916 | D03350 |
| 1 | hsa_4916 | D04024 |
| 1 | hsa_4916 | D04025 |
| 1 | hsa_4919 | D01441 |
| 1 | hsa_4919 | D01977 |
| 1 | hsa_4919 | D03218 |
| 1 | hsa_4919 | D03350 |
| 1 | hsa_4919 | D04024 |
| 1 | hsa_4919 | D04025 |
| 1 | hsa_4920 | D01441 |
| 1 | hsa_4920 | D01977 |
| 1 | hsa_4920 | D03218 |
| 1 | hsa_4920 | D03350 |
| 1 | hsa_4920 | D04024 |
| 1 | hsa_4920 | D04025 |
| 1 | hsa_4921 | D01441 |
| 1 | hsa_4921 | D01977 |
| 1 | hsa_4921 | D03218 |
| 1 | hsa_4921 | D03350 |
| 1 | hsa_4921 | D04024 |
| 1 | hsa_4921 | D04025 |
| 1 | hsa_4942 | D00332 |
| 1 | hsa_4942 | D00475 |
| 1 | hsa_4953 | D00829 |
| 1 | hsa_495 | D00455 |
| 1 | hsa_495 | D01984 |
| 1 | hsa_49 | D00043 |
| 1 | hsa_49 | D00160 |
| 1 | hsa_501 | D00131 |
| 1 | hsa_5033 | D00018 |
| 1 | hsa_5045 | D00043 |
| 1 | hsa_5045 | D00160 |
| 1 | hsa_50484 | D02566 |
| 1 | hsa_50484 | D03670 |
| 1 | hsa_50487 | D01223 |
| 1 | hsa_5049 | D01223 |
| 1 | hsa_5050 | D01223 |
| 1 | hsa_5051 | D01223 |
| 1 | hsa_5052 | D00217 |
| 1 | hsa_5052 | D00577 |
| 1 | hsa_5052 | D03670 |
| 1 | hsa_5053 | D00021 |
| 1 | hsa_5091 | D00029 |
| 1 | hsa_50940 | D00227 |
| 1 | hsa_50940 | D00231 |
| 1 | hsa_50940 | D00371 |
| 1 | hsa_50940 | D00417 |
| 1 | hsa_50940 | D01133 |
| 1 | hsa_50940 | D01198 |
| 1 | hsa_50940 | D01690 |
| 1 | hsa_50940 | D01704 |
| 1 | hsa_50940 | D01712 |
| 1 | hsa_50940 | D02008 |
| 1 | hsa_50940 | D02017 |
| 1 | hsa_50940 | D02042 |
| 1 | hsa_50940 | D02229 |
| 1 | hsa_50940 | D02655 |
| 1 | hsa_50940 | D02731 |
| 1 | hsa_5095 | D00029 |
| 1 | hsa_5095 | D00039 |
| 1 | hsa_5095 | D00041 |
| 1 | hsa_5095 | D00065 |
| 1 | hsa_5096 | D00029 |
| 1 | hsa_5096 | D00039 |
| 1 | hsa_5096 | D00041 |
| 1 | hsa_5096 | D00065 |
| 1 | hsa_50 | D00037 |
| 1 | hsa_51004 | D00139 |
| 1 | hsa_51004 | D00225 |
| 1 | hsa_51004 | D00380 |
| 1 | hsa_51004 | D00394 |
| 1 | hsa_51004 | D00410 |
| 1 | hsa_51004 | D00437 |
| 1 | hsa_51004 | D00528 |
| 1 | hsa_51004 | D00542 |
| 1 | hsa_51004 | D00574 |
| 1 | hsa_51004 | D01071 |
| 1 | hsa_51095 | D00417 |
| 1 | hsa_51095 | D00501 |
| 1 | hsa_51095 | D00528 |
| 1 | hsa_51166 | D00332 |
| 1 | hsa_51172 | D00417 |
| 1 | hsa_51172 | D00501 |
| 1 | hsa_51172 | D00528 |
| 1 | hsa_51181 | D00136 |
| 1 | hsa_51181 | D00219 |
| 1 | hsa_51181 | D00410 |
| 1 | hsa_51205 | D00103 |
| 1 | hsa_51207 | D00107 |
| 1 | hsa_51207 | D00184 |
| 1 | hsa_5122 | D00043 |
| 1 | hsa_5122 | D00160 |
| 1 | hsa_51251 | D00501 |
| 1 | hsa_5126 | D00043 |
| 1 | hsa_5126 | D00160 |
| 1 | hsa_51292 | D03798 |
| 1 | hsa_51302 | D00139 |
| 1 | hsa_51302 | D00225 |
| 1 | hsa_51302 | D00380 |
| 1 | hsa_51302 | D00394 |
| 1 | hsa_51302 | D00410 |
| 1 | hsa_51302 | D00437 |
| 1 | hsa_51302 | D00528 |
| 1 | hsa_51302 | D00542 |
| 1 | hsa_51302 | D00574 |
| 1 | hsa_51302 | D01071 |
| 1 | hsa_51365 | D01223 |
| 1 | hsa_5136 | D00227 |
| 1 | hsa_5136 | D00231 |
| 1 | hsa_5136 | D00371 |
| 1 | hsa_5136 | D00417 |
| 1 | hsa_5136 | D00501 |
| 1 | hsa_5136 | D00528 |
| 1 | hsa_5136 | D00691 |
| 1 | hsa_5136 | D01133 |
| 1 | hsa_5136 | D01198 |
| 1 | hsa_5136 | D01690 |
| 1 | hsa_5136 | D01704 |
| 1 | hsa_5136 | D01712 |
| 1 | hsa_5136 | D02008 |
| 1 | hsa_5136 | D02017 |
| 1 | hsa_5136 | D02042 |
| 1 | hsa_5136 | D02229 |
| 1 | hsa_5136 | D02655 |
| 1 | hsa_5136 | D02731 |
| 1 | hsa_5137 | D00227 |
| 1 | hsa_5137 | D00231 |
| 1 | hsa_5137 | D00371 |
| 1 | hsa_5137 | D00417 |
| 1 | hsa_5137 | D00501 |
| 1 | hsa_5137 | D00528 |
| 1 | hsa_5137 | D00691 |
| 1 | hsa_5137 | D01133 |
| 1 | hsa_5137 | D01198 |
| 1 | hsa_5137 | D01690 |
| 1 | hsa_5137 | D01704 |
| 1 | hsa_5137 | D01712 |
| 1 | hsa_5137 | D02008 |
| 1 | hsa_5137 | D02017 |
| 1 | hsa_5137 | D02042 |
| 1 | hsa_5137 | D02229 |
| 1 | hsa_5137 | D02655 |
| 1 | hsa_5137 | D02731 |
| 1 | hsa_5138 | D00227 |
| 1 | hsa_5138 | D00231 |
| 1 | hsa_5138 | D00371 |
| 1 | hsa_5138 | D00417 |
| 1 | hsa_5138 | D00501 |
| 1 | hsa_5138 | D00528 |
| 1 | hsa_5138 | D00691 |
| 1 | hsa_5138 | D01133 |
| 1 | hsa_5138 | D01198 |
| 1 | hsa_5138 | D01690 |
| 1 | hsa_5138 | D01704 |
| 1 | hsa_5138 | D01712 |
| 1 | hsa_5138 | D02008 |
| 1 | hsa_5138 | D02017 |
| 1 | hsa_5138 | D02042 |
| 1 | hsa_5138 | D02229 |
| 1 | hsa_5138 | D02655 |
| 1 | hsa_5138 | D02731 |
| 1 | hsa_513 | D00542 |
| 1 | hsa_513 | D00543 |
| 1 | hsa_513 | D00544 |
| 1 | hsa_513 | D00545 |
| 1 | hsa_513 | D00546 |
| 1 | hsa_513 | D00547 |
| 1 | hsa_5140 | D00227 |
| 1 | hsa_5140 | D00231 |
| 1 | hsa_5140 | D00371 |
| 1 | hsa_5140 | D00417 |
| 1 | hsa_5140 | D00501 |
| 1 | hsa_5140 | D00528 |
| 1 | hsa_5140 | D00691 |
| 1 | hsa_5140 | D01133 |
| 1 | hsa_5140 | D01198 |
| 1 | hsa_5140 | D01690 |
| 1 | hsa_5140 | D01704 |
| 1 | hsa_5140 | D01712 |
| 1 | hsa_5140 | D02008 |
| 1 | hsa_5140 | D02017 |
| 1 | hsa_5140 | D02042 |
| 1 | hsa_5140 | D02229 |
| 1 | hsa_5140 | D02655 |
| 1 | hsa_5140 | D02731 |
| 1 | hsa_5141 | D00227 |
| 1 | hsa_5141 | D00231 |
| 1 | hsa_5141 | D00371 |
| 1 | hsa_5141 | D00417 |
| 1 | hsa_5141 | D00501 |
| 1 | hsa_5141 | D00528 |
| 1 | hsa_5141 | D00691 |
| 1 | hsa_5141 | D01133 |
| 1 | hsa_5141 | D01198 |
| 1 | hsa_5141 | D01690 |
| 1 | hsa_5141 | D01704 |
| 1 | hsa_5141 | D01712 |
| 1 | hsa_5141 | D02008 |
| 1 | hsa_5141 | D02017 |
| 1 | hsa_5141 | D02042 |
| 1 | hsa_5141 | D02229 |
| 1 | hsa_5141 | D02655 |
| 1 | hsa_5141 | D02731 |
| 1 | hsa_5142 | D00227 |
| 1 | hsa_5142 | D00231 |
| 1 | hsa_5142 | D00371 |
| 1 | hsa_5142 | D00417 |
| 1 | hsa_5142 | D00501 |
| 1 | hsa_5142 | D00528 |
| 1 | hsa_5142 | D00691 |
| 1 | hsa_5142 | D01133 |
| 1 | hsa_5142 | D01198 |
| 1 | hsa_5142 | D01690 |
| 1 | hsa_5142 | D01704 |
| 1 | hsa_5142 | D01712 |
| 1 | hsa_5142 | D02008 |
| 1 | hsa_5142 | D02017 |
| 1 | hsa_5142 | D02042 |
| 1 | hsa_5142 | D02229 |
| 1 | hsa_5142 | D02655 |
| 1 | hsa_5142 | D02731 |
| 1 | hsa_5143 | D00227 |
| 1 | hsa_5143 | D00231 |
| 1 | hsa_5143 | D00371 |
| 1 | hsa_5143 | D00417 |
| 1 | hsa_5143 | D00501 |
| 1 | hsa_5143 | D00528 |
| 1 | hsa_5143 | D00691 |
| 1 | hsa_5143 | D01133 |
| 1 | hsa_5143 | D01198 |
| 1 | hsa_5143 | D01690 |
| 1 | hsa_5143 | D01704 |
| 1 | hsa_5143 | D01712 |
| 1 | hsa_5143 | D02008 |
| 1 | hsa_5143 | D02017 |
| 1 | hsa_5143 | D02042 |
| 1 | hsa_5143 | D02229 |
| 1 | hsa_5143 | D02655 |
| 1 | hsa_5143 | D02731 |
| 1 | hsa_5144 | D00227 |
| 1 | hsa_5144 | D00231 |
| 1 | hsa_5144 | D00371 |
| 1 | hsa_5144 | D00417 |
| 1 | hsa_5144 | D00501 |
| 1 | hsa_5144 | D00528 |
| 1 | hsa_5144 | D00691 |
| 1 | hsa_5144 | D01133 |
| 1 | hsa_5144 | D01198 |
| 1 | hsa_5144 | D01690 |
| 1 | hsa_5144 | D01704 |
| 1 | hsa_5144 | D01712 |
| 1 | hsa_5144 | D02008 |
| 1 | hsa_5144 | D02017 |
| 1 | hsa_5144 | D02042 |
| 1 | hsa_5144 | D02229 |
| 1 | hsa_5144 | D02655 |
| 1 | hsa_5144 | D02731 |
| 1 | hsa_5145 | D00417 |
| 1 | hsa_5145 | D00501 |
| 1 | hsa_5145 | D00528 |
| 1 | hsa_5146 | D00417 |
| 1 | hsa_5146 | D00501 |
| 1 | hsa_5146 | D00528 |
| 1 | hsa_5147 | D00227 |
| 1 | hsa_5147 | D00231 |
| 1 | hsa_5147 | D00371 |
| 1 | hsa_5147 | D00417 |
| 1 | hsa_5147 | D00501 |
| 1 | hsa_5147 | D00528 |
| 1 | hsa_5147 | D00691 |
| 1 | hsa_5147 | D01133 |
| 1 | hsa_5147 | D01198 |
| 1 | hsa_5147 | D01690 |
| 1 | hsa_5147 | D01704 |
| 1 | hsa_5147 | D01712 |
| 1 | hsa_5147 | D02008 |
| 1 | hsa_5147 | D02017 |
| 1 | hsa_5147 | D02042 |
| 1 | hsa_5147 | D02229 |
| 1 | hsa_5147 | D02655 |
| 1 | hsa_5147 | D02731 |
| 1 | hsa_5148 | D00227 |
| 1 | hsa_5148 | D00231 |
| 1 | hsa_5148 | D00371 |
| 1 | hsa_5148 | D00417 |
| 1 | hsa_5148 | D00501 |
| 1 | hsa_5148 | D00528 |
| 1 | hsa_5148 | D00691 |
| 1 | hsa_5148 | D01133 |
| 1 | hsa_5148 | D01198 |
| 1 | hsa_5148 | D01690 |
| 1 | hsa_5148 | D01704 |
| 1 | hsa_5148 | D01712 |
| 1 | hsa_5148 | D02008 |
| 1 | hsa_5148 | D02017 |
| 1 | hsa_5148 | D02042 |
| 1 | hsa_5148 | D02229 |
| 1 | hsa_5148 | D02655 |
| 1 | hsa_5148 | D02731 |
| 1 | hsa_5149 | D00227 |
| 1 | hsa_5149 | D00231 |
| 1 | hsa_5149 | D00371 |
| 1 | hsa_5149 | D00417 |
| 1 | hsa_5149 | D00501 |
| 1 | hsa_5149 | D00528 |
| 1 | hsa_5149 | D00691 |
| 1 | hsa_5149 | D01133 |
| 1 | hsa_5149 | D01198 |
| 1 | hsa_5149 | D01690 |
| 1 | hsa_5149 | D01704 |
| 1 | hsa_5149 | D01712 |
| 1 | hsa_5149 | D02008 |
| 1 | hsa_5149 | D02017 |
| 1 | hsa_5149 | D02042 |
| 1 | hsa_5149 | D02229 |
| 1 | hsa_5149 | D02655 |
| 1 | hsa_5149 | D02731 |
| 1 | hsa_5150 | D00227 |
| 1 | hsa_5150 | D00231 |
| 1 | hsa_5150 | D00371 |
| 1 | hsa_5150 | D00417 |
| 1 | hsa_5150 | D01133 |
| 1 | hsa_5150 | D01198 |
| 1 | hsa_5150 | D01332 |
| 1 | hsa_5150 | D01690 |
| 1 | hsa_5150 | D01704 |
| 1 | hsa_5150 | D01712 |
| 1 | hsa_5150 | D02008 |
| 1 | hsa_5150 | D02017 |
| 1 | hsa_5150 | D02042 |
| 1 | hsa_5150 | D02229 |
| 1 | hsa_5150 | D02655 |
| 1 | hsa_5150 | D02731 |
| 1 | hsa_5151 | D00227 |
| 1 | hsa_5151 | D00231 |
| 1 | hsa_5151 | D00371 |
| 1 | hsa_5151 | D00417 |
| 1 | hsa_5151 | D00501 |
| 1 | hsa_5151 | D00528 |
| 1 | hsa_5151 | D00691 |
| 1 | hsa_5151 | D01133 |
| 1 | hsa_5151 | D01198 |
| 1 | hsa_5151 | D01690 |
| 1 | hsa_5151 | D01704 |
| 1 | hsa_5151 | D01712 |
| 1 | hsa_5151 | D02008 |
| 1 | hsa_5151 | D02017 |
| 1 | hsa_5151 | D02042 |
| 1 | hsa_5151 | D02229 |
| 1 | hsa_5151 | D02655 |
| 1 | hsa_5151 | D02731 |
| 1 | hsa_5152 | D00227 |
| 1 | hsa_5152 | D00231 |
| 1 | hsa_5152 | D00371 |
| 1 | hsa_5152 | D00417 |
| 1 | hsa_5152 | D00501 |
| 1 | hsa_5152 | D00528 |
| 1 | hsa_5152 | D01133 |
| 1 | hsa_5152 | D01198 |
| 1 | hsa_5152 | D01690 |
| 1 | hsa_5152 | D01704 |
| 1 | hsa_5152 | D01712 |
| 1 | hsa_5152 | D02008 |
| 1 | hsa_5152 | D02017 |
| 1 | hsa_5152 | D02042 |
| 1 | hsa_5152 | D02229 |
| 1 | hsa_5152 | D02655 |
| 1 | hsa_5152 | D02731 |
| 1 | hsa_5156 | D01441 |
| 1 | hsa_5156 | D01977 |
| 1 | hsa_5156 | D03218 |
| 1 | hsa_5156 | D03350 |
| 1 | hsa_5156 | D04024 |
| 1 | hsa_5156 | D04025 |
| 1 | hsa_5158 | D00417 |
| 1 | hsa_5158 | D00501 |
| 1 | hsa_5158 | D00528 |
| 1 | hsa_5159 | D01441 |
| 1 | hsa_5159 | D01977 |
| 1 | hsa_5159 | D03218 |
| 1 | hsa_5159 | D03350 |
| 1 | hsa_5159 | D04024 |
| 1 | hsa_5159 | D04025 |
| 1 | hsa_51645 | D00107 |
| 1 | hsa_5167 | D00043 |
| 1 | hsa_5167 | D00417 |
| 1 | hsa_5167 | D00423 |
| 1 | hsa_5167 | D00501 |
| 1 | hsa_5167 | D00528 |
| 1 | hsa_5168 | D00417 |
| 1 | hsa_5168 | D00501 |
| 1 | hsa_5168 | D00528 |
| 1 | hsa_5169 | D00417 |
| 1 | hsa_5169 | D00501 |
| 1 | hsa_5169 | D00528 |
| 1 | hsa_51727 | D02368 |
| 1 | hsa_51 | D00185 |
| 1 | hsa_51 | D00293 |
| 1 | hsa_51 | D00332 |
| 1 | hsa_51 | D00530 |
| 1 | hsa_51 | D00549 |
| 1 | hsa_5294 | D03670 |
| 1 | hsa_52 | D00103 |
| 1 | hsa_5319 | D01223 |
| 1 | hsa_5320 | D01223 |
| 1 | hsa_5321 | D00324 |
| 1 | hsa_5321 | D00325 |
| 1 | hsa_5321 | D00328 |
| 1 | hsa_5321 | D01223 |
| 1 | hsa_5321 | D01367 |
| 1 | hsa_5321 | D01825 |
| 1 | hsa_5321 | D02289 |
| 1 | hsa_5322 | D01223 |
| 1 | hsa_5327 | D00043 |
| 1 | hsa_5327 | D00160 |
| 1 | hsa_5328 | D00043 |
| 1 | hsa_5328 | D00160 |
| 1 | hsa_5328 | D01136 |
| 1 | hsa_5330 | D00417 |
| 1 | hsa_5330 | D00501 |
| 1 | hsa_5330 | D00528 |
| 1 | hsa_5331 | D00417 |
| 1 | hsa_5331 | D00501 |
| 1 | hsa_5331 | D00528 |
| 1 | hsa_5332 | D00417 |
| 1 | hsa_5332 | D00501 |
| 1 | hsa_5332 | D00528 |
| 1 | hsa_5333 | D00417 |
| 1 | hsa_5333 | D00501 |
| 1 | hsa_5333 | D00528 |
| 1 | hsa_5335 | D00417 |
| 1 | hsa_5335 | D00501 |
| 1 | hsa_5335 | D00528 |
| 1 | hsa_5336 | D00417 |
| 1 | hsa_5336 | D00501 |
| 1 | hsa_5336 | D00528 |
| 1 | hsa_5337 | D00417 |
| 1 | hsa_5337 | D00501 |
| 1 | hsa_5337 | D00528 |
| 1 | hsa_5338 | D00417 |
| 1 | hsa_5338 | D00501 |
| 1 | hsa_5338 | D00528 |
| 1 | hsa_5340 | D00043 |
| 1 | hsa_5340 | D00160 |
| 1 | hsa_5340 | D01136 |
| 1 | hsa_5351 | D00018 |
| 1 | hsa_53938 | D00107 |
| 1 | hsa_53 | D00103 |
| 1 | hsa_5406 | D01223 |
| 1 | hsa_5406 | D01346 |
| 1 | hsa_5406 | D04028 |
| 1 | hsa_5407 | D01223 |
| 1 | hsa_5407 | D04028 |
| 1 | hsa_5408 | D01223 |
| 1 | hsa_5408 | D04028 |
| 1 | hsa_5422 | D01907 |
| 1 | hsa_5423 | D00168 |
| 1 | hsa_5444 | D00043 |
| 1 | hsa_5445 | D00043 |
| 1 | hsa_5447 | D00208 |
| 1 | hsa_5447 | D00965 |
| 1 | hsa_54490 | D01276 |
| 1 | hsa_54575 | D01276 |
| 1 | hsa_54576 | D01276 |
| 1 | hsa_54577 | D01276 |
| 1 | hsa_54578 | D01276 |
| 1 | hsa_54579 | D01276 |
| 1 | hsa_54600 | D01276 |
| 1 | hsa_54657 | D01276 |
| 1 | hsa_54658 | D01276 |
| 1 | hsa_54659 | D01276 |
| 1 | hsa_54677 | D02176 |
| 1 | hsa_5470 | D00107 |
| 1 | hsa_5470 | D00184 |
| 1 | hsa_5475 | D00107 |
| 1 | hsa_5475 | D00184 |
| 1 | hsa_5476 | D00043 |
| 1 | hsa_5478 | D00107 |
| 1 | hsa_5479 | D00107 |
| 1 | hsa_5481 | D00107 |
| 1 | hsa_54878 | D00043 |
| 1 | hsa_5494 | D00107 |
| 1 | hsa_5494 | D00184 |
| 1 | hsa_5495 | D00107 |
| 1 | hsa_5495 | D00184 |
| 1 | hsa_5496 | D00107 |
| 1 | hsa_5496 | D00184 |
| 1 | hsa_5499 | D00107 |
| 1 | hsa_5499 | D00184 |
| 1 | hsa_54 | D00103 |
| 1 | hsa_5500 | D00107 |
| 1 | hsa_5500 | D00184 |
| 1 | hsa_5501 | D00107 |
| 1 | hsa_5501 | D00184 |
| 1 | hsa_5515 | D00107 |
| 1 | hsa_5515 | D00184 |
| 1 | hsa_5516 | D00107 |
| 1 | hsa_5516 | D00184 |
| 1 | hsa_5523 | D00107 |
| 1 | hsa_5523 | D00184 |
| 1 | hsa_5530 | D00107 |
| 1 | hsa_5530 | D00184 |
| 1 | hsa_55312 | D00050 |
| 1 | hsa_5531 | D00107 |
| 1 | hsa_5531 | D00184 |
| 1 | hsa_5532 | D00107 |
| 1 | hsa_5532 | D00184 |
| 1 | hsa_5533 | D00107 |
| 1 | hsa_5533 | D00184 |
| 1 | hsa_55359 | D01441 |
| 1 | hsa_5536 | D00107 |
| 1 | hsa_5536 | D00184 |
| 1 | hsa_5537 | D00107 |
| 1 | hsa_5537 | D00184 |
| 1 | hsa_5538 | D05341 |
| 1 | hsa_5547 | D00043 |
| 1 | hsa_5550 | D00043 |
| 1 | hsa_5550 | D00160 |
| 1 | hsa_55512 | D00417 |
| 1 | hsa_55512 | D00501 |
| 1 | hsa_55512 | D00528 |
| 1 | hsa_5562 | D02769 |
| 1 | hsa_55775 | D00417 |
| 1 | hsa_55775 | D00501 |
| 1 | hsa_55775 | D00528 |
| 1 | hsa_55811 | D00002 |
| 1 | hsa_558 | D01441 |
| 1 | hsa_558 | D01977 |
| 1 | hsa_558 | D03218 |
| 1 | hsa_558 | D03350 |
| 1 | hsa_558 | D04024 |
| 1 | hsa_558 | D04025 |
| 1 | hsa_55902 | D02769 |
| 1 | hsa_5594 | D01840 |
| 1 | hsa_5594 | D03115 |
| 1 | hsa_5594 | D03736 |
| 1 | hsa_5595 | D01840 |
| 1 | hsa_5595 | D03115 |
| 1 | hsa_5595 | D03736 |
| 1 | hsa_5596 | D01840 |
| 1 | hsa_5596 | D03115 |
| 1 | hsa_5596 | D03736 |
| 1 | hsa_5597 | D01840 |
| 1 | hsa_5597 | D03115 |
| 1 | hsa_5597 | D03736 |
| 1 | hsa_5598 | D01840 |
| 1 | hsa_5598 | D03115 |
| 1 | hsa_5598 | D03736 |
| 1 | hsa_5599 | D01840 |
| 1 | hsa_5599 | D03115 |
| 1 | hsa_5599 | D03736 |
| 1 | hsa_55 | D00103 |
| 1 | hsa_5600 | D01840 |
| 1 | hsa_5600 | D03115 |
| 1 | hsa_5600 | D03736 |
| 1 | hsa_5601 | D01840 |
| 1 | hsa_5601 | D03115 |
| 1 | hsa_5601 | D03736 |
| 1 | hsa_5602 | D01840 |
| 1 | hsa_5602 | D03115 |
| 1 | hsa_5602 | D03736 |
| 1 | hsa_5603 | D01840 |
| 1 | hsa_5603 | D03115 |
| 1 | hsa_5603 | D03736 |
| 1 | hsa_5604 | D01441 |
| 1 | hsa_5605 | D01441 |
| 1 | hsa_5606 | D01441 |
| 1 | hsa_5607 | D01441 |
| 1 | hsa_5608 | D01441 |
| 1 | hsa_5624 | D02335 |
| 1 | hsa_5625 | D00035 |
| 1 | hsa_5645 | D00043 |
| 1 | hsa_5645 | D00160 |
| 1 | hsa_5646 | D00043 |
| 1 | hsa_5646 | D00160 |
| 1 | hsa_5650 | D00043 |
| 1 | hsa_5650 | D00160 |
| 1 | hsa_5651 | D00043 |
| 1 | hsa_5651 | D00160 |
| 1 | hsa_5657 | D00043 |
| 1 | hsa_5657 | D00160 |
| 1 | hsa_56922 | D00029 |
| 1 | hsa_57016 | D00136 |
| 1 | hsa_57176 | D00039 |
| 1 | hsa_5740 | D00139 |
| 1 | hsa_5740 | D00225 |
| 1 | hsa_5740 | D00380 |
| 1 | hsa_5740 | D00394 |
| 1 | hsa_5740 | D00410 |
| 1 | hsa_5740 | D00437 |
| 1 | hsa_5740 | D00510 |
| 1 | hsa_5740 | D00528 |
| 1 | hsa_5740 | D00542 |
| 1 | hsa_5740 | D00574 |
| 1 | hsa_5740 | D01071 |
| 1 | hsa_5740 | D03670 |
| 1 | hsa_5742 | D00097 |
| 1 | hsa_5742 | D00109 |
| 1 | hsa_5742 | D00118 |
| 1 | hsa_5742 | D00120 |
| 1 | hsa_5742 | D00126 |
| 1 | hsa_5742 | D00127 |
| 1 | hsa_5742 | D00130 |
| 1 | hsa_5742 | D00132 |
| 1 | hsa_5742 | D00141 |
| 1 | hsa_5742 | D00158 |
| 1 | hsa_5742 | D00169 |
| 1 | hsa_5742 | D00217 |
| 1 | hsa_5742 | D00315 |
| 1 | hsa_5742 | D00330 |
| 1 | hsa_5742 | D00377 |
| 1 | hsa_5742 | D00425 |
| 1 | hsa_5742 | D00452 |
| 1 | hsa_5742 | D00463 |
| 1 | hsa_5742 | D00510 |
| 1 | hsa_5742 | D00566 |
| 1 | hsa_5742 | D00567 |
| 1 | hsa_5742 | D00568 |
| 1 | hsa_5742 | D00810 |
| 1 | hsa_5742 | D00813 |
| 1 | hsa_5742 | D00827 |
| 1 | hsa_5742 | D00903 |
| 1 | hsa_5742 | D00904 |
| 1 | hsa_5742 | D00968 |
| 1 | hsa_5742 | D00969 |
| 1 | hsa_5742 | D00970 |
| 1 | hsa_5742 | D01122 |
| 1 | hsa_5742 | D01183 |
| 1 | hsa_5742 | D01325 |
| 1 | hsa_5742 | D01364 |
| 1 | hsa_5742 | D01397 |
| 1 | hsa_5742 | D01475 |
| 1 | hsa_5742 | D01513 |
| 1 | hsa_5742 | D01547 |
| 1 | hsa_5742 | D01565 |
| 1 | hsa_5742 | D01578 |
| 1 | hsa_5742 | D01582 |
| 1 | hsa_5742 | D01709 |
| 1 | hsa_5742 | D01718 |
| 1 | hsa_5742 | D01765 |
| 1 | hsa_5742 | D01767 |
| 1 | hsa_5742 | D01811 |
| 1 | hsa_5742 | D01866 |
| 1 | hsa_5742 | D01974 |
| 1 | hsa_5742 | D02110 |
| 1 | hsa_5742 | D02290 |
| 1 | hsa_5742 | D02341 |
| 1 | hsa_5742 | D02350 |
| 1 | hsa_5742 | D02355 |
| 1 | hsa_5742 | D02709 |
| 1 | hsa_5742 | D03689 |
| 1 | hsa_5742 | D03710 |
| 1 | hsa_5742 | D03712 |
| 1 | hsa_5742 | D03714 |
| 1 | hsa_5742 | D03716 |
| 1 | hsa_5742 | D03717 |
| 1 | hsa_5743 | D00109 |
| 1 | hsa_5743 | D00118 |
| 1 | hsa_5743 | D00120 |
| 1 | hsa_5743 | D00126 |
| 1 | hsa_5743 | D00127 |
| 1 | hsa_5743 | D00130 |
| 1 | hsa_5743 | D00132 |
| 1 | hsa_5743 | D00141 |
| 1 | hsa_5743 | D00158 |
| 1 | hsa_5743 | D00169 |
| 1 | hsa_5743 | D00217 |
| 1 | hsa_5743 | D00315 |
| 1 | hsa_5743 | D00330 |
| 1 | hsa_5743 | D00377 |
| 1 | hsa_5743 | D00425 |
| 1 | hsa_5743 | D00449 |
| 1 | hsa_5743 | D00452 |
| 1 | hsa_5743 | D00463 |
| 1 | hsa_5743 | D00510 |
| 1 | hsa_5743 | D00566 |
| 1 | hsa_5743 | D00567 |
| 1 | hsa_5743 | D00568 |
| 1 | hsa_5743 | D00810 |
| 1 | hsa_5743 | D00813 |
| 1 | hsa_5743 | D00827 |
| 1 | hsa_5743 | D00903 |
| 1 | hsa_5743 | D00904 |
| 1 | hsa_5743 | D00968 |
| 1 | hsa_5743 | D00969 |
| 1 | hsa_5743 | D00970 |
| 1 | hsa_5743 | D01122 |
| 1 | hsa_5743 | D01183 |
| 1 | hsa_5743 | D01325 |
| 1 | hsa_5743 | D01364 |
| 1 | hsa_5743 | D01397 |
| 1 | hsa_5743 | D01475 |
| 1 | hsa_5743 | D01513 |
| 1 | hsa_5743 | D01547 |
| 1 | hsa_5743 | D01565 |
| 1 | hsa_5743 | D01578 |
| 1 | hsa_5743 | D01582 |
| 1 | hsa_5743 | D01709 |
| 1 | hsa_5743 | D01718 |
| 1 | hsa_5743 | D01765 |
| 1 | hsa_5743 | D01767 |
| 1 | hsa_5743 | D01811 |
| 1 | hsa_5743 | D01866 |
| 1 | hsa_5743 | D01974 |
| 1 | hsa_5743 | D02110 |
| 1 | hsa_5743 | D02290 |
| 1 | hsa_5743 | D02341 |
| 1 | hsa_5743 | D02350 |
| 1 | hsa_5743 | D02355 |
| 1 | hsa_5743 | D02709 |
| 1 | hsa_5743 | D03689 |
| 1 | hsa_5743 | D03710 |
| 1 | hsa_5743 | D03712 |
| 1 | hsa_5743 | D03714 |
| 1 | hsa_5743 | D03716 |
| 1 | hsa_5743 | D03717 |
| 1 | hsa_5747 | D01441 |
| 1 | hsa_5754 | D01441 |
| 1 | hsa_5754 | D01977 |
| 1 | hsa_5754 | D03218 |
| 1 | hsa_5754 | D03350 |
| 1 | hsa_5754 | D04024 |
| 1 | hsa_5754 | D04025 |
| 1 | hsa_57665 | D00501 |
| 1 | hsa_58190 | D00107 |
| 1 | hsa_58190 | D00184 |
| 1 | hsa_5831 | D00035 |
| 1 | hsa_5834 | D02769 |
| 1 | hsa_5836 | D02769 |
| 1 | hsa_5837 | D02769 |
| 1 | hsa_5860 | D00002 |
| 1 | hsa_586 | D00039 |
| 1 | hsa_586 | D00065 |
| 1 | hsa_586 | D00332 |
| 1 | hsa_587 | D00065 |
| 1 | hsa_587 | D00332 |
| 1 | hsa_590 | D00043 |
| 1 | hsa_590 | D00196 |
| 1 | hsa_590 | D00667 |
| 1 | hsa_590 | D00670 |
| 1 | hsa_590 | D00733 |
| 1 | hsa_590 | D00805 |
| 1 | hsa_590 | D01118 |
| 1 | hsa_590 | D01223 |
| 1 | hsa_590 | D02193 |
| 1 | hsa_590 | D02729 |
| 1 | hsa_590 | D03822 |
| 1 | hsa_5972 | D03738 |
| 1 | hsa_5972 | D03741 |
| 1 | hsa_5972 | D03743 |
| 1 | hsa_5979 | D01441 |
| 1 | hsa_5979 | D01977 |
| 1 | hsa_5979 | D03218 |
| 1 | hsa_5979 | D03350 |
| 1 | hsa_5979 | D04024 |
| 1 | hsa_5979 | D04025 |
| 1 | hsa_6098 | D01441 |
| 1 | hsa_6098 | D01977 |
| 1 | hsa_6098 | D03218 |
| 1 | hsa_6098 | D03350 |
| 1 | hsa_6098 | D04024 |
| 1 | hsa_6098 | D04025 |
| 1 | hsa_613 | D01441 |
| 1 | hsa_6240 | D01907 |
| 1 | hsa_6240 | D02368 |
| 1 | hsa_6240 | D03670 |
| 1 | hsa_6241 | D03670 |
| 1 | hsa_6259 | D01441 |
| 1 | hsa_6259 | D01977 |
| 1 | hsa_6259 | D03218 |
| 1 | hsa_6259 | D03350 |
| 1 | hsa_6259 | D04024 |
| 1 | hsa_6259 | D04025 |
| 1 | hsa_6300 | D01840 |
| 1 | hsa_6300 | D03115 |
| 1 | hsa_6300 | D03736 |
| 1 | hsa_63036 | D00043 |
| 1 | hsa_63036 | D00160 |
| 1 | hsa_635 | D00037 |
| 1 | hsa_63904 | D00107 |
| 1 | hsa_63904 | D00184 |
| 1 | hsa_64087 | D00029 |
| 1 | hsa_6416 | D01441 |
| 1 | hsa_64499 | D00043 |
| 1 | hsa_64499 | D00160 |
| 1 | hsa_645 | D00050 |
| 1 | hsa_64600 | D01223 |
| 1 | hsa_64802 | D00002 |
| 1 | hsa_64816 | D00139 |
| 1 | hsa_64816 | D00225 |
| 1 | hsa_64816 | D00380 |
| 1 | hsa_64816 | D00394 |
| 1 | hsa_64816 | D00410 |
| 1 | hsa_64816 | D00437 |
| 1 | hsa_64816 | D00528 |
| 1 | hsa_64816 | D00542 |
| 1 | hsa_64816 | D00574 |
| 1 | hsa_64816 | D00960 |
| 1 | hsa_64816 | D00964 |
| 1 | hsa_64816 | D01071 |
| 1 | hsa_64816 | D01425 |
| 1 | hsa_64816 | D02451 |
| 1 | hsa_64816 | D03670 |
| 1 | hsa_64816 | D03778 |
| 1 | hsa_64816 | D03781 |
| 1 | hsa_64816 | D03784 |
| 1 | hsa_64850 | D00332 |
| 1 | hsa_64902 | D00332 |
| 1 | hsa_657 | D01441 |
| 1 | hsa_658 | D01441 |
| 1 | hsa_6609 | D00417 |
| 1 | hsa_6609 | D00501 |
| 1 | hsa_6609 | D00528 |
| 1 | hsa_660 | D01441 |
| 1 | hsa_6610 | D00417 |
| 1 | hsa_6610 | D00501 |
| 1 | hsa_6610 | D00528 |
| 1 | hsa_6646 | D01966 |
| 1 | hsa_6646 | D03012 |
| 1 | hsa_6646 | D03734 |
| 1 | hsa_6646 | D03735 |
| 1 | hsa_6652 | D00002 |
| 1 | hsa_670 | D01223 |
| 1 | hsa_6713 | D02375 |
| 1 | hsa_6714 | D01441 |
| 1 | hsa_6716 | D03034 |
| 1 | hsa_6725 | D01441 |
| 1 | hsa_6768 | D00043 |
| 1 | hsa_6768 | D00160 |
| 1 | hsa_6799 | D00217 |
| 1 | hsa_6799 | D00418 |
| 1 | hsa_683 | D00036 |
| 1 | hsa_686 | D00029 |
| 1 | hsa_6897 | D00041 |
| 1 | hsa_6898 | D00021 |
| 1 | hsa_6898 | D00332 |
| 1 | hsa_695 | D01441 |
| 1 | hsa_7006 | D01441 |
| 1 | hsa_7010 | D01441 |
| 1 | hsa_7010 | D01977 |
| 1 | hsa_7010 | D03218 |
| 1 | hsa_7010 | D03350 |
| 1 | hsa_7010 | D04024 |
| 1 | hsa_7010 | D04025 |
| 1 | hsa_7015 | D00579 |
| 1 | hsa_7015 | D02267 |
| 1 | hsa_7046 | D01441 |
| 1 | hsa_7054 | D00021 |
| 1 | hsa_7054 | D00762 |
| 1 | hsa_7075 | D01441 |
| 1 | hsa_7075 | D01977 |
| 1 | hsa_7075 | D03218 |
| 1 | hsa_7075 | D03350 |
| 1 | hsa_7075 | D04024 |
| 1 | hsa_7075 | D04025 |
| 1 | hsa_7083 | D00584 |
| 1 | hsa_7084 | D00584 |
| 1 | hsa_7150 | D01061 |
| 1 | hsa_7150 | D01432 |
| 1 | hsa_7150 | D01911 |
| 1 | hsa_7150 | D02168 |
| 1 | hsa_7150 | D02756 |
| 1 | hsa_7150 | D04031 |
| 1 | hsa_7153 | D00125 |
| 1 | hsa_7153 | D00183 |
| 1 | hsa_7153 | D00186 |
| 1 | hsa_7153 | D01264 |
| 1 | hsa_7153 | D01275 |
| 1 | hsa_7153 | D01885 |
| 1 | hsa_7153 | D01911 |
| 1 | hsa_7153 | D02166 |
| 1 | hsa_7153 | D02214 |
| 1 | hsa_7153 | D02321 |
| 1 | hsa_7153 | D02333 |
| 1 | hsa_7153 | D02698 |
| 1 | hsa_7153 | D02756 |
| 1 | hsa_7153 | D03899 |
| 1 | hsa_7155 | D00125 |
| 1 | hsa_7155 | D00183 |
| 1 | hsa_7155 | D00186 |
| 1 | hsa_7155 | D01264 |
| 1 | hsa_7155 | D01275 |
| 1 | hsa_7155 | D01885 |
| 1 | hsa_7155 | D01911 |
| 1 | hsa_7155 | D02166 |
| 1 | hsa_7155 | D02214 |
| 1 | hsa_7155 | D02321 |
| 1 | hsa_7155 | D02333 |
| 1 | hsa_7155 | D02698 |
| 1 | hsa_7155 | D02756 |
| 1 | hsa_7155 | D03899 |
| 1 | hsa_7156 | D01061 |
| 1 | hsa_7156 | D01432 |
| 1 | hsa_7156 | D01911 |
| 1 | hsa_7156 | D02168 |
| 1 | hsa_7156 | D02756 |
| 1 | hsa_7156 | D04031 |
| 1 | hsa_7172 | D00377 |
| 1 | hsa_7173 | D00401 |
| 1 | hsa_7173 | D00562 |
| 1 | hsa_7174 | D00043 |
| 1 | hsa_7174 | D00160 |
| 1 | hsa_7294 | D01441 |
| 1 | hsa_7297 | D01441 |
| 1 | hsa_7298 | D00584 |
| 1 | hsa_7298 | D01064 |
| 1 | hsa_7298 | D01211 |
| 1 | hsa_7298 | D01223 |
| 1 | hsa_7298 | D02368 |
| 1 | hsa_7298 | D03828 |
| 1 | hsa_7298 | D04197 |
| 1 | hsa_7299 | D03034 |
| 1 | hsa_7301 | D01441 |
| 1 | hsa_7301 | D01977 |
| 1 | hsa_7301 | D03218 |
| 1 | hsa_7301 | D03350 |
| 1 | hsa_7301 | D04024 |
| 1 | hsa_7301 | D04025 |
| 1 | hsa_7363 | D01276 |
| 1 | hsa_7364 | D01276 |
| 1 | hsa_7365 | D01276 |
| 1 | hsa_7366 | D01276 |
| 1 | hsa_7367 | D01276 |
| 1 | hsa_7371 | D00037 |
| 1 | hsa_7372 | D00584 |
| 1 | hsa_7378 | D00584 |
| 1 | hsa_7453 | D00401 |
| 1 | hsa_7453 | D00562 |
| 1 | hsa_7498 | D00005 |
| 1 | hsa_7498 | D00224 |
| 1 | hsa_7525 | D01441 |
| 1 | hsa_7535 | D01441 |
| 1 | hsa_759 | D00218 |
| 1 | hsa_759 | D00294 |
| 1 | hsa_759 | D00340 |
| 1 | hsa_759 | D00518 |
| 1 | hsa_759 | D00519 |
| 1 | hsa_759 | D00538 |
| 1 | hsa_759 | D00650 |
| 1 | hsa_759 | D00651 |
| 1 | hsa_759 | D00652 |
| 1 | hsa_759 | D00653 |
| 1 | hsa_759 | D00654 |
| 1 | hsa_759 | D00655 |
| 1 | hsa_759 | D00656 |
| 1 | hsa_759 | D00658 |
| 1 | hsa_759 | D00703 |
| 1 | hsa_759 | D00709 |
| 1 | hsa_759 | D01196 |
| 1 | hsa_759 | D01256 |
| 1 | hsa_759 | D02356 |
| 1 | hsa_759 | D02441 |
| 1 | hsa_760 | D00218 |
| 1 | hsa_760 | D00294 |
| 1 | hsa_760 | D00340 |
| 1 | hsa_760 | D00518 |
| 1 | hsa_760 | D00519 |
| 1 | hsa_760 | D00537 |
| 1 | hsa_760 | D00538 |
| 1 | hsa_760 | D00650 |
| 1 | hsa_760 | D00651 |
| 1 | hsa_760 | D00652 |
| 1 | hsa_760 | D00653 |
| 1 | hsa_760 | D00654 |
| 1 | hsa_760 | D00655 |
| 1 | hsa_760 | D00656 |
| 1 | hsa_760 | D00658 |
| 1 | hsa_760 | D00703 |
| 1 | hsa_760 | D01196 |
| 1 | hsa_760 | D01256 |
| 1 | hsa_761 | D00218 |
| 1 | hsa_761 | D00340 |
| 1 | hsa_761 | D00518 |
| 1 | hsa_761 | D00519 |
| 1 | hsa_761 | D00538 |
| 1 | hsa_761 | D00652 |
| 1 | hsa_761 | D00653 |
| 1 | hsa_761 | D00655 |
| 1 | hsa_761 | D01196 |
| 1 | hsa_762 | D00218 |
| 1 | hsa_762 | D00294 |
| 1 | hsa_762 | D00340 |
| 1 | hsa_762 | D00518 |
| 1 | hsa_762 | D00519 |
| 1 | hsa_762 | D00537 |
| 1 | hsa_762 | D00538 |
| 1 | hsa_762 | D00650 |
| 1 | hsa_762 | D00651 |
| 1 | hsa_762 | D00652 |
| 1 | hsa_762 | D00653 |
| 1 | hsa_762 | D00654 |
| 1 | hsa_762 | D00655 |
| 1 | hsa_762 | D00656 |
| 1 | hsa_762 | D00658 |
| 1 | hsa_762 | D01196 |
| 1 | hsa_762 | D01256 |
| 1 | hsa_763 | D00218 |
| 1 | hsa_763 | D00340 |
| 1 | hsa_763 | D00518 |
| 1 | hsa_763 | D00519 |
| 1 | hsa_763 | D00538 |
| 1 | hsa_763 | D00652 |
| 1 | hsa_763 | D00653 |
| 1 | hsa_763 | D00655 |
| 1 | hsa_763 | D01196 |
| 1 | hsa_765 | D00218 |
| 1 | hsa_765 | D00340 |
| 1 | hsa_765 | D00518 |
| 1 | hsa_765 | D00519 |
| 1 | hsa_765 | D00538 |
| 1 | hsa_765 | D00652 |
| 1 | hsa_765 | D00653 |
| 1 | hsa_765 | D00655 |
| 1 | hsa_765 | D01196 |
| 1 | hsa_766 | D00218 |
| 1 | hsa_766 | D00340 |
| 1 | hsa_766 | D00518 |
| 1 | hsa_766 | D00519 |
| 1 | hsa_766 | D00538 |
| 1 | hsa_766 | D00652 |
| 1 | hsa_766 | D00653 |
| 1 | hsa_766 | D00655 |
| 1 | hsa_766 | D01196 |
| 1 | hsa_767 | D00218 |
| 1 | hsa_767 | D00340 |
| 1 | hsa_767 | D00518 |
| 1 | hsa_767 | D00519 |
| 1 | hsa_767 | D00538 |
| 1 | hsa_767 | D00652 |
| 1 | hsa_767 | D00653 |
| 1 | hsa_767 | D00655 |
| 1 | hsa_767 | D01196 |
| 1 | hsa_768 | D00218 |
| 1 | hsa_768 | D00340 |
| 1 | hsa_768 | D00518 |
| 1 | hsa_768 | D00519 |
| 1 | hsa_768 | D00538 |
| 1 | hsa_768 | D00652 |
| 1 | hsa_768 | D00653 |
| 1 | hsa_768 | D00655 |
| 1 | hsa_768 | D01196 |
| 1 | hsa_771 | D00218 |
| 1 | hsa_771 | D00340 |
| 1 | hsa_771 | D00518 |
| 1 | hsa_771 | D00519 |
| 1 | hsa_771 | D00538 |
| 1 | hsa_771 | D00652 |
| 1 | hsa_771 | D00653 |
| 1 | hsa_771 | D00655 |
| 1 | hsa_771 | D01196 |
| 1 | hsa_780 | D01441 |
| 1 | hsa_780 | D01977 |
| 1 | hsa_780 | D03218 |
| 1 | hsa_780 | D03350 |
| 1 | hsa_780 | D04024 |
| 1 | hsa_780 | D04025 |
| 1 | hsa_79001 | D02335 |
| 1 | hsa_79001 | D03798 |
| 1 | hsa_7957 | D00107 |
| 1 | hsa_7957 | D00184 |
| 1 | hsa_79799 | D01276 |
| 1 | hsa_80339 | D01223 |
| 1 | hsa_80339 | D04028 |
| 1 | hsa_80824 | D00107 |
| 1 | hsa_80824 | D00184 |
| 1 | hsa_81579 | D01223 |
| 1 | hsa_8192 | D00043 |
| 1 | hsa_8192 | D00160 |
| 1 | hsa_8288 | D00097 |
| 1 | hsa_8288 | D00217 |
| 1 | hsa_8288 | D00562 |
| 1 | hsa_8288 | D00577 |
| 1 | hsa_8288 | D03670 |
| 1 | hsa_834 | D00496 |
| 1 | hsa_8398 | D01223 |
| 1 | hsa_8399 | D01223 |
| 1 | hsa_84152 | D00107 |
| 1 | hsa_84152 | D00184 |
| 1 | hsa_84171 | D00270 |
| 1 | hsa_8435 | D01966 |
| 1 | hsa_8435 | D03012 |
| 1 | hsa_8435 | D03734 |
| 1 | hsa_8435 | D03735 |
| 1 | hsa_84532 | D02769 |
| 1 | hsa_84618 | D00501 |
| 1 | hsa_84695 | D00270 |
| 1 | hsa_84706 | D00332 |
| 1 | hsa_84812 | D00417 |
| 1 | hsa_84812 | D00501 |
| 1 | hsa_84812 | D00528 |
| 1 | hsa_8513 | D01223 |
| 1 | hsa_8513 | D04028 |
| 1 | hsa_8529 | D00139 |
| 1 | hsa_8529 | D00225 |
| 1 | hsa_8529 | D00380 |
| 1 | hsa_8529 | D00394 |
| 1 | hsa_8529 | D00410 |
| 1 | hsa_8529 | D00437 |
| 1 | hsa_8529 | D00528 |
| 1 | hsa_8529 | D00542 |
| 1 | hsa_8529 | D00574 |
| 1 | hsa_8529 | D01071 |
| 1 | hsa_8529 | D03670 |
| 1 | hsa_85313 | D00107 |
| 1 | hsa_8555 | D00107 |
| 1 | hsa_8555 | D00184 |
| 1 | hsa_8556 | D00107 |
| 1 | hsa_8556 | D00184 |
| 1 | hsa_8622 | D00227 |
| 1 | hsa_8622 | D00231 |
| 1 | hsa_8622 | D00371 |
| 1 | hsa_8622 | D00417 |
| 1 | hsa_8622 | D00501 |
| 1 | hsa_8622 | D00528 |
| 1 | hsa_8622 | D00691 |
| 1 | hsa_8622 | D01133 |
| 1 | hsa_8622 | D01198 |
| 1 | hsa_8622 | D01690 |
| 1 | hsa_8622 | D01704 |
| 1 | hsa_8622 | D01712 |
| 1 | hsa_8622 | D02008 |
| 1 | hsa_8622 | D02017 |
| 1 | hsa_8622 | D02042 |
| 1 | hsa_8622 | D02229 |
| 1 | hsa_8622 | D02655 |
| 1 | hsa_8622 | D02731 |
| 1 | hsa_8654 | D00037 |
| 1 | hsa_8654 | D00227 |
| 1 | hsa_8654 | D00231 |
| 1 | hsa_8654 | D00371 |
| 1 | hsa_8654 | D00417 |
| 1 | hsa_8654 | D00501 |
| 1 | hsa_8654 | D00528 |
| 1 | hsa_8654 | D01133 |
| 1 | hsa_8654 | D01198 |
| 1 | hsa_8654 | D01690 |
| 1 | hsa_8654 | D01704 |
| 1 | hsa_8654 | D01712 |
| 1 | hsa_8654 | D02008 |
| 1 | hsa_8654 | D02017 |
| 1 | hsa_8654 | D02042 |
| 1 | hsa_8654 | D02229 |
| 1 | hsa_8654 | D02655 |
| 1 | hsa_8654 | D02731 |
| 1 | hsa_8836 | D00070 |
| 1 | hsa_8854 | D00094 |
| 1 | hsa_8940 | D01061 |
| 1 | hsa_8940 | D01432 |
| 1 | hsa_8940 | D01911 |
| 1 | hsa_8940 | D02168 |
| 1 | hsa_8940 | D02756 |
| 1 | hsa_8940 | D04031 |
| 1 | hsa_8972 | D00216 |
| 1 | hsa_8972 | D00625 |
| 1 | hsa_8972 | D01665 |
| 1 | hsa_8972 | D03433 |
| 1 | hsa_9023 | D00139 |
| 1 | hsa_9023 | D00225 |
| 1 | hsa_9023 | D00380 |
| 1 | hsa_9023 | D00394 |
| 1 | hsa_9023 | D00410 |
| 1 | hsa_9023 | D00437 |
| 1 | hsa_9023 | D00528 |
| 1 | hsa_9023 | D00542 |
| 1 | hsa_9023 | D00574 |
| 1 | hsa_9023 | D01071 |
| 1 | hsa_9023 | D03670 |
| 1 | hsa_9088 | D01441 |
| 1 | hsa_90 | D01441 |
| 1 | hsa_91039 | D00043 |
| 1 | hsa_9150 | D00107 |
| 1 | hsa_9150 | D00184 |
| 1 | hsa_91 | D01441 |
| 1 | hsa_93650 | D00103 |
| 1 | hsa_9388 | D01223 |
| 1 | hsa_9388 | D04028 |
| 1 | hsa_93 | D01441 |
| 1 | hsa_94009 | D00043 |
| 1 | hsa_94009 | D00160 |
| 1 | hsa_9420 | D00139 |
| 1 | hsa_9420 | D00225 |
| 1 | hsa_9420 | D00380 |
| 1 | hsa_9420 | D00394 |
| 1 | hsa_9420 | D00410 |
| 1 | hsa_9420 | D00437 |
| 1 | hsa_9420 | D00528 |
| 1 | hsa_9420 | D00542 |
| 1 | hsa_9420 | D00574 |
| 1 | hsa_9420 | D01071 |
| 1 | hsa_94 | D01441 |
| 1 | hsa_9563 | D01223 |
| 1 | hsa_9601 | D03798 |
| 1 | hsa_9641 | D00448 |
| 1 | hsa_9647 | D00107 |
| 1 | hsa_9647 | D00184 |
| 1 | hsa_9945 | D00332 |
| 1 | hsa_9955 | D00037 |
| 2 | hsa_10056 | D00410 |
| 2 | hsa_10056 | D00494 |
| 2 | hsa_10056 | D00503 |
| 2 | hsa_10056 | D00650 |
| 2 | hsa_10056 | D00733 |
| 2 | hsa_10056 | D00995 |
| 2 | hsa_10056 | D01136 |
| 2 | hsa_10056 | D01825 |
| 2 | hsa_10056 | D02176 |
| 2 | hsa_10056 | D02451 |
| 2 | hsa_100 | D00005 |
| 2 | hsa_100 | D00039 |
| 2 | hsa_100 | D00283 |
| 2 | hsa_100 | D00753 |
| 2 | hsa_100 | D03765 |
| 2 | hsa_100 | D03776 |
| 2 | hsa_10188 | D00052 |
| 2 | hsa_10188 | D00127 |
| 2 | hsa_10188 | D00183 |
| 2 | hsa_10188 | D00216 |
| 2 | hsa_10188 | D00562 |
| 2 | hsa_10188 | D00650 |
| 2 | hsa_10188 | D01240 |
| 2 | hsa_10188 | D01667 |
| 2 | hsa_10188 | D03012 |
| 2 | hsa_10188 | D03717 |
| 2 | hsa_10188 | D03733 |
| 2 | hsa_10188 | D03752 |
| 2 | hsa_10269 | D00049 |
| 2 | hsa_10269 | D00130 |
| 2 | hsa_10269 | D00153 |
| 2 | hsa_10269 | D00903 |
| 2 | hsa_10269 | D00970 |
| 2 | hsa_10269 | D01984 |
| 2 | hsa_10269 | D02321 |
| 2 | hsa_10279 | D00394 |
| 2 | hsa_10279 | D00488 |
| 2 | hsa_10279 | D00960 |
| 2 | hsa_10279 | D01578 |
| 2 | hsa_10279 | D02168 |
| 2 | hsa_10279 | D02560 |
| 2 | hsa_10279 | D03775 |
| 2 | hsa_10295 | D00510 |
| 2 | hsa_10295 | D00771 |
| 2 | hsa_10295 | D00964 |
| 2 | hsa_10295 | D01183 |
| 2 | hsa_10295 | D02194 |
| 2 | hsa_10295 | D02258 |
| 2 | hsa_10295 | D02350 |
| 2 | hsa_10295 | D02356 |
| 2 | hsa_10327 | D00002 |
| 2 | hsa_10327 | D00070 |
| 2 | hsa_10327 | D00139 |
| 2 | hsa_10327 | D00421 |
| 2 | hsa_10327 | D00423 |
| 2 | hsa_10327 | D00656 |
| 2 | hsa_10327 | D01256 |
| 2 | hsa_10327 | D01907 |
| 2 | hsa_10327 | D01973 |
| 2 | hsa_10327 | D02350 |
| 2 | hsa_10327 | D02441 |
| 2 | hsa_1033 | D00130 |
| 2 | hsa_1033 | D00132 |
| 2 | hsa_1033 | D00196 |
| 2 | hsa_1033 | D00547 |
| 2 | hsa_1033 | D00574 |
| 2 | hsa_1033 | D00654 |
| 2 | hsa_1033 | D00893 |
| 2 | hsa_1033 | D01061 |
| 2 | hsa_1033 | D01811 |
| 2 | hsa_1033 | D01825 |
| 2 | hsa_1033 | D02166 |
| 2 | hsa_1033 | D02451 |
| 2 | hsa_1033 | D02671 |
| 2 | hsa_1033 | D03738 |
| 2 | hsa_10461 | D00224 |
| 2 | hsa_10461 | D00503 |
| 2 | hsa_10461 | D00622 |
| 2 | hsa_10461 | D00630 |
| 2 | hsa_10461 | D00965 |
| 2 | hsa_10461 | D02328 |
| 2 | hsa_10461 | D02562 |
| 2 | hsa_10461 | D03643 |
| 2 | hsa_10461 | D03778 |
| 2 | hsa_10461 | D05458 |
| 2 | hsa_10549 | D00451 |
| 2 | hsa_10549 | D00545 |
| 2 | hsa_10549 | D01332 |
| 2 | hsa_10549 | D02418 |
| 2 | hsa_10549 | D04197 |
| 2 | hsa_1056 | D00109 |
| 2 | hsa_1056 | D00145 |
| 2 | hsa_1056 | D00315 |
| 2 | hsa_1056 | D00330 |
| 2 | hsa_1056 | D00377 |
| 2 | hsa_1056 | D00401 |
| 2 | hsa_1056 | D00417 |
| 2 | hsa_1056 | D00568 |
| 2 | hsa_1056 | D00964 |
| 2 | hsa_1056 | D01001 |
| 2 | hsa_1056 | D01974 |
| 2 | hsa_1056 | D02115 |
| 2 | hsa_1056 | D02176 |
| 2 | hsa_1056 | D02559 |
| 2 | hsa_1056 | D03769 |
| 2 | hsa_10667 | D00018 |
| 2 | hsa_10667 | D00094 |
| 2 | hsa_10667 | D00332 |
| 2 | hsa_10667 | D00398 |
| 2 | hsa_10667 | D00651 |
| 2 | hsa_10667 | D00965 |
| 2 | hsa_10667 | D01704 |
| 2 | hsa_10667 | D01718 |
| 2 | hsa_10667 | D01866 |
| 2 | hsa_10667 | D01981 |
| 2 | hsa_10667 | D02487 |
| 2 | hsa_10667 | D02564 |
| 2 | hsa_10720 | D00107 |
| 2 | hsa_10720 | D00186 |
| 2 | hsa_10720 | D00620 |
| 2 | hsa_10720 | D00625 |
| 2 | hsa_10720 | D00947 |
| 2 | hsa_10720 | D02214 |
| 2 | hsa_10720 | D03743 |
| 2 | hsa_10747 | D00505 |
| 2 | hsa_10747 | D00726 |
| 2 | hsa_10747 | D00965 |
| 2 | hsa_10747 | D01475 |
| 2 | hsa_10747 | D02671 |
| 2 | hsa_10747 | D03218 |
| 2 | hsa_10747 | D03805 |
| 2 | hsa_10747 | D05458 |
| 2 | hsa_107 | D00043 |
| 2 | hsa_107 | D00055 |
| 2 | hsa_107 | D00183 |
| 2 | hsa_107 | D00596 |
| 2 | hsa_107 | D00969 |
| 2 | hsa_107 | D01211 |
| 2 | hsa_107 | D02258 |
| 2 | hsa_107 | D02580 |
| 2 | hsa_10825 | D00391 |
| 2 | hsa_10825 | D00516 |
| 2 | hsa_10825 | D00577 |
| 2 | hsa_10825 | D00623 |
| 2 | hsa_10825 | D00625 |
| 2 | hsa_10825 | D01475 |
| 2 | hsa_10825 | D02068 |
| 2 | hsa_10825 | D02168 |
| 2 | hsa_10825 | D03077 |
| 2 | hsa_10846 | D00035 |
| 2 | hsa_10846 | D00185 |
| 2 | hsa_10846 | D00448 |
| 2 | hsa_10846 | D01844 |
| 2 | hsa_10846 | D02166 |
| 2 | hsa_10846 | D02355 |
| 2 | hsa_10846 | D02580 |
| 2 | hsa_10846 | D03803 |
| 2 | hsa_10858 | D00131 |
| 2 | hsa_10858 | D00533 |
| 2 | hsa_10858 | D02368 |
| 2 | hsa_10858 | D02579 |
| 2 | hsa_10858 | D02655 |
| 2 | hsa_10858 | D02731 |
| 2 | hsa_10858 | D03077 |
| 2 | hsa_10858 | D03720 |
| 2 | hsa_10858 | D03763 |
| 2 | hsa_108 | D00052 |
| 2 | hsa_108 | D00537 |
| 2 | hsa_108 | D00785 |
| 2 | hsa_108 | D02342 |
| 2 | hsa_108 | D03643 |
| 2 | hsa_108 | D03670 |
| 2 | hsa_108 | D03733 |
| 2 | hsa_108 | D03778 |
| 2 | hsa_10901 | D00148 |
| 2 | hsa_10901 | D00377 |
| 2 | hsa_10901 | D00510 |
| 2 | hsa_10901 | D00622 |
| 2 | hsa_10901 | D00904 |
| 2 | hsa_10901 | D01061 |
| 2 | hsa_10901 | D01915 |
| 2 | hsa_10901 | D03670 |
| 2 | hsa_10901 | D03710 |
| 2 | hsa_10901 | D03735 |
| 2 | hsa_10901 | D03743 |
| 2 | hsa_10901 | D04292 |
| 2 | hsa_10924 | D00005 |
| 2 | hsa_10924 | D00333 |
| 2 | hsa_10924 | D00569 |
| 2 | hsa_10924 | D00826 |
| 2 | hsa_10924 | D01027 |
| 2 | hsa_10924 | D01071 |
| 2 | hsa_10924 | D01441 |
| 2 | hsa_10924 | D01513 |
| 2 | hsa_10924 | D02418 |
| 2 | hsa_10924 | D02769 |
| 2 | hsa_10935 | D00127 |
| 2 | hsa_10935 | D00187 |
| 2 | hsa_10935 | D00902 |
| 2 | hsa_10935 | D02173 |
| 2 | hsa_10935 | D02194 |
| 2 | hsa_10941 | D00142 |
| 2 | hsa_10941 | D00332 |
| 2 | hsa_10941 | D00369 |
| 2 | hsa_10941 | D00417 |
| 2 | hsa_10941 | D00421 |
| 2 | hsa_10941 | D00494 |
| 2 | hsa_10941 | D00620 |
| 2 | hsa_10941 | D00947 |
| 2 | hsa_10941 | D00964 |
| 2 | hsa_10941 | D02655 |
| 2 | hsa_109 | D00188 |
| 2 | hsa_109 | D00224 |
| 2 | hsa_109 | D00342 |
| 2 | hsa_109 | D00437 |
| 2 | hsa_109 | D00805 |
| 2 | hsa_109 | D01370 |
| 2 | hsa_109 | D02115 |
| 2 | hsa_109 | D02267 |
| 2 | hsa_109 | D02731 |
| 2 | hsa_109 | D03115 |
| 2 | hsa_109 | D03776 |
| 2 | hsa_109 | D03899 |
| 2 | hsa_10 | D00132 |
| 2 | hsa_10 | D00418 |
| 2 | hsa_10 | D00421 |
| 2 | hsa_10 | D00549 |
| 2 | hsa_10 | D00810 |
| 2 | hsa_10 | D01180 |
| 2 | hsa_10 | D01228 |
| 2 | hsa_10 | D01367 |
| 2 | hsa_10 | D01712 |
| 2 | hsa_10 | D01840 |
| 2 | hsa_10 | D01844 |
| 2 | hsa_10 | D02173 |
| 2 | hsa_10 | D03034 |
| 2 | hsa_10 | D03643 |
| 2 | hsa_10 | D03787 |
| 2 | hsa_11072 | D00036 |
| 2 | hsa_11072 | D00054 |
| 2 | hsa_11072 | D00120 |
| 2 | hsa_11072 | D00252 |
| 2 | hsa_11072 | D00519 |
| 2 | hsa_11072 | D00658 |
| 2 | hsa_11072 | D00826 |
| 2 | hsa_11072 | D02375 |
| 2 | hsa_11072 | D02756 |
| 2 | hsa_11072 | D03772 |
| 2 | hsa_11072 | D03798 |
| 2 | hsa_111 | D00055 |
| 2 | hsa_111 | D00125 |
| 2 | hsa_111 | D00187 |
| 2 | hsa_111 | D01183 |
| 2 | hsa_111 | D01441 |
| 2 | hsa_111 | D02333 |
| 2 | hsa_111 | D03798 |
| 2 | hsa_11202 | D00037 |
| 2 | hsa_11202 | D00315 |
| 2 | hsa_11202 | D00515 |
| 2 | hsa_11202 | D00562 |
| 2 | hsa_11202 | D00584 |
| 2 | hsa_11202 | D00653 |
| 2 | hsa_11202 | D02068 |
| 2 | hsa_11202 | D02418 |
| 2 | hsa_11202 | D03752 |
| 2 | hsa_11221 | D00208 |
| 2 | hsa_11221 | D00417 |
| 2 | hsa_11221 | D00519 |
| 2 | hsa_11221 | D00542 |
| 2 | hsa_11221 | D01136 |
| 2 | hsa_11221 | D02110 |
| 2 | hsa_11221 | D02559 |
| 2 | hsa_11221 | D03716 |
| 2 | hsa_11238 | D00038 |
| 2 | hsa_11238 | D00041 |
| 2 | hsa_11238 | D00107 |
| 2 | hsa_11238 | D00283 |
| 2 | hsa_11238 | D00322 |
| 2 | hsa_11238 | D00546 |
| 2 | hsa_11238 | D00827 |
| 2 | hsa_11238 | D01027 |
| 2 | hsa_11238 | D01122 |
| 2 | hsa_11266 | D00362 |
| 2 | hsa_11266 | D00503 |
| 2 | hsa_11266 | D00516 |
| 2 | hsa_11266 | D00549 |
| 2 | hsa_11266 | D02328 |
| 2 | hsa_11266 | D02341 |
| 2 | hsa_11266 | D03710 |
| 2 | hsa_112 | D00141 |
| 2 | hsa_112 | D00452 |
| 2 | hsa_112 | D00536 |
| 2 | hsa_112 | D00884 |
| 2 | hsa_112 | D01001 |
| 2 | hsa_112 | D01709 |
| 2 | hsa_112 | D02289 |
| 2 | hsa_112 | D02564 |
| 2 | hsa_112 | D04029 |
| 2 | hsa_112 | D04031 |
| 2 | hsa_11330 | D00039 |
| 2 | hsa_11330 | D00222 |
| 2 | hsa_11330 | D00225 |
| 2 | hsa_11330 | D00322 |
| 2 | hsa_11330 | D01122 |
| 2 | hsa_11330 | D01364 |
| 2 | hsa_11343 | D00038 |
| 2 | hsa_11343 | D00218 |
| 2 | hsa_11343 | D00813 |
| 2 | hsa_11343 | D00965 |
| 2 | hsa_11343 | D01001 |
| 2 | hsa_11343 | D01064 |
| 2 | hsa_11343 | D01966 |
| 2 | hsa_11343 | D02579 |
| 2 | hsa_11343 | D03722 |
| 2 | hsa_11343 | D03778 |
| 2 | hsa_11343 | D04966 |
| 2 | hsa_113 | D00285 |
| 2 | hsa_113 | D00294 |
| 2 | hsa_113 | D00325 |
| 2 | hsa_113 | D00655 |
| 2 | hsa_113 | D00703 |
| 2 | hsa_113 | D00995 |
| 2 | hsa_113 | D01069 |
| 2 | hsa_113 | D01211 |
| 2 | hsa_113 | D01973 |
| 2 | hsa_113 | D02304 |
| 2 | hsa_1147 | D00196 |
| 2 | hsa_1147 | D00401 |
| 2 | hsa_1147 | D00423 |
| 2 | hsa_1147 | D00494 |
| 2 | hsa_1147 | D00892 |
| 2 | hsa_1147 | D01276 |
| 2 | hsa_1147 | D01432 |
| 2 | hsa_1147 | D01475 |
| 2 | hsa_1147 | D02229 |
| 2 | hsa_1147 | D02580 |
| 2 | hsa_1147 | D03781 |
| 2 | hsa_114 | D00227 |
| 2 | hsa_114 | D00231 |
| 2 | hsa_114 | D00786 |
| 2 | hsa_114 | D01118 |
| 2 | hsa_114 | D01198 |
| 2 | hsa_114 | D01547 |
| 2 | hsa_115 | D00120 |
| 2 | hsa_115 | D00546 |
| 2 | hsa_115 | D02563 |
| 2 | hsa_115 | D03735 |
| 2 | hsa_116447 | D00018 |
| 2 | hsa_116447 | D00158 |
| 2 | hsa_116447 | D00667 |
| 2 | hsa_116447 | D00762 |
| 2 | hsa_116447 | D01578 |
| 2 | hsa_116447 | D01915 |
| 2 | hsa_116447 | D02562 |
| 2 | hsa_116447 | D02709 |
| 2 | hsa_116447 | D03733 |
| 2 | hsa_1178 | D00107 |
| 2 | hsa_1178 | D00118 |
| 2 | hsa_1178 | D00127 |
| 2 | hsa_1178 | D00155 |
| 2 | hsa_1178 | D00342 |
| 2 | hsa_1178 | D00394 |
| 2 | hsa_1178 | D01256 |
| 2 | hsa_1178 | D02566 |
| 2 | hsa_1200 | D00127 |
| 2 | hsa_1200 | D00285 |
| 2 | hsa_1200 | D00463 |
| 2 | hsa_1200 | D00513 |
| 2 | hsa_1200 | D00810 |
| 2 | hsa_1200 | D01367 |
| 2 | hsa_1200 | D02342 |
| 2 | hsa_1200 | D02368 |
| 2 | hsa_1200 | D04025 |
| 2 | hsa_1215 | D00065 |
| 2 | hsa_1215 | D00188 |
| 2 | hsa_1215 | D00279 |
| 2 | hsa_1215 | D00383 |
| 2 | hsa_1215 | D00455 |
| 2 | hsa_1215 | D00538 |
| 2 | hsa_1215 | D00568 |
| 2 | hsa_1215 | D01332 |
| 2 | hsa_1215 | D01840 |
| 2 | hsa_1215 | D02110 |
| 2 | hsa_1215 | D02308 |
| 2 | hsa_124 | D00283 |
| 2 | hsa_124 | D00521 |
| 2 | hsa_124 | D00596 |
| 2 | hsa_124 | D00885 |
| 2 | hsa_124 | D01240 |
| 2 | hsa_124 | D01547 |
| 2 | hsa_124 | D02441 |
| 2 | hsa_124 | D03807 |
| 2 | hsa_125 | D00294 |
| 2 | hsa_125 | D00455 |
| 2 | hsa_125 | D00535 |
| 2 | hsa_125 | D00537 |
| 2 | hsa_125 | D00546 |
| 2 | hsa_125 | D00998 |
| 2 | hsa_125 | D03776 |
| 2 | hsa_125 | D05407 |
| 2 | hsa_1267 | D00218 |
| 2 | hsa_1267 | D00270 |
| 2 | hsa_1267 | D00475 |
| 2 | hsa_1267 | D00892 |
| 2 | hsa_1267 | D00969 |
| 2 | hsa_1267 | D01547 |
| 2 | hsa_1267 | D01966 |
| 2 | hsa_126 | D00142 |
| 2 | hsa_126 | D00885 |
| 2 | hsa_126 | D00964 |
| 2 | hsa_126 | D01069 |
| 2 | hsa_126 | D01332 |
| 2 | hsa_126 | D02168 |
| 2 | hsa_126 | D03828 |
| 2 | hsa_128853 | D00148 |
| 2 | hsa_128853 | D00771 |
| 2 | hsa_128853 | D01907 |
| 2 | hsa_128 | D00045 |
| 2 | hsa_128 | D00094 |
| 2 | hsa_128 | D00332 |
| 2 | hsa_128 | D00566 |
| 2 | hsa_128 | D00969 |
| 2 | hsa_128 | D00970 |
| 2 | hsa_128 | D01888 |
| 2 | hsa_128 | D02335 |
| 2 | hsa_128 | D02350 |
| 2 | hsa_128 | D03728 |
| 2 | hsa_128 | D05341 |
| 2 | hsa_129807 | D00142 |
| 2 | hsa_129807 | D00494 |
| 2 | hsa_129807 | D01119 |
| 2 | hsa_130399 | D00005 |
| 2 | hsa_130399 | D00383 |
| 2 | hsa_130399 | D00496 |
| 2 | hsa_130399 | D00530 |
| 2 | hsa_130399 | D00651 |
| 2 | hsa_130399 | D00658 |
| 2 | hsa_130399 | D01136 |
| 2 | hsa_130399 | D01240 |
| 2 | hsa_130399 | D01256 |
| 2 | hsa_130399 | D02308 |
| 2 | hsa_130399 | D02563 |
| 2 | hsa_130399 | D03115 |
| 2 | hsa_130399 | D03760 |
| 2 | hsa_130399 | D04025 |
| 2 | hsa_1312 | D00029 |
| 2 | hsa_1312 | D00510 |
| 2 | hsa_1312 | D00543 |
| 2 | hsa_1312 | D00566 |
| 2 | hsa_1312 | D00904 |
| 2 | hsa_1312 | D01582 |
| 2 | hsa_1312 | D01704 |
| 2 | hsa_1312 | D04029 |
| 2 | hsa_131 | D00300 |
| 2 | hsa_131 | D00380 |
| 2 | hsa_131 | D00452 |
| 2 | hsa_131 | D00579 |
| 2 | hsa_131 | D02304 |
| 2 | hsa_131 | D03716 |
| 2 | hsa_132160 | D00038 |
| 2 | hsa_132160 | D00055 |
| 2 | hsa_132160 | D00070 |
| 2 | hsa_132160 | D00185 |
| 2 | hsa_132160 | D00315 |
| 2 | hsa_132160 | D00670 |
| 2 | hsa_132160 | D01198 |
| 2 | hsa_132160 | D01547 |
| 2 | hsa_132160 | D03218 |
| 2 | hsa_132 | D00107 |
| 2 | hsa_132 | D00414 |
| 2 | hsa_132 | D00887 |
| 2 | hsa_132 | D01275 |
| 2 | hsa_132 | D03670 |
| 2 | hsa_132 | D03741 |
| 2 | hsa_132 | D04197 |
| 2 | hsa_133121 | D00014 |
| 2 | hsa_133121 | D00125 |
| 2 | hsa_133121 | D00300 |
| 2 | hsa_133121 | D00998 |
| 2 | hsa_133121 | D04966 |
| 2 | hsa_134510 | D00423 |
| 2 | hsa_134510 | D00512 |
| 2 | hsa_134510 | D00536 |
| 2 | hsa_134510 | D00753 |
| 2 | hsa_134510 | D03805 |
| 2 | hsa_1360 | D00054 |
| 2 | hsa_1360 | D00203 |
| 2 | hsa_1360 | D00377 |
| 2 | hsa_1360 | D00414 |
| 2 | hsa_1360 | D00423 |
| 2 | hsa_1360 | D00563 |
| 2 | hsa_1360 | D00887 |
| 2 | hsa_1360 | D01547 |
| 2 | hsa_1360 | D01690 |
| 2 | hsa_1360 | D02110 |
| 2 | hsa_1360 | D03816 |
| 2 | hsa_1363 | D00142 |
| 2 | hsa_1363 | D00885 |
| 2 | hsa_1363 | D00947 |
| 2 | hsa_1363 | D01667 |
| 2 | hsa_1363 | D02168 |
| 2 | hsa_1363 | D02355 |
| 2 | hsa_1363 | D02487 |
| 2 | hsa_1363 | D03738 |
| 2 | hsa_1374 | D00014 |
| 2 | hsa_1374 | D00186 |
| 2 | hsa_1374 | D00900 |
| 2 | hsa_1374 | D00903 |
| 2 | hsa_1374 | D01211 |
| 2 | hsa_1374 | D01364 |
| 2 | hsa_1374 | D02561 |
| 2 | hsa_1374 | D05458 |
| 2 | hsa_1376 | D00510 |
| 2 | hsa_1376 | D00530 |
| 2 | hsa_1376 | D00829 |
| 2 | hsa_1376 | D01441 |
| 2 | hsa_1376 | D01811 |
| 2 | hsa_1376 | D01966 |
| 2 | hsa_1376 | D02756 |
| 2 | hsa_1376 | D03712 |
| 2 | hsa_1376 | D04028 |
| 2 | hsa_1384 | D00037 |
| 2 | hsa_1384 | D00364 |
| 2 | hsa_1384 | D00577 |
| 2 | hsa_1384 | D01122 |
| 2 | hsa_1384 | D01364 |
| 2 | hsa_1384 | D01918 |
| 2 | hsa_1384 | D02323 |
| 2 | hsa_1384 | D03736 |
| 2 | hsa_1384 | D03798 |
| 2 | hsa_13 | D00383 |
| 2 | hsa_13 | D00805 |
| 2 | hsa_13 | D01718 |
| 2 | hsa_13 | D01765 |
| 2 | hsa_13 | D02214 |
| 2 | hsa_13 | D02368 |
| 2 | hsa_13 | D03350 |
| 2 | hsa_13 | D04028 |
| 2 | hsa_142679 | D00039 |
| 2 | hsa_142679 | D00094 |
| 2 | hsa_142679 | D00383 |
| 2 | hsa_142679 | D00568 |
| 2 | hsa_142679 | D00622 |
| 2 | hsa_142679 | D00752 |
| 2 | hsa_142679 | D00882 |
| 2 | hsa_142679 | D01240 |
| 2 | hsa_142679 | D01364 |
| 2 | hsa_142679 | D01690 |
| 2 | hsa_142679 | D01977 |
| 2 | hsa_142679 | D03720 |
| 2 | hsa_1429 | D00021 |
| 2 | hsa_1429 | D00364 |
| 2 | hsa_1429 | D00398 |
| 2 | hsa_1429 | D02375 |
| 2 | hsa_1431 | D00169 |
| 2 | hsa_1431 | D00218 |
| 2 | hsa_1431 | D00325 |
| 2 | hsa_1431 | D00410 |
| 2 | hsa_1431 | D01133 |
| 2 | hsa_1431 | D01811 |
| 2 | hsa_1431 | D01974 |
| 2 | hsa_1431 | D02194 |
| 2 | hsa_1431 | D02368 |
| 2 | hsa_1432 | D00132 |
| 2 | hsa_1432 | D00168 |
| 2 | hsa_1432 | D00203 |
| 2 | hsa_1432 | D00359 |
| 2 | hsa_1432 | D00371 |
| 2 | hsa_1432 | D00387 |
| 2 | hsa_1432 | D00414 |
| 2 | hsa_1432 | D00416 |
| 2 | hsa_1432 | D00625 |
| 2 | hsa_1432 | D00781 |
| 2 | hsa_1432 | D01966 |
| 2 | hsa_1432 | D03714 |
| 2 | hsa_1436 | D00014 |
| 2 | hsa_1436 | D00963 |
| 2 | hsa_1436 | D02564 |
| 2 | hsa_1436 | D02655 |
| 2 | hsa_1436 | D03714 |
| 2 | hsa_1436 | D03776 |
| 2 | hsa_1436 | D03807 |
| 2 | hsa_1445 | D00132 |
| 2 | hsa_1445 | D00142 |
| 2 | hsa_1445 | D00184 |
| 2 | hsa_1445 | D00216 |
| 2 | hsa_1445 | D00234 |
| 2 | hsa_1445 | D00279 |
| 2 | hsa_1445 | D00401 |
| 2 | hsa_1445 | D00512 |
| 2 | hsa_1445 | D00513 |
| 2 | hsa_1445 | D00658 |
| 2 | hsa_1445 | D00805 |
| 2 | hsa_1445 | D00887 |
| 2 | hsa_1445 | D01256 |
| 2 | hsa_1445 | D01968 |
| 2 | hsa_1445 | D02333 |
| 2 | hsa_1445 | D02355 |
| 2 | hsa_1445 | D02375 |
| 2 | hsa_1445 | D03767 |
| 2 | hsa_1445 | D03806 |
| 2 | hsa_1445 | D05407 |
| 2 | hsa_150290 | D00579 |
| 2 | hsa_150290 | D00655 |
| 2 | hsa_150290 | D03807 |
| 2 | hsa_1504 | D00328 |
| 2 | hsa_1504 | D00330 |
| 2 | hsa_1504 | D00448 |
| 2 | hsa_1504 | D00533 |
| 2 | hsa_1504 | D00542 |
| 2 | hsa_1504 | D00995 |
| 2 | hsa_1504 | D01118 |
| 2 | hsa_1504 | D01425 |
| 2 | hsa_1504 | D01549 |
| 2 | hsa_1504 | D01866 |
| 2 | hsa_1504 | D03689 |
| 2 | hsa_151531 | D00007 |
| 2 | hsa_151531 | D00218 |
| 2 | hsa_151531 | D00650 |
| 2 | hsa_151531 | D01196 |
| 2 | hsa_151531 | D01325 |
| 2 | hsa_151531 | D01425 |
| 2 | hsa_151531 | D02335 |
| 2 | hsa_151531 | D02671 |
| 2 | hsa_151531 | D03710 |
| 2 | hsa_151531 | D04983 |
| 2 | hsa_1537 | D00225 |
| 2 | hsa_1537 | D00383 |
| 2 | hsa_1537 | D00650 |
| 2 | hsa_1537 | D00968 |
| 2 | hsa_1537 | D01475 |
| 2 | hsa_1537 | D01918 |
| 2 | hsa_1537 | D02214 |
| 2 | hsa_1543 | D00130 |
| 2 | hsa_1543 | D00148 |
| 2 | hsa_1543 | D00188 |
| 2 | hsa_1543 | D00274 |
| 2 | hsa_1543 | D00315 |
| 2 | hsa_1543 | D01549 |
| 2 | hsa_1543 | D01709 |
| 2 | hsa_1543 | D01885 |
| 2 | hsa_1543 | D03816 |
| 2 | hsa_1543 | D05407 |
| 2 | hsa_1544 | D00036 |
| 2 | hsa_1544 | D00065 |
| 2 | hsa_1544 | D00136 |
| 2 | hsa_1544 | D00185 |
| 2 | hsa_1544 | D00417 |
| 2 | hsa_1544 | D00448 |
| 2 | hsa_1544 | D00519 |
| 2 | hsa_1544 | D01133 |
| 2 | hsa_1544 | D02328 |
| 2 | hsa_1544 | D02563 |
| 2 | hsa_1544 | D02756 |
| 2 | hsa_1544 | D03601 |
| 2 | hsa_1544 | D03756 |
| 2 | hsa_1544 | D03805 |
| 2 | hsa_1545 | D00270 |
| 2 | hsa_1545 | D00538 |
| 2 | hsa_1545 | D00543 |
| 2 | hsa_1545 | D00550 |
| 2 | hsa_1545 | D00903 |
| 2 | hsa_1545 | D01097 |
| 2 | hsa_1545 | D01709 |
| 2 | hsa_1545 | D02267 |
| 2 | hsa_1545 | D02333 |
| 2 | hsa_1545 | D02556 |
| 2 | hsa_1545 | D02709 |
| 2 | hsa_1545 | D04292 |
| 2 | hsa_1548 | D00039 |
| 2 | hsa_1548 | D00196 |
| 2 | hsa_1548 | D00325 |
| 2 | hsa_1548 | D00383 |
| 2 | hsa_1548 | D00459 |
| 2 | hsa_1548 | D00518 |
| 2 | hsa_1548 | D00650 |
| 2 | hsa_1548 | D01061 |
| 2 | hsa_1548 | D01346 |
| 2 | hsa_1548 | D01549 |
| 2 | hsa_1548 | D02173 |
| 2 | hsa_1549 | D00118 |
| 2 | hsa_1549 | D00252 |
| 2 | hsa_1549 | D00516 |
| 2 | hsa_1549 | D00786 |
| 2 | hsa_1549 | D01118 |
| 2 | hsa_1549 | D02368 |
| 2 | hsa_1549 | D03440 |
| 2 | hsa_1549 | D03716 |
| 2 | hsa_1549 | D03752 |
| 2 | hsa_1551 | D00103 |
| 2 | hsa_1551 | D00118 |
| 2 | hsa_1551 | D00188 |
| 2 | hsa_1551 | D00198 |
| 2 | hsa_1551 | D00333 |
| 2 | hsa_1551 | D00518 |
| 2 | hsa_1551 | D00709 |
| 2 | hsa_1551 | D01198 |
| 2 | hsa_1551 | D01275 |
| 2 | hsa_1551 | D01718 |
| 2 | hsa_1551 | D01977 |
| 2 | hsa_1551 | D02729 |
| 2 | hsa_1553 | D00315 |
| 2 | hsa_1553 | D00513 |
| 2 | hsa_1553 | D00624 |
| 2 | hsa_1553 | D00903 |
| 2 | hsa_1553 | D00963 |
| 2 | hsa_1553 | D00965 |
| 2 | hsa_1553 | D01001 |
| 2 | hsa_1553 | D01275 |
| 2 | hsa_1553 | D01974 |
| 2 | hsa_1553 | D03826 |
| 2 | hsa_1555 | D00218 |
| 2 | hsa_1555 | D00651 |
| 2 | hsa_1555 | D00903 |
| 2 | hsa_1555 | D00995 |
| 2 | hsa_1555 | D01180 |
| 2 | hsa_1555 | D01240 |
| 2 | hsa_1555 | D01907 |
| 2 | hsa_1555 | D01966 |
| 2 | hsa_1555 | D02769 |
| 2 | hsa_1555 | D03760 |
| 2 | hsa_1557 | D00521 |
| 2 | hsa_1557 | D00994 |
| 2 | hsa_1557 | D02168 |
| 2 | hsa_1558 | D00007 |
| 2 | hsa_1558 | D00132 |
| 2 | hsa_1558 | D00274 |
| 2 | hsa_1558 | D00826 |
| 2 | hsa_1558 | D00893 |
| 2 | hsa_1558 | D00969 |
| 2 | hsa_1558 | D01397 |
| 2 | hsa_1558 | D01432 |
| 2 | hsa_1558 | D01667 |
| 2 | hsa_1558 | D02562 |
| 2 | hsa_1558 | D03716 |
| 2 | hsa_1559 | D00014 |
| 2 | hsa_1559 | D02835 |
| 2 | hsa_1559 | D03743 |
| 2 | hsa_1562 | D00070 |
| 2 | hsa_1562 | D00160 |
| 2 | hsa_1562 | D00168 |
| 2 | hsa_1562 | D00377 |
| 2 | hsa_1562 | D00494 |
| 2 | hsa_1562 | D00654 |
| 2 | hsa_1562 | D00813 |
| 2 | hsa_1562 | D00965 |
| 2 | hsa_1562 | D01211 |
| 2 | hsa_1562 | D01665 |
| 2 | hsa_1562 | D02559 |
| 2 | hsa_1562 | D03034 |
| 2 | hsa_1562 | D03733 |
| 2 | hsa_1565 | D00037 |
| 2 | hsa_1565 | D00188 |
| 2 | hsa_1565 | D00501 |
| 2 | hsa_1565 | D00579 |
| 2 | hsa_1565 | D00653 |
| 2 | hsa_1565 | D00826 |
| 2 | hsa_1565 | D00903 |
| 2 | hsa_1565 | D01690 |
| 2 | hsa_1565 | D01918 |
| 2 | hsa_1565 | D02017 |
| 2 | hsa_1565 | D02559 |
| 2 | hsa_1565 | D04028 |
| 2 | hsa_1571 | D00387 |
| 2 | hsa_1571 | D00753 |
| 2 | hsa_1571 | D01198 |
| 2 | hsa_1571 | D01918 |
| 2 | hsa_1571 | D02042 |
| 2 | hsa_1571 | D04983 |
| 2 | hsa_1572 | D00132 |
| 2 | hsa_1572 | D00892 |
| 2 | hsa_1572 | D01119 |
| 2 | hsa_1572 | D01767 |
| 2 | hsa_1572 | D01844 |
| 2 | hsa_1572 | D03735 |
| 2 | hsa_1573 | D00007 |
| 2 | hsa_1573 | D00038 |
| 2 | hsa_1573 | D00043 |
| 2 | hsa_1573 | D00070 |
| 2 | hsa_1573 | D00160 |
| 2 | hsa_1573 | D00359 |
| 2 | hsa_1573 | D01332 |
| 2 | hsa_1573 | D01712 |
| 2 | hsa_1573 | D01918 |
| 2 | hsa_1573 | D02562 |
| 2 | hsa_1573 | D03772 |
| 2 | hsa_1573 | D03775 |
| 2 | hsa_1573 | D04966 |
| 2 | hsa_1573 | D05341 |
| 2 | hsa_1576 | D00027 |
| 2 | hsa_1576 | D00324 |
| 2 | hsa_1576 | D00547 |
| 2 | hsa_1576 | D00620 |
| 2 | hsa_1576 | D00625 |
| 2 | hsa_1576 | D01688 |
| 2 | hsa_1576 | D02487 |
| 2 | hsa_1577 | D00109 |
| 2 | hsa_1577 | D00234 |
| 2 | hsa_1577 | D00650 |
| 2 | hsa_1577 | D00882 |
| 2 | hsa_1577 | D00884 |
| 2 | hsa_1577 | D01136 |
| 2 | hsa_1577 | D01866 |
| 2 | hsa_1577 | D01907 |
| 2 | hsa_1577 | D01981 |
| 2 | hsa_1577 | D03643 |
| 2 | hsa_1579 | D00185 |
| 2 | hsa_1579 | D00315 |
| 2 | hsa_1579 | D03350 |
| 2 | hsa_1579 | D04197 |
| 2 | hsa_1580 | D00501 |
| 2 | hsa_1580 | D00530 |
| 2 | hsa_1580 | D00651 |
| 2 | hsa_1580 | D00965 |
| 2 | hsa_1580 | D01061 |
| 2 | hsa_1580 | D03765 |
| 2 | hsa_1581 | D00007 |
| 2 | hsa_1581 | D00455 |
| 2 | hsa_1581 | D00463 |
| 2 | hsa_1581 | D00625 |
| 2 | hsa_1581 | D00995 |
| 2 | hsa_1581 | D01001 |
| 2 | hsa_1581 | D01180 |
| 2 | hsa_1581 | D01840 |
| 2 | hsa_1581 | D01888 |
| 2 | hsa_1581 | D03731 |
| 2 | hsa_1581 | D03752 |
| 2 | hsa_1582 | D00018 |
| 2 | hsa_1582 | D00294 |
| 2 | hsa_1582 | D00364 |
| 2 | hsa_1582 | D00566 |
| 2 | hsa_1582 | D00752 |
| 2 | hsa_1582 | D01425 |
| 2 | hsa_1582 | D02835 |
| 2 | hsa_1582 | D03805 |
| 2 | hsa_1583 | D00227 |
| 2 | hsa_1583 | D00251 |
| 2 | hsa_1583 | D00454 |
| 2 | hsa_1583 | D00538 |
| 2 | hsa_1583 | D01973 |
| 2 | hsa_1583 | D03743 |
| 2 | hsa_1584 | D00065 |
| 2 | hsa_1584 | D00118 |
| 2 | hsa_1584 | D00563 |
| 2 | hsa_1584 | D00904 |
| 2 | hsa_1584 | D00968 |
| 2 | hsa_1584 | D03756 |
| 2 | hsa_1584 | D03807 |
| 2 | hsa_1584 | D05407 |
| 2 | hsa_1585 | D00227 |
| 2 | hsa_1585 | D00620 |
| 2 | hsa_1585 | D00658 |
| 2 | hsa_1585 | D01709 |
| 2 | hsa_1585 | D03712 |
| 2 | hsa_1585 | D03753 |
| 2 | hsa_1586 | D00183 |
| 2 | hsa_1586 | D00328 |
| 2 | hsa_1586 | D00752 |
| 2 | hsa_1586 | D00805 |
| 2 | hsa_1586 | D00998 |
| 2 | hsa_1586 | D02315 |
| 2 | hsa_1586 | D02563 |
| 2 | hsa_1586 | D02729 |
| 2 | hsa_1586 | D03772 |
| 2 | hsa_1586 | D03778 |
| 2 | hsa_1586 | D04025 |
| 2 | hsa_1588 | D00533 |
| 2 | hsa_1588 | D00566 |
| 2 | hsa_1588 | D00620 |
| 2 | hsa_1588 | D01704 |
| 2 | hsa_1588 | D03751 |
| 2 | hsa_1588 | D03767 |
| 2 | hsa_1589 | D00198 |
| 2 | hsa_1589 | D00330 |
| 2 | hsa_1589 | D00537 |
| 2 | hsa_1589 | D00726 |
| 2 | hsa_1589 | D00762 |
| 2 | hsa_1589 | D00827 |
| 2 | hsa_1589 | D01578 |
| 2 | hsa_1589 | D01911 |
| 2 | hsa_1589 | D02068 |
| 2 | hsa_1589 | D02563 |
| 2 | hsa_1589 | D02655 |
| 2 | hsa_1589 | D03601 |
| 2 | hsa_1593 | D00148 |
| 2 | hsa_1593 | D00621 |
| 2 | hsa_1593 | D01918 |
| 2 | hsa_1593 | D03218 |
| 2 | hsa_1593 | D03722 |
| 2 | hsa_1593 | D03728 |
| 2 | hsa_1594 | D00196 |
| 2 | hsa_1594 | D00317 |
| 2 | hsa_1594 | D00622 |
| 2 | hsa_1594 | D00670 |
| 2 | hsa_1594 | D00781 |
| 2 | hsa_1594 | D00813 |
| 2 | hsa_1594 | D01119 |
| 2 | hsa_1594 | D01325 |
| 2 | hsa_1595 | D00285 |
| 2 | hsa_1595 | D01069 |
| 2 | hsa_1595 | D01223 |
| 2 | hsa_1595 | D02308 |
| 2 | hsa_1595 | D02579 |
| 2 | hsa_1610 | D00183 |
| 2 | hsa_1610 | D00283 |
| 2 | hsa_1610 | D00324 |
| 2 | hsa_1610 | D00359 |
| 2 | hsa_1610 | D02173 |
| 2 | hsa_1610 | D02580 |
| 2 | hsa_1610 | D03776 |
| 2 | hsa_1621 | D00136 |
| 2 | hsa_1621 | D00417 |
| 2 | hsa_1621 | D00488 |
| 2 | hsa_1621 | D00623 |
| 2 | hsa_1621 | D00994 |
| 2 | hsa_1621 | D00998 |
| 2 | hsa_1621 | D02115 |
| 2 | hsa_1621 | D02176 |
| 2 | hsa_1621 | D03720 |
| 2 | hsa_1621 | D03728 |
| 2 | hsa_1633 | D00103 |
| 2 | hsa_1633 | D00298 |
| 2 | hsa_1633 | D00596 |
| 2 | hsa_1633 | D01367 |
| 2 | hsa_1633 | D02289 |
| 2 | hsa_1636 | D00002 |
| 2 | hsa_1636 | D00130 |
| 2 | hsa_1636 | D00231 |
| 2 | hsa_1636 | D00656 |
| 2 | hsa_1636 | D01071 |
| 2 | hsa_1636 | D02451 |
| 2 | hsa_1636 | D04029 |
| 2 | hsa_1645 | D00141 |
| 2 | hsa_1645 | D00169 |
| 2 | hsa_1645 | D00503 |
| 2 | hsa_1645 | D00653 |
| 2 | hsa_1645 | D00893 |
| 2 | hsa_1645 | D01001 |
| 2 | hsa_1645 | D01862 |
| 2 | hsa_1675 | D00039 |
| 2 | hsa_1675 | D00054 |
| 2 | hsa_1675 | D00451 |
| 2 | hsa_1675 | D01180 |
| 2 | hsa_1675 | D01475 |
| 2 | hsa_1675 | D01549 |
| 2 | hsa_1675 | D03803 |
| 2 | hsa_1675 | D04024 |
| 2 | hsa_1719 | D00362 |
| 2 | hsa_1719 | D00550 |
| 2 | hsa_1719 | D00568 |
| 2 | hsa_1719 | D00726 |
| 2 | hsa_1719 | D01332 |
| 2 | hsa_1719 | D01364 |
| 2 | hsa_1719 | D03710 |
| 2 | hsa_1719 | D03712 |
| 2 | hsa_1719 | D03752 |
| 2 | hsa_1723 | D00224 |
| 2 | hsa_1723 | D00398 |
| 2 | hsa_1723 | D00425 |
| 2 | hsa_1723 | D00805 |
| 2 | hsa_1723 | D01071 |
| 2 | hsa_1723 | D01164 |
| 2 | hsa_1723 | D01196 |
| 2 | hsa_1723 | D01397 |
| 2 | hsa_1723 | D03828 |
| 2 | hsa_1725 | D00054 |
| 2 | hsa_1725 | D00342 |
| 2 | hsa_1725 | D00449 |
| 2 | hsa_1725 | D00494 |
| 2 | hsa_1725 | D00947 |
| 2 | hsa_1725 | D01275 |
| 2 | hsa_1725 | D01425 |
| 2 | hsa_1725 | D01915 |
| 2 | hsa_1725 | D02110 |
| 2 | hsa_1728 | D00038 |
| 2 | hsa_1728 | D00371 |
| 2 | hsa_1728 | D00451 |
| 2 | hsa_1728 | D00563 |
| 2 | hsa_1728 | D03807 |
| 2 | hsa_1728 | D03826 |
| 2 | hsa_1800 | D00227 |
| 2 | hsa_1800 | D00423 |
| 2 | hsa_1800 | D00733 |
| 2 | hsa_1800 | D01196 |
| 2 | hsa_1800 | D01346 |
| 2 | hsa_1800 | D02304 |
| 2 | hsa_1800 | D03115 |
| 2 | hsa_1803 | D00377 |
| 2 | hsa_1803 | D00623 |
| 2 | hsa_1803 | D01097 |
| 2 | hsa_1803 | D01198 |
| 2 | hsa_1803 | D01432 |
| 2 | hsa_1803 | D02335 |
| 2 | hsa_1806 | D00094 |
| 2 | hsa_1806 | D00960 |
| 2 | hsa_1806 | D01256 |
| 2 | hsa_1806 | D02194 |
| 2 | hsa_1806 | D02671 |
| 2 | hsa_1806 | D03753 |
| 2 | hsa_1843 | D00029 |
| 2 | hsa_1843 | D00198 |
| 2 | hsa_1843 | D00274 |
| 2 | hsa_1843 | D00380 |
| 2 | hsa_1843 | D00542 |
| 2 | hsa_1843 | D00785 |
| 2 | hsa_1843 | D00903 |
| 2 | hsa_1843 | D01275 |
| 2 | hsa_1843 | D01984 |
| 2 | hsa_1843 | D02110 |
| 2 | hsa_1843 | D02655 |
| 2 | hsa_1843 | D03077 |
| 2 | hsa_1843 | D03756 |
| 2 | hsa_1844 | D00155 |
| 2 | hsa_1844 | D00359 |
| 2 | hsa_1844 | D00425 |
| 2 | hsa_1844 | D00620 |
| 2 | hsa_1844 | D01432 |
| 2 | hsa_1844 | D02558 |
| 2 | hsa_1845 | D00656 |
| 2 | hsa_1845 | D01475 |
| 2 | hsa_1845 | D01888 |
| 2 | hsa_1845 | D02561 |
| 2 | hsa_1846 | D00018 |
| 2 | hsa_1846 | D00902 |
| 2 | hsa_1846 | D02214 |
| 2 | hsa_1846 | D02356 |
| 2 | hsa_1846 | D02561 |
| 2 | hsa_1846 | D02581 |
| 2 | hsa_1846 | D03758 |
| 2 | hsa_1847 | D00577 |
| 2 | hsa_1847 | D01709 |
| 2 | hsa_1847 | D01911 |
| 2 | hsa_1847 | D05458 |
| 2 | hsa_1848 | D00185 |
| 2 | hsa_1848 | D00528 |
| 2 | hsa_1848 | D00625 |
| 2 | hsa_1848 | D00653 |
| 2 | hsa_1848 | D00968 |
| 2 | hsa_1848 | D03077 |
| 2 | hsa_1849 | D00218 |
| 2 | hsa_1849 | D00330 |
| 2 | hsa_1849 | D00401 |
| 2 | hsa_1849 | D00709 |
| 2 | hsa_1849 | D01001 |
| 2 | hsa_1849 | D02328 |
| 2 | hsa_1849 | D02731 |
| 2 | hsa_1850 | D00120 |
| 2 | hsa_1850 | D00131 |
| 2 | hsa_1850 | D00362 |
| 2 | hsa_1850 | D00577 |
| 2 | hsa_1850 | D00752 |
| 2 | hsa_1850 | D00889 |
| 2 | hsa_1850 | D01180 |
| 2 | hsa_1850 | D01397 |
| 2 | hsa_1850 | D02193 |
| 2 | hsa_1850 | D02451 |
| 2 | hsa_1850 | D03773 |
| 2 | hsa_1852 | D00300 |
| 2 | hsa_1852 | D00425 |
| 2 | hsa_1852 | D00624 |
| 2 | hsa_1852 | D01765 |
| 2 | hsa_1852 | D01968 |
| 2 | hsa_1852 | D02267 |
| 2 | hsa_1852 | D02368 |
| 2 | hsa_1852 | D03758 |
| 2 | hsa_1852 | D04966 |
| 2 | hsa_1890 | D00045 |
| 2 | hsa_1890 | D00052 |
| 2 | hsa_1890 | D00120 |
| 2 | hsa_1890 | D00141 |
| 2 | hsa_1890 | D00155 |
| 2 | hsa_1890 | D00218 |
| 2 | hsa_1890 | D00621 |
| 2 | hsa_1890 | D00960 |
| 2 | hsa_1890 | D00968 |
| 2 | hsa_1890 | D02110 |
| 2 | hsa_1890 | D03433 |
| 2 | hsa_1890 | D03822 |
| 2 | hsa_189 | D00593 |
| 2 | hsa_189 | D00889 |
| 2 | hsa_189 | D01071 |
| 2 | hsa_189 | D01164 |
| 2 | hsa_189 | D01578 |
| 2 | hsa_189 | D02229 |
| 2 | hsa_189 | D02258 |
| 2 | hsa_189 | D02562 |
| 2 | hsa_189 | D03758 |
| 2 | hsa_18 | D00002 |
| 2 | hsa_18 | D00294 |
| 2 | hsa_18 | D00455 |
| 2 | hsa_18 | D00545 |
| 2 | hsa_18 | D01441 |
| 2 | hsa_18 | D02321 |
| 2 | hsa_18 | D02418 |
| 2 | hsa_18 | D03776 |
| 2 | hsa_191 | D00126 |
| 2 | hsa_191 | D00136 |
| 2 | hsa_191 | D00142 |
| 2 | hsa_191 | D00293 |
| 2 | hsa_191 | D00294 |
| 2 | hsa_191 | D00434 |
| 2 | hsa_191 | D00549 |
| 2 | hsa_191 | D01122 |
| 2 | hsa_191 | D02304 |
| 2 | hsa_191 | D03756 |
| 2 | hsa_191 | D03807 |
| 2 | hsa_1956 | D00126 |
| 2 | hsa_1956 | D00188 |
| 2 | hsa_1956 | D00359 |
| 2 | hsa_1956 | D00401 |
| 2 | hsa_1956 | D00546 |
| 2 | hsa_1956 | D01240 |
| 2 | hsa_1956 | D01915 |
| 2 | hsa_196883 | D00120 |
| 2 | hsa_196883 | D00380 |
| 2 | hsa_196883 | D00416 |
| 2 | hsa_196883 | D00501 |
| 2 | hsa_196883 | D00505 |
| 2 | hsa_196883 | D00528 |
| 2 | hsa_196883 | D01704 |
| 2 | hsa_196883 | D02418 |
| 2 | hsa_196883 | D02709 |
| 2 | hsa_1969 | D00518 |
| 2 | hsa_1969 | D00813 |
| 2 | hsa_1969 | D00826 |
| 2 | hsa_1969 | D01211 |
| 2 | hsa_1969 | D02115 |
| 2 | hsa_1969 | D02356 |
| 2 | hsa_1990 | D00036 |
| 2 | hsa_1990 | D00041 |
| 2 | hsa_1990 | D00496 |
| 2 | hsa_1990 | D03882 |
| 2 | hsa_1991 | D00032 |
| 2 | hsa_1991 | D00252 |
| 2 | hsa_1991 | D01180 |
| 2 | hsa_1991 | D02321 |
| 2 | hsa_1991 | D03769 |
| 2 | hsa_1991 | D04029 |
| 2 | hsa_1991 | D04966 |
| 2 | hsa_199974 | D00448 |
| 2 | hsa_199974 | D00513 |
| 2 | hsa_199974 | D00965 |
| 2 | hsa_199974 | D03767 |
| 2 | hsa_199974 | D03775 |
| 2 | hsa_2041 | D00027 |
| 2 | hsa_2041 | D00103 |
| 2 | hsa_2041 | D00141 |
| 2 | hsa_2041 | D00198 |
| 2 | hsa_2041 | D00219 |
| 2 | hsa_2041 | D00454 |
| 2 | hsa_2041 | D00752 |
| 2 | hsa_2041 | D00829 |
| 2 | hsa_2041 | D01027 |
| 2 | hsa_2041 | D01064 |
| 2 | hsa_2041 | D01547 |
| 2 | hsa_2041 | D01665 |
| 2 | hsa_2041 | D02563 |
| 2 | hsa_2041 | D02564 |
| 2 | hsa_2042 | D00185 |
| 2 | hsa_2042 | D00203 |
| 2 | hsa_2042 | D00496 |
| 2 | hsa_2042 | D00623 |
| 2 | hsa_2042 | D00752 |
| 2 | hsa_2042 | D00813 |
| 2 | hsa_2042 | D00970 |
| 2 | hsa_2042 | D01325 |
| 2 | hsa_2042 | D01718 |
| 2 | hsa_2042 | D02068 |
| 2 | hsa_2042 | D02308 |
| 2 | hsa_2043 | D00130 |
| 2 | hsa_2043 | D00533 |
| 2 | hsa_2043 | D00538 |
| 2 | hsa_2043 | D01027 |
| 2 | hsa_2043 | D02341 |
| 2 | hsa_2043 | D02368 |
| 2 | hsa_2043 | D03758 |
| 2 | hsa_2043 | D03805 |
| 2 | hsa_2044 | D00487 |
| 2 | hsa_2044 | D01061 |
| 2 | hsa_2044 | D01211 |
| 2 | hsa_2044 | D03735 |
| 2 | hsa_2044 | D03753 |
| 2 | hsa_2045 | D00097 |
| 2 | hsa_2045 | D00283 |
| 2 | hsa_2045 | D00452 |
| 2 | hsa_2045 | D00496 |
| 2 | hsa_2045 | D00652 |
| 2 | hsa_2045 | D00667 |
| 2 | hsa_2045 | D02115 |
| 2 | hsa_2045 | D02580 |
| 2 | hsa_2045 | D03440 |
| 2 | hsa_2045 | D03807 |
| 2 | hsa_2045 | D03882 |
| 2 | hsa_2045 | D05458 |
| 2 | hsa_2046 | D00394 |
| 2 | hsa_2046 | D00542 |
| 2 | hsa_2046 | D01432 |
| 2 | hsa_2046 | D01513 |
| 2 | hsa_2046 | D01862 |
| 2 | hsa_2046 | D02655 |
| 2 | hsa_2047 | D00448 |
| 2 | hsa_2047 | D00521 |
| 2 | hsa_2047 | D02731 |
| 2 | hsa_2048 | D00120 |
| 2 | hsa_2048 | D00136 |
| 2 | hsa_2048 | D00369 |
| 2 | hsa_2048 | D00459 |
| 2 | hsa_2048 | D00513 |
| 2 | hsa_2048 | D00752 |
| 2 | hsa_2048 | D02042 |
| 2 | hsa_2048 | D02229 |
| 2 | hsa_2048 | D03012 |
| 2 | hsa_2048 | D03741 |
| 2 | hsa_2049 | D00032 |
| 2 | hsa_2049 | D00103 |
| 2 | hsa_2049 | D00455 |
| 2 | hsa_2049 | D00463 |
| 2 | hsa_2049 | D00624 |
| 2 | hsa_2049 | D01900 |
| 2 | hsa_2049 | D02368 |
| 2 | hsa_2049 | D02487 |
| 2 | hsa_2049 | D02729 |
| 2 | hsa_2049 | D03765 |
| 2 | hsa_2049 | D03788 |
| 2 | hsa_2050 | D00142 |
| 2 | hsa_2050 | D00512 |
| 2 | hsa_2050 | D01367 |
| 2 | hsa_2050 | D01432 |
| 2 | hsa_2050 | D03717 |
| 2 | hsa_2050 | D03735 |
| 2 | hsa_2051 | D00217 |
| 2 | hsa_2051 | D00904 |
| 2 | hsa_2051 | D01712 |
| 2 | hsa_2051 | D02560 |
| 2 | hsa_2051 | D02581 |
| 2 | hsa_2051 | D03763 |
| 2 | hsa_2051 | D03787 |
| 2 | hsa_2058 | D00300 |
| 2 | hsa_2058 | D00398 |
| 2 | hsa_2058 | D00813 |
| 2 | hsa_2058 | D01119 |
| 2 | hsa_2058 | D01180 |
| 2 | hsa_2058 | D01842 |
| 2 | hsa_2058 | D02556 |
| 2 | hsa_2058 | D02562 |
| 2 | hsa_2058 | D03763 |
| 2 | hsa_2058 | D03767 |
| 2 | hsa_2064 | D00437 |
| 2 | hsa_2064 | D00574 |
| 2 | hsa_2065 | D00002 |
| 2 | hsa_2065 | D00187 |
| 2 | hsa_2065 | D00322 |
| 2 | hsa_2065 | D00566 |
| 2 | hsa_2065 | D01133 |
| 2 | hsa_2065 | D02110 |
| 2 | hsa_2065 | D02581 |
| 2 | hsa_2065 | D03077 |
| 2 | hsa_2065 | D03433 |
| 2 | hsa_2065 | D03733 |
| 2 | hsa_2066 | D00359 |
| 2 | hsa_2066 | D00463 |
| 2 | hsa_2066 | D00762 |
| 2 | hsa_2066 | D02350 |
| 2 | hsa_2098 | D00029 |
| 2 | hsa_2098 | D00188 |
| 2 | hsa_2098 | D00377 |
| 2 | hsa_2098 | D00463 |
| 2 | hsa_2098 | D00624 |
| 2 | hsa_2098 | D01346 |
| 2 | hsa_2098 | D03689 |
| 2 | hsa_2135 | D00103 |
| 2 | hsa_2135 | D00130 |
| 2 | hsa_2135 | D00217 |
| 2 | hsa_2135 | D00762 |
| 2 | hsa_2135 | D01973 |
| 2 | hsa_2135 | D02561 |
| 2 | hsa_2135 | D03034 |
| 2 | hsa_2135 | D03773 |
| 2 | hsa_2147 | D00620 |
| 2 | hsa_2147 | D01475 |
| 2 | hsa_2147 | D01709 |
| 2 | hsa_2147 | D02559 |
| 2 | hsa_2147 | D02729 |
| 2 | hsa_2147 | D02769 |
| 2 | hsa_2147 | D03350 |
| 2 | hsa_2147 | D03758 |
| 2 | hsa_2147 | D03767 |
| 2 | hsa_2147 | D03769 |
| 2 | hsa_2147 | D03776 |
| 2 | hsa_2155 | D00279 |
| 2 | hsa_2155 | D00369 |
| 2 | hsa_2155 | D00387 |
| 2 | hsa_2155 | D00623 |
| 2 | hsa_2155 | D00753 |
| 2 | hsa_2155 | D02560 |
| 2 | hsa_2155 | D03769 |
| 2 | hsa_2158 | D00027 |
| 2 | hsa_2158 | D00158 |
| 2 | hsa_2158 | D00184 |
| 2 | hsa_2158 | D00234 |
| 2 | hsa_2158 | D00270 |
| 2 | hsa_2158 | D00521 |
| 2 | hsa_2158 | D00964 |
| 2 | hsa_2158 | D01164 |
| 2 | hsa_2158 | D01973 |
| 2 | hsa_2158 | D01981 |
| 2 | hsa_2158 | D02342 |
| 2 | hsa_2159 | D00094 |
| 2 | hsa_2159 | D00184 |
| 2 | hsa_2159 | D01915 |
| 2 | hsa_2159 | D03712 |
| 2 | hsa_2159 | D03736 |
| 2 | hsa_2159 | D03752 |
| 2 | hsa_2160 | D00463 |
| 2 | hsa_2160 | D00826 |
| 2 | hsa_2160 | D00960 |
| 2 | hsa_2160 | D02176 |
| 2 | hsa_2160 | D02289 |
| 2 | hsa_2160 | D02355 |
| 2 | hsa_2160 | D02559 |
| 2 | hsa_2160 | D03731 |
| 2 | hsa_2160 | D03775 |
| 2 | hsa_2160 | D05458 |
| 2 | hsa_2161 | D00153 |
| 2 | hsa_2161 | D00216 |
| 2 | hsa_2161 | D00387 |
| 2 | hsa_2161 | D01842 |
| 2 | hsa_2161 | D01984 |
| 2 | hsa_216 | D00043 |
| 2 | hsa_216 | D00120 |
| 2 | hsa_216 | D00217 |
| 2 | hsa_216 | D00401 |
| 2 | hsa_216 | D00451 |
| 2 | hsa_216 | D00549 |
| 2 | hsa_216 | D01665 |
| 2 | hsa_216 | D02308 |
| 2 | hsa_216 | D02731 |
| 2 | hsa_216 | D03775 |
| 2 | hsa_217 | D00039 |
| 2 | hsa_217 | D00270 |
| 2 | hsa_217 | D00387 |
| 2 | hsa_217 | D00434 |
| 2 | hsa_217 | D01211 |
| 2 | hsa_217 | D01866 |
| 2 | hsa_217 | D01888 |
| 2 | hsa_217 | D02341 |
| 2 | hsa_217 | D03034 |
| 2 | hsa_2180 | D00414 |
| 2 | hsa_2180 | D00892 |
| 2 | hsa_2180 | D01061 |
| 2 | hsa_2180 | D01513 |
| 2 | hsa_2180 | D01844 |
| 2 | hsa_2180 | D01918 |
| 2 | hsa_2180 | D03822 |
| 2 | hsa_2185 | D00045 |
| 2 | hsa_2185 | D00049 |
| 2 | hsa_2185 | D00126 |
| 2 | hsa_2185 | D00560 |
| 2 | hsa_2185 | D00752 |
| 2 | hsa_2185 | D00829 |
| 2 | hsa_2185 | D02328 |
| 2 | hsa_2185 | D03689 |
| 2 | hsa_2185 | D03720 |
| 2 | hsa_2185 | D03728 |
| 2 | hsa_2185 | D03807 |
| 2 | hsa_218 | D00050 |
| 2 | hsa_218 | D00216 |
| 2 | hsa_218 | D00369 |
| 2 | hsa_218 | D00423 |
| 2 | hsa_218 | D00544 |
| 2 | hsa_218 | D00621 |
| 2 | hsa_218 | D00970 |
| 2 | hsa_218 | D01097 |
| 2 | hsa_218 | D02756 |
| 2 | hsa_218 | D03716 |
| 2 | hsa_218 | D03765 |
| 2 | hsa_2193 | D00168 |
| 2 | hsa_2193 | D00496 |
| 2 | hsa_2193 | D00501 |
| 2 | hsa_2193 | D00537 |
| 2 | hsa_2193 | D01164 |
| 2 | hsa_2193 | D01240 |
| 2 | hsa_2193 | D02308 |
| 2 | hsa_2193 | D02375 |
| 2 | hsa_219 | D00054 |
| 2 | hsa_219 | D00139 |
| 2 | hsa_219 | D00330 |
| 2 | hsa_219 | D00567 |
| 2 | hsa_219 | D00658 |
| 2 | hsa_219 | D00885 |
| 2 | hsa_219 | D00969 |
| 2 | hsa_219 | D01690 |
| 2 | hsa_219 | D01866 |
| 2 | hsa_219 | D02194 |
| 2 | hsa_219 | D02267 |
| 2 | hsa_219 | D02709 |
| 2 | hsa_219 | D03350 |
| 2 | hsa_219 | D03775 |
| 2 | hsa_219 | D03781 |
| 2 | hsa_2224 | D00186 |
| 2 | hsa_2224 | D00475 |
| 2 | hsa_2224 | D00515 |
| 2 | hsa_2224 | D03077 |
| 2 | hsa_2232 | D00224 |
| 2 | hsa_2232 | D00781 |
| 2 | hsa_2232 | D00827 |
| 2 | hsa_2232 | D01264 |
| 2 | hsa_2232 | D01885 |
| 2 | hsa_2235 | D00052 |
| 2 | hsa_2235 | D00488 |
| 2 | hsa_2235 | D00535 |
| 2 | hsa_2235 | D00813 |
| 2 | hsa_2235 | D02068 |
| 2 | hsa_2235 | D02315 |
| 2 | hsa_2235 | D02350 |
| 2 | hsa_2235 | D04029 |
| 2 | hsa_223 | D00038 |
| 2 | hsa_223 | D00651 |
| 2 | hsa_223 | D00965 |
| 2 | hsa_223 | D03736 |
| 2 | hsa_2241 | D00049 |
| 2 | hsa_2241 | D00387 |
| 2 | hsa_2241 | D00417 |
| 2 | hsa_2241 | D00535 |
| 2 | hsa_2241 | D00566 |
| 2 | hsa_2241 | D01547 |
| 2 | hsa_2241 | D03012 |
| 2 | hsa_2241 | D03350 |
| 2 | hsa_2242 | D00021 |
| 2 | hsa_2242 | D00029 |
| 2 | hsa_2242 | D00097 |
| 2 | hsa_2242 | D00252 |
| 2 | hsa_2242 | D00332 |
| 2 | hsa_2242 | D00342 |
| 2 | hsa_2242 | D00394 |
| 2 | hsa_2242 | D00487 |
| 2 | hsa_2242 | D01196 |
| 2 | hsa_2242 | D01981 |
| 2 | hsa_2242 | D02556 |
| 2 | hsa_2242 | D03788 |
| 2 | hsa_224 | D00049 |
| 2 | hsa_224 | D00252 |
| 2 | hsa_224 | D03601 |
| 2 | hsa_224 | D03758 |
| 2 | hsa_225689 | D00158 |
| 2 | hsa_225689 | D00219 |
| 2 | hsa_225689 | D00251 |
| 2 | hsa_225689 | D00285 |
| 2 | hsa_225689 | D00545 |
| 2 | hsa_225689 | D00650 |
| 2 | hsa_225689 | D01256 |
| 2 | hsa_225689 | D01842 |
| 2 | hsa_225689 | D02323 |
| 2 | hsa_2260 | D00054 |
| 2 | hsa_2260 | D00231 |
| 2 | hsa_2260 | D00325 |
| 2 | hsa_2260 | D00753 |
| 2 | hsa_2260 | D00900 |
| 2 | hsa_2260 | D01397 |
| 2 | hsa_2260 | D02580 |
| 2 | hsa_2261 | D00203 |
| 2 | hsa_2261 | D00401 |
| 2 | hsa_2261 | D02193 |
| 2 | hsa_2261 | D02229 |
| 2 | hsa_2261 | D02769 |
| 2 | hsa_2261 | D03775 |
| 2 | hsa_2263 | D00043 |
| 2 | hsa_2263 | D00283 |
| 2 | hsa_2263 | D00398 |
| 2 | hsa_2263 | D00410 |
| 2 | hsa_2263 | D01547 |
| 2 | hsa_2263 | D02115 |
| 2 | hsa_2264 | D00139 |
| 2 | hsa_2264 | D00225 |
| 2 | hsa_2264 | D00383 |
| 2 | hsa_2264 | D01704 |
| 2 | hsa_2264 | D01918 |
| 2 | hsa_2264 | D01984 |
| 2 | hsa_2264 | D03712 |
| 2 | hsa_2280 | D00049 |
| 2 | hsa_2280 | D01704 |
| 2 | hsa_2280 | D01885 |
| 2 | hsa_2280 | D02350 |
| 2 | hsa_22843 | D00018 |
| 2 | hsa_22843 | D00103 |
| 2 | hsa_22843 | D00421 |
| 2 | hsa_22843 | D00518 |
| 2 | hsa_22843 | D00535 |
| 2 | hsa_22843 | D00691 |
| 2 | hsa_22843 | D00709 |
| 2 | hsa_22843 | D00970 |
| 2 | hsa_22843 | D03218 |
| 2 | hsa_22843 | D03440 |
| 2 | hsa_22954 | D00141 |
| 2 | hsa_22954 | D00401 |
| 2 | hsa_22954 | D00449 |
| 2 | hsa_22954 | D00968 |
| 2 | hsa_22954 | D01001 |
| 2 | hsa_22954 | D01164 |
| 2 | hsa_22954 | D01196 |
| 2 | hsa_22954 | D01240 |
| 2 | hsa_22954 | D02368 |
| 2 | hsa_22954 | D02756 |
| 2 | hsa_22954 | D04983 |
| 2 | hsa_22978 | D00055 |
| 2 | hsa_22978 | D00187 |
| 2 | hsa_22978 | D00196 |
| 2 | hsa_22978 | D00218 |
| 2 | hsa_22978 | D00622 |
| 2 | hsa_22978 | D00655 |
| 2 | hsa_22978 | D01119 |
| 2 | hsa_22978 | D01367 |
| 2 | hsa_22978 | D01667 |
| 2 | hsa_22978 | D03689 |
| 2 | hsa_22978 | D03722 |
| 2 | hsa_22978 | D03787 |
| 2 | hsa_22978 | D03798 |
| 2 | hsa_22978 | D03828 |
| 2 | hsa_23035 | D00097 |
| 2 | hsa_23035 | D00158 |
| 2 | hsa_23035 | D00501 |
| 2 | hsa_23035 | D00515 |
| 2 | hsa_23035 | D00546 |
| 2 | hsa_23035 | D00560 |
| 2 | hsa_23035 | D02008 |
| 2 | hsa_23035 | D03731 |
| 2 | hsa_231 | D00055 |
| 2 | hsa_231 | D00132 |
| 2 | hsa_231 | D00569 |
| 2 | hsa_231 | D00810 |
| 2 | hsa_231 | D00968 |
| 2 | hsa_231 | D01918 |
| 2 | hsa_231 | D02166 |
| 2 | hsa_231 | D04029 |
| 2 | hsa_2321 | D00045 |
| 2 | hsa_2321 | D00094 |
| 2 | hsa_2321 | D00577 |
| 2 | hsa_2321 | D00893 |
| 2 | hsa_2321 | D00960 |
| 2 | hsa_2321 | D01667 |
| 2 | hsa_2321 | D01981 |
| 2 | hsa_2321 | D02166 |
| 2 | hsa_2321 | D02564 |
| 2 | hsa_2322 | D00027 |
| 2 | hsa_2322 | D00029 |
| 2 | hsa_2322 | D00050 |
| 2 | hsa_2322 | D00505 |
| 2 | hsa_2322 | D00893 |
| 2 | hsa_2322 | D01180 |
| 2 | hsa_2322 | D01767 |
| 2 | hsa_2322 | D02566 |
| 2 | hsa_2322 | D06238 |
| 2 | hsa_23236 | D00596 |
| 2 | hsa_23236 | D01370 |
| 2 | hsa_23236 | D01582 |
| 2 | hsa_23236 | D01974 |
| 2 | hsa_23236 | D02193 |
| 2 | hsa_23236 | D03712 |
| 2 | hsa_23236 | D04966 |
| 2 | hsa_23239 | D00371 |
| 2 | hsa_23239 | D00496 |
| 2 | hsa_23239 | D00535 |
| 2 | hsa_23239 | D00658 |
| 2 | hsa_23239 | D00762 |
| 2 | hsa_23239 | D01973 |
| 2 | hsa_23239 | D02487 |
| 2 | hsa_23239 | D02559 |
| 2 | hsa_23239 | D03769 |
| 2 | hsa_2324 | D00120 |
| 2 | hsa_2324 | D00274 |
| 2 | hsa_2324 | D00521 |
| 2 | hsa_2324 | D00566 |
| 2 | hsa_2324 | D00584 |
| 2 | hsa_2324 | D00889 |
| 2 | hsa_2324 | D01547 |
| 2 | hsa_2324 | D01715 |
| 2 | hsa_2324 | D02304 |
| 2 | hsa_2324 | D03722 |
| 2 | hsa_2324 | D04028 |
| 2 | hsa_2326 | D01425 |
| 2 | hsa_2326 | D02017 |
| 2 | hsa_2326 | D03115 |
| 2 | hsa_2326 | D03773 |
| 2 | hsa_2328 | D00463 |
| 2 | hsa_2328 | D00528 |
| 2 | hsa_2328 | D00658 |
| 2 | hsa_2328 | D00805 |
| 2 | hsa_2328 | D00827 |
| 2 | hsa_2328 | D02418 |
| 2 | hsa_2328 | D03882 |
| 2 | hsa_2328 | D04966 |
| 2 | hsa_2328 | D06238 |
| 2 | hsa_2339 | D00623 |
| 2 | hsa_2339 | D00947 |
| 2 | hsa_2339 | D00964 |
| 2 | hsa_2339 | D02194 |
| 2 | hsa_2339 | D02315 |
| 2 | hsa_2339 | D02698 |
| 2 | hsa_2342 | D00425 |
| 2 | hsa_2342 | D02068 |
| 2 | hsa_2342 | D02333 |
| 2 | hsa_2342 | D03767 |
| 2 | hsa_23430 | D00052 |
| 2 | hsa_23430 | D00882 |
| 2 | hsa_23430 | D00969 |
| 2 | hsa_23430 | D01364 |
| 2 | hsa_23430 | D03736 |
| 2 | hsa_23430 | D04292 |
| 2 | hsa_23430 | D04983 |
| 2 | hsa_23436 | D00070 |
| 2 | hsa_23436 | D00142 |
| 2 | hsa_23436 | D00148 |
| 2 | hsa_23436 | D00294 |
| 2 | hsa_23436 | D00568 |
| 2 | hsa_23436 | D00579 |
| 2 | hsa_23436 | D00654 |
| 2 | hsa_23436 | D00902 |
| 2 | hsa_23436 | D00998 |
| 2 | hsa_23436 | D01164 |
| 2 | hsa_23436 | D01325 |
| 2 | hsa_23436 | D03751 |
| 2 | hsa_23475 | D00139 |
| 2 | hsa_23475 | D00279 |
| 2 | hsa_23475 | D00421 |
| 2 | hsa_23475 | D00620 |
| 2 | hsa_23475 | D00625 |
| 2 | hsa_23475 | D00670 |
| 2 | hsa_23475 | D00903 |
| 2 | hsa_23475 | D01183 |
| 2 | hsa_23475 | D02835 |
| 2 | hsa_23475 | D03433 |
| 2 | hsa_23475 | D03767 |
| 2 | hsa_23475 | D03882 |
| 2 | hsa_2356 | D00036 |
| 2 | hsa_2356 | D00186 |
| 2 | hsa_2356 | D01370 |
| 2 | hsa_2356 | D01915 |
| 2 | hsa_2356 | D01974 |
| 2 | hsa_23632 | D00398 |
| 2 | hsa_23632 | D00455 |
| 2 | hsa_23632 | D00510 |
| 2 | hsa_23632 | D00651 |
| 2 | hsa_23632 | D01264 |
| 2 | hsa_23632 | D01578 |
| 2 | hsa_23632 | D03736 |
| 2 | hsa_23632 | D04029 |
| 2 | hsa_238 | D00332 |
| 2 | hsa_238 | D00658 |
| 2 | hsa_238 | D02176 |
| 2 | hsa_238 | D02214 |
| 2 | hsa_239 | D00005 |
| 2 | hsa_239 | D00014 |
| 2 | hsa_239 | D00027 |
| 2 | hsa_239 | D00136 |
| 2 | hsa_239 | D00298 |
| 2 | hsa_239 | D00893 |
| 2 | hsa_239 | D01223 |
| 2 | hsa_239 | D01578 |
| 2 | hsa_239 | D02323 |
| 2 | hsa_239 | D02328 |
| 2 | hsa_240 | D00065 |
| 2 | hsa_240 | D00650 |
| 2 | hsa_240 | D00656 |
| 2 | hsa_240 | D00995 |
| 2 | hsa_240 | D03218 |
| 2 | hsa_240 | D03440 |
| 2 | hsa_240 | D03710 |
| 2 | hsa_240 | D03781 |
| 2 | hsa_240 | D05458 |
| 2 | hsa_242 | D00038 |
| 2 | hsa_242 | D00127 |
| 2 | hsa_242 | D00168 |
| 2 | hsa_242 | D00216 |
| 2 | hsa_242 | D00251 |
| 2 | hsa_242 | D00330 |
| 2 | hsa_242 | D00434 |
| 2 | hsa_242 | D00651 |
| 2 | hsa_242 | D01122 |
| 2 | hsa_242 | D02008 |
| 2 | hsa_242 | D02068 |
| 2 | hsa_242 | D03753 |
| 2 | hsa_242 | D03773 |
| 2 | hsa_246 | D00187 |
| 2 | hsa_246 | D00325 |
| 2 | hsa_246 | D00333 |
| 2 | hsa_246 | D00377 |
| 2 | hsa_246 | D03722 |
| 2 | hsa_246 | D03752 |
| 2 | hsa_247 | D00094 |
| 2 | hsa_247 | D00208 |
| 2 | hsa_247 | D00371 |
| 2 | hsa_247 | D00496 |
| 2 | hsa_247 | D00528 |
| 2 | hsa_247 | D00537 |
| 2 | hsa_247 | D00703 |
| 2 | hsa_247 | D00753 |
| 2 | hsa_247 | D01264 |
| 2 | hsa_247 | D01346 |
| 2 | hsa_247 | D01665 |
| 2 | hsa_247 | D01885 |
| 2 | hsa_247 | D03781 |
| 2 | hsa_2534 | D00270 |
| 2 | hsa_2534 | D00535 |
| 2 | hsa_2534 | D00900 |
| 2 | hsa_2534 | D02579 |
| 2 | hsa_2534 | D03689 |
| 2 | hsa_2534 | D03826 |
| 2 | hsa_2534 | D05458 |
| 2 | hsa_2548 | D00002 |
| 2 | hsa_2548 | D00391 |
| 2 | hsa_2548 | D00885 |
| 2 | hsa_2548 | D02193 |
| 2 | hsa_2548 | D02556 |
| 2 | hsa_2548 | D03714 |
| 2 | hsa_25796 | D00050 |
| 2 | hsa_25796 | D00097 |
| 2 | hsa_25796 | D00217 |
| 2 | hsa_25796 | D00882 |
| 2 | hsa_25796 | D00960 |
| 2 | hsa_25796 | D03350 |
| 2 | hsa_25796 | D03734 |
| 2 | hsa_25796 | D03756 |
| 2 | hsa_25796 | D03778 |
| 2 | hsa_25824 | D00005 |
| 2 | hsa_25824 | D00300 |
| 2 | hsa_25824 | D00452 |
| 2 | hsa_25824 | D00596 |
| 2 | hsa_25824 | D00805 |
| 2 | hsa_25824 | D00969 |
| 2 | hsa_25824 | D01968 |
| 2 | hsa_25824 | D02258 |
| 2 | hsa_25824 | D02308 |
| 2 | hsa_25824 | D03720 |
| 2 | hsa_2582 | D00005 |
| 2 | hsa_2582 | D00125 |
| 2 | hsa_2582 | D00536 |
| 2 | hsa_2582 | D00624 |
| 2 | hsa_2582 | D00786 |
| 2 | hsa_2582 | D00900 |
| 2 | hsa_2582 | D01264 |
| 2 | hsa_2582 | D02335 |
| 2 | hsa_2582 | D02729 |
| 2 | hsa_2582 | D03798 |
| 2 | hsa_2595 | D00463 |
| 2 | hsa_2595 | D00654 |
| 2 | hsa_2595 | D00726 |
| 2 | hsa_2595 | D00965 |
| 2 | hsa_2595 | D01097 |
| 2 | hsa_2595 | D02835 |
| 2 | hsa_2595 | D03440 |
| 2 | hsa_2595 | D03689 |
| 2 | hsa_2595 | D03828 |
| 2 | hsa_2597 | D00118 |
| 2 | hsa_2597 | D00160 |
| 2 | hsa_2597 | D00234 |
| 2 | hsa_2597 | D00359 |
| 2 | hsa_2597 | D00625 |
| 2 | hsa_2597 | D01061 |
| 2 | hsa_2597 | D02323 |
| 2 | hsa_2597 | D02566 |
| 2 | hsa_2597 | D02581 |
| 2 | hsa_2597 | D03788 |
| 2 | hsa_25 | D00145 |
| 2 | hsa_25 | D00332 |
| 2 | hsa_25 | D00434 |
| 2 | hsa_25 | D00513 |
| 2 | hsa_25 | D02258 |
| 2 | hsa_25 | D02375 |
| 2 | hsa_25 | D04031 |
| 2 | hsa_25 | D05458 |
| 2 | hsa_260293 | D00045 |
| 2 | hsa_260293 | D00054 |
| 2 | hsa_260293 | D00155 |
| 2 | hsa_260293 | D00160 |
| 2 | hsa_260293 | D01765 |
| 2 | hsa_260293 | D01900 |
| 2 | hsa_26279 | D00055 |
| 2 | hsa_26279 | D00231 |
| 2 | hsa_26279 | D00621 |
| 2 | hsa_26279 | D00655 |
| 2 | hsa_26279 | D01180 |
| 2 | hsa_26279 | D01196 |
| 2 | hsa_26279 | D01264 |
| 2 | hsa_26279 | D01828 |
| 2 | hsa_26279 | D02321 |
| 2 | hsa_26279 | D03218 |
| 2 | hsa_26279 | D03734 |
| 2 | hsa_26279 | D04966 |
| 2 | hsa_2638 | D00132 |
| 2 | hsa_2638 | D00216 |
| 2 | hsa_2638 | D01704 |
| 2 | hsa_2638 | D02289 |
| 2 | hsa_2638 | D03826 |
| 2 | hsa_2639 | D00126 |
| 2 | hsa_2639 | D00127 |
| 2 | hsa_2639 | D00279 |
| 2 | hsa_2639 | D00568 |
| 2 | hsa_2639 | D00892 |
| 2 | hsa_2639 | D02671 |
| 2 | hsa_2639 | D03756 |
| 2 | hsa_2639 | D04983 |
| 2 | hsa_2639 | D05407 |
| 2 | hsa_2673 | D00279 |
| 2 | hsa_2673 | D00813 |
| 2 | hsa_2673 | D01900 |
| 2 | hsa_2673 | D02356 |
| 2 | hsa_2673 | D02731 |
| 2 | hsa_2673 | D03769 |
| 2 | hsa_2677 | D00041 |
| 2 | hsa_2677 | D00342 |
| 2 | hsa_2677 | D00359 |
| 2 | hsa_2677 | D02323 |
| 2 | hsa_2677 | D03710 |
| 2 | hsa_27032 | D00227 |
| 2 | hsa_27032 | D00451 |
| 2 | hsa_27032 | D00494 |
| 2 | hsa_27032 | D00510 |
| 2 | hsa_27032 | D00562 |
| 2 | hsa_27032 | D00703 |
| 2 | hsa_27032 | D02042 |
| 2 | hsa_27034 | D00043 |
| 2 | hsa_27034 | D00054 |
| 2 | hsa_27034 | D00437 |
| 2 | hsa_27034 | D00533 |
| 2 | hsa_27034 | D01346 |
| 2 | hsa_27034 | D01513 |
| 2 | hsa_27034 | D01844 |
| 2 | hsa_27034 | D01866 |
| 2 | hsa_27034 | D02731 |
| 2 | hsa_27034 | D03735 |
| 2 | hsa_27034 | D03882 |
| 2 | hsa_270 | D00342 |
| 2 | hsa_270 | D00391 |
| 2 | hsa_270 | D00544 |
| 2 | hsa_270 | D00810 |
| 2 | hsa_270 | D01211 |
| 2 | hsa_270 | D02229 |
| 2 | hsa_270 | D05458 |
| 2 | hsa_27115 | D00332 |
| 2 | hsa_27115 | D00416 |
| 2 | hsa_27115 | D00512 |
| 2 | hsa_2746 | D00035 |
| 2 | hsa_2746 | D00217 |
| 2 | hsa_2746 | D00364 |
| 2 | hsa_2746 | D00503 |
| 2 | hsa_2746 | D00516 |
| 2 | hsa_2746 | D00530 |
| 2 | hsa_2746 | D00624 |
| 2 | hsa_2746 | D00651 |
| 2 | hsa_2746 | D01118 |
| 2 | hsa_2746 | D01122 |
| 2 | hsa_2746 | D01885 |
| 2 | hsa_2746 | D01900 |
| 2 | hsa_2746 | D01915 |
| 2 | hsa_2746 | D03735 |
| 2 | hsa_2766 | D00342 |
| 2 | hsa_2766 | D00501 |
| 2 | hsa_2766 | D00568 |
| 2 | hsa_2766 | D00651 |
| 2 | hsa_2766 | D00670 |
| 2 | hsa_2766 | D00947 |
| 2 | hsa_2766 | D01276 |
| 2 | hsa_2766 | D01974 |
| 2 | hsa_2766 | D02008 |
| 2 | hsa_2766 | D02561 |
| 2 | hsa_2766 | D03115 |
| 2 | hsa_2766 | D03760 |
| 2 | hsa_279 | D00055 |
| 2 | hsa_279 | D00545 |
| 2 | hsa_279 | D00549 |
| 2 | hsa_279 | D00568 |
| 2 | hsa_279 | D00771 |
| 2 | hsa_279 | D00805 |
| 2 | hsa_279 | D00889 |
| 2 | hsa_279 | D01709 |
| 2 | hsa_279 | D02110 |
| 2 | hsa_279 | D02304 |
| 2 | hsa_279 | D02580 |
| 2 | hsa_2806 | D00369 |
| 2 | hsa_2806 | D00398 |
| 2 | hsa_2806 | D00568 |
| 2 | hsa_2806 | D00569 |
| 2 | hsa_2806 | D01911 |
| 2 | hsa_2806 | D02110 |
| 2 | hsa_2806 | D02321 |
| 2 | hsa_2806 | D04966 |
| 2 | hsa_28227 | D00328 |
| 2 | hsa_28227 | D00421 |
| 2 | hsa_28227 | D00494 |
| 2 | hsa_28227 | D01264 |
| 2 | hsa_2822 | D00186 |
| 2 | hsa_2822 | D00328 |
| 2 | hsa_2822 | D00364 |
| 2 | hsa_2822 | D00550 |
| 2 | hsa_2822 | D00593 |
| 2 | hsa_2822 | D00650 |
| 2 | hsa_2822 | D00947 |
| 2 | hsa_2822 | D00963 |
| 2 | hsa_2822 | D00994 |
| 2 | hsa_2822 | D01432 |
| 2 | hsa_2822 | D01513 |
| 2 | hsa_2822 | D01582 |
| 2 | hsa_2822 | D01984 |
| 2 | hsa_2822 | D02564 |
| 2 | hsa_2822 | D03756 |
| 2 | hsa_284541 | D00037 |
| 2 | hsa_284541 | D00315 |
| 2 | hsa_284541 | D00503 |
| 2 | hsa_284541 | D00566 |
| 2 | hsa_284541 | D00624 |
| 2 | hsa_284541 | D00753 |
| 2 | hsa_284541 | D01346 |
| 2 | hsa_284541 | D01811 |
| 2 | hsa_284541 | D01825 |
| 2 | hsa_284541 | D01828 |
| 2 | hsa_284541 | D03798 |
| 2 | hsa_285220 | D00222 |
| 2 | hsa_285220 | D00417 |
| 2 | hsa_285220 | D00418 |
| 2 | hsa_285220 | D00487 |
| 2 | hsa_285220 | D00596 |
| 2 | hsa_285220 | D00752 |
| 2 | hsa_285220 | D01582 |
| 2 | hsa_285220 | D01866 |
| 2 | hsa_285220 | D03601 |
| 2 | hsa_285220 | D05458 |
| 2 | hsa_2880 | D00039 |
| 2 | hsa_2880 | D00153 |
| 2 | hsa_2880 | D00186 |
| 2 | hsa_2880 | D00188 |
| 2 | hsa_2880 | D00217 |
| 2 | hsa_2880 | D00322 |
| 2 | hsa_2880 | D00771 |
| 2 | hsa_2880 | D01667 |
| 2 | hsa_2880 | D01765 |
| 2 | hsa_2880 | D03752 |
| 2 | hsa_2882 | D00970 |
| 2 | hsa_2882 | D01196 |
| 2 | hsa_2882 | D01565 |
| 2 | hsa_2882 | D01915 |
| 2 | hsa_2882 | D02017 |
| 2 | hsa_2882 | D03077 |
| 2 | hsa_2882 | D03760 |
| 2 | hsa_28972 | D00153 |
| 2 | hsa_28972 | D00658 |
| 2 | hsa_28972 | D02008 |
| 2 | hsa_28972 | D02563 |
| 2 | hsa_28972 | D03735 |
| 2 | hsa_28972 | D03899 |
| 2 | hsa_28 | D00005 |
| 2 | hsa_28 | D00007 |
| 2 | hsa_28 | D00330 |
| 2 | hsa_28 | D00414 |
| 2 | hsa_28 | D00423 |
| 2 | hsa_28 | D00536 |
| 2 | hsa_28 | D00893 |
| 2 | hsa_28 | D00963 |
| 2 | hsa_28 | D01064 |
| 2 | hsa_28 | D03034 |
| 2 | hsa_2936 | D00032 |
| 2 | hsa_2936 | D00622 |
| 2 | hsa_2936 | D01578 |
| 2 | hsa_2936 | D01690 |
| 2 | hsa_2936 | D02068 |
| 2 | hsa_2936 | D02335 |
| 2 | hsa_2936 | D02566 |
| 2 | hsa_2936 | D02729 |
| 2 | hsa_2950 | D00045 |
| 2 | hsa_2950 | D00519 |
| 2 | hsa_2950 | D00709 |
| 2 | hsa_2950 | D01097 |
| 2 | hsa_2950 | D02335 |
| 2 | hsa_2950 | D02350 |
| 2 | hsa_2950 | D03741 |
| 2 | hsa_2950 | D03778 |
| 2 | hsa_29785 | D00065 |
| 2 | hsa_29785 | D00224 |
| 2 | hsa_29785 | D00892 |
| 2 | hsa_29785 | D01275 |
| 2 | hsa_29785 | D02017 |
| 2 | hsa_29785 | D02176 |
| 2 | hsa_29785 | D02193 |
| 2 | hsa_29785 | D02655 |
| 2 | hsa_29785 | D03712 |
| 2 | hsa_29785 | D03753 |
| 2 | hsa_29920 | D00183 |
| 2 | hsa_29920 | D00513 |
| 2 | hsa_29920 | D00521 |
| 2 | hsa_29920 | D00545 |
| 2 | hsa_29920 | D01228 |
| 2 | hsa_29920 | D01475 |
| 2 | hsa_29920 | D01966 |
| 2 | hsa_29920 | D03731 |
| 2 | hsa_29941 | D00333 |
| 2 | hsa_29941 | D00380 |
| 2 | hsa_29941 | D00383 |
| 2 | hsa_29941 | D00733 |
| 2 | hsa_29941 | D00969 |
| 2 | hsa_29968 | D00219 |
| 2 | hsa_29968 | D00252 |
| 2 | hsa_29968 | D00380 |
| 2 | hsa_29968 | D00510 |
| 2 | hsa_3001 | D00126 |
| 2 | hsa_3001 | D00569 |
| 2 | hsa_3001 | D00667 |
| 2 | hsa_3001 | D02729 |
| 2 | hsa_3002 | D00153 |
| 2 | hsa_3002 | D00421 |
| 2 | hsa_3002 | D00496 |
| 2 | hsa_3002 | D01164 |
| 2 | hsa_3002 | D01811 |
| 2 | hsa_3002 | D02580 |
| 2 | hsa_3002 | D02709 |
| 2 | hsa_3002 | D03803 |
| 2 | hsa_3028 | D00562 |
| 2 | hsa_3028 | D01346 |
| 2 | hsa_3028 | D02289 |
| 2 | hsa_3028 | D02290 |
| 2 | hsa_3028 | D03775 |
| 2 | hsa_3033 | D00132 |
| 2 | hsa_3033 | D00216 |
| 2 | hsa_3033 | D00549 |
| 2 | hsa_3033 | D00753 |
| 2 | hsa_3033 | D03601 |
| 2 | hsa_3033 | D03716 |
| 2 | hsa_3034 | D00054 |
| 2 | hsa_3034 | D00118 |
| 2 | hsa_3034 | D00216 |
| 2 | hsa_3034 | D00294 |
| 2 | hsa_3034 | D00547 |
| 2 | hsa_3034 | D00579 |
| 2 | hsa_3034 | D00752 |
| 2 | hsa_3034 | D00964 |
| 2 | hsa_3034 | D01275 |
| 2 | hsa_3034 | D01900 |
| 2 | hsa_3034 | D02017 |
| 2 | hsa_3034 | D03712 |
| 2 | hsa_3034 | D05341 |
| 2 | hsa_3035 | D00510 |
| 2 | hsa_3035 | D00512 |
| 2 | hsa_3035 | D00533 |
| 2 | hsa_3035 | D00566 |
| 2 | hsa_3035 | D00902 |
| 2 | hsa_3035 | D01578 |
| 2 | hsa_3035 | D03714 |
| 2 | hsa_3055 | D00153 |
| 2 | hsa_3055 | D00188 |
| 2 | hsa_3055 | D00394 |
| 2 | hsa_3055 | D00425 |
| 2 | hsa_3055 | D00536 |
| 2 | hsa_3055 | D00563 |
| 2 | hsa_3055 | D00574 |
| 2 | hsa_3055 | D01885 |
| 2 | hsa_3055 | D02580 |
| 2 | hsa_3055 | D03733 |
| 2 | hsa_3067 | D00300 |
| 2 | hsa_3067 | D00416 |
| 2 | hsa_3067 | D00537 |
| 2 | hsa_3067 | D00538 |
| 2 | hsa_3067 | D00574 |
| 2 | hsa_3067 | D01973 |
| 2 | hsa_3067 | D02835 |
| 2 | hsa_306 | D00065 |
| 2 | hsa_306 | D00097 |
| 2 | hsa_306 | D00142 |
| 2 | hsa_306 | D00410 |
| 2 | hsa_306 | D00434 |
| 2 | hsa_306 | D00494 |
| 2 | hsa_306 | D00533 |
| 2 | hsa_306 | D00546 |
| 2 | hsa_306 | D00621 |
| 2 | hsa_306 | D03736 |
| 2 | hsa_306 | D03778 |
| 2 | hsa_306 | D03828 |
| 2 | hsa_306 | D04197 |
| 2 | hsa_30814 | D00231 |
| 2 | hsa_30814 | D00414 |
| 2 | hsa_30814 | D00569 |
| 2 | hsa_30814 | D00968 |
| 2 | hsa_30814 | D00969 |
| 2 | hsa_30814 | D03769 |
| 2 | hsa_30814 | D04028 |
| 2 | hsa_30833 | D01332 |
| 2 | hsa_30 | D00094 |
| 2 | hsa_30 | D00158 |
| 2 | hsa_30 | D00332 |
| 2 | hsa_30 | D00434 |
| 2 | hsa_30 | D00568 |
| 2 | hsa_30 | D00621 |
| 2 | hsa_30 | D01064 |
| 2 | hsa_30 | D01133 |
| 2 | hsa_30 | D01712 |
| 2 | hsa_30 | D01765 |
| 2 | hsa_30 | D01915 |
| 2 | hsa_30 | D02321 |
| 2 | hsa_313 | D00125 |
| 2 | hsa_313 | D00364 |
| 2 | hsa_313 | D00377 |
| 2 | hsa_313 | D01136 |
| 2 | hsa_313 | D01370 |
| 2 | hsa_313 | D01688 |
| 2 | hsa_313 | D01715 |
| 2 | hsa_313 | D01974 |
| 2 | hsa_313 | D02441 |
| 2 | hsa_313 | D05407 |
| 2 | hsa_3141 | D00018 |
| 2 | hsa_3141 | D00052 |
| 2 | hsa_3141 | D00196 |
| 2 | hsa_3141 | D00252 |
| 2 | hsa_3141 | D00322 |
| 2 | hsa_3141 | D01097 |
| 2 | hsa_3141 | D01180 |
| 2 | hsa_3141 | D01840 |
| 2 | hsa_3141 | D03012 |
| 2 | hsa_3141 | D03643 |
| 2 | hsa_3156 | D00513 |
| 2 | hsa_3156 | D00667 |
| 2 | hsa_3156 | D00813 |
| 2 | hsa_3156 | D03751 |
| 2 | hsa_31 | D00035 |
| 2 | hsa_31 | D00125 |
| 2 | hsa_31 | D00542 |
| 2 | hsa_31 | D00574 |
| 2 | hsa_31 | D01198 |
| 2 | hsa_31 | D01842 |
| 2 | hsa_31 | D02341 |
| 2 | hsa_326625 | D00414 |
| 2 | hsa_326625 | D00496 |
| 2 | hsa_326625 | D00771 |
| 2 | hsa_326625 | D00885 |
| 2 | hsa_326625 | D01164 |
| 2 | hsa_326625 | D01688 |
| 2 | hsa_326625 | D02194 |
| 2 | hsa_326625 | D03728 |
| 2 | hsa_327 | D00139 |
| 2 | hsa_327 | D00452 |
| 2 | hsa_327 | D00544 |
| 2 | hsa_327 | D00884 |
| 2 | hsa_327 | D00995 |
| 2 | hsa_327 | D01844 |
| 2 | hsa_327 | D02487 |
| 2 | hsa_327 | D02558 |
| 2 | hsa_327 | D02563 |
| 2 | hsa_3283 | D00549 |
| 2 | hsa_3283 | D00569 |
| 2 | hsa_3283 | D00960 |
| 2 | hsa_3283 | D01118 |
| 2 | hsa_3283 | D01715 |
| 2 | hsa_3283 | D02561 |
| 2 | hsa_3283 | D03077 |
| 2 | hsa_3283 | D03798 |
| 2 | hsa_3290 | D00038 |
| 2 | hsa_3290 | D00103 |
| 2 | hsa_3290 | D00118 |
| 2 | hsa_3290 | D00417 |
| 2 | hsa_3290 | D00521 |
| 2 | hsa_3290 | D00528 |
| 2 | hsa_3290 | D00535 |
| 2 | hsa_3290 | D00547 |
| 2 | hsa_3290 | D02214 |
| 2 | hsa_3290 | D02556 |
| 2 | hsa_3290 | D04029 |
| 2 | hsa_3291 | D00027 |
| 2 | hsa_3291 | D00043 |
| 2 | hsa_3291 | D00052 |
| 2 | hsa_3291 | D00103 |
| 2 | hsa_3291 | D00418 |
| 2 | hsa_3291 | D00515 |
| 2 | hsa_3291 | D00752 |
| 2 | hsa_3291 | D00969 |
| 2 | hsa_3291 | D01565 |
| 2 | hsa_3291 | D02289 |
| 2 | hsa_3292 | D00035 |
| 2 | hsa_3292 | D00049 |
| 2 | hsa_3292 | D00369 |
| 2 | hsa_3292 | D00475 |
| 2 | hsa_3292 | D00515 |
| 2 | hsa_3292 | D00538 |
| 2 | hsa_3292 | D01397 |
| 2 | hsa_3292 | D01888 |
| 2 | hsa_3292 | D02068 |
| 2 | hsa_3292 | D02729 |
| 2 | hsa_3292 | D03728 |
| 2 | hsa_3292 | D03806 |
| 2 | hsa_3295 | D00387 |
| 2 | hsa_3295 | D00762 |
| 2 | hsa_3295 | D00882 |
| 2 | hsa_3295 | D01061 |
| 2 | hsa_3295 | D01211 |
| 2 | hsa_3295 | D01565 |
| 2 | hsa_3295 | D01888 |
| 2 | hsa_3295 | D02581 |
| 2 | hsa_32 | D00418 |
| 2 | hsa_32 | D00658 |
| 2 | hsa_32 | D00970 |
| 2 | hsa_32 | D01862 |
| 2 | hsa_32 | D01866 |
| 2 | hsa_32 | D02008 |
| 2 | hsa_32 | D02289 |
| 2 | hsa_32 | D02769 |
| 2 | hsa_32 | D03767 |
| 2 | hsa_3376 | D00449 |
| 2 | hsa_3376 | D00947 |
| 2 | hsa_3376 | D01862 |
| 2 | hsa_3376 | D02564 |
| 2 | hsa_3376 | D02709 |
| 2 | hsa_3376 | D03776 |
| 2 | hsa_3376 | D03806 |
| 2 | hsa_339221 | D00132 |
| 2 | hsa_339221 | D00251 |
| 2 | hsa_339221 | D00421 |
| 2 | hsa_339221 | D01061 |
| 2 | hsa_339221 | D01275 |
| 2 | hsa_339221 | D01862 |
| 2 | hsa_339221 | D03720 |
| 2 | hsa_3416 | D00574 |
| 2 | hsa_3416 | D00965 |
| 2 | hsa_3416 | D02698 |
| 2 | hsa_3480 | D00437 |
| 2 | hsa_3480 | D00494 |
| 2 | hsa_3480 | D01885 |
| 2 | hsa_3480 | D02008 |
| 2 | hsa_3480 | D02308 |
| 2 | hsa_3480 | D03735 |
| 2 | hsa_3480 | D03823 |
| 2 | hsa_349565 | D00131 |
| 2 | hsa_349565 | D00188 |
| 2 | hsa_349565 | D00535 |
| 2 | hsa_349565 | D00781 |
| 2 | hsa_349565 | D00968 |
| 2 | hsa_349565 | D00970 |
| 2 | hsa_349565 | D02835 |
| 2 | hsa_349565 | D03773 |
| 2 | hsa_34 | D00132 |
| 2 | hsa_34 | D01364 |
| 2 | hsa_34 | D01718 |
| 2 | hsa_34 | D01888 |
| 2 | hsa_34 | D02328 |
| 2 | hsa_34 | D03767 |
| 2 | hsa_34 | D03772 |
| 2 | hsa_353 | D00155 |
| 2 | hsa_353 | D00234 |
| 2 | hsa_353 | D00342 |
| 2 | hsa_353 | D00656 |
| 2 | hsa_353 | D00753 |
| 2 | hsa_353 | D01840 |
| 2 | hsa_353 | D01915 |
| 2 | hsa_353 | D03769 |
| 2 | hsa_354 | D00070 |
| 2 | hsa_354 | D00454 |
| 2 | hsa_354 | D01119 |
| 2 | hsa_354 | D01332 |
| 2 | hsa_354 | D02323 |
| 2 | hsa_354 | D02835 |
| 2 | hsa_354 | D03115 |
| 2 | hsa_3551 | D00827 |
| 2 | hsa_3551 | D01582 |
| 2 | hsa_3551 | D01918 |
| 2 | hsa_3551 | D02560 |
| 2 | hsa_3551 | D03829 |
| 2 | hsa_35 | D00153 |
| 2 | hsa_35 | D00218 |
| 2 | hsa_35 | D00434 |
| 2 | hsa_35 | D01223 |
| 2 | hsa_35 | D01907 |
| 2 | hsa_35 | D02356 |
| 2 | hsa_3612 | D00217 |
| 2 | hsa_3612 | D00494 |
| 2 | hsa_3612 | D00622 |
| 2 | hsa_3612 | D00998 |
| 2 | hsa_3612 | D01325 |
| 2 | hsa_3612 | D02375 |
| 2 | hsa_3612 | D03115 |
| 2 | hsa_3612 | D03776 |
| 2 | hsa_3614 | D00222 |
| 2 | hsa_3614 | D00503 |
| 2 | hsa_3614 | D00568 |
| 2 | hsa_3614 | D00579 |
| 2 | hsa_3614 | D01888 |
| 2 | hsa_3614 | D02335 |
| 2 | hsa_3614 | D05407 |
| 2 | hsa_3615 | D00421 |
| 2 | hsa_3615 | D00562 |
| 2 | hsa_3615 | D00620 |
| 2 | hsa_3615 | D01183 |
| 2 | hsa_3615 | D01704 |
| 2 | hsa_3615 | D03743 |
| 2 | hsa_3615 | D03775 |
| 2 | hsa_3615 | D04028 |
| 2 | hsa_3643 | D00362 |
| 2 | hsa_3643 | D00369 |
| 2 | hsa_3643 | D00449 |
| 2 | hsa_3643 | D00547 |
| 2 | hsa_3643 | D00567 |
| 2 | hsa_3643 | D00969 |
| 2 | hsa_3643 | D06238 |
| 2 | hsa_3645 | D03440 |
| 2 | hsa_3645 | D03689 |
| 2 | hsa_3645 | D03823 |
| 2 | hsa_36 | D00359 |
| 2 | hsa_36 | D00487 |
| 2 | hsa_36 | D00528 |
| 2 | hsa_36 | D00650 |
| 2 | hsa_36 | D00652 |
| 2 | hsa_36 | D01888 |
| 2 | hsa_36 | D02559 |
| 2 | hsa_36 | D03012 |
| 2 | hsa_36 | D03712 |
| 2 | hsa_36 | D03805 |
| 2 | hsa_3702 | D00014 |
| 2 | hsa_3702 | D00029 |
| 2 | hsa_3702 | D00142 |
| 2 | hsa_3702 | D00947 |
| 2 | hsa_3702 | D03778 |
| 2 | hsa_3702 | D03781 |
| 2 | hsa_3712 | D00158 |
| 2 | hsa_3712 | D00274 |
| 2 | hsa_3712 | D00294 |
| 2 | hsa_3712 | D01425 |
| 2 | hsa_3712 | D01513 |
| 2 | hsa_3712 | D03738 |
| 2 | hsa_3712 | D03758 |
| 2 | hsa_3716 | D00021 |
| 2 | hsa_3716 | D00488 |
| 2 | hsa_3716 | D01118 |
| 2 | hsa_3716 | D01223 |
| 2 | hsa_3716 | D01900 |
| 2 | hsa_3716 | D02168 |
| 2 | hsa_3717 | D00169 |
| 2 | hsa_3717 | D00380 |
| 2 | hsa_3717 | D00652 |
| 2 | hsa_3717 | D00752 |
| 2 | hsa_3717 | D01513 |
| 2 | hsa_3717 | D01828 |
| 2 | hsa_3717 | D03826 |
| 2 | hsa_3717 | D05341 |
| 2 | hsa_3718 | D00148 |
| 2 | hsa_3718 | D00332 |
| 2 | hsa_3718 | D00391 |
| 2 | hsa_3718 | D00416 |
| 2 | hsa_3718 | D00965 |
| 2 | hsa_3718 | D03720 |
| 2 | hsa_3718 | D03767 |
| 2 | hsa_3735 | D00670 |
| 2 | hsa_3735 | D01767 |
| 2 | hsa_3735 | D01866 |
| 2 | hsa_3735 | D02566 |
| 2 | hsa_3735 | D03601 |
| 2 | hsa_3735 | D03733 |
| 2 | hsa_3735 | D03798 |
| 2 | hsa_377677 | D00035 |
| 2 | hsa_377677 | D00118 |
| 2 | hsa_377677 | D00125 |
| 2 | hsa_377677 | D00224 |
| 2 | hsa_377677 | D00285 |
| 2 | hsa_377677 | D00625 |
| 2 | hsa_377677 | D00904 |
| 2 | hsa_377677 | D01667 |
| 2 | hsa_377677 | D01712 |
| 2 | hsa_377677 | D02258 |
| 2 | hsa_377677 | D02671 |
| 2 | hsa_3791 | D00027 |
| 2 | hsa_3791 | D00300 |
| 2 | hsa_3791 | D00330 |
| 2 | hsa_3791 | D00546 |
| 2 | hsa_3791 | D00947 |
| 2 | hsa_3791 | D01844 |
| 2 | hsa_3791 | D01888 |
| 2 | hsa_3791 | D02304 |
| 2 | hsa_3791 | D02355 |
| 2 | hsa_3791 | D03806 |
| 2 | hsa_3791 | D03826 |
| 2 | hsa_3791 | D05458 |
| 2 | hsa_3815 | D00188 |
| 2 | hsa_3815 | D00503 |
| 2 | hsa_3815 | D03738 |
| 2 | hsa_3815 | D03753 |
| 2 | hsa_3815 | D03826 |
| 2 | hsa_3816 | D00887 |
| 2 | hsa_3816 | D01907 |
| 2 | hsa_3816 | D02356 |
| 2 | hsa_3816 | D02418 |
| 2 | hsa_3817 | D00039 |
| 2 | hsa_3817 | D00155 |
| 2 | hsa_3817 | D00283 |
| 2 | hsa_3817 | D00340 |
| 2 | hsa_3817 | D00359 |
| 2 | hsa_3817 | D00452 |
| 2 | hsa_3817 | D01180 |
| 2 | hsa_3817 | D01900 |
| 2 | hsa_3817 | D01911 |
| 2 | hsa_3817 | D02558 |
| 2 | hsa_3817 | D03710 |
| 2 | hsa_3817 | D03743 |
| 2 | hsa_3818 | D00027 |
| 2 | hsa_3818 | D00224 |
| 2 | hsa_3818 | D00387 |
| 2 | hsa_3818 | D00487 |
| 2 | hsa_3818 | D00494 |
| 2 | hsa_3818 | D00510 |
| 2 | hsa_3818 | D00530 |
| 2 | hsa_3818 | D00568 |
| 2 | hsa_3818 | D00621 |
| 2 | hsa_3818 | D00968 |
| 2 | hsa_3818 | D01346 |
| 2 | hsa_3818 | D01977 |
| 2 | hsa_3818 | D03806 |
| 2 | hsa_3818 | D04031 |
| 2 | hsa_38 | D00227 |
| 2 | hsa_38 | D01709 |
| 2 | hsa_38 | D01715 |
| 2 | hsa_38 | D02558 |
| 2 | hsa_38 | D02731 |
| 2 | hsa_3906 | D00043 |
| 2 | hsa_3906 | D00049 |
| 2 | hsa_3906 | D00120 |
| 2 | hsa_3906 | D00130 |
| 2 | hsa_3906 | D00153 |
| 2 | hsa_3906 | D00416 |
| 2 | hsa_3906 | D00505 |
| 2 | hsa_3906 | D00903 |
| 2 | hsa_3906 | D01840 |
| 2 | hsa_3906 | D03440 |
| 2 | hsa_3906 | D03753 |
| 2 | hsa_3906 | D04024 |
| 2 | hsa_390956 | D00786 |
| 2 | hsa_390956 | D01122 |
| 2 | hsa_390956 | D01397 |
| 2 | hsa_390956 | D02168 |
| 2 | hsa_390956 | D03218 |
| 2 | hsa_390956 | D03601 |
| 2 | hsa_390956 | D03735 |
| 2 | hsa_3932 | D00185 |
| 2 | hsa_3932 | D00566 |
| 2 | hsa_3932 | D00653 |
| 2 | hsa_3932 | D00656 |
| 2 | hsa_3932 | D00884 |
| 2 | hsa_3932 | D01765 |
| 2 | hsa_3932 | D03710 |
| 2 | hsa_3939 | D00994 |
| 2 | hsa_3939 | D01918 |
| 2 | hsa_3939 | D02017 |
| 2 | hsa_3939 | D02709 |
| 2 | hsa_3945 | D00041 |
| 2 | hsa_3945 | D00579 |
| 2 | hsa_3945 | D01966 |
| 2 | hsa_3945 | D02418 |
| 2 | hsa_3988 | D00141 |
| 2 | hsa_3988 | D00377 |
| 2 | hsa_3988 | D00394 |
| 2 | hsa_3988 | D00487 |
| 2 | hsa_3988 | D00584 |
| 2 | hsa_3988 | D01001 |
| 2 | hsa_3988 | D01133 |
| 2 | hsa_3988 | D01704 |
| 2 | hsa_3988 | D01888 |
| 2 | hsa_3988 | D02166 |
| 2 | hsa_3988 | D02267 |
| 2 | hsa_3988 | D04029 |
| 2 | hsa_3988 | D04966 |
| 2 | hsa_3990 | D00014 |
| 2 | hsa_3990 | D00037 |
| 2 | hsa_3990 | D00070 |
| 2 | hsa_3990 | D00186 |
| 2 | hsa_3990 | D00398 |
| 2 | hsa_3990 | D00569 |
| 2 | hsa_3990 | D00947 |
| 2 | hsa_3990 | D01704 |
| 2 | hsa_3990 | D02561 |
| 2 | hsa_3990 | D02566 |
| 2 | hsa_3990 | D03440 |
| 2 | hsa_3990 | D03735 |
| 2 | hsa_3990 | D03741 |
| 2 | hsa_3990 | D03751 |
| 2 | hsa_3990 | D03765 |
| 2 | hsa_3990 | D03805 |
| 2 | hsa_3991 | D00118 |
| 2 | hsa_3991 | D00315 |
| 2 | hsa_3991 | D00328 |
| 2 | hsa_3991 | D00437 |
| 2 | hsa_3991 | D00494 |
| 2 | hsa_3991 | D00515 |
| 2 | hsa_3991 | D00544 |
| 2 | hsa_3991 | D02579 |
| 2 | hsa_3991 | D03756 |
| 2 | hsa_4017 | D00227 |
| 2 | hsa_4017 | D00252 |
| 2 | hsa_4017 | D00325 |
| 2 | hsa_4017 | D01325 |
| 2 | hsa_4017 | D01811 |
| 2 | hsa_4017 | D02289 |
| 2 | hsa_4017 | D04029 |
| 2 | hsa_4023 | D00328 |
| 2 | hsa_4023 | D00463 |
| 2 | hsa_4023 | D00577 |
| 2 | hsa_4023 | D01582 |
| 2 | hsa_4023 | D02166 |
| 2 | hsa_4023 | D02308 |
| 2 | hsa_4023 | D04024 |
| 2 | hsa_4025 | D00107 |
| 2 | hsa_4025 | D00187 |
| 2 | hsa_4025 | D00203 |
| 2 | hsa_4025 | D00340 |
| 2 | hsa_4025 | D00425 |
| 2 | hsa_4025 | D00449 |
| 2 | hsa_4025 | D01712 |
| 2 | hsa_4025 | D02315 |
| 2 | hsa_4025 | D03218 |
| 2 | hsa_4048 | D00036 |
| 2 | hsa_4048 | D00383 |
| 2 | hsa_4048 | D00625 |
| 2 | hsa_4048 | D00753 |
| 2 | hsa_4048 | D00947 |
| 2 | hsa_4048 | D00969 |
| 2 | hsa_4048 | D02698 |
| 2 | hsa_4051 | D00018 |
| 2 | hsa_4051 | D00283 |
| 2 | hsa_4051 | D00298 |
| 2 | hsa_4051 | D00322 |
| 2 | hsa_4051 | D00781 |
| 2 | hsa_4051 | D02194 |
| 2 | hsa_4051 | D02341 |
| 2 | hsa_4051 | D03689 |
| 2 | hsa_4051 | D03823 |
| 2 | hsa_4051 | D04292 |
| 2 | hsa_4051 | D04966 |
| 2 | hsa_4058 | D00029 |
| 2 | hsa_4058 | D00136 |
| 2 | hsa_4058 | D00234 |
| 2 | hsa_4058 | D00315 |
| 2 | hsa_4058 | D00519 |
| 2 | hsa_4058 | D00542 |
| 2 | hsa_4058 | D01844 |
| 2 | hsa_4058 | D01866 |
| 2 | hsa_4058 | D01907 |
| 2 | hsa_4058 | D01918 |
| 2 | hsa_4058 | D02451 |
| 2 | hsa_4058 | D02581 |
| 2 | hsa_4058 | D02756 |
| 2 | hsa_4058 | D03758 |
| 2 | hsa_4058 | D04031 |
| 2 | hsa_4067 | D00153 |
| 2 | hsa_4067 | D00208 |
| 2 | hsa_4067 | D00231 |
| 2 | hsa_4067 | D00234 |
| 2 | hsa_4067 | D00418 |
| 2 | hsa_4067 | D00538 |
| 2 | hsa_4067 | D00630 |
| 2 | hsa_4067 | D00829 |
| 2 | hsa_4067 | D01688 |
| 2 | hsa_4067 | D01709 |
| 2 | hsa_4067 | D02267 |
| 2 | hsa_4067 | D02559 |
| 2 | hsa_4067 | D02671 |
| 2 | hsa_4067 | D04024 |
| 2 | hsa_4128 | D00168 |
| 2 | hsa_4128 | D00208 |
| 2 | hsa_4128 | D00283 |
| 2 | hsa_4128 | D00434 |
| 2 | hsa_4128 | D00670 |
| 2 | hsa_4128 | D00786 |
| 2 | hsa_4128 | D01133 |
| 2 | hsa_4128 | D01240 |
| 2 | hsa_4128 | D01367 |
| 2 | hsa_4128 | D02168 |
| 2 | hsa_4128 | D02729 |
| 2 | hsa_4129 | D00097 |
| 2 | hsa_4129 | D00126 |
| 2 | hsa_4129 | D00417 |
| 2 | hsa_4129 | D00726 |
| 2 | hsa_4129 | D02267 |
| 2 | hsa_4129 | D03828 |
| 2 | hsa_4143 | D00021 |
| 2 | hsa_4143 | D00045 |
| 2 | hsa_4143 | D00141 |
| 2 | hsa_4143 | D01275 |
| 2 | hsa_4145 | D00007 |
| 2 | hsa_4145 | D00036 |
| 2 | hsa_4145 | D00142 |
| 2 | hsa_4145 | D00364 |
| 2 | hsa_4145 | D00543 |
| 2 | hsa_4145 | D01578 |
| 2 | hsa_4145 | D02731 |
| 2 | hsa_4190 | D00279 |
| 2 | hsa_4190 | D00325 |
| 2 | hsa_4190 | D00364 |
| 2 | hsa_4190 | D00505 |
| 2 | hsa_4190 | D01565 |
| 2 | hsa_4190 | D01973 |
| 2 | hsa_4190 | D02563 |
| 2 | hsa_4190 | D03751 |
| 2 | hsa_4191 | D00145 |
| 2 | hsa_4191 | D00503 |
| 2 | hsa_4191 | D00656 |
| 2 | hsa_4191 | D00785 |
| 2 | hsa_4191 | D01275 |
| 2 | hsa_4191 | D01862 |
| 2 | hsa_4191 | D02173 |
| 2 | hsa_4191 | D02563 |
| 2 | hsa_4191 | D03712 |
| 2 | hsa_4191 | D03716 |
| 2 | hsa_4191 | D03829 |
| 2 | hsa_4191 | D05458 |
| 2 | hsa_4200 | D00126 |
| 2 | hsa_4200 | D00183 |
| 2 | hsa_4200 | D00452 |
| 2 | hsa_4200 | D00562 |
| 2 | hsa_4200 | D01918 |
| 2 | hsa_4200 | D02068 |
| 2 | hsa_4200 | D02168 |
| 2 | hsa_4200 | D03752 |
| 2 | hsa_4200 | D03758 |
| 2 | hsa_4233 | D00127 |
| 2 | hsa_4233 | D00394 |
| 2 | hsa_4233 | D00496 |
| 2 | hsa_4233 | D00544 |
| 2 | hsa_4233 | D00771 |
| 2 | hsa_4233 | D01069 |
| 2 | hsa_4233 | D01196 |
| 2 | hsa_4233 | D01367 |
| 2 | hsa_4233 | D02356 |
| 2 | hsa_4233 | D03728 |
| 2 | hsa_4233 | D03807 |
| 2 | hsa_4282 | D00132 |
| 2 | hsa_4282 | D00584 |
| 2 | hsa_4282 | D00691 |
| 2 | hsa_4282 | D00726 |
| 2 | hsa_4282 | D00752 |
| 2 | hsa_4282 | D02110 |
| 2 | hsa_4282 | D02560 |
| 2 | hsa_4282 | D03670 |
| 2 | hsa_4282 | D03781 |
| 2 | hsa_4311 | D00036 |
| 2 | hsa_4311 | D00107 |
| 2 | hsa_4311 | D00216 |
| 2 | hsa_4311 | D00475 |
| 2 | hsa_4311 | D00501 |
| 2 | hsa_4311 | D00652 |
| 2 | hsa_4311 | D00998 |
| 2 | hsa_4311 | D02173 |
| 2 | hsa_4329 | D00052 |
| 2 | hsa_4329 | D00414 |
| 2 | hsa_4329 | D00781 |
| 2 | hsa_4329 | D00904 |
| 2 | hsa_4329 | D01061 |
| 2 | hsa_4329 | D01825 |
| 2 | hsa_4329 | D02176 |
| 2 | hsa_4329 | D02709 |
| 2 | hsa_4329 | D03689 |
| 2 | hsa_4329 | D05341 |
| 2 | hsa_4353 | D00018 |
| 2 | hsa_4353 | D00118 |
| 2 | hsa_4353 | D00423 |
| 2 | hsa_4353 | D00519 |
| 2 | hsa_4353 | D00579 |
| 2 | hsa_4353 | D00624 |
| 2 | hsa_4353 | D00670 |
| 2 | hsa_4353 | D00786 |
| 2 | hsa_4353 | D00947 |
| 2 | hsa_4353 | D01136 |
| 2 | hsa_4353 | D01275 |
| 2 | hsa_4353 | D01688 |
| 2 | hsa_4353 | D01915 |
| 2 | hsa_4353 | D03806 |
| 2 | hsa_4353 | D03807 |
| 2 | hsa_4353 | D04025 |
| 2 | hsa_4353 | D04028 |
| 2 | hsa_43 | D00029 |
| 2 | hsa_43 | D00103 |
| 2 | hsa_43 | D00475 |
| 2 | hsa_43 | D00512 |
| 2 | hsa_43 | D00546 |
| 2 | hsa_43 | D02731 |
| 2 | hsa_43 | D03433 |
| 2 | hsa_43 | D03736 |
| 2 | hsa_444 | D00141 |
| 2 | hsa_444 | D00651 |
| 2 | hsa_444 | D00658 |
| 2 | hsa_444 | D00805 |
| 2 | hsa_444 | D00892 |
| 2 | hsa_444 | D00969 |
| 2 | hsa_444 | D01547 |
| 2 | hsa_444 | D01907 |
| 2 | hsa_444 | D02729 |
| 2 | hsa_444 | D03077 |
| 2 | hsa_4482 | D00234 |
| 2 | hsa_4482 | D00624 |
| 2 | hsa_4482 | D01432 |
| 2 | hsa_4486 | D00203 |
| 2 | hsa_4486 | D00487 |
| 2 | hsa_4486 | D00965 |
| 2 | hsa_4486 | D01918 |
| 2 | hsa_4486 | D03689 |
| 2 | hsa_4548 | D00414 |
| 2 | hsa_4548 | D00448 |
| 2 | hsa_4548 | D00512 |
| 2 | hsa_4548 | D00515 |
| 2 | hsa_4548 | D00562 |
| 2 | hsa_4548 | D00654 |
| 2 | hsa_4548 | D00709 |
| 2 | hsa_4548 | D01974 |
| 2 | hsa_4548 | D02068 |
| 2 | hsa_4548 | D02110 |
| 2 | hsa_4548 | D02729 |
| 2 | hsa_4548 | D03643 |
| 2 | hsa_4548 | D03751 |
| 2 | hsa_4552 | D00510 |
| 2 | hsa_4552 | D02328 |
| 2 | hsa_4552 | D03350 |
| 2 | hsa_4552 | D03756 |
| 2 | hsa_4593 | D00036 |
| 2 | hsa_4593 | D00097 |
| 2 | hsa_4593 | D00620 |
| 2 | hsa_4593 | D00892 |
| 2 | hsa_4593 | D00963 |
| 2 | hsa_4593 | D01196 |
| 2 | hsa_4593 | D01866 |
| 2 | hsa_4593 | D02323 |
| 2 | hsa_4593 | D03899 |
| 2 | hsa_4594 | D00125 |
| 2 | hsa_4594 | D01441 |
| 2 | hsa_4594 | D01690 |
| 2 | hsa_4594 | D01825 |
| 2 | hsa_4594 | D01915 |
| 2 | hsa_4594 | D02176 |
| 2 | hsa_4594 | D02451 |
| 2 | hsa_4594 | D03798 |
| 2 | hsa_4594 | D03829 |
| 2 | hsa_4758 | D00317 |
| 2 | hsa_4758 | D00401 |
| 2 | hsa_4758 | D00463 |
| 2 | hsa_4758 | D00503 |
| 2 | hsa_4758 | D00670 |
| 2 | hsa_4758 | D00893 |
| 2 | hsa_4758 | D01811 |
| 2 | hsa_4758 | D02115 |
| 2 | hsa_4759 | D00231 |
| 2 | hsa_4759 | D00371 |
| 2 | hsa_4759 | D00459 |
| 2 | hsa_4759 | D00596 |
| 2 | hsa_4759 | D00889 |
| 2 | hsa_4759 | D01064 |
| 2 | hsa_4759 | D02355 |
| 2 | hsa_4759 | D03433 |
| 2 | hsa_4759 | D03803 |
| 2 | hsa_4759 | D04292 |
| 2 | hsa_476 | D00324 |
| 2 | hsa_476 | D00328 |
| 2 | hsa_476 | D00369 |
| 2 | hsa_476 | D00425 |
| 2 | hsa_476 | D00535 |
| 2 | hsa_476 | D00691 |
| 2 | hsa_476 | D01370 |
| 2 | hsa_476 | D01888 |
| 2 | hsa_4835 | D01840 |
| 2 | hsa_4835 | D01885 |
| 2 | hsa_4835 | D01900 |
| 2 | hsa_4835 | D03012 |
| 2 | hsa_4835 | D03753 |
| 2 | hsa_4835 | D04024 |
| 2 | hsa_4835 | D04983 |
| 2 | hsa_4837 | D00097 |
| 2 | hsa_4837 | D00398 |
| 2 | hsa_4837 | D00417 |
| 2 | hsa_4837 | D00560 |
| 2 | hsa_4837 | D01565 |
| 2 | hsa_4837 | D02355 |
| 2 | hsa_4837 | D02356 |
| 2 | hsa_4837 | D02368 |
| 2 | hsa_4837 | D04197 |
| 2 | hsa_4860 | D00103 |
| 2 | hsa_4860 | D00158 |
| 2 | hsa_4860 | D00198 |
| 2 | hsa_4860 | D00333 |
| 2 | hsa_4860 | D00518 |
| 2 | hsa_4860 | D00781 |
| 2 | hsa_4860 | D00900 |
| 2 | hsa_4860 | D01276 |
| 2 | hsa_4860 | D01715 |
| 2 | hsa_4860 | D02356 |
| 2 | hsa_4860 | D02835 |
| 2 | hsa_4860 | D03822 |
| 2 | hsa_4881 | D00183 |
| 2 | hsa_4881 | D00330 |
| 2 | hsa_4881 | D00437 |
| 2 | hsa_4881 | D00494 |
| 2 | hsa_4881 | D00566 |
| 2 | hsa_4881 | D01825 |
| 2 | hsa_4881 | D01911 |
| 2 | hsa_4881 | D01977 |
| 2 | hsa_4881 | D02166 |
| 2 | hsa_4881 | D02671 |
| 2 | hsa_4881 | D03798 |
| 2 | hsa_4907 | D00039 |
| 2 | hsa_4907 | D00186 |
| 2 | hsa_4907 | D00227 |
| 2 | hsa_4907 | D00283 |
| 2 | hsa_4907 | D00533 |
| 2 | hsa_4907 | D00733 |
| 2 | hsa_4907 | D01164 |
| 2 | hsa_4907 | D01180 |
| 2 | hsa_4907 | D01367 |
| 2 | hsa_4907 | D02451 |
| 2 | hsa_4907 | D03798 |
| 2 | hsa_4914 | D00398 |
| 2 | hsa_4914 | D00423 |
| 2 | hsa_4914 | D00622 |
| 2 | hsa_4914 | D00889 |
| 2 | hsa_4914 | D01667 |
| 2 | hsa_4914 | D02564 |
| 2 | hsa_4914 | D03115 |
| 2 | hsa_4914 | D03752 |
| 2 | hsa_4914 | D03788 |
| 2 | hsa_4914 | D03806 |
| 2 | hsa_4915 | D00203 |
| 2 | hsa_4915 | D00218 |
| 2 | hsa_4915 | D00300 |
| 2 | hsa_4915 | D00519 |
| 2 | hsa_4915 | D00785 |
| 2 | hsa_4915 | D00994 |
| 2 | hsa_4915 | D02558 |
| 2 | hsa_4915 | D03760 |
| 2 | hsa_4915 | D03765 |
| 2 | hsa_4915 | D03803 |
| 2 | hsa_4915 | D05458 |
| 2 | hsa_4916 | D00342 |
| 2 | hsa_4916 | D00621 |
| 2 | hsa_4916 | D00653 |
| 2 | hsa_4916 | D00903 |
| 2 | hsa_4916 | D01136 |
| 2 | hsa_4916 | D01765 |
| 2 | hsa_4916 | D02017 |
| 2 | hsa_4916 | D02355 |
| 2 | hsa_4916 | D03733 |
| 2 | hsa_4916 | D03735 |
| 2 | hsa_4919 | D00007 |
| 2 | hsa_4919 | D00094 |
| 2 | hsa_4919 | D00136 |
| 2 | hsa_4919 | D00293 |
| 2 | hsa_4919 | D00398 |
| 2 | hsa_4919 | D00425 |
| 2 | hsa_4919 | D00726 |
| 2 | hsa_4919 | D00892 |
| 2 | hsa_4919 | D01549 |
| 2 | hsa_4919 | D02289 |
| 2 | hsa_4919 | D03717 |
| 2 | hsa_4919 | D04031 |
| 2 | hsa_4920 | D00494 |
| 2 | hsa_4920 | D00538 |
| 2 | hsa_4920 | D00546 |
| 2 | hsa_4920 | D00691 |
| 2 | hsa_4920 | D00947 |
| 2 | hsa_4920 | D01432 |
| 2 | hsa_4920 | D03798 |
| 2 | hsa_4920 | D03807 |
| 2 | hsa_4921 | D00094 |
| 2 | hsa_4921 | D00120 |
| 2 | hsa_4921 | D00448 |
| 2 | hsa_4921 | D00475 |
| 2 | hsa_4921 | D00535 |
| 2 | hsa_4921 | D00543 |
| 2 | hsa_4921 | D00560 |
| 2 | hsa_4921 | D00579 |
| 2 | hsa_4921 | D00658 |
| 2 | hsa_4921 | D01346 |
| 2 | hsa_4921 | D02168 |
| 2 | hsa_4921 | D02559 |
| 2 | hsa_4921 | D03712 |
| 2 | hsa_4921 | D03882 |
| 2 | hsa_4921 | D04197 |
| 2 | hsa_4942 | D00120 |
| 2 | hsa_4942 | D00252 |
| 2 | hsa_4942 | D00463 |
| 2 | hsa_4942 | D00691 |
| 2 | hsa_4942 | D01071 |
| 2 | hsa_4942 | D01183 |
| 2 | hsa_4942 | D01549 |
| 2 | hsa_4942 | D03753 |
| 2 | hsa_4942 | D03776 |
| 2 | hsa_4942 | D03822 |
| 2 | hsa_4953 | D00188 |
| 2 | hsa_4953 | D00416 |
| 2 | hsa_4953 | D00425 |
| 2 | hsa_4953 | D00488 |
| 2 | hsa_4953 | D00562 |
| 2 | hsa_4953 | D00667 |
| 2 | hsa_4953 | D01061 |
| 2 | hsa_4953 | D01256 |
| 2 | hsa_4953 | D01475 |
| 2 | hsa_4953 | D01915 |
| 2 | hsa_4953 | D02566 |
| 2 | hsa_4953 | D03350 |
| 2 | hsa_4953 | D03689 |
| 2 | hsa_495 | D00041 |
| 2 | hsa_495 | D00136 |
| 2 | hsa_495 | D00196 |
| 2 | hsa_495 | D00391 |
| 2 | hsa_495 | D00752 |
| 2 | hsa_495 | D02418 |
| 2 | hsa_495 | D02655 |
| 2 | hsa_495 | D02731 |
| 2 | hsa_49 | D00300 |
| 2 | hsa_49 | D00417 |
| 2 | hsa_49 | D00487 |
| 2 | hsa_49 | D00781 |
| 2 | hsa_49 | D01223 |
| 2 | hsa_49 | D01397 |
| 2 | hsa_49 | D01513 |
| 2 | hsa_49 | D01549 |
| 2 | hsa_49 | D01840 |
| 2 | hsa_49 | D01984 |
| 2 | hsa_49 | D02008 |
| 2 | hsa_49 | D02563 |
| 2 | hsa_49 | D03115 |
| 2 | hsa_49 | D03670 |
| 2 | hsa_49 | D03798 |
| 2 | hsa_49 | D03822 |
| 2 | hsa_501 | D00032 |
| 2 | hsa_501 | D00035 |
| 2 | hsa_501 | D00535 |
| 2 | hsa_501 | D00563 |
| 2 | hsa_501 | D00709 |
| 2 | hsa_501 | D01027 |
| 2 | hsa_501 | D01397 |
| 2 | hsa_501 | D02166 |
| 2 | hsa_501 | D02368 |
| 2 | hsa_501 | D02564 |
| 2 | hsa_5033 | D00196 |
| 2 | hsa_5033 | D00274 |
| 2 | hsa_5033 | D00535 |
| 2 | hsa_5033 | D00620 |
| 2 | hsa_5033 | D00885 |
| 2 | hsa_5033 | D00887 |
| 2 | hsa_5033 | D02166 |
| 2 | hsa_5033 | D02214 |
| 2 | hsa_5033 | D02835 |
| 2 | hsa_5033 | D03788 |
| 2 | hsa_5033 | D03899 |
| 2 | hsa_5045 | D00018 |
| 2 | hsa_5045 | D00231 |
| 2 | hsa_5045 | D00448 |
| 2 | hsa_5045 | D00459 |
| 2 | hsa_5045 | D00651 |
| 2 | hsa_5045 | D01441 |
| 2 | hsa_5045 | D01688 |
| 2 | hsa_5045 | D02835 |
| 2 | hsa_5045 | D03784 |
| 2 | hsa_50484 | D00132 |
| 2 | hsa_50484 | D00904 |
| 2 | hsa_50484 | D03741 |
| 2 | hsa_50487 | D00726 |
| 2 | hsa_50487 | D02671 |
| 2 | hsa_50487 | D03743 |
| 2 | hsa_50487 | D03758 |
| 2 | hsa_50487 | D03829 |
| 2 | hsa_5049 | D00021 |
| 2 | hsa_5049 | D00317 |
| 2 | hsa_5049 | D00518 |
| 2 | hsa_5049 | D02193 |
| 2 | hsa_5049 | D02355 |
| 2 | hsa_5049 | D02756 |
| 2 | hsa_5049 | D03822 |
| 2 | hsa_5050 | D00126 |
| 2 | hsa_5050 | D00518 |
| 2 | hsa_5050 | D00550 |
| 2 | hsa_5050 | D00810 |
| 2 | hsa_5050 | D00829 |
| 2 | hsa_5050 | D00887 |
| 2 | hsa_5050 | D01001 |
| 2 | hsa_5050 | D02110 |
| 2 | hsa_5050 | D02315 |
| 2 | hsa_5050 | D03077 |
| 2 | hsa_5051 | D00125 |
| 2 | hsa_5051 | D00169 |
| 2 | hsa_5051 | D00449 |
| 2 | hsa_5051 | D00655 |
| 2 | hsa_5051 | D00964 |
| 2 | hsa_5051 | D01071 |
| 2 | hsa_5051 | D02068 |
| 2 | hsa_5051 | D02321 |
| 2 | hsa_5051 | D02355 |
| 2 | hsa_5051 | D04197 |
| 2 | hsa_5051 | D04966 |
| 2 | hsa_5052 | D00130 |
| 2 | hsa_5052 | D00298 |
| 2 | hsa_5052 | D00300 |
| 2 | hsa_5052 | D00463 |
| 2 | hsa_5052 | D00568 |
| 2 | hsa_5052 | D00703 |
| 2 | hsa_5052 | D00965 |
| 2 | hsa_5052 | D02563 |
| 2 | hsa_5052 | D03716 |
| 2 | hsa_5052 | D04029 |
| 2 | hsa_5053 | D00184 |
| 2 | hsa_5053 | D00293 |
| 2 | hsa_5053 | D02173 |
| 2 | hsa_5053 | D03077 |
| 2 | hsa_5091 | D00184 |
| 2 | hsa_5091 | D00494 |
| 2 | hsa_5091 | D00654 |
| 2 | hsa_5091 | D01136 |
| 2 | hsa_5091 | D01346 |
| 2 | hsa_5091 | D01825 |
| 2 | hsa_5091 | D03714 |
| 2 | hsa_50940 | D00216 |
| 2 | hsa_50940 | D00315 |
| 2 | hsa_50940 | D00369 |
| 2 | hsa_50940 | D00577 |
| 2 | hsa_50940 | D00753 |
| 2 | hsa_50940 | D00813 |
| 2 | hsa_50940 | D01578 |
| 2 | hsa_50940 | D02166 |
| 2 | hsa_50940 | D02368 |
| 2 | hsa_50940 | D03440 |
| 2 | hsa_50940 | D03733 |
| 2 | hsa_50940 | D03752 |
| 2 | hsa_50940 | D04029 |
| 2 | hsa_5095 | D00186 |
| 2 | hsa_5095 | D00227 |
| 2 | hsa_5095 | D00298 |
| 2 | hsa_5095 | D01118 |
| 2 | hsa_5095 | D02323 |
| 2 | hsa_5095 | D02709 |
| 2 | hsa_5095 | D03670 |
| 2 | hsa_5095 | D03788 |
| 2 | hsa_5096 | D00126 |
| 2 | hsa_5096 | D00332 |
| 2 | hsa_5096 | D00398 |
| 2 | hsa_5096 | D00475 |
| 2 | hsa_5096 | D00903 |
| 2 | hsa_5096 | D01097 |
| 2 | hsa_5096 | D01196 |
| 2 | hsa_5096 | D01665 |
| 2 | hsa_5096 | D01715 |
| 2 | hsa_5096 | D02342 |
| 2 | hsa_5096 | D03760 |
| 2 | hsa_50 | D00027 |
| 2 | hsa_50 | D00454 |
| 2 | hsa_50 | D00622 |
| 2 | hsa_50 | D00884 |
| 2 | hsa_50 | D01276 |
| 2 | hsa_50 | D02487 |
| 2 | hsa_50 | D03752 |
| 2 | hsa_50 | D03773 |
| 2 | hsa_51004 | D00043 |
| 2 | hsa_51004 | D00132 |
| 2 | hsa_51004 | D00544 |
| 2 | hsa_51004 | D01119 |
| 2 | hsa_51004 | D01840 |
| 2 | hsa_51004 | D02193 |
| 2 | hsa_51004 | D02335 |
| 2 | hsa_51004 | D02671 |
| 2 | hsa_51004 | D03798 |
| 2 | hsa_51004 | D03826 |
| 2 | hsa_51095 | D00434 |
| 2 | hsa_51095 | D00452 |
| 2 | hsa_51095 | D00786 |
| 2 | hsa_51095 | D00827 |
| 2 | hsa_51095 | D00829 |
| 2 | hsa_51095 | D02308 |
| 2 | hsa_51095 | D03728 |
| 2 | hsa_51095 | D03784 |
| 2 | hsa_51166 | D00279 |
| 2 | hsa_51166 | D00391 |
| 2 | hsa_51166 | D00401 |
| 2 | hsa_51166 | D00567 |
| 2 | hsa_51166 | D00691 |
| 2 | hsa_51166 | D01582 |
| 2 | hsa_51166 | D02193 |
| 2 | hsa_51172 | D00054 |
| 2 | hsa_51172 | D00726 |
| 2 | hsa_51172 | D00994 |
| 2 | hsa_51172 | D03720 |
| 2 | hsa_51181 | D00103 |
| 2 | hsa_51181 | D00562 |
| 2 | hsa_51181 | D00667 |
| 2 | hsa_51181 | D00998 |
| 2 | hsa_51181 | D01061 |
| 2 | hsa_51181 | D01712 |
| 2 | hsa_51181 | D02558 |
| 2 | hsa_51181 | D03716 |
| 2 | hsa_51205 | D00136 |
| 2 | hsa_51205 | D00222 |
| 2 | hsa_51205 | D00380 |
| 2 | hsa_51205 | D00454 |
| 2 | hsa_51205 | D00513 |
| 2 | hsa_51205 | D00887 |
| 2 | hsa_51205 | D00900 |
| 2 | hsa_51205 | D01974 |
| 2 | hsa_51205 | D02323 |
| 2 | hsa_51205 | D03899 |
| 2 | hsa_51207 | D00037 |
| 2 | hsa_51207 | D00203 |
| 2 | hsa_51207 | D00251 |
| 2 | hsa_51207 | D00377 |
| 2 | hsa_51207 | D00451 |
| 2 | hsa_51207 | D00528 |
| 2 | hsa_51207 | D02562 |
| 2 | hsa_51207 | D02698 |
| 2 | hsa_51207 | D03710 |
| 2 | hsa_51207 | D03733 |
| 2 | hsa_51207 | D03798 |
| 2 | hsa_5122 | D00298 |
| 2 | hsa_5122 | D00342 |
| 2 | hsa_5122 | D00786 |
| 2 | hsa_5122 | D01667 |
| 2 | hsa_5122 | D02558 |
| 2 | hsa_5122 | D03773 |
| 2 | hsa_5122 | D03788 |
| 2 | hsa_51251 | D00052 |
| 2 | hsa_51251 | D00188 |
| 2 | hsa_51251 | D00274 |
| 2 | hsa_51251 | D00279 |
| 2 | hsa_51251 | D00342 |
| 2 | hsa_51251 | D00544 |
| 2 | hsa_51251 | D00622 |
| 2 | hsa_51251 | D00726 |
| 2 | hsa_51251 | D01325 |
| 2 | hsa_51251 | D01364 |
| 2 | hsa_51251 | D01547 |
| 2 | hsa_51251 | D03350 |
| 2 | hsa_51251 | D03752 |
| 2 | hsa_51251 | D03805 |
| 2 | hsa_5126 | D00188 |
| 2 | hsa_5126 | D00364 |
| 2 | hsa_5126 | D00377 |
| 2 | hsa_5126 | D00538 |
| 2 | hsa_5126 | D02335 |
| 2 | hsa_5126 | D03433 |
| 2 | hsa_5126 | D03722 |
| 2 | hsa_5126 | D04024 |
| 2 | hsa_51292 | D00142 |
| 2 | hsa_51292 | D00325 |
| 2 | hsa_51292 | D00537 |
| 2 | hsa_51292 | D00620 |
| 2 | hsa_51292 | D01709 |
| 2 | hsa_51292 | D01715 |
| 2 | hsa_51292 | D03440 |
| 2 | hsa_51292 | D03753 |
| 2 | hsa_51302 | D00516 |
| 2 | hsa_51302 | D00535 |
| 2 | hsa_51302 | D00593 |
| 2 | hsa_51302 | D00621 |
| 2 | hsa_51302 | D00829 |
| 2 | hsa_51302 | D00995 |
| 2 | hsa_51302 | D02017 |
| 2 | hsa_51302 | D02355 |
| 2 | hsa_51302 | D02487 |
| 2 | hsa_51365 | D00188 |
| 2 | hsa_51365 | D00294 |
| 2 | hsa_51365 | D00544 |
| 2 | hsa_51365 | D01885 |
| 2 | hsa_51365 | D03440 |
| 2 | hsa_51365 | D03716 |
| 2 | hsa_51365 | D03776 |
| 2 | hsa_51365 | D04983 |
| 2 | hsa_5136 | D00002 |
| 2 | hsa_5136 | D00148 |
| 2 | hsa_5136 | D00421 |
| 2 | hsa_5136 | D00577 |
| 2 | hsa_5136 | D01667 |
| 2 | hsa_5136 | D02173 |
| 2 | hsa_5136 | D02176 |
| 2 | hsa_5136 | D02350 |
| 2 | hsa_5136 | D03722 |
| 2 | hsa_5136 | D03781 |
| 2 | hsa_5136 | D05341 |
| 2 | hsa_5137 | D00049 |
| 2 | hsa_5137 | D00125 |
| 2 | hsa_5137 | D00188 |
| 2 | hsa_5137 | D00270 |
| 2 | hsa_5137 | D00567 |
| 2 | hsa_5137 | D00625 |
| 2 | hsa_5137 | D00826 |
| 2 | hsa_5137 | D02258 |
| 2 | hsa_5137 | D02368 |
| 2 | hsa_5137 | D02769 |
| 2 | hsa_5137 | D03670 |
| 2 | hsa_5137 | D03776 |
| 2 | hsa_5137 | D03828 |
| 2 | hsa_5137 | D04024 |
| 2 | hsa_5138 | D00234 |
| 2 | hsa_5138 | D00317 |
| 2 | hsa_5138 | D00369 |
| 2 | hsa_5138 | D00726 |
| 2 | hsa_5138 | D01211 |
| 2 | hsa_5138 | D02115 |
| 2 | hsa_5138 | D02441 |
| 2 | hsa_5138 | D03765 |
| 2 | hsa_513 | D00218 |
| 2 | hsa_513 | D00579 |
| 2 | hsa_513 | D01136 |
| 2 | hsa_513 | D01228 |
| 2 | hsa_513 | D02756 |
| 2 | hsa_513 | D03716 |
| 2 | hsa_513 | D03720 |
| 2 | hsa_5140 | D00035 |
| 2 | hsa_5140 | D00519 |
| 2 | hsa_5140 | D01840 |
| 2 | hsa_5141 | D00032 |
| 2 | hsa_5141 | D00224 |
| 2 | hsa_5141 | D00577 |
| 2 | hsa_5141 | D01549 |
| 2 | hsa_5141 | D03743 |
| 2 | hsa_5142 | D00315 |
| 2 | hsa_5142 | D00416 |
| 2 | hsa_5142 | D00496 |
| 2 | hsa_5142 | D00596 |
| 2 | hsa_5142 | D00998 |
| 2 | hsa_5142 | D01981 |
| 2 | hsa_5142 | D03788 |
| 2 | hsa_5143 | D00125 |
| 2 | hsa_5143 | D00142 |
| 2 | hsa_5143 | D00298 |
| 2 | hsa_5143 | D00342 |
| 2 | hsa_5143 | D00487 |
| 2 | hsa_5143 | D01001 |
| 2 | hsa_5143 | D01275 |
| 2 | hsa_5143 | D01325 |
| 2 | hsa_5143 | D02328 |
| 2 | hsa_5143 | D03012 |
| 2 | hsa_5143 | D03714 |
| 2 | hsa_5143 | D03733 |
| 2 | hsa_5143 | D03751 |
| 2 | hsa_5144 | D00536 |
| 2 | hsa_5144 | D00726 |
| 2 | hsa_5144 | D02308 |
| 2 | hsa_5144 | D02368 |
| 2 | hsa_5144 | D02441 |
| 2 | hsa_5144 | D03077 |
| 2 | hsa_5144 | D03751 |
| 2 | hsa_5144 | D03758 |
| 2 | hsa_5144 | D03772 |
| 2 | hsa_5144 | D03806 |
| 2 | hsa_5144 | D03899 |
| 2 | hsa_5145 | D00127 |
| 2 | hsa_5145 | D00330 |
| 2 | hsa_5145 | D00505 |
| 2 | hsa_5145 | D00882 |
| 2 | hsa_5145 | D01425 |
| 2 | hsa_5145 | D01767 |
| 2 | hsa_5145 | D02173 |
| 2 | hsa_5145 | D02328 |
| 2 | hsa_5145 | D02368 |
| 2 | hsa_5145 | D02564 |
| 2 | hsa_5145 | D02566 |
| 2 | hsa_5145 | D03788 |
| 2 | hsa_5146 | D00562 |
| 2 | hsa_5146 | D00887 |
| 2 | hsa_5146 | D01667 |
| 2 | hsa_5146 | D02333 |
| 2 | hsa_5146 | D03733 |
| 2 | hsa_5147 | D00448 |
| 2 | hsa_5147 | D00537 |
| 2 | hsa_5147 | D00624 |
| 2 | hsa_5147 | D00753 |
| 2 | hsa_5147 | D00786 |
| 2 | hsa_5147 | D01183 |
| 2 | hsa_5147 | D01240 |
| 2 | hsa_5147 | D01767 |
| 2 | hsa_5147 | D02556 |
| 2 | hsa_5147 | D03738 |
| 2 | hsa_5147 | D03769 |
| 2 | hsa_5147 | D03899 |
| 2 | hsa_5147 | D04024 |
| 2 | hsa_5148 | D00538 |
| 2 | hsa_5148 | D00753 |
| 2 | hsa_5148 | D01974 |
| 2 | hsa_5148 | D02290 |
| 2 | hsa_5149 | D00279 |
| 2 | hsa_5149 | D00283 |
| 2 | hsa_5149 | D00325 |
| 2 | hsa_5149 | D00449 |
| 2 | hsa_5149 | D00547 |
| 2 | hsa_5149 | D01346 |
| 2 | hsa_5149 | D01974 |
| 2 | hsa_5149 | D02258 |
| 2 | hsa_5149 | D03716 |
| 2 | hsa_5149 | D03738 |
| 2 | hsa_5149 | D04983 |
| 2 | hsa_5150 | D00052 |
| 2 | hsa_5150 | D00340 |
| 2 | hsa_5150 | D00596 |
| 2 | hsa_5150 | D00726 |
| 2 | hsa_5150 | D01097 |
| 2 | hsa_5150 | D01825 |
| 2 | hsa_5150 | D02315 |
| 2 | hsa_5150 | D02368 |
| 2 | hsa_5150 | D02561 |
| 2 | hsa_5150 | D02835 |
| 2 | hsa_5151 | D00054 |
| 2 | hsa_5151 | D01211 |
| 2 | hsa_5151 | D02487 |
| 2 | hsa_5151 | D03743 |
| 2 | hsa_5151 | D03765 |
| 2 | hsa_5151 | D03806 |
| 2 | hsa_5152 | D00487 |
| 2 | hsa_5152 | D00567 |
| 2 | hsa_5152 | D00998 |
| 2 | hsa_5152 | D01027 |
| 2 | hsa_5152 | D01325 |
| 2 | hsa_5152 | D01885 |
| 2 | hsa_5152 | D02769 |
| 2 | hsa_5152 | D03806 |
| 2 | hsa_5156 | D00394 |
| 2 | hsa_5156 | D00459 |
| 2 | hsa_5156 | D00463 |
| 2 | hsa_5156 | D00533 |
| 2 | hsa_5156 | D02556 |
| 2 | hsa_5156 | D02562 |
| 2 | hsa_5156 | D03734 |
| 2 | hsa_5156 | D03752 |
| 2 | hsa_5156 | D03773 |
| 2 | hsa_5156 | D03776 |
| 2 | hsa_5156 | D04983 |
| 2 | hsa_5158 | D00021 |
| 2 | hsa_5158 | D00050 |
| 2 | hsa_5158 | D00251 |
| 2 | hsa_5158 | D00425 |
| 2 | hsa_5158 | D00449 |
| 2 | hsa_5158 | D01240 |
| 2 | hsa_5158 | D02258 |
| 2 | hsa_5158 | D02579 |
| 2 | hsa_5158 | D03722 |
| 2 | hsa_5158 | D04028 |
| 2 | hsa_5158 | D04031 |
| 2 | hsa_5159 | D00052 |
| 2 | hsa_5159 | D00094 |
| 2 | hsa_5159 | D00153 |
| 2 | hsa_5159 | D00168 |
| 2 | hsa_5159 | D00188 |
| 2 | hsa_5159 | D00417 |
| 2 | hsa_5159 | D00452 |
| 2 | hsa_5159 | D00543 |
| 2 | hsa_5159 | D00904 |
| 2 | hsa_5159 | D02556 |
| 2 | hsa_5159 | D03643 |
| 2 | hsa_51645 | D00142 |
| 2 | hsa_51645 | D00203 |
| 2 | hsa_51645 | D00270 |
| 2 | hsa_51645 | D00387 |
| 2 | hsa_51645 | D00574 |
| 2 | hsa_51645 | D00810 |
| 2 | hsa_51645 | D00995 |
| 2 | hsa_51645 | D01840 |
| 2 | hsa_51645 | D02115 |
| 2 | hsa_51645 | D02579 |
| 2 | hsa_51645 | D04983 |
| 2 | hsa_51645 | D05341 |
| 2 | hsa_5167 | D00126 |
| 2 | hsa_5167 | D00544 |
| 2 | hsa_5167 | D00621 |
| 2 | hsa_5167 | D01183 |
| 2 | hsa_5167 | D01767 |
| 2 | hsa_5168 | D00120 |
| 2 | hsa_5168 | D00198 |
| 2 | hsa_5168 | D00283 |
| 2 | hsa_5168 | D00300 |
| 2 | hsa_5168 | D00330 |
| 2 | hsa_5168 | D00387 |
| 2 | hsa_5168 | D00437 |
| 2 | hsa_5168 | D00655 |
| 2 | hsa_5168 | D01136 |
| 2 | hsa_5168 | D01825 |
| 2 | hsa_5168 | D03712 |
| 2 | hsa_5168 | D03733 |
| 2 | hsa_5168 | D03778 |
| 2 | hsa_5169 | D00158 |
| 2 | hsa_5169 | D00494 |
| 2 | hsa_5169 | D00537 |
| 2 | hsa_5169 | D00995 |
| 2 | hsa_5169 | D01432 |
| 2 | hsa_5169 | D01828 |
| 2 | hsa_51727 | D00097 |
| 2 | hsa_51727 | D00340 |
| 2 | hsa_51727 | D00377 |
| 2 | hsa_51727 | D00417 |
| 2 | hsa_51727 | D00623 |
| 2 | hsa_51727 | D03012 |
| 2 | hsa_51727 | D03440 |
| 2 | hsa_51727 | D04292 |
| 2 | hsa_51 | D00049 |
| 2 | hsa_51 | D00065 |
| 2 | hsa_51 | D00168 |
| 2 | hsa_51 | D00562 |
| 2 | hsa_51 | D00709 |
| 2 | hsa_51 | D00753 |
| 2 | hsa_51 | D00810 |
| 2 | hsa_51 | D00969 |
| 2 | hsa_51 | D01164 |
| 2 | hsa_51 | D03601 |
| 2 | hsa_51 | D03738 |
| 2 | hsa_51 | D03756 |
| 2 | hsa_51 | D04028 |
| 2 | hsa_5294 | D00126 |
| 2 | hsa_5294 | D00227 |
| 2 | hsa_5294 | D00364 |
| 2 | hsa_5294 | D00434 |
| 2 | hsa_5294 | D00805 |
| 2 | hsa_5294 | D01211 |
| 2 | hsa_5294 | D01276 |
| 2 | hsa_5294 | D02418 |
| 2 | hsa_5294 | D04028 |
| 2 | hsa_52 | D00621 |
| 2 | hsa_52 | D02341 |
| 2 | hsa_52 | D02729 |
| 2 | hsa_52 | D03784 |
| 2 | hsa_5319 | D00136 |
| 2 | hsa_5319 | D00364 |
| 2 | hsa_5319 | D00567 |
| 2 | hsa_5319 | D00771 |
| 2 | hsa_5319 | D00903 |
| 2 | hsa_5319 | D01911 |
| 2 | hsa_5319 | D02289 |
| 2 | hsa_5320 | D00136 |
| 2 | hsa_5320 | D00333 |
| 2 | hsa_5320 | D00547 |
| 2 | hsa_5320 | D00654 |
| 2 | hsa_5320 | D01578 |
| 2 | hsa_5320 | D01866 |
| 2 | hsa_5320 | D02289 |
| 2 | hsa_5320 | D02579 |
| 2 | hsa_5320 | D04025 |
| 2 | hsa_5321 | D00451 |
| 2 | hsa_5321 | D00496 |
| 2 | hsa_5321 | D00521 |
| 2 | hsa_5321 | D00900 |
| 2 | hsa_5321 | D00994 |
| 2 | hsa_5321 | D01915 |
| 2 | hsa_5321 | D01984 |
| 2 | hsa_5321 | D02356 |
| 2 | hsa_5321 | D05407 |
| 2 | hsa_5322 | D00234 |
| 2 | hsa_5322 | D00401 |
| 2 | hsa_5322 | D00726 |
| 2 | hsa_5322 | D01164 |
| 2 | hsa_5322 | D01397 |
| 2 | hsa_5322 | D01911 |
| 2 | hsa_5322 | D03077 |
| 2 | hsa_5322 | D03899 |
| 2 | hsa_5322 | D05341 |
| 2 | hsa_5327 | D00145 |
| 2 | hsa_5327 | D00538 |
| 2 | hsa_5327 | D00545 |
| 2 | hsa_5327 | D00567 |
| 2 | hsa_5327 | D00579 |
| 2 | hsa_5327 | D01842 |
| 2 | hsa_5327 | D03012 |
| 2 | hsa_5327 | D03728 |
| 2 | hsa_5327 | D03734 |
| 2 | hsa_5327 | D03775 |
| 2 | hsa_5328 | D00332 |
| 2 | hsa_5328 | D00371 |
| 2 | hsa_5328 | D01064 |
| 2 | hsa_5328 | D01549 |
| 2 | hsa_5328 | D01811 |
| 2 | hsa_5328 | D01844 |
| 2 | hsa_5328 | D02581 |
| 2 | hsa_5328 | D03012 |
| 2 | hsa_5328 | D03772 |
| 2 | hsa_5328 | D03806 |
| 2 | hsa_5330 | D00274 |
| 2 | hsa_5330 | D00960 |
| 2 | hsa_5330 | D01715 |
| 2 | hsa_5330 | D02559 |
| 2 | hsa_5331 | D00007 |
| 2 | hsa_5331 | D00437 |
| 2 | hsa_5331 | D01432 |
| 2 | hsa_5331 | D02350 |
| 2 | hsa_5331 | D05407 |
| 2 | hsa_5332 | D00032 |
| 2 | hsa_5332 | D00654 |
| 2 | hsa_5332 | D00884 |
| 2 | hsa_5332 | D00889 |
| 2 | hsa_5332 | D01001 |
| 2 | hsa_5332 | D01364 |
| 2 | hsa_5332 | D01966 |
| 2 | hsa_5332 | D01974 |
| 2 | hsa_5332 | D02068 |
| 2 | hsa_5332 | D02355 |
| 2 | hsa_5332 | D02671 |
| 2 | hsa_5332 | D03433 |
| 2 | hsa_5332 | D03803 |
| 2 | hsa_5333 | D00332 |
| 2 | hsa_5333 | D00437 |
| 2 | hsa_5333 | D00452 |
| 2 | hsa_5333 | D00968 |
| 2 | hsa_5333 | D01370 |
| 2 | hsa_5333 | D03751 |
| 2 | hsa_5335 | D00002 |
| 2 | hsa_5335 | D00139 |
| 2 | hsa_5335 | D00324 |
| 2 | hsa_5335 | D00535 |
| 2 | hsa_5335 | D00543 |
| 2 | hsa_5335 | D00593 |
| 2 | hsa_5335 | D00733 |
| 2 | hsa_5335 | D01180 |
| 2 | hsa_5335 | D02214 |
| 2 | hsa_5335 | D04029 |
| 2 | hsa_5336 | D00274 |
| 2 | hsa_5336 | D00994 |
| 2 | hsa_5336 | D01256 |
| 2 | hsa_5336 | D02487 |
| 2 | hsa_5336 | D02729 |
| 2 | hsa_5336 | D03826 |
| 2 | hsa_5336 | D04025 |
| 2 | hsa_5337 | D00186 |
| 2 | hsa_5337 | D00317 |
| 2 | hsa_5337 | D00452 |
| 2 | hsa_5337 | D00496 |
| 2 | hsa_5337 | D00584 |
| 2 | hsa_5337 | D00656 |
| 2 | hsa_5337 | D00781 |
| 2 | hsa_5337 | D01027 |
| 2 | hsa_5338 | D00198 |
| 2 | hsa_5338 | D01367 |
| 2 | hsa_5338 | D02315 |
| 2 | hsa_5338 | D02835 |
| 2 | hsa_5340 | D00050 |
| 2 | hsa_5340 | D00324 |
| 2 | hsa_5340 | D00752 |
| 2 | hsa_5340 | D00781 |
| 2 | hsa_5340 | D01397 |
| 2 | hsa_5340 | D01718 |
| 2 | hsa_5340 | D02579 |
| 2 | hsa_5340 | D03823 |
| 2 | hsa_5340 | D04028 |
| 2 | hsa_5340 | D06238 |
| 2 | hsa_5351 | D00216 |
| 2 | hsa_5351 | D00455 |
| 2 | hsa_5351 | D00623 |
| 2 | hsa_5351 | D00655 |
| 2 | hsa_5351 | D00903 |
| 2 | hsa_5351 | D00970 |
| 2 | hsa_5351 | D02110 |
| 2 | hsa_5351 | D02561 |
| 2 | hsa_5351 | D02698 |
| 2 | hsa_5351 | D02729 |
| 2 | hsa_53938 | D00148 |
| 2 | hsa_53938 | D00325 |
| 2 | hsa_53938 | D00394 |
| 2 | hsa_53938 | D00577 |
| 2 | hsa_53938 | D00596 |
| 2 | hsa_53938 | D00650 |
| 2 | hsa_53938 | D00691 |
| 2 | hsa_53938 | D03828 |
| 2 | hsa_53938 | D05407 |
| 2 | hsa_53 | D00036 |
| 2 | hsa_53 | D01118 |
| 2 | hsa_53 | D02441 |
| 2 | hsa_53 | D02560 |
| 2 | hsa_53 | D03741 |
| 2 | hsa_53 | D03751 |
| 2 | hsa_53 | D05407 |
| 2 | hsa_53 | D05458 |
| 2 | hsa_5406 | D00018 |
| 2 | hsa_5406 | D00153 |
| 2 | hsa_5406 | D00285 |
| 2 | hsa_5406 | D00325 |
| 2 | hsa_5406 | D00332 |
| 2 | hsa_5406 | D00547 |
| 2 | hsa_5406 | D01582 |
| 2 | hsa_5406 | D01767 |
| 2 | hsa_5406 | D01907 |
| 2 | hsa_5406 | D02068 |
| 2 | hsa_5407 | D00251 |
| 2 | hsa_5407 | D00515 |
| 2 | hsa_5407 | D01069 |
| 2 | hsa_5407 | D01900 |
| 2 | hsa_5407 | D02581 |
| 2 | hsa_5408 | D00050 |
| 2 | hsa_5408 | D00130 |
| 2 | hsa_5408 | D00785 |
| 2 | hsa_5408 | D01071 |
| 2 | hsa_5408 | D01332 |
| 2 | hsa_5408 | D02564 |
| 2 | hsa_5422 | D00007 |
| 2 | hsa_5422 | D00342 |
| 2 | hsa_5422 | D01061 |
| 2 | hsa_5422 | D01441 |
| 2 | hsa_5422 | D01547 |
| 2 | hsa_5422 | D01977 |
| 2 | hsa_5422 | D02559 |
| 2 | hsa_5423 | D00041 |
| 2 | hsa_5423 | D00208 |
| 2 | hsa_5423 | D00771 |
| 2 | hsa_5423 | D00826 |
| 2 | hsa_5423 | D01256 |
| 2 | hsa_5423 | D02321 |
| 2 | hsa_5423 | D02341 |
| 2 | hsa_5423 | D03716 |
| 2 | hsa_5423 | D03798 |
| 2 | hsa_5444 | D00052 |
| 2 | hsa_5444 | D00132 |
| 2 | hsa_5444 | D00322 |
| 2 | hsa_5444 | D00488 |
| 2 | hsa_5444 | D00596 |
| 2 | hsa_5444 | D01136 |
| 2 | hsa_5444 | D02561 |
| 2 | hsa_5444 | D03798 |
| 2 | hsa_5445 | D01968 |
| 2 | hsa_5445 | D02328 |
| 2 | hsa_5445 | D02341 |
| 2 | hsa_5445 | D02559 |
| 2 | hsa_5445 | D02835 |
| 2 | hsa_5447 | D00002 |
| 2 | hsa_5447 | D00007 |
| 2 | hsa_5447 | D00097 |
| 2 | hsa_5447 | D00333 |
| 2 | hsa_5447 | D00434 |
| 2 | hsa_5447 | D00451 |
| 2 | hsa_5447 | D00579 |
| 2 | hsa_5447 | D00652 |
| 2 | hsa_5447 | D01240 |
| 2 | hsa_5447 | D02350 |
| 2 | hsa_5447 | D02375 |
| 2 | hsa_5447 | D03784 |
| 2 | hsa_5447 | D04292 |
| 2 | hsa_54490 | D00141 |
| 2 | hsa_54490 | D00168 |
| 2 | hsa_54490 | D00294 |
| 2 | hsa_54490 | D00410 |
| 2 | hsa_54490 | D00421 |
| 2 | hsa_54490 | D00569 |
| 2 | hsa_54490 | D02563 |
| 2 | hsa_54490 | D03734 |
| 2 | hsa_54490 | D04031 |
| 2 | hsa_54575 | D00455 |
| 2 | hsa_54575 | D00733 |
| 2 | hsa_54575 | D01136 |
| 2 | hsa_54575 | D01275 |
| 2 | hsa_54575 | D01370 |
| 2 | hsa_54575 | D02166 |
| 2 | hsa_54575 | D02341 |
| 2 | hsa_54575 | D02356 |
| 2 | hsa_54575 | D03722 |
| 2 | hsa_54576 | D00274 |
| 2 | hsa_54576 | D00317 |
| 2 | hsa_54576 | D00391 |
| 2 | hsa_54576 | D00475 |
| 2 | hsa_54576 | D00545 |
| 2 | hsa_54576 | D00560 |
| 2 | hsa_54576 | D00567 |
| 2 | hsa_54576 | D00652 |
| 2 | hsa_54576 | D01198 |
| 2 | hsa_54576 | D02418 |
| 2 | hsa_54576 | D03829 |
| 2 | hsa_54577 | D00225 |
| 2 | hsa_54577 | D00513 |
| 2 | hsa_54577 | D00709 |
| 2 | hsa_54577 | D00887 |
| 2 | hsa_54577 | D00889 |
| 2 | hsa_54577 | D02110 |
| 2 | hsa_54577 | D03012 |
| 2 | hsa_54577 | D03752 |
| 2 | hsa_54578 | D00294 |
| 2 | hsa_54578 | D00494 |
| 2 | hsa_54578 | D00535 |
| 2 | hsa_54578 | D00998 |
| 2 | hsa_54578 | D03767 |
| 2 | hsa_54578 | D03829 |
| 2 | hsa_54578 | D04292 |
| 2 | hsa_54579 | D00342 |
| 2 | hsa_54579 | D00762 |
| 2 | hsa_54579 | D01900 |
| 2 | hsa_54579 | D02341 |
| 2 | hsa_54579 | D02356 |
| 2 | hsa_54579 | D03798 |
| 2 | hsa_54579 | D03803 |
| 2 | hsa_54579 | D03828 |
| 2 | hsa_54600 | D00158 |
| 2 | hsa_54600 | D00383 |
| 2 | hsa_54600 | D00459 |
| 2 | hsa_54600 | D00488 |
| 2 | hsa_54600 | D00670 |
| 2 | hsa_54600 | D00904 |
| 2 | hsa_54600 | D01198 |
| 2 | hsa_54600 | D01667 |
| 2 | hsa_54600 | D02173 |
| 2 | hsa_54600 | D03714 |
| 2 | hsa_54657 | D01688 |
| 2 | hsa_54657 | D01984 |
| 2 | hsa_54657 | D02441 |
| 2 | hsa_54657 | D02556 |
| 2 | hsa_54657 | D02580 |
| 2 | hsa_54658 | D00168 |
| 2 | hsa_54658 | D00449 |
| 2 | hsa_54659 | D00018 |
| 2 | hsa_54659 | D00070 |
| 2 | hsa_54659 | D00293 |
| 2 | hsa_54659 | D00333 |
| 2 | hsa_54659 | D00377 |
| 2 | hsa_54659 | D00451 |
| 2 | hsa_54659 | D00542 |
| 2 | hsa_54659 | D00624 |
| 2 | hsa_54659 | D00691 |
| 2 | hsa_54659 | D01549 |
| 2 | hsa_54659 | D01665 |
| 2 | hsa_54659 | D02168 |
| 2 | hsa_54659 | D02562 |
| 2 | hsa_54659 | D02671 |
| 2 | hsa_54659 | D02756 |
| 2 | hsa_54677 | D00145 |
| 2 | hsa_54677 | D00359 |
| 2 | hsa_54677 | D00892 |
| 2 | hsa_54677 | D00893 |
| 2 | hsa_54677 | D01709 |
| 2 | hsa_54677 | D03752 |
| 2 | hsa_5470 | D00070 |
| 2 | hsa_5470 | D00160 |
| 2 | hsa_5470 | D00183 |
| 2 | hsa_5470 | D00294 |
| 2 | hsa_5470 | D00387 |
| 2 | hsa_5470 | D00964 |
| 2 | hsa_5470 | D00994 |
| 2 | hsa_5470 | D03115 |
| 2 | hsa_5470 | D03720 |
| 2 | hsa_5475 | D00168 |
| 2 | hsa_5475 | D00217 |
| 2 | hsa_5475 | D00328 |
| 2 | hsa_5475 | D00652 |
| 2 | hsa_5475 | D00884 |
| 2 | hsa_5475 | D01064 |
| 2 | hsa_5475 | D01240 |
| 2 | hsa_5475 | D02355 |
| 2 | hsa_5475 | D03689 |
| 2 | hsa_5476 | D00035 |
| 2 | hsa_5476 | D00184 |
| 2 | hsa_5476 | D00216 |
| 2 | hsa_5476 | D00371 |
| 2 | hsa_5476 | D00550 |
| 2 | hsa_5476 | D03440 |
| 2 | hsa_5476 | D03822 |
| 2 | hsa_5478 | D00131 |
| 2 | hsa_5478 | D00333 |
| 2 | hsa_5478 | D00452 |
| 2 | hsa_5478 | D00762 |
| 2 | hsa_5478 | D01061 |
| 2 | hsa_5478 | D03829 |
| 2 | hsa_5479 | D00005 |
| 2 | hsa_5479 | D00753 |
| 2 | hsa_5479 | D00969 |
| 2 | hsa_5479 | D01364 |
| 2 | hsa_5479 | D02556 |
| 2 | hsa_5479 | D03077 |
| 2 | hsa_5481 | D00035 |
| 2 | hsa_5481 | D00394 |
| 2 | hsa_5481 | D00513 |
| 2 | hsa_5481 | D00550 |
| 2 | hsa_5481 | D00563 |
| 2 | hsa_5481 | D02068 |
| 2 | hsa_5481 | D02110 |
| 2 | hsa_5481 | D02168 |
| 2 | hsa_5481 | D02368 |
| 2 | hsa_5481 | D03738 |
| 2 | hsa_5481 | D04028 |
| 2 | hsa_54878 | D00528 |
| 2 | hsa_54878 | D00579 |
| 2 | hsa_54878 | D01118 |
| 2 | hsa_54878 | D01704 |
| 2 | hsa_54878 | D01765 |
| 2 | hsa_54878 | D02580 |
| 2 | hsa_54878 | D03643 |
| 2 | hsa_54878 | D03767 |
| 2 | hsa_54878 | D03775 |
| 2 | hsa_5494 | D00002 |
| 2 | hsa_5494 | D00041 |
| 2 | hsa_5494 | D00045 |
| 2 | hsa_5494 | D00094 |
| 2 | hsa_5494 | D00103 |
| 2 | hsa_5494 | D01119 |
| 2 | hsa_5494 | D01825 |
| 2 | hsa_5494 | D02321 |
| 2 | hsa_5495 | D00125 |
| 2 | hsa_5495 | D00203 |
| 2 | hsa_5495 | D00371 |
| 2 | hsa_5495 | D00650 |
| 2 | hsa_5495 | D02451 |
| 2 | hsa_5495 | D03734 |
| 2 | hsa_5496 | D00538 |
| 2 | hsa_5496 | D00654 |
| 2 | hsa_5496 | D01981 |
| 2 | hsa_5496 | D02333 |
| 2 | hsa_5496 | D03643 |
| 2 | hsa_5496 | D03756 |
| 2 | hsa_5496 | D03798 |
| 2 | hsa_5499 | D00141 |
| 2 | hsa_5499 | D00274 |
| 2 | hsa_5499 | D00810 |
| 2 | hsa_5499 | D01180 |
| 2 | hsa_5499 | D01718 |
| 2 | hsa_5499 | D01900 |
| 2 | hsa_5499 | D02166 |
| 2 | hsa_5499 | D02350 |
| 2 | hsa_54 | D00416 |
| 2 | hsa_54 | D00829 |
| 2 | hsa_54 | D00904 |
| 2 | hsa_54 | D02580 |
| 2 | hsa_54 | D04025 |
| 2 | hsa_5500 | D00153 |
| 2 | hsa_5500 | D00332 |
| 2 | hsa_5500 | D00394 |
| 2 | hsa_5500 | D00503 |
| 2 | hsa_5500 | D00505 |
| 2 | hsa_5500 | D00528 |
| 2 | hsa_5500 | D00543 |
| 2 | hsa_5500 | D00771 |
| 2 | hsa_5500 | D00960 |
| 2 | hsa_5500 | D01346 |
| 2 | hsa_5500 | D01688 |
| 2 | hsa_5500 | D01718 |
| 2 | hsa_5500 | D02560 |
| 2 | hsa_5500 | D02835 |
| 2 | hsa_5500 | D03767 |
| 2 | hsa_5501 | D00007 |
| 2 | hsa_5501 | D00127 |
| 2 | hsa_5501 | D00141 |
| 2 | hsa_5501 | D00342 |
| 2 | hsa_5501 | D00410 |
| 2 | hsa_5501 | D00579 |
| 2 | hsa_5501 | D01667 |
| 2 | hsa_5501 | D01811 |
| 2 | hsa_5501 | D03736 |
| 2 | hsa_5501 | D03775 |
| 2 | hsa_5501 | D03784 |
| 2 | hsa_5501 | D03787 |
| 2 | hsa_5515 | D00036 |
| 2 | hsa_5515 | D00279 |
| 2 | hsa_5515 | D02229 |
| 2 | hsa_5515 | D03753 |
| 2 | hsa_5515 | D03788 |
| 2 | hsa_5516 | D00315 |
| 2 | hsa_5516 | D01198 |
| 2 | hsa_5516 | D02166 |
| 2 | hsa_5516 | D03670 |
| 2 | hsa_5523 | D00383 |
| 2 | hsa_5523 | D00620 |
| 2 | hsa_5523 | D00653 |
| 2 | hsa_5523 | D01001 |
| 2 | hsa_5523 | D01346 |
| 2 | hsa_5523 | D01888 |
| 2 | hsa_5523 | D02229 |
| 2 | hsa_5523 | D03728 |
| 2 | hsa_5523 | D04197 |
| 2 | hsa_5530 | D00039 |
| 2 | hsa_5530 | D00054 |
| 2 | hsa_5530 | D00065 |
| 2 | hsa_5530 | D00153 |
| 2 | hsa_5530 | D00203 |
| 2 | hsa_5530 | D00274 |
| 2 | hsa_5530 | D00300 |
| 2 | hsa_5530 | D00459 |
| 2 | hsa_5530 | D00501 |
| 2 | hsa_5530 | D00596 |
| 2 | hsa_5530 | D00965 |
| 2 | hsa_55312 | D00298 |
| 2 | hsa_55312 | D00651 |
| 2 | hsa_55312 | D01968 |
| 2 | hsa_55312 | D01981 |
| 2 | hsa_55312 | D02328 |
| 2 | hsa_55312 | D03735 |
| 2 | hsa_5531 | D00198 |
| 2 | hsa_5531 | D00216 |
| 2 | hsa_5531 | D00298 |
| 2 | hsa_5531 | D01196 |
| 2 | hsa_5531 | D01346 |
| 2 | hsa_5531 | D02068 |
| 2 | hsa_5531 | D02173 |
| 2 | hsa_5531 | D02267 |
| 2 | hsa_5531 | D03784 |
| 2 | hsa_5531 | D03805 |
| 2 | hsa_5532 | D00007 |
| 2 | hsa_5532 | D00198 |
| 2 | hsa_5532 | D00225 |
| 2 | hsa_5532 | D00391 |
| 2 | hsa_5532 | D00434 |
| 2 | hsa_5532 | D00622 |
| 2 | hsa_5532 | D00623 |
| 2 | hsa_5532 | D01712 |
| 2 | hsa_5532 | D02321 |
| 2 | hsa_5532 | D02579 |
| 2 | hsa_5532 | D02769 |
| 2 | hsa_5532 | D03752 |
| 2 | hsa_5532 | D03775 |
| 2 | hsa_5533 | D00052 |
| 2 | hsa_5533 | D00196 |
| 2 | hsa_5533 | D00510 |
| 2 | hsa_5533 | D00569 |
| 2 | hsa_5533 | D00691 |
| 2 | hsa_5533 | D00884 |
| 2 | hsa_5533 | D01364 |
| 2 | hsa_5533 | D01397 |
| 2 | hsa_5533 | D01811 |
| 2 | hsa_5533 | D02333 |
| 2 | hsa_5533 | D02579 |
| 2 | hsa_5533 | D03756 |
| 2 | hsa_5533 | D03760 |
| 2 | hsa_55359 | D00027 |
| 2 | hsa_55359 | D00251 |
| 2 | hsa_55359 | D00293 |
| 2 | hsa_55359 | D00503 |
| 2 | hsa_55359 | D00656 |
| 2 | hsa_55359 | D00965 |
| 2 | hsa_55359 | D01842 |
| 2 | hsa_55359 | D02176 |
| 2 | hsa_5536 | D00394 |
| 2 | hsa_5536 | D00577 |
| 2 | hsa_5536 | D01718 |
| 2 | hsa_5537 | D00005 |
| 2 | hsa_5537 | D00027 |
| 2 | hsa_5537 | D00188 |
| 2 | hsa_5537 | D00328 |
| 2 | hsa_5537 | D00496 |
| 2 | hsa_5537 | D01276 |
| 2 | hsa_5537 | D01842 |
| 2 | hsa_5537 | D03722 |
| 2 | hsa_5538 | D00109 |
| 2 | hsa_5538 | D00459 |
| 2 | hsa_5538 | D00538 |
| 2 | hsa_5538 | D00969 |
| 2 | hsa_5538 | D01180 |
| 2 | hsa_5538 | D02267 |
| 2 | hsa_5538 | D02487 |
| 2 | hsa_5538 | D03803 |
| 2 | hsa_5547 | D00227 |
| 2 | hsa_5547 | D00283 |
| 2 | hsa_5547 | D00394 |
| 2 | hsa_5547 | D00416 |
| 2 | hsa_5547 | D01097 |
| 2 | hsa_5547 | D01977 |
| 2 | hsa_5547 | D02556 |
| 2 | hsa_5547 | D02655 |
| 2 | hsa_5547 | D03440 |
| 2 | hsa_5547 | D04024 |
| 2 | hsa_5550 | D00018 |
| 2 | hsa_5550 | D00437 |
| 2 | hsa_5550 | D00505 |
| 2 | hsa_5550 | D00512 |
| 2 | hsa_5550 | D00538 |
| 2 | hsa_5550 | D00584 |
| 2 | hsa_5550 | D02193 |
| 2 | hsa_5550 | D02342 |
| 2 | hsa_55512 | D00300 |
| 2 | hsa_55512 | D00369 |
| 2 | hsa_55512 | D00398 |
| 2 | hsa_55512 | D00543 |
| 2 | hsa_55512 | D03731 |
| 2 | hsa_55512 | D03738 |
| 2 | hsa_5562 | D00103 |
| 2 | hsa_5562 | D00145 |
| 2 | hsa_5562 | D00148 |
| 2 | hsa_5562 | D00416 |
| 2 | hsa_5562 | D00530 |
| 2 | hsa_5562 | D01256 |
| 2 | hsa_5562 | D01370 |
| 2 | hsa_5562 | D02315 |
| 2 | hsa_5562 | D02487 |
| 2 | hsa_55775 | D00103 |
| 2 | hsa_55775 | D00132 |
| 2 | hsa_55775 | D00196 |
| 2 | hsa_55775 | D00274 |
| 2 | hsa_55775 | D00810 |
| 2 | hsa_55775 | D00829 |
| 2 | hsa_55775 | D01915 |
| 2 | hsa_55775 | D03601 |
| 2 | hsa_55775 | D03710 |
| 2 | hsa_55775 | D03823 |
| 2 | hsa_55811 | D00270 |
| 2 | hsa_55811 | D00274 |
| 2 | hsa_55811 | D00387 |
| 2 | hsa_55811 | D00510 |
| 2 | hsa_55811 | D01240 |
| 2 | hsa_55811 | D02308 |
| 2 | hsa_55811 | D03803 |
| 2 | hsa_55811 | D03805 |
| 2 | hsa_558 | D00038 |
| 2 | hsa_558 | D00097 |
| 2 | hsa_558 | D00169 |
| 2 | hsa_558 | D00231 |
| 2 | hsa_558 | D00786 |
| 2 | hsa_558 | D03440 |
| 2 | hsa_558 | D03601 |
| 2 | hsa_558 | D03712 |
| 2 | hsa_558 | D03743 |
| 2 | hsa_558 | D04966 |
| 2 | hsa_558 | D05407 |
| 2 | hsa_55902 | D00035 |
| 2 | hsa_55902 | D00317 |
| 2 | hsa_55902 | D00455 |
| 2 | hsa_55902 | D01885 |
| 2 | hsa_55902 | D02110 |
| 2 | hsa_55902 | D02560 |
| 2 | hsa_55902 | D03218 |
| 2 | hsa_55902 | D03670 |
| 2 | hsa_55902 | D03899 |
| 2 | hsa_5594 | D00039 |
| 2 | hsa_5594 | D00451 |
| 2 | hsa_5594 | D00903 |
| 2 | hsa_5594 | D01256 |
| 2 | hsa_5594 | D01582 |
| 2 | hsa_5594 | D02566 |
| 2 | hsa_5595 | D00733 |
| 2 | hsa_5595 | D02229 |
| 2 | hsa_5595 | D03728 |
| 2 | hsa_5595 | D03776 |
| 2 | hsa_5595 | D03822 |
| 2 | hsa_5596 | D00185 |
| 2 | hsa_5596 | D00203 |
| 2 | hsa_5596 | D00270 |
| 2 | hsa_5596 | D00364 |
| 2 | hsa_5596 | D00528 |
| 2 | hsa_5596 | D00670 |
| 2 | hsa_5596 | D00965 |
| 2 | hsa_5596 | D01240 |
| 2 | hsa_5596 | D01364 |
| 2 | hsa_5596 | D03433 |
| 2 | hsa_5597 | D00148 |
| 2 | hsa_5597 | D01001 |
| 2 | hsa_5597 | D01275 |
| 2 | hsa_5597 | D01549 |
| 2 | hsa_5597 | D03733 |
| 2 | hsa_5597 | D03753 |
| 2 | hsa_5598 | D00054 |
| 2 | hsa_5598 | D00153 |
| 2 | hsa_5598 | D00425 |
| 2 | hsa_5598 | D00998 |
| 2 | hsa_5598 | D02258 |
| 2 | hsa_5598 | D02729 |
| 2 | hsa_5598 | D03738 |
| 2 | hsa_5598 | D03778 |
| 2 | hsa_5599 | D00050 |
| 2 | hsa_5599 | D00109 |
| 2 | hsa_5599 | D00294 |
| 2 | hsa_5599 | D00623 |
| 2 | hsa_5599 | D00624 |
| 2 | hsa_55 | D00224 |
| 2 | hsa_55 | D00550 |
| 2 | hsa_55 | D00810 |
| 2 | hsa_55 | D00892 |
| 2 | hsa_55 | D01765 |
| 2 | hsa_55 | D03823 |
| 2 | hsa_5600 | D00052 |
| 2 | hsa_5600 | D00332 |
| 2 | hsa_5600 | D00364 |
| 2 | hsa_5600 | D00455 |
| 2 | hsa_5600 | D00543 |
| 2 | hsa_5600 | D00968 |
| 2 | hsa_5600 | D01582 |
| 2 | hsa_5600 | D02168 |
| 2 | hsa_5600 | D02441 |
| 2 | hsa_5601 | D00158 |
| 2 | hsa_5601 | D00322 |
| 2 | hsa_5601 | D00330 |
| 2 | hsa_5601 | D00563 |
| 2 | hsa_5601 | D00902 |
| 2 | hsa_5601 | D02110 |
| 2 | hsa_5601 | D02229 |
| 2 | hsa_5601 | D03077 |
| 2 | hsa_5601 | D03712 |
| 2 | hsa_5602 | D00359 |
| 2 | hsa_5602 | D00418 |
| 2 | hsa_5602 | D00518 |
| 2 | hsa_5602 | D00652 |
| 2 | hsa_5602 | D01136 |
| 2 | hsa_5602 | D01397 |
| 2 | hsa_5602 | D01709 |
| 2 | hsa_5602 | D02368 |
| 2 | hsa_5602 | D02655 |
| 2 | hsa_5603 | D00362 |
| 2 | hsa_5603 | D00416 |
| 2 | hsa_5603 | D00455 |
| 2 | hsa_5603 | D00536 |
| 2 | hsa_5603 | D00762 |
| 2 | hsa_5603 | D01547 |
| 2 | hsa_5603 | D01582 |
| 2 | hsa_5603 | D02655 |
| 2 | hsa_5603 | D02731 |
| 2 | hsa_5603 | D04028 |
| 2 | hsa_5604 | D00126 |
| 2 | hsa_5604 | D00293 |
| 2 | hsa_5604 | D00394 |
| 2 | hsa_5604 | D00596 |
| 2 | hsa_5604 | D00947 |
| 2 | hsa_5604 | D01001 |
| 2 | hsa_5604 | D01370 |
| 2 | hsa_5604 | D02580 |
| 2 | hsa_5604 | D03736 |
| 2 | hsa_5605 | D00994 |
| 2 | hsa_5605 | D02341 |
| 2 | hsa_5606 | D00126 |
| 2 | hsa_5606 | D00136 |
| 2 | hsa_5606 | D00270 |
| 2 | hsa_5606 | D00625 |
| 2 | hsa_5606 | D00656 |
| 2 | hsa_5606 | D02304 |
| 2 | hsa_5606 | D03758 |
| 2 | hsa_5607 | D00362 |
| 2 | hsa_5607 | D00454 |
| 2 | hsa_5607 | D00563 |
| 2 | hsa_5607 | D00584 |
| 2 | hsa_5607 | D00810 |
| 2 | hsa_5607 | D01918 |
| 2 | hsa_5607 | D02655 |
| 2 | hsa_5607 | D04029 |
| 2 | hsa_5608 | D00038 |
| 2 | hsa_5608 | D00139 |
| 2 | hsa_5608 | D00231 |
| 2 | hsa_5608 | D00401 |
| 2 | hsa_5608 | D00423 |
| 2 | hsa_5608 | D00505 |
| 2 | hsa_5608 | D00593 |
| 2 | hsa_5608 | D00781 |
| 2 | hsa_5608 | D00968 |
| 2 | hsa_5608 | D02173 |
| 2 | hsa_5608 | D03781 |
| 2 | hsa_5624 | D00065 |
| 2 | hsa_5624 | D00131 |
| 2 | hsa_5624 | D00196 |
| 2 | hsa_5624 | D00425 |
| 2 | hsa_5624 | D00624 |
| 2 | hsa_5624 | D00670 |
| 2 | hsa_5624 | D00810 |
| 2 | hsa_5624 | D01715 |
| 2 | hsa_5624 | D04966 |
| 2 | hsa_5625 | D00126 |
| 2 | hsa_5625 | D00222 |
| 2 | hsa_5625 | D00325 |
| 2 | hsa_5625 | D00454 |
| 2 | hsa_5625 | D00488 |
| 2 | hsa_5625 | D00549 |
| 2 | hsa_5625 | D00670 |
| 2 | hsa_5625 | D01133 |
| 2 | hsa_5625 | D01712 |
| 2 | hsa_5625 | D02017 |
| 2 | hsa_5625 | D02168 |
| 2 | hsa_5625 | D03806 |
| 2 | hsa_5625 | D04966 |
| 2 | hsa_5645 | D00401 |
| 2 | hsa_5645 | D00410 |
| 2 | hsa_5645 | D00994 |
| 2 | hsa_5645 | D02304 |
| 2 | hsa_5645 | D02323 |
| 2 | hsa_5645 | D02341 |
| 2 | hsa_5645 | D03778 |
| 2 | hsa_5646 | D01064 |
| 2 | hsa_5646 | D01665 |
| 2 | hsa_5646 | D02564 |
| 2 | hsa_5650 | D00203 |
| 2 | hsa_5650 | D00448 |
| 2 | hsa_5650 | D00449 |
| 2 | hsa_5650 | D00455 |
| 2 | hsa_5650 | D00505 |
| 2 | hsa_5650 | D00519 |
| 2 | hsa_5650 | D00733 |
| 2 | hsa_5650 | D01275 |
| 2 | hsa_5650 | D02323 |
| 2 | hsa_5650 | D02368 |
| 2 | hsa_5650 | D03772 |
| 2 | hsa_5651 | D00027 |
| 2 | hsa_5651 | D00449 |
| 2 | hsa_5651 | D00513 |
| 2 | hsa_5651 | D00516 |
| 2 | hsa_5651 | D00624 |
| 2 | hsa_5651 | D00995 |
| 2 | hsa_5651 | D03807 |
| 2 | hsa_5651 | D06238 |
| 2 | hsa_5657 | D00118 |
| 2 | hsa_5657 | D00315 |
| 2 | hsa_5657 | D00521 |
| 2 | hsa_5657 | D00593 |
| 2 | hsa_5657 | D00785 |
| 2 | hsa_5657 | D00885 |
| 2 | hsa_5657 | D01133 |
| 2 | hsa_5657 | D01364 |
| 2 | hsa_5657 | D01842 |
| 2 | hsa_5657 | D01888 |
| 2 | hsa_5657 | D02258 |
| 2 | hsa_5657 | D02566 |
| 2 | hsa_5657 | D03741 |
| 2 | hsa_5657 | D05341 |
| 2 | hsa_56922 | D00050 |
| 2 | hsa_56922 | D00155 |
| 2 | hsa_56922 | D00203 |
| 2 | hsa_56922 | D00455 |
| 2 | hsa_56922 | D00563 |
| 2 | hsa_56922 | D00902 |
| 2 | hsa_56922 | D01064 |
| 2 | hsa_56922 | D01718 |
| 2 | hsa_56922 | D02342 |
| 2 | hsa_56922 | D02355 |
| 2 | hsa_56922 | D02556 |
| 2 | hsa_56922 | D02698 |
| 2 | hsa_57016 | D00882 |
| 2 | hsa_57016 | D00965 |
| 2 | hsa_57016 | D01069 |
| 2 | hsa_57016 | D01984 |
| 2 | hsa_57016 | D03751 |
| 2 | hsa_57176 | D00054 |
| 2 | hsa_57176 | D00516 |
| 2 | hsa_57176 | D00653 |
| 2 | hsa_57176 | D01069 |
| 2 | hsa_57176 | D01211 |
| 2 | hsa_57176 | D01709 |
| 2 | hsa_57176 | D02110 |
| 2 | hsa_57176 | D02671 |
| 2 | hsa_57176 | D03753 |
| 2 | hsa_5740 | D00130 |
| 2 | hsa_5740 | D00434 |
| 2 | hsa_5740 | D00449 |
| 2 | hsa_5740 | D01513 |
| 2 | hsa_5740 | D02290 |
| 2 | hsa_5740 | D02729 |
| 2 | hsa_5740 | D03753 |
| 2 | hsa_5740 | D03765 |
| 2 | hsa_5740 | D04024 |
| 2 | hsa_5740 | D04025 |
| 2 | hsa_5742 | D00018 |
| 2 | hsa_5742 | D00065 |
| 2 | hsa_5742 | D00139 |
| 2 | hsa_5742 | D00187 |
| 2 | hsa_5742 | D00222 |
| 2 | hsa_5742 | D00562 |
| 2 | hsa_5742 | D00621 |
| 2 | hsa_5742 | D01984 |
| 2 | hsa_5742 | D05407 |
| 2 | hsa_5743 | D00041 |
| 2 | hsa_5743 | D00094 |
| 2 | hsa_5743 | D00535 |
| 2 | hsa_5743 | D00546 |
| 2 | hsa_5743 | D00630 |
| 2 | hsa_5743 | D01180 |
| 2 | hsa_5743 | D01981 |
| 2 | hsa_5743 | D02323 |
| 2 | hsa_5743 | D02562 |
| 2 | hsa_5743 | D04025 |
| 2 | hsa_5747 | D00567 |
| 2 | hsa_5747 | D02008 |
| 2 | hsa_5754 | D00383 |
| 2 | hsa_5754 | D00968 |
| 2 | hsa_5754 | D01513 |
| 2 | hsa_5754 | D02487 |
| 2 | hsa_5754 | D02560 |
| 2 | hsa_57665 | D00132 |
| 2 | hsa_57665 | D00283 |
| 2 | hsa_57665 | D00325 |
| 2 | hsa_57665 | D00394 |
| 2 | hsa_57665 | D00425 |
| 2 | hsa_57665 | D00494 |
| 2 | hsa_57665 | D00623 |
| 2 | hsa_57665 | D01332 |
| 2 | hsa_57665 | D02068 |
| 2 | hsa_57665 | D02560 |
| 2 | hsa_57665 | D03012 |
| 2 | hsa_57665 | D03765 |
| 2 | hsa_57665 | D03769 |
| 2 | hsa_58190 | D00007 |
| 2 | hsa_58190 | D00521 |
| 2 | hsa_58190 | D00900 |
| 2 | hsa_58190 | D01228 |
| 2 | hsa_58190 | D01968 |
| 2 | hsa_58190 | D03751 |
| 2 | hsa_5831 | D00120 |
| 2 | hsa_5831 | D00298 |
| 2 | hsa_5831 | D00448 |
| 2 | hsa_5831 | D00455 |
| 2 | hsa_5831 | D01228 |
| 2 | hsa_5831 | D01718 |
| 2 | hsa_5831 | D01966 |
| 2 | hsa_5834 | D00036 |
| 2 | hsa_5834 | D00208 |
| 2 | hsa_5834 | D00219 |
| 2 | hsa_5834 | D00410 |
| 2 | hsa_5834 | D00513 |
| 2 | hsa_5834 | D00562 |
| 2 | hsa_5834 | D00771 |
| 2 | hsa_5834 | D00829 |
| 2 | hsa_5834 | D00900 |
| 2 | hsa_5834 | D01475 |
| 2 | hsa_5834 | D02558 |
| 2 | hsa_5836 | D00094 |
| 2 | hsa_5836 | D00160 |
| 2 | hsa_5836 | D00330 |
| 2 | hsa_5836 | D00371 |
| 2 | hsa_5836 | D00550 |
| 2 | hsa_5836 | D00622 |
| 2 | hsa_5836 | D00813 |
| 2 | hsa_5836 | D01332 |
| 2 | hsa_5836 | D02194 |
| 2 | hsa_5836 | D02566 |
| 2 | hsa_5837 | D00094 |
| 2 | hsa_5837 | D00452 |
| 2 | hsa_5837 | D00964 |
| 2 | hsa_5837 | D01027 |
| 2 | hsa_5837 | D01425 |
| 2 | hsa_5837 | D02335 |
| 2 | hsa_5837 | D03722 |
| 2 | hsa_5837 | D03816 |
| 2 | hsa_5837 | D03828 |
| 2 | hsa_5837 | D03899 |
| 2 | hsa_5860 | D00052 |
| 2 | hsa_5860 | D00294 |
| 2 | hsa_5860 | D00410 |
| 2 | hsa_5860 | D00448 |
| 2 | hsa_5860 | D01097 |
| 2 | hsa_5860 | D01582 |
| 2 | hsa_5860 | D01977 |
| 2 | hsa_5860 | D02214 |
| 2 | hsa_5860 | D02290 |
| 2 | hsa_5860 | D03741 |
| 2 | hsa_586 | D00274 |
| 2 | hsa_586 | D01276 |
| 2 | hsa_586 | D01973 |
| 2 | hsa_586 | D04031 |
| 2 | hsa_587 | D00002 |
| 2 | hsa_587 | D00029 |
| 2 | hsa_587 | D00252 |
| 2 | hsa_587 | D00294 |
| 2 | hsa_587 | D00410 |
| 2 | hsa_587 | D00535 |
| 2 | hsa_587 | D00650 |
| 2 | hsa_587 | D00786 |
| 2 | hsa_587 | D02176 |
| 2 | hsa_590 | D00050 |
| 2 | hsa_590 | D00452 |
| 2 | hsa_590 | D00535 |
| 2 | hsa_590 | D01027 |
| 2 | hsa_590 | D01918 |
| 2 | hsa_590 | D02671 |
| 2 | hsa_590 | D03734 |
| 2 | hsa_590 | D03784 |
| 2 | hsa_5972 | D00380 |
| 2 | hsa_5972 | D00463 |
| 2 | hsa_5972 | D01915 |
| 2 | hsa_5972 | D01966 |
| 2 | hsa_5972 | D02110 |
| 2 | hsa_5972 | D02166 |
| 2 | hsa_5972 | D03758 |
| 2 | hsa_5972 | D05341 |
| 2 | hsa_5979 | D00153 |
| 2 | hsa_5979 | D00198 |
| 2 | hsa_5979 | D00560 |
| 2 | hsa_5979 | D00970 |
| 2 | hsa_5979 | D01690 |
| 2 | hsa_5979 | D01712 |
| 2 | hsa_5979 | D01974 |
| 2 | hsa_5979 | D02321 |
| 2 | hsa_5979 | D02323 |
| 2 | hsa_5979 | D03738 |
| 2 | hsa_6098 | D00216 |
| 2 | hsa_6098 | D00513 |
| 2 | hsa_6098 | D00542 |
| 2 | hsa_6098 | D00969 |
| 2 | hsa_6098 | D01061 |
| 2 | hsa_6098 | D02229 |
| 2 | hsa_6098 | D02341 |
| 2 | hsa_6098 | D02579 |
| 2 | hsa_6098 | D03753 |
| 2 | hsa_6098 | D03769 |
| 2 | hsa_6098 | D04029 |
| 2 | hsa_6098 | D04983 |
| 2 | hsa_613 | D00039 |
| 2 | hsa_613 | D00401 |
| 2 | hsa_613 | D00544 |
| 2 | hsa_613 | D00625 |
| 2 | hsa_613 | D00892 |
| 2 | hsa_613 | D01136 |
| 2 | hsa_613 | D01425 |
| 2 | hsa_613 | D03829 |
| 2 | hsa_613 | D04197 |
| 2 | hsa_6240 | D00218 |
| 2 | hsa_6240 | D00543 |
| 2 | hsa_6240 | D01844 |
| 2 | hsa_6240 | D01866 |
| 2 | hsa_6240 | D01977 |
| 2 | hsa_6240 | D02194 |
| 2 | hsa_6241 | D00449 |
| 2 | hsa_6241 | D00560 |
| 2 | hsa_6241 | D00630 |
| 2 | hsa_6241 | D00733 |
| 2 | hsa_6241 | D01688 |
| 2 | hsa_6241 | D01825 |
| 2 | hsa_6241 | D01907 |
| 2 | hsa_6241 | D01977 |
| 2 | hsa_6241 | D02342 |
| 2 | hsa_6241 | D02355 |
| 2 | hsa_6241 | D03716 |
| 2 | hsa_6259 | D00132 |
| 2 | hsa_6259 | D00487 |
| 2 | hsa_6259 | D00562 |
| 2 | hsa_6259 | D00994 |
| 2 | hsa_6259 | D01228 |
| 2 | hsa_6259 | D03012 |
| 2 | hsa_6259 | D03689 |
| 2 | hsa_6259 | D03807 |
| 2 | hsa_6300 | D00038 |
| 2 | hsa_6300 | D00377 |
| 2 | hsa_6300 | D01690 |
| 2 | hsa_6300 | D01974 |
| 2 | hsa_6300 | D03034 |
| 2 | hsa_63036 | D00130 |
| 2 | hsa_63036 | D00142 |
| 2 | hsa_63036 | D00227 |
| 2 | hsa_63036 | D00401 |
| 2 | hsa_63036 | D00416 |
| 2 | hsa_63036 | D00596 |
| 2 | hsa_63036 | D01885 |
| 2 | hsa_63036 | D02451 |
| 2 | hsa_63036 | D02709 |
| 2 | hsa_63036 | D03720 |
| 2 | hsa_63036 | D04197 |
| 2 | hsa_635 | D00394 |
| 2 | hsa_635 | D00494 |
| 2 | hsa_635 | D00621 |
| 2 | hsa_635 | D00994 |
| 2 | hsa_635 | D01240 |
| 2 | hsa_635 | D02008 |
| 2 | hsa_635 | D03733 |
| 2 | hsa_635 | D03805 |
| 2 | hsa_635 | D04966 |
| 2 | hsa_63904 | D00126 |
| 2 | hsa_63904 | D00577 |
| 2 | hsa_63904 | D00622 |
| 2 | hsa_63904 | D00960 |
| 2 | hsa_64087 | D00039 |
| 2 | hsa_64087 | D00118 |
| 2 | hsa_64087 | D00155 |
| 2 | hsa_64087 | D00188 |
| 2 | hsa_64087 | D00330 |
| 2 | hsa_64087 | D00542 |
| 2 | hsa_64087 | D00550 |
| 2 | hsa_64087 | D00655 |
| 2 | hsa_64087 | D00733 |
| 2 | hsa_64087 | D00882 |
| 2 | hsa_64087 | D01061 |
| 2 | hsa_64087 | D02173 |
| 2 | hsa_64087 | D02835 |
| 2 | hsa_64087 | D03218 |
| 2 | hsa_64087 | D03643 |
| 2 | hsa_64087 | D03731 |
| 2 | hsa_64087 | D03763 |
| 2 | hsa_6416 | D00184 |
| 2 | hsa_6416 | D00294 |
| 2 | hsa_6416 | D00322 |
| 2 | hsa_6416 | D00359 |
| 2 | hsa_6416 | D00369 |
| 2 | hsa_6416 | D00488 |
| 2 | hsa_6416 | D00569 |
| 2 | hsa_6416 | D00593 |
| 2 | hsa_6416 | D00703 |
| 2 | hsa_6416 | D00900 |
| 2 | hsa_6416 | D00964 |
| 2 | hsa_6416 | D00970 |
| 2 | hsa_6416 | D01196 |
| 2 | hsa_6416 | D03689 |
| 2 | hsa_6416 | D03767 |
| 2 | hsa_64499 | D00035 |
| 2 | hsa_64499 | D00691 |
| 2 | hsa_64499 | D01136 |
| 2 | hsa_64499 | D06238 |
| 2 | hsa_645 | D00131 |
| 2 | hsa_645 | D00501 |
| 2 | hsa_645 | D00544 |
| 2 | hsa_645 | D01973 |
| 2 | hsa_645 | D03735 |
| 2 | hsa_645 | D03775 |
| 2 | hsa_64600 | D00018 |
| 2 | hsa_64600 | D00094 |
| 2 | hsa_64600 | D00142 |
| 2 | hsa_64600 | D00322 |
| 2 | hsa_64600 | D00512 |
| 2 | hsa_64600 | D00562 |
| 2 | hsa_64600 | D00829 |
| 2 | hsa_64600 | D00887 |
| 2 | hsa_64600 | D02115 |
| 2 | hsa_64600 | D02304 |
| 2 | hsa_64600 | D02368 |
| 2 | hsa_64600 | D03731 |
| 2 | hsa_64600 | D03752 |
| 2 | hsa_64802 | D00136 |
| 2 | hsa_64802 | D00421 |
| 2 | hsa_64802 | D00887 |
| 2 | hsa_64802 | D01866 |
| 2 | hsa_64802 | D02561 |
| 2 | hsa_64802 | D02581 |
| 2 | hsa_64802 | D02655 |
| 2 | hsa_64816 | D00231 |
| 2 | hsa_64816 | D01718 |
| 2 | hsa_64816 | D02323 |
| 2 | hsa_64850 | D00141 |
| 2 | hsa_64850 | D00169 |
| 2 | hsa_64850 | D00198 |
| 2 | hsa_64850 | D00231 |
| 2 | hsa_64850 | D00494 |
| 2 | hsa_64850 | D00703 |
| 2 | hsa_64850 | D01844 |
| 2 | hsa_64850 | D01907 |
| 2 | hsa_64850 | D03440 |
| 2 | hsa_64850 | D03689 |
| 2 | hsa_64902 | D00049 |
| 2 | hsa_64902 | D00120 |
| 2 | hsa_64902 | D00325 |
| 2 | hsa_64902 | D00377 |
| 2 | hsa_64902 | D00488 |
| 2 | hsa_64902 | D00785 |
| 2 | hsa_64902 | D00968 |
| 2 | hsa_64902 | D00969 |
| 2 | hsa_64902 | D01027 |
| 2 | hsa_64902 | D01918 |
| 2 | hsa_64902 | D02333 |
| 2 | hsa_64902 | D02341 |
| 2 | hsa_64902 | D04983 |
| 2 | hsa_657 | D00330 |
| 2 | hsa_657 | D01578 |
| 2 | hsa_657 | D03784 |
| 2 | hsa_658 | D00652 |
| 2 | hsa_658 | D00658 |
| 2 | hsa_658 | D03433 |
| 2 | hsa_658 | D03763 |
| 2 | hsa_658 | D03803 |
| 2 | hsa_6609 | D00029 |
| 2 | hsa_6609 | D00126 |
| 2 | hsa_6609 | D00130 |
| 2 | hsa_6609 | D00217 |
| 2 | hsa_6609 | D00518 |
| 2 | hsa_6609 | D00752 |
| 2 | hsa_6609 | D01885 |
| 2 | hsa_6609 | D01915 |
| 2 | hsa_6609 | D02350 |
| 2 | hsa_6609 | D03753 |
| 2 | hsa_6609 | D03756 |
| 2 | hsa_660 | D00359 |
| 2 | hsa_660 | D00503 |
| 2 | hsa_660 | D00563 |
| 2 | hsa_660 | D01900 |
| 2 | hsa_660 | D02193 |
| 2 | hsa_660 | D03689 |
| 2 | hsa_6610 | D00109 |
| 2 | hsa_6610 | D00369 |
| 2 | hsa_6610 | D00475 |
| 2 | hsa_6610 | D00503 |
| 2 | hsa_6610 | D00518 |
| 2 | hsa_6610 | D01071 |
| 2 | hsa_6610 | D01325 |
| 2 | hsa_6610 | D01825 |
| 2 | hsa_6646 | D00139 |
| 2 | hsa_6646 | D00621 |
| 2 | hsa_6646 | D00670 |
| 2 | hsa_6646 | D00893 |
| 2 | hsa_6646 | D00998 |
| 2 | hsa_6646 | D02267 |
| 2 | hsa_6652 | D00018 |
| 2 | hsa_6652 | D00169 |
| 2 | hsa_6652 | D00203 |
| 2 | hsa_6652 | D00394 |
| 2 | hsa_6652 | D00658 |
| 2 | hsa_6652 | D01211 |
| 2 | hsa_6652 | D01432 |
| 2 | hsa_6652 | D01866 |
| 2 | hsa_6652 | D01918 |
| 2 | hsa_6652 | D02581 |
| 2 | hsa_6652 | D04966 |
| 2 | hsa_670 | D00148 |
| 2 | hsa_670 | D00183 |
| 2 | hsa_670 | D00300 |
| 2 | hsa_670 | D00786 |
| 2 | hsa_670 | D01264 |
| 2 | hsa_670 | D01712 |
| 2 | hsa_6713 | D00097 |
| 2 | hsa_6713 | D00160 |
| 2 | hsa_6713 | D00579 |
| 2 | hsa_6713 | D00703 |
| 2 | hsa_6713 | D01256 |
| 2 | hsa_6713 | D01425 |
| 2 | hsa_6713 | D01475 |
| 2 | hsa_6713 | D02008 |
| 2 | hsa_6713 | D02333 |
| 2 | hsa_6713 | D03350 |
| 2 | hsa_6714 | D00132 |
| 2 | hsa_6714 | D00421 |
| 2 | hsa_6714 | D00709 |
| 2 | hsa_6714 | D02308 |
| 2 | hsa_6714 | D04966 |
| 2 | hsa_6716 | D00324 |
| 2 | hsa_6716 | D03767 |
| 2 | hsa_6716 | D04024 |
| 2 | hsa_6725 | D01918 |
| 2 | hsa_6725 | D02698 |
| 2 | hsa_6725 | D03760 |
| 2 | hsa_6725 | D03763 |
| 2 | hsa_6725 | D03765 |
| 2 | hsa_6725 | D03816 |
| 2 | hsa_6725 | D03882 |
| 2 | hsa_6725 | D04024 |
| 2 | hsa_6768 | D00294 |
| 2 | hsa_6768 | D00387 |
| 2 | hsa_6768 | D00651 |
| 2 | hsa_6768 | D00781 |
| 2 | hsa_6768 | D00964 |
| 2 | hsa_6768 | D01582 |
| 2 | hsa_6768 | D01828 |
| 2 | hsa_6768 | D03077 |
| 2 | hsa_6768 | D03767 |
| 2 | hsa_6799 | D00148 |
| 2 | hsa_6799 | D00342 |
| 2 | hsa_6799 | D00544 |
| 2 | hsa_6799 | D00547 |
| 2 | hsa_6799 | D00574 |
| 2 | hsa_6799 | D00651 |
| 2 | hsa_6799 | D01133 |
| 2 | hsa_6799 | D02115 |
| 2 | hsa_6799 | D02335 |
| 2 | hsa_6799 | D02341 |
| 2 | hsa_6799 | D03816 |
| 2 | hsa_683 | D00317 |
| 2 | hsa_683 | D00475 |
| 2 | hsa_683 | D00884 |
| 2 | hsa_683 | D00970 |
| 2 | hsa_683 | D01885 |
| 2 | hsa_683 | D04197 |
| 2 | hsa_686 | D00070 |
| 2 | hsa_686 | D00120 |
| 2 | hsa_686 | D00148 |
| 2 | hsa_686 | D00285 |
| 2 | hsa_686 | D00452 |
| 2 | hsa_686 | D00545 |
| 2 | hsa_686 | D00650 |
| 2 | hsa_686 | D00709 |
| 2 | hsa_686 | D01027 |
| 2 | hsa_686 | D01966 |
| 2 | hsa_686 | D02008 |
| 2 | hsa_686 | D03670 |
| 2 | hsa_686 | D03689 |
| 2 | hsa_6897 | D00049 |
| 2 | hsa_6897 | D00065 |
| 2 | hsa_6897 | D00139 |
| 2 | hsa_6897 | D00148 |
| 2 | hsa_6897 | D00208 |
| 2 | hsa_6897 | D00416 |
| 2 | hsa_6897 | D00510 |
| 2 | hsa_6897 | D00550 |
| 2 | hsa_6897 | D00667 |
| 2 | hsa_6897 | D00726 |
| 2 | hsa_6897 | D00785 |
| 2 | hsa_6897 | D01183 |
| 2 | hsa_6897 | D01196 |
| 2 | hsa_6897 | D02368 |
| 2 | hsa_6897 | D02580 |
| 2 | hsa_6897 | D03756 |
| 2 | hsa_6897 | D03763 |
| 2 | hsa_6898 | D00394 |
| 2 | hsa_6898 | D00569 |
| 2 | hsa_6898 | D00652 |
| 2 | hsa_6898 | D00654 |
| 2 | hsa_6898 | D00762 |
| 2 | hsa_6898 | D00904 |
| 2 | hsa_6898 | D01183 |
| 2 | hsa_6898 | D01900 |
| 2 | hsa_6898 | D02304 |
| 2 | hsa_6898 | D02375 |
| 2 | hsa_6898 | D03731 |
| 2 | hsa_6898 | D03772 |
| 2 | hsa_6898 | D03823 |
| 2 | hsa_6898 | D03882 |
| 2 | hsa_695 | D00369 |
| 2 | hsa_695 | D00425 |
| 2 | hsa_695 | D00547 |
| 2 | hsa_695 | D00650 |
| 2 | hsa_695 | D00781 |
| 2 | hsa_695 | D01183 |
| 2 | hsa_695 | D01667 |
| 2 | hsa_695 | D01715 |
| 2 | hsa_695 | D02561 |
| 2 | hsa_695 | D03763 |
| 2 | hsa_7006 | D00518 |
| 2 | hsa_7006 | D01840 |
| 2 | hsa_7006 | D01981 |
| 2 | hsa_7006 | D02176 |
| 2 | hsa_7006 | D02333 |
| 2 | hsa_7006 | D03773 |
| 2 | hsa_7010 | D00002 |
| 2 | hsa_7010 | D00032 |
| 2 | hsa_7010 | D00293 |
| 2 | hsa_7010 | D00528 |
| 2 | hsa_7010 | D00753 |
| 2 | hsa_7010 | D00885 |
| 2 | hsa_7010 | D00902 |
| 2 | hsa_7010 | D00903 |
| 2 | hsa_7015 | D00018 |
| 2 | hsa_7015 | D00107 |
| 2 | hsa_7015 | D00252 |
| 2 | hsa_7015 | D00330 |
| 2 | hsa_7015 | D00423 |
| 2 | hsa_7015 | D00562 |
| 2 | hsa_7015 | D00829 |
| 2 | hsa_7015 | D01264 |
| 2 | hsa_7015 | D02176 |
| 2 | hsa_7015 | D02655 |
| 2 | hsa_7015 | D02835 |
| 2 | hsa_7015 | D03734 |
| 2 | hsa_7015 | D03773 |
| 2 | hsa_7046 | D00094 |
| 2 | hsa_7046 | D00330 |
| 2 | hsa_7046 | D00549 |
| 2 | hsa_7046 | D00567 |
| 2 | hsa_7046 | D00884 |
| 2 | hsa_7046 | D00889 |
| 2 | hsa_7046 | D03803 |
| 2 | hsa_7054 | D00394 |
| 2 | hsa_7054 | D00434 |
| 2 | hsa_7054 | D01981 |
| 2 | hsa_7054 | D02559 |
| 2 | hsa_7054 | D04197 |
| 2 | hsa_7075 | D00231 |
| 2 | hsa_7075 | D00371 |
| 2 | hsa_7075 | D00546 |
| 2 | hsa_7075 | D00810 |
| 2 | hsa_7075 | D01513 |
| 2 | hsa_7075 | D01665 |
| 2 | hsa_7075 | D02115 |
| 2 | hsa_7075 | D02173 |
| 2 | hsa_7075 | D02451 |
| 2 | hsa_7083 | D00513 |
| 2 | hsa_7083 | D00904 |
| 2 | hsa_7083 | D03781 |
| 2 | hsa_7084 | D00052 |
| 2 | hsa_7084 | D00125 |
| 2 | hsa_7084 | D00160 |
| 2 | hsa_7084 | D00322 |
| 2 | hsa_7084 | D00324 |
| 2 | hsa_7084 | D00418 |
| 2 | hsa_7084 | D00463 |
| 2 | hsa_7084 | D00887 |
| 2 | hsa_7084 | D01118 |
| 2 | hsa_7084 | D01915 |
| 2 | hsa_7084 | D02110 |
| 2 | hsa_7084 | D06238 |
| 2 | hsa_7150 | D00039 |
| 2 | hsa_7150 | D00107 |
| 2 | hsa_7150 | D00225 |
| 2 | hsa_7150 | D00227 |
| 2 | hsa_7150 | D00547 |
| 2 | hsa_7150 | D00653 |
| 2 | hsa_7150 | D01256 |
| 2 | hsa_7150 | D01811 |
| 2 | hsa_7150 | D01828 |
| 2 | hsa_7150 | D01974 |
| 2 | hsa_7150 | D02355 |
| 2 | hsa_7150 | D03601 |
| 2 | hsa_7150 | D04292 |
| 2 | hsa_7153 | D00037 |
| 2 | hsa_7153 | D00132 |
| 2 | hsa_7153 | D00279 |
| 2 | hsa_7153 | D00332 |
| 2 | hsa_7153 | D00653 |
| 2 | hsa_7153 | D02835 |
| 2 | hsa_7153 | D03773 |
| 2 | hsa_7155 | D00227 |
| 2 | hsa_7155 | D00625 |
| 2 | hsa_7155 | D00703 |
| 2 | hsa_7155 | D00963 |
| 2 | hsa_7155 | D01071 |
| 2 | hsa_7155 | D01180 |
| 2 | hsa_7155 | D01709 |
| 2 | hsa_7155 | D01842 |
| 2 | hsa_7155 | D01984 |
| 2 | hsa_7155 | D02581 |
| 2 | hsa_7156 | D00109 |
| 2 | hsa_7156 | D00503 |
| 2 | hsa_7156 | D00620 |
| 2 | hsa_7156 | D00726 |
| 2 | hsa_7156 | D00884 |
| 2 | hsa_7156 | D02580 |
| 2 | hsa_7172 | D00652 |
| 2 | hsa_7172 | D00786 |
| 2 | hsa_7172 | D01981 |
| 2 | hsa_7172 | D02356 |
| 2 | hsa_7172 | D03760 |
| 2 | hsa_7173 | D00332 |
| 2 | hsa_7173 | D00963 |
| 2 | hsa_7173 | D01133 |
| 2 | hsa_7173 | D01547 |
| 2 | hsa_7173 | D01811 |
| 2 | hsa_7173 | D01981 |
| 2 | hsa_7173 | D03798 |
| 2 | hsa_7174 | D00065 |
| 2 | hsa_7174 | D00208 |
| 2 | hsa_7174 | D00251 |
| 2 | hsa_7174 | D00380 |
| 2 | hsa_7174 | D00394 |
| 2 | hsa_7174 | D00658 |
| 2 | hsa_7174 | D00885 |
| 2 | hsa_7174 | D01918 |
| 2 | hsa_7174 | D01966 |
| 2 | hsa_7294 | D00188 |
| 2 | hsa_7294 | D00781 |
| 2 | hsa_7294 | D01180 |
| 2 | hsa_7294 | D01973 |
| 2 | hsa_7294 | D02341 |
| 2 | hsa_7294 | D03218 |
| 2 | hsa_7294 | D03743 |
| 2 | hsa_7297 | D00451 |
| 2 | hsa_7297 | D00512 |
| 2 | hsa_7297 | D00726 |
| 2 | hsa_7297 | D01136 |
| 2 | hsa_7297 | D02333 |
| 2 | hsa_7298 | D00225 |
| 2 | hsa_7298 | D00434 |
| 2 | hsa_7298 | D00455 |
| 2 | hsa_7298 | D00995 |
| 2 | hsa_7298 | D02563 |
| 2 | hsa_7298 | D04983 |
| 2 | hsa_7299 | D00325 |
| 2 | hsa_7299 | D00333 |
| 2 | hsa_7299 | D00621 |
| 2 | hsa_7299 | D00651 |
| 2 | hsa_7299 | D00670 |
| 2 | hsa_7299 | D01825 |
| 2 | hsa_7299 | D01862 |
| 2 | hsa_7299 | D01885 |
| 2 | hsa_7299 | D03776 |
| 2 | hsa_7301 | D00227 |
| 2 | hsa_7301 | D00274 |
| 2 | hsa_7301 | D00324 |
| 2 | hsa_7301 | D00362 |
| 2 | hsa_7301 | D00709 |
| 2 | hsa_7301 | D01223 |
| 2 | hsa_7301 | D01565 |
| 2 | hsa_7301 | D01712 |
| 2 | hsa_7301 | D02341 |
| 2 | hsa_7301 | D03034 |
| 2 | hsa_7301 | D03643 |
| 2 | hsa_7301 | D03738 |
| 2 | hsa_7363 | D00043 |
| 2 | hsa_7363 | D01122 |
| 2 | hsa_7363 | D01136 |
| 2 | hsa_7363 | D01888 |
| 2 | hsa_7363 | D02564 |
| 2 | hsa_7363 | D03218 |
| 2 | hsa_7364 | D00216 |
| 2 | hsa_7364 | D00889 |
| 2 | hsa_7364 | D02375 |
| 2 | hsa_7364 | D03714 |
| 2 | hsa_7364 | D04966 |
| 2 | hsa_7365 | D00120 |
| 2 | hsa_7365 | D00251 |
| 2 | hsa_7365 | D00567 |
| 2 | hsa_7365 | D00827 |
| 2 | hsa_7365 | D00884 |
| 2 | hsa_7365 | D00970 |
| 2 | hsa_7365 | D01704 |
| 2 | hsa_7365 | D02323 |
| 2 | hsa_7365 | D02335 |
| 2 | hsa_7365 | D03689 |
| 2 | hsa_7365 | D03772 |
| 2 | hsa_7365 | D03773 |
| 2 | hsa_7366 | D00630 |
| 2 | hsa_7366 | D01704 |
| 2 | hsa_7366 | D01842 |
| 2 | hsa_7366 | D02368 |
| 2 | hsa_7367 | D00187 |
| 2 | hsa_7367 | D00219 |
| 2 | hsa_7367 | D00563 |
| 2 | hsa_7367 | D00733 |
| 2 | hsa_7367 | D00884 |
| 2 | hsa_7367 | D01325 |
| 2 | hsa_7367 | D03788 |
| 2 | hsa_7371 | D00279 |
| 2 | hsa_7371 | D00463 |
| 2 | hsa_7371 | D00569 |
| 2 | hsa_7371 | D02355 |
| 2 | hsa_7371 | D03350 |
| 2 | hsa_7371 | D03758 |
| 2 | hsa_7372 | D00107 |
| 2 | hsa_7372 | D00148 |
| 2 | hsa_7372 | D00418 |
| 2 | hsa_7372 | D00423 |
| 2 | hsa_7372 | D00451 |
| 2 | hsa_7372 | D00562 |
| 2 | hsa_7372 | D01119 |
| 2 | hsa_7372 | D01718 |
| 2 | hsa_7372 | D02173 |
| 2 | hsa_7372 | D03753 |
| 2 | hsa_7372 | D04024 |
| 2 | hsa_7378 | D00377 |
| 2 | hsa_7378 | D00753 |
| 2 | hsa_7378 | D01183 |
| 2 | hsa_7378 | D01704 |
| 2 | hsa_7378 | D02258 |
| 2 | hsa_7453 | D00018 |
| 2 | hsa_7453 | D00120 |
| 2 | hsa_7453 | D00187 |
| 2 | hsa_7453 | D00451 |
| 2 | hsa_7453 | D00969 |
| 2 | hsa_7453 | D01256 |
| 2 | hsa_7453 | D01811 |
| 2 | hsa_7453 | D02258 |
| 2 | hsa_7453 | D02564 |
| 2 | hsa_7453 | D03882 |
| 2 | hsa_7498 | D00107 |
| 2 | hsa_7498 | D00142 |
| 2 | hsa_7498 | D00324 |
| 2 | hsa_7498 | D00624 |
| 2 | hsa_7498 | D02321 |
| 2 | hsa_7498 | D02563 |
| 2 | hsa_7498 | D03728 |
| 2 | hsa_7498 | D03743 |
| 2 | hsa_7498 | D04031 |
| 2 | hsa_7525 | D00293 |
| 2 | hsa_7525 | D00425 |
| 2 | hsa_7525 | D01240 |
| 2 | hsa_7525 | D02194 |
| 2 | hsa_7525 | D03077 |
| 2 | hsa_7525 | D03218 |
| 2 | hsa_7535 | D00037 |
| 2 | hsa_7535 | D00155 |
| 2 | hsa_7535 | D00183 |
| 2 | hsa_7535 | D00270 |
| 2 | hsa_7535 | D00298 |
| 2 | hsa_7535 | D00510 |
| 2 | hsa_7535 | D00658 |
| 2 | hsa_7535 | D00882 |
| 2 | hsa_7535 | D00887 |
| 2 | hsa_7535 | D00965 |
| 2 | hsa_7535 | D02315 |
| 2 | hsa_7535 | D02581 |
| 2 | hsa_759 | D00029 |
| 2 | hsa_759 | D00380 |
| 2 | hsa_759 | D00516 |
| 2 | hsa_759 | D00546 |
| 2 | hsa_759 | D00893 |
| 2 | hsa_759 | D02115 |
| 2 | hsa_760 | D00423 |
| 2 | hsa_760 | D00521 |
| 2 | hsa_760 | D00621 |
| 2 | hsa_760 | D01907 |
| 2 | hsa_760 | D01977 |
| 2 | hsa_760 | D02042 |
| 2 | hsa_760 | D02176 |
| 2 | hsa_760 | D02563 |
| 2 | hsa_760 | D03034 |
| 2 | hsa_760 | D03670 |
| 2 | hsa_761 | D00007 |
| 2 | hsa_761 | D00198 |
| 2 | hsa_761 | D00667 |
| 2 | hsa_761 | D00813 |
| 2 | hsa_761 | D00889 |
| 2 | hsa_761 | D00963 |
| 2 | hsa_761 | D03822 |
| 2 | hsa_762 | D00496 |
| 2 | hsa_762 | D00545 |
| 2 | hsa_762 | D00813 |
| 2 | hsa_762 | D01690 |
| 2 | hsa_762 | D01811 |
| 2 | hsa_762 | D03115 |
| 2 | hsa_762 | D03760 |
| 2 | hsa_762 | D03823 |
| 2 | hsa_762 | D04031 |
| 2 | hsa_763 | D00142 |
| 2 | hsa_763 | D00203 |
| 2 | hsa_763 | D00298 |
| 2 | hsa_763 | D02487 |
| 2 | hsa_763 | D03772 |
| 2 | hsa_763 | D03776 |
| 2 | hsa_765 | D00227 |
| 2 | hsa_765 | D00549 |
| 2 | hsa_765 | D00726 |
| 2 | hsa_765 | D00771 |
| 2 | hsa_765 | D01767 |
| 2 | hsa_765 | D02335 |
| 2 | hsa_765 | D03826 |
| 2 | hsa_766 | D00279 |
| 2 | hsa_766 | D00437 |
| 2 | hsa_766 | D00537 |
| 2 | hsa_766 | D01276 |
| 2 | hsa_766 | D01513 |
| 2 | hsa_766 | D01549 |
| 2 | hsa_766 | D01718 |
| 2 | hsa_766 | D02304 |
| 2 | hsa_766 | D02562 |
| 2 | hsa_766 | D03034 |
| 2 | hsa_766 | D03710 |
| 2 | hsa_766 | D03720 |
| 2 | hsa_767 | D00148 |
| 2 | hsa_767 | D00362 |
| 2 | hsa_767 | D00377 |
| 2 | hsa_767 | D00512 |
| 2 | hsa_767 | D00968 |
| 2 | hsa_767 | D01183 |
| 2 | hsa_767 | D01332 |
| 2 | hsa_767 | D01966 |
| 2 | hsa_767 | D02756 |
| 2 | hsa_767 | D03643 |
| 2 | hsa_767 | D03689 |
| 2 | hsa_767 | D03806 |
| 2 | hsa_768 | D00421 |
| 2 | hsa_768 | D00544 |
| 2 | hsa_768 | D00566 |
| 2 | hsa_768 | D00998 |
| 2 | hsa_768 | D01240 |
| 2 | hsa_768 | D02110 |
| 2 | hsa_768 | D02835 |
| 2 | hsa_768 | D03034 |
| 2 | hsa_771 | D00097 |
| 2 | hsa_771 | D00216 |
| 2 | hsa_771 | D00418 |
| 2 | hsa_771 | D00455 |
| 2 | hsa_771 | D00513 |
| 2 | hsa_771 | D00533 |
| 2 | hsa_771 | D00560 |
| 2 | hsa_771 | D00887 |
| 2 | hsa_771 | D01840 |
| 2 | hsa_771 | D03722 |
| 2 | hsa_771 | D03741 |
| 2 | hsa_771 | D04028 |
| 2 | hsa_771 | D05341 |
| 2 | hsa_780 | D00417 |
| 2 | hsa_780 | D00434 |
| 2 | hsa_780 | D00448 |
| 2 | hsa_780 | D00827 |
| 2 | hsa_780 | D00903 |
| 2 | hsa_780 | D01256 |
| 2 | hsa_780 | D01346 |
| 2 | hsa_780 | D01370 |
| 2 | hsa_780 | D03826 |
| 2 | hsa_79001 | D00125 |
| 2 | hsa_79001 | D00234 |
| 2 | hsa_79001 | D00566 |
| 2 | hsa_79001 | D00579 |
| 2 | hsa_79001 | D00652 |
| 2 | hsa_79001 | D00826 |
| 2 | hsa_79001 | D00829 |
| 2 | hsa_79001 | D01133 |
| 2 | hsa_79001 | D01425 |
| 2 | hsa_79001 | D01907 |
| 2 | hsa_79001 | D03643 |
| 2 | hsa_7957 | D00315 |
| 2 | hsa_7957 | D00418 |
| 2 | hsa_7957 | D00512 |
| 2 | hsa_7957 | D00519 |
| 2 | hsa_7957 | D00623 |
| 2 | hsa_7957 | D00670 |
| 2 | hsa_7957 | D00753 |
| 2 | hsa_7957 | D01367 |
| 2 | hsa_7957 | D02290 |
| 2 | hsa_7957 | D02562 |
| 2 | hsa_79799 | D00234 |
| 2 | hsa_79799 | D00285 |
| 2 | hsa_79799 | D00546 |
| 2 | hsa_79799 | D00624 |
| 2 | hsa_79799 | D00771 |
| 2 | hsa_79799 | D01180 |
| 2 | hsa_79799 | D01441 |
| 2 | hsa_80339 | D00035 |
| 2 | hsa_80339 | D00279 |
| 2 | hsa_80339 | D00359 |
| 2 | hsa_80339 | D00459 |
| 2 | hsa_80339 | D00653 |
| 2 | hsa_80339 | D00658 |
| 2 | hsa_80339 | D00968 |
| 2 | hsa_80339 | D01228 |
| 2 | hsa_80339 | D02068 |
| 2 | hsa_80339 | D02323 |
| 2 | hsa_80339 | D03115 |
| 2 | hsa_80339 | D03720 |
| 2 | hsa_80339 | D03816 |
| 2 | hsa_80339 | D03823 |
| 2 | hsa_80824 | D00401 |
| 2 | hsa_80824 | D00454 |
| 2 | hsa_80824 | D00709 |
| 2 | hsa_80824 | D01690 |
| 2 | hsa_80824 | D02308 |
| 2 | hsa_80824 | D02321 |
| 2 | hsa_80824 | D03807 |
| 2 | hsa_81579 | D00451 |
| 2 | hsa_81579 | D00562 |
| 2 | hsa_81579 | D00810 |
| 2 | hsa_81579 | D00893 |
| 2 | hsa_81579 | D00902 |
| 2 | hsa_81579 | D00970 |
| 2 | hsa_81579 | D01183 |
| 2 | hsa_81579 | D01425 |
| 2 | hsa_81579 | D01475 |
| 2 | hsa_81579 | D01866 |
| 2 | hsa_81579 | D01900 |
| 2 | hsa_81579 | D02176 |
| 2 | hsa_81579 | D02194 |
| 2 | hsa_81579 | D03769 |
| 2 | hsa_81579 | D03775 |
| 2 | hsa_8192 | D00416 |
| 2 | hsa_8192 | D00512 |
| 2 | hsa_8192 | D00620 |
| 2 | hsa_8192 | D01071 |
| 2 | hsa_8192 | D01198 |
| 2 | hsa_8192 | D01223 |
| 2 | hsa_8192 | D01911 |
| 2 | hsa_8192 | D03735 |
| 2 | hsa_8192 | D03751 |
| 2 | hsa_8192 | D03823 |
| 2 | hsa_8288 | D00283 |
| 2 | hsa_8288 | D00543 |
| 2 | hsa_8288 | D01027 |
| 2 | hsa_8288 | D01811 |
| 2 | hsa_8288 | D02115 |
| 2 | hsa_8288 | D03751 |
| 2 | hsa_834 | D00283 |
| 2 | hsa_834 | D00516 |
| 2 | hsa_834 | D01397 |
| 2 | hsa_834 | D03787 |
| 2 | hsa_834 | D03828 |
| 2 | hsa_8398 | D00771 |
| 2 | hsa_8398 | D01565 |
| 2 | hsa_8398 | D01966 |
| 2 | hsa_8398 | D01984 |
| 2 | hsa_8398 | D02115 |
| 2 | hsa_8399 | D00569 |
| 2 | hsa_84152 | D00049 |
| 2 | hsa_84152 | D00279 |
| 2 | hsa_84152 | D00377 |
| 2 | hsa_84152 | D00437 |
| 2 | hsa_84152 | D00995 |
| 2 | hsa_84152 | D01071 |
| 2 | hsa_84152 | D02581 |
| 2 | hsa_84152 | D03767 |
| 2 | hsa_84152 | D03828 |
| 2 | hsa_84152 | D03829 |
| 2 | hsa_84171 | D00132 |
| 2 | hsa_84171 | D00160 |
| 2 | hsa_84171 | D00369 |
| 2 | hsa_84171 | D00387 |
| 2 | hsa_84171 | D00487 |
| 2 | hsa_84171 | D00547 |
| 2 | hsa_84171 | D02008 |
| 2 | hsa_84171 | D02709 |
| 2 | hsa_84171 | D03788 |
| 2 | hsa_8435 | D00002 |
| 2 | hsa_8435 | D00359 |
| 2 | hsa_8435 | D00362 |
| 2 | hsa_8435 | D00519 |
| 2 | hsa_8435 | D00560 |
| 2 | hsa_8435 | D00653 |
| 2 | hsa_8435 | D00785 |
| 2 | hsa_8435 | D01441 |
| 2 | hsa_8435 | D02333 |
| 2 | hsa_8435 | D02487 |
| 2 | hsa_8435 | D06238 |
| 2 | hsa_84532 | D00153 |
| 2 | hsa_84532 | D00503 |
| 2 | hsa_84532 | D00622 |
| 2 | hsa_84532 | D01276 |
| 2 | hsa_84532 | D01513 |
| 2 | hsa_84532 | D01844 |
| 2 | hsa_84532 | D03077 |
| 2 | hsa_84532 | D03769 |
| 2 | hsa_84532 | D03784 |
| 2 | hsa_84618 | D00324 |
| 2 | hsa_84618 | D00496 |
| 2 | hsa_84618 | D00547 |
| 2 | hsa_84618 | D01704 |
| 2 | hsa_84618 | D02355 |
| 2 | hsa_84618 | D03115 |
| 2 | hsa_84618 | D03643 |
| 2 | hsa_84618 | D03710 |
| 2 | hsa_84618 | D03807 |
| 2 | hsa_84695 | D00007 |
| 2 | hsa_84695 | D01027 |
| 2 | hsa_84695 | D01211 |
| 2 | hsa_84695 | D02290 |
| 2 | hsa_84695 | D03115 |
| 2 | hsa_84695 | D03805 |
| 2 | hsa_84706 | D00398 |
| 2 | hsa_84706 | D00414 |
| 2 | hsa_84706 | D00670 |
| 2 | hsa_84706 | D00887 |
| 2 | hsa_84706 | D01578 |
| 2 | hsa_84706 | D01825 |
| 2 | hsa_84706 | D02709 |
| 2 | hsa_84706 | D03826 |
| 2 | hsa_84812 | D00752 |
| 2 | hsa_84812 | D01027 |
| 2 | hsa_84812 | D01122 |
| 2 | hsa_84812 | D01966 |
| 2 | hsa_84812 | D02008 |
| 2 | hsa_84812 | D02333 |
| 2 | hsa_84812 | D02487 |
| 2 | hsa_8513 | D00131 |
| 2 | hsa_8513 | D00203 |
| 2 | hsa_8513 | D00503 |
| 2 | hsa_8513 | D02110 |
| 2 | hsa_8513 | D03218 |
| 2 | hsa_8513 | D05341 |
| 2 | hsa_8529 | D00094 |
| 2 | hsa_8529 | D00183 |
| 2 | hsa_8529 | D00234 |
| 2 | hsa_8529 | D01122 |
| 2 | hsa_8529 | D02214 |
| 2 | hsa_8529 | D02333 |
| 2 | hsa_8529 | D02655 |
| 2 | hsa_8529 | D03077 |
| 2 | hsa_8529 | D03734 |
| 2 | hsa_85313 | D00043 |
| 2 | hsa_85313 | D00186 |
| 2 | hsa_85313 | D00293 |
| 2 | hsa_85313 | D00547 |
| 2 | hsa_85313 | D00703 |
| 2 | hsa_85313 | D00884 |
| 2 | hsa_85313 | D01119 |
| 2 | hsa_85313 | D01513 |
| 2 | hsa_85313 | D02756 |
| 2 | hsa_85313 | D03034 |
| 2 | hsa_85313 | D03440 |
| 2 | hsa_8555 | D00097 |
| 2 | hsa_8555 | D00550 |
| 2 | hsa_8555 | D00810 |
| 2 | hsa_8555 | D00889 |
| 2 | hsa_8555 | D00969 |
| 2 | hsa_8555 | D01709 |
| 2 | hsa_8555 | D02560 |
| 2 | hsa_8555 | D02564 |
| 2 | hsa_8555 | D03012 |
| 2 | hsa_8556 | D00317 |
| 2 | hsa_8556 | D00410 |
| 2 | hsa_8556 | D00501 |
| 2 | hsa_8556 | D00544 |
| 2 | hsa_8556 | D00650 |
| 2 | hsa_8556 | D00670 |
| 2 | hsa_8556 | D00902 |
| 2 | hsa_8556 | D01119 |
| 2 | hsa_8556 | D01915 |
| 2 | hsa_8556 | D03601 |
| 2 | hsa_8556 | D03829 |
| 2 | hsa_8622 | D00065 |
| 2 | hsa_8622 | D00519 |
| 2 | hsa_8622 | D00623 |
| 2 | hsa_8622 | D00652 |
| 2 | hsa_8622 | D01196 |
| 2 | hsa_8622 | D01325 |
| 2 | hsa_8622 | D02368 |
| 2 | hsa_8654 | D00014 |
| 2 | hsa_8654 | D00198 |
| 2 | hsa_8654 | D00421 |
| 2 | hsa_8654 | D00885 |
| 2 | hsa_8654 | D00998 |
| 2 | hsa_8654 | D02115 |
| 2 | hsa_8836 | D02068 |
| 2 | hsa_8836 | D05458 |
| 2 | hsa_8854 | D00050 |
| 2 | hsa_8854 | D00544 |
| 2 | hsa_8854 | D00547 |
| 2 | hsa_8854 | D00786 |
| 2 | hsa_8854 | D00947 |
| 2 | hsa_8854 | D00960 |
| 2 | hsa_8854 | D00998 |
| 2 | hsa_8854 | D01183 |
| 2 | hsa_8854 | D01767 |
| 2 | hsa_8854 | D02267 |
| 2 | hsa_8854 | D03822 |
| 2 | hsa_8940 | D00005 |
| 2 | hsa_8940 | D00168 |
| 2 | hsa_8940 | D00369 |
| 2 | hsa_8940 | D00377 |
| 2 | hsa_8940 | D00546 |
| 2 | hsa_8940 | D00752 |
| 2 | hsa_8940 | D01071 |
| 2 | hsa_8940 | D02068 |
| 2 | hsa_8940 | D03828 |
| 2 | hsa_8940 | D04024 |
| 2 | hsa_8972 | D00021 |
| 2 | hsa_8972 | D00125 |
| 2 | hsa_8972 | D00145 |
| 2 | hsa_8972 | D00168 |
| 2 | hsa_8972 | D00183 |
| 2 | hsa_8972 | D00414 |
| 2 | hsa_8972 | D00512 |
| 2 | hsa_8972 | D00903 |
| 2 | hsa_8972 | D00947 |
| 2 | hsa_8972 | D01223 |
| 2 | hsa_8972 | D01765 |
| 2 | hsa_8972 | D03788 |
| 2 | hsa_9023 | D00127 |
| 2 | hsa_9023 | D00224 |
| 2 | hsa_9023 | D00892 |
| 2 | hsa_9023 | D00965 |
| 2 | hsa_9023 | D01811 |
| 2 | hsa_9023 | D03034 |
| 2 | hsa_9023 | D03689 |
| 2 | hsa_9023 | D04292 |
| 2 | hsa_9088 | D00049 |
| 2 | hsa_9088 | D00203 |
| 2 | hsa_9088 | D00391 |
| 2 | hsa_9088 | D00425 |
| 2 | hsa_9088 | D00434 |
| 2 | hsa_9088 | D00542 |
| 2 | hsa_9088 | D00563 |
| 2 | hsa_9088 | D00596 |
| 2 | hsa_9088 | D02068 |
| 2 | hsa_9088 | D05407 |
| 2 | hsa_90 | D00035 |
| 2 | hsa_90 | D00131 |
| 2 | hsa_90 | D00158 |
| 2 | hsa_90 | D00196 |
| 2 | hsa_90 | D00542 |
| 2 | hsa_90 | D00593 |
| 2 | hsa_90 | D00786 |
| 2 | hsa_90 | D00829 |
| 2 | hsa_90 | D01712 |
| 2 | hsa_90 | D01765 |
| 2 | hsa_90 | D03784 |
| 2 | hsa_91039 | D00029 |
| 2 | hsa_91039 | D00097 |
| 2 | hsa_91039 | D00131 |
| 2 | hsa_91039 | D01064 |
| 2 | hsa_91039 | D01547 |
| 2 | hsa_91039 | D02115 |
| 2 | hsa_91039 | D02487 |
| 2 | hsa_91039 | D02579 |
| 2 | hsa_91039 | D02835 |
| 2 | hsa_9150 | D00219 |
| 2 | hsa_9150 | D00630 |
| 2 | hsa_9150 | D00810 |
| 2 | hsa_9150 | D01027 |
| 2 | hsa_9150 | D01069 |
| 2 | hsa_9150 | D01180 |
| 2 | hsa_9150 | D01828 |
| 2 | hsa_9150 | D02193 |
| 2 | hsa_9150 | D02418 |
| 2 | hsa_9150 | D03829 |
| 2 | hsa_91 | D00054 |
| 2 | hsa_91 | D00216 |
| 2 | hsa_91 | D00225 |
| 2 | hsa_91 | D00418 |
| 2 | hsa_91 | D01397 |
| 2 | hsa_91 | D01513 |
| 2 | hsa_91 | D01688 |
| 2 | hsa_91 | D03643 |
| 2 | hsa_91 | D03716 |
| 2 | hsa_93650 | D00005 |
| 2 | hsa_93650 | D00887 |
| 2 | hsa_93650 | D01097 |
| 2 | hsa_93650 | D01767 |
| 2 | hsa_93650 | D02556 |
| 2 | hsa_93650 | D05341 |
| 2 | hsa_9388 | D00198 |
| 2 | hsa_9388 | D00538 |
| 2 | hsa_9388 | D00550 |
| 2 | hsa_9388 | D00786 |
| 2 | hsa_9388 | D01136 |
| 2 | hsa_9388 | D01196 |
| 2 | hsa_9388 | D02323 |
| 2 | hsa_9388 | D02350 |
| 2 | hsa_9388 | D03823 |
| 2 | hsa_9388 | D04029 |
| 2 | hsa_93 | D00018 |
| 2 | hsa_93 | D00125 |
| 2 | hsa_93 | D00188 |
| 2 | hsa_93 | D00459 |
| 2 | hsa_93 | D00550 |
| 2 | hsa_93 | D02193 |
| 2 | hsa_93 | D02289 |
| 2 | hsa_93 | D03218 |
| 2 | hsa_94009 | D00065 |
| 2 | hsa_94009 | D00208 |
| 2 | hsa_94009 | D00562 |
| 2 | hsa_94009 | D00994 |
| 2 | hsa_94009 | D02562 |
| 2 | hsa_94009 | D02655 |
| 2 | hsa_94009 | D02729 |
| 2 | hsa_94009 | D03115 |
| 2 | hsa_94009 | D03822 |
| 2 | hsa_94009 | D03828 |
| 2 | hsa_9420 | D00132 |
| 2 | hsa_9420 | D00234 |
| 2 | hsa_9420 | D00455 |
| 2 | hsa_9420 | D00505 |
| 2 | hsa_9420 | D00593 |
| 2 | hsa_9420 | D03741 |
| 2 | hsa_9420 | D03816 |
| 2 | hsa_9420 | D04292 |
| 2 | hsa_94 | D00039 |
| 2 | hsa_94 | D00530 |
| 2 | hsa_94 | D00538 |
| 2 | hsa_94 | D00658 |
| 2 | hsa_94 | D01276 |
| 2 | hsa_94 | D01715 |
| 2 | hsa_94 | D01911 |
| 2 | hsa_94 | D02556 |
| 2 | hsa_9563 | D00055 |
| 2 | hsa_9563 | D00139 |
| 2 | hsa_9563 | D00155 |
| 2 | hsa_9563 | D00410 |
| 2 | hsa_9563 | D00960 |
| 2 | hsa_9563 | D02308 |
| 2 | hsa_9563 | D03738 |
| 2 | hsa_9563 | D03776 |
| 2 | hsa_9563 | D05341 |
| 2 | hsa_9601 | D00054 |
| 2 | hsa_9601 | D00142 |
| 2 | hsa_9601 | D00294 |
| 2 | hsa_9601 | D00359 |
| 2 | hsa_9601 | D00452 |
| 2 | hsa_9601 | D00463 |
| 2 | hsa_9601 | D00538 |
| 2 | hsa_9601 | D02342 |
| 2 | hsa_9601 | D03720 |
| 2 | hsa_9641 | D00139 |
| 2 | hsa_9641 | D00142 |
| 2 | hsa_9641 | D00198 |
| 2 | hsa_9641 | D00391 |
| 2 | hsa_9641 | D00487 |
| 2 | hsa_9641 | D00549 |
| 2 | hsa_9641 | D02333 |
| 2 | hsa_9641 | D02418 |
| 2 | hsa_9641 | D02756 |
| 2 | hsa_9647 | D00035 |
| 2 | hsa_9647 | D00132 |
| 2 | hsa_9647 | D00218 |
| 2 | hsa_9647 | D00274 |
| 2 | hsa_9647 | D00544 |
| 2 | hsa_9647 | D01918 |
| 2 | hsa_9647 | D02731 |
| 2 | hsa_9647 | D03752 |
| 2 | hsa_9647 | D03753 |
| 2 | hsa_9647 | D03781 |
| 2 | hsa_9945 | D00530 |
| 2 | hsa_9945 | D01578 |
| 2 | hsa_9945 | D02042 |
| 2 | hsa_9945 | D02290 |
| 2 | hsa_9955 | D00038 |
| 2 | hsa_9955 | D00125 |
| 2 | hsa_9955 | D00203 |
| 2 | hsa_9955 | D00584 |
| 2 | hsa_9955 | D00805 |
| 2 | hsa_9955 | D01133 |
| 2 | hsa_9955 | D01364 |
| 2 | hsa_9955 | D02304 |
| 2 | hsa_9955 | D02558 |
| 2 | hsa_9955 | D02561 |
| 2 | hsa_9955 | D02579 |
| 2 | hsa_9955 | D03753 |
