# Supplementary material for: Predicting Drug-Target Interaction Networks Based on Functional Groups and Biological Features
Source: PLoS One. 2010 Mar 11;5(3):e9603. doi: 10.1371/journal.pone.0009603 (PMC2836373; doi:10.1371/journal.pone.0009603)
Supplement: Online Supporting Information S2 — The benchmark dataset for the drug-target ion channel interaction system. It contains 4,116 gene-drug pair samples, of which 1,372 are positive and 2,744 negative. The 1st column of the table indicates the nature of samples with 1 for positive and 2 for negative; the 2nd column shows the code of target gene; and the 3rd column shows the code of drug. All the detailed information for the genes and drugs listed here can be found in KEGG via their codes (see the caption of Online Supporting Information A for further explanation). (3.35 MB DOC) [file pone.0009603.s002.doc]

**Online Supporting Information S2**: The benchmark dataset for the drug-target ion channel interaction system. It contains 4,116 gene-drug pair samples, of which 1,372 are positive and 2,744 negative. The 1st column of the table indicates the nature of samples with 1 for positive and 2 for negative; the 2nd column shows the code of target gene; and the 3rd column shows the code of drug. All the detailed information for the genes and drugs listed here can be found in KEGG via their codes (see the caption of Online Supporting Information A for further explanation).

| **Group** | **Target Gene** | **Drug** |
| --- | --- | --- |
| 1 | hsa_10008 | D00294 |
| 1 | hsa_10008 | D02356 |
| 1 | hsa_10060 | D02356 |
| 1 | hsa_10369 | D00332 |
| 1 | hsa_10369 | D00437 |
| 1 | hsa_10369 | D00512 |
| 1 | hsa_10369 | D00542 |
| 1 | hsa_10369 | D00560 |
| 1 | hsa_10369 | D00618 |
| 1 | hsa_10369 | D00629 |
| 1 | hsa_10369 | D01295 |
| 1 | hsa_10369 | D02356 |
| 1 | hsa_1080 | D00227 |
| 1 | hsa_1080 | D00336 |
| 1 | hsa_1080 | D01453 |
| 1 | hsa_1080 | D01712 |
| 1 | hsa_1080 | D02409 |
| 1 | hsa_11254 | D00035 |
| 1 | hsa_11280 | D00110 |
| 1 | hsa_1134 | D00195 |
| 1 | hsa_1134 | D00611 |
| 1 | hsa_1134 | D00726 |
| 1 | hsa_1134 | D02101 |
| 1 | hsa_1134 | D02173 |
| 1 | hsa_1134 | D02204 |
| 1 | hsa_1134 | D02207 |
| 1 | hsa_1134 | D03826 |
| 1 | hsa_1134 | D05156 |
| 1 | hsa_1134 | D05453 |
| 1 | hsa_1135 | D00195 |
| 1 | hsa_1135 | D00499 |
| 1 | hsa_1135 | D00524 |
| 1 | hsa_1135 | D00611 |
| 1 | hsa_1135 | D00726 |
| 1 | hsa_1135 | D00759 |
| 1 | hsa_1135 | D00760 |
| 1 | hsa_1135 | D00761 |
| 1 | hsa_1135 | D00765 |
| 1 | hsa_1135 | D00779 |
| 1 | hsa_1135 | D02101 |
| 1 | hsa_1135 | D02173 |
| 1 | hsa_1135 | D02204 |
| 1 | hsa_1135 | D02207 |
| 1 | hsa_1135 | D03742 |
| 1 | hsa_1135 | D03826 |
| 1 | hsa_1135 | D05156 |
| 1 | hsa_1135 | D05453 |
| 1 | hsa_1136 | D00195 |
| 1 | hsa_1136 | D00499 |
| 1 | hsa_1136 | D00524 |
| 1 | hsa_1136 | D00611 |
| 1 | hsa_1136 | D00726 |
| 1 | hsa_1136 | D02101 |
| 1 | hsa_1136 | D02173 |
| 1 | hsa_1136 | D02204 |
| 1 | hsa_1136 | D02207 |
| 1 | hsa_1136 | D03826 |
| 1 | hsa_1136 | D05156 |
| 1 | hsa_1136 | D05453 |
| 1 | hsa_1137 | D00195 |
| 1 | hsa_1137 | D00499 |
| 1 | hsa_1137 | D00524 |
| 1 | hsa_1137 | D00611 |
| 1 | hsa_1137 | D00726 |
| 1 | hsa_1137 | D02101 |
| 1 | hsa_1137 | D02173 |
| 1 | hsa_1137 | D02204 |
| 1 | hsa_1137 | D02207 |
| 1 | hsa_1137 | D03826 |
| 1 | hsa_1137 | D05156 |
| 1 | hsa_1137 | D05453 |
| 1 | hsa_1138 | D00195 |
| 1 | hsa_1138 | D00611 |
| 1 | hsa_1138 | D02101 |
| 1 | hsa_1138 | D02173 |
| 1 | hsa_1138 | D02204 |
| 1 | hsa_1138 | D02207 |
| 1 | hsa_1138 | D03826 |
| 1 | hsa_1138 | D05156 |
| 1 | hsa_1138 | D05453 |
| 1 | hsa_1139 | D00195 |
| 1 | hsa_1139 | D00611 |
| 1 | hsa_1139 | D00726 |
| 1 | hsa_1139 | D02101 |
| 1 | hsa_1139 | D02173 |
| 1 | hsa_1139 | D02204 |
| 1 | hsa_1139 | D02207 |
| 1 | hsa_1139 | D03826 |
| 1 | hsa_1139 | D05156 |
| 1 | hsa_1139 | D05453 |
| 1 | hsa_1140 | D00726 |
| 1 | hsa_1141 | D00499 |
| 1 | hsa_1141 | D00524 |
| 1 | hsa_1141 | D00574 |
| 1 | hsa_1141 | D00726 |
| 1 | hsa_1141 | D00960 |
| 1 | hsa_1141 | D00963 |
| 1 | hsa_1141 | D00964 |
| 1 | hsa_1144 | D00726 |
| 1 | hsa_1145 | D00499 |
| 1 | hsa_1145 | D00524 |
| 1 | hsa_1145 | D00726 |
| 1 | hsa_116443 | D00332 |
| 1 | hsa_116443 | D00536 |
| 1 | hsa_116443 | D00711 |
| 1 | hsa_116443 | D00775 |
| 1 | hsa_116443 | D01118 |
| 1 | hsa_116443 | D03742 |
| 1 | hsa_116443 | D03878 |
| 1 | hsa_116444 | D00136 |
| 1 | hsa_116444 | D00711 |
| 1 | hsa_116444 | D03878 |
| 1 | hsa_1181 | D04790 |
| 1 | hsa_169522 | D00294 |
| 1 | hsa_169522 | D02356 |
| 1 | hsa_170572 | D00633 |
| 1 | hsa_170572 | D00677 |
| 1 | hsa_170572 | D00678 |
| 1 | hsa_170572 | D02041 |
| 1 | hsa_200909 | D00633 |
| 1 | hsa_200909 | D00677 |
| 1 | hsa_200909 | D00678 |
| 1 | hsa_200909 | D02041 |
| 1 | hsa_22953 | D00528 |
| 1 | hsa_23630 | D00294 |
| 1 | hsa_23630 | D02356 |
| 1 | hsa_23704 | D00294 |
| 1 | hsa_23704 | D02356 |
| 1 | hsa_2554 | D00225 |
| 1 | hsa_2554 | D00293 |
| 1 | hsa_2554 | D00329 |
| 1 | hsa_2554 | D00331 |
| 1 | hsa_2554 | D00338 |
| 1 | hsa_2554 | D00464 |
| 1 | hsa_2554 | D00474 |
| 1 | hsa_2554 | D00499 |
| 1 | hsa_2554 | D00530 |
| 1 | hsa_2554 | D00537 |
| 1 | hsa_2554 | D00542 |
| 1 | hsa_2554 | D00543 |
| 1 | hsa_2554 | D00544 |
| 1 | hsa_2554 | D00545 |
| 1 | hsa_2554 | D00546 |
| 1 | hsa_2554 | D00547 |
| 1 | hsa_2554 | D00548 |
| 1 | hsa_2554 | D00549 |
| 1 | hsa_2554 | D00550 |
| 1 | hsa_2554 | D00694 |
| 1 | hsa_2554 | D00697 |
| 1 | hsa_2554 | D00704 |
| 1 | hsa_2554 | D00711 |
| 1 | hsa_2554 | D01071 |
| 1 | hsa_2554 | D01253 |
| 1 | hsa_2554 | D02207 |
| 1 | hsa_2554 | D02253 |
| 1 | hsa_2554 | D02461 |
| 1 | hsa_2554 | D03180 |
| 1 | hsa_2554 | D04985 |
| 1 | hsa_2554 | D05461 |
| 1 | hsa_2554 | D06106 |
| 1 | hsa_2555 | D00225 |
| 1 | hsa_2555 | D00293 |
| 1 | hsa_2555 | D00329 |
| 1 | hsa_2555 | D00331 |
| 1 | hsa_2555 | D00499 |
| 1 | hsa_2555 | D00530 |
| 1 | hsa_2555 | D00545 |
| 1 | hsa_2555 | D00547 |
| 1 | hsa_2555 | D00548 |
| 1 | hsa_2555 | D00549 |
| 1 | hsa_2555 | D00550 |
| 1 | hsa_2555 | D00711 |
| 1 | hsa_2555 | D02207 |
| 1 | hsa_2555 | D02253 |
| 1 | hsa_2555 | D02461 |
| 1 | hsa_2555 | D05461 |
| 1 | hsa_2556 | D00225 |
| 1 | hsa_2556 | D00293 |
| 1 | hsa_2556 | D00329 |
| 1 | hsa_2556 | D00331 |
| 1 | hsa_2556 | D00499 |
| 1 | hsa_2556 | D00530 |
| 1 | hsa_2556 | D00545 |
| 1 | hsa_2556 | D00547 |
| 1 | hsa_2556 | D00548 |
| 1 | hsa_2556 | D00549 |
| 1 | hsa_2556 | D00550 |
| 1 | hsa_2556 | D00711 |
| 1 | hsa_2556 | D02207 |
| 1 | hsa_2556 | D02253 |
| 1 | hsa_2556 | D02461 |
| 1 | hsa_2556 | D05461 |
| 1 | hsa_2557 | D00225 |
| 1 | hsa_2557 | D00293 |
| 1 | hsa_2557 | D00329 |
| 1 | hsa_2557 | D00331 |
| 1 | hsa_2557 | D00499 |
| 1 | hsa_2557 | D00530 |
| 1 | hsa_2557 | D00545 |
| 1 | hsa_2557 | D00547 |
| 1 | hsa_2557 | D00548 |
| 1 | hsa_2557 | D00549 |
| 1 | hsa_2557 | D00550 |
| 1 | hsa_2557 | D00711 |
| 1 | hsa_2557 | D02207 |
| 1 | hsa_2557 | D02253 |
| 1 | hsa_2557 | D02461 |
| 1 | hsa_2557 | D05461 |
| 1 | hsa_2558 | D00225 |
| 1 | hsa_2558 | D00293 |
| 1 | hsa_2558 | D00329 |
| 1 | hsa_2558 | D00331 |
| 1 | hsa_2558 | D00499 |
| 1 | hsa_2558 | D00530 |
| 1 | hsa_2558 | D00545 |
| 1 | hsa_2558 | D00547 |
| 1 | hsa_2558 | D00548 |
| 1 | hsa_2558 | D00549 |
| 1 | hsa_2558 | D00550 |
| 1 | hsa_2558 | D00711 |
| 1 | hsa_2558 | D02207 |
| 1 | hsa_2558 | D02253 |
| 1 | hsa_2558 | D02461 |
| 1 | hsa_2558 | D05461 |
| 1 | hsa_2559 | D00225 |
| 1 | hsa_2559 | D00293 |
| 1 | hsa_2559 | D00329 |
| 1 | hsa_2559 | D00331 |
| 1 | hsa_2559 | D00499 |
| 1 | hsa_2559 | D00530 |
| 1 | hsa_2559 | D00545 |
| 1 | hsa_2559 | D00547 |
| 1 | hsa_2559 | D00548 |
| 1 | hsa_2559 | D00549 |
| 1 | hsa_2559 | D00550 |
| 1 | hsa_2559 | D00711 |
| 1 | hsa_2559 | D02207 |
| 1 | hsa_2559 | D02253 |
| 1 | hsa_2559 | D02461 |
| 1 | hsa_2559 | D05461 |
| 1 | hsa_2560 | D00225 |
| 1 | hsa_2560 | D00293 |
| 1 | hsa_2560 | D00329 |
| 1 | hsa_2560 | D00499 |
| 1 | hsa_2560 | D00530 |
| 1 | hsa_2560 | D00545 |
| 1 | hsa_2560 | D00547 |
| 1 | hsa_2560 | D00548 |
| 1 | hsa_2560 | D00549 |
| 1 | hsa_2560 | D00550 |
| 1 | hsa_2561 | D00225 |
| 1 | hsa_2561 | D00293 |
| 1 | hsa_2561 | D00329 |
| 1 | hsa_2561 | D00499 |
| 1 | hsa_2561 | D00530 |
| 1 | hsa_2561 | D00545 |
| 1 | hsa_2561 | D00547 |
| 1 | hsa_2561 | D00548 |
| 1 | hsa_2561 | D00549 |
| 1 | hsa_2561 | D00550 |
| 1 | hsa_2562 | D00225 |
| 1 | hsa_2562 | D00293 |
| 1 | hsa_2562 | D00329 |
| 1 | hsa_2562 | D00499 |
| 1 | hsa_2562 | D00530 |
| 1 | hsa_2562 | D00545 |
| 1 | hsa_2562 | D00547 |
| 1 | hsa_2562 | D00548 |
| 1 | hsa_2562 | D00549 |
| 1 | hsa_2562 | D00550 |
| 1 | hsa_2563 | D00225 |
| 1 | hsa_2563 | D00293 |
| 1 | hsa_2563 | D00329 |
| 1 | hsa_2563 | D00499 |
| 1 | hsa_2563 | D00530 |
| 1 | hsa_2563 | D00545 |
| 1 | hsa_2563 | D00547 |
| 1 | hsa_2563 | D00548 |
| 1 | hsa_2563 | D00549 |
| 1 | hsa_2563 | D00550 |
| 1 | hsa_2564 | D00225 |
| 1 | hsa_2564 | D00293 |
| 1 | hsa_2564 | D00329 |
| 1 | hsa_2564 | D00499 |
| 1 | hsa_2564 | D00530 |
| 1 | hsa_2564 | D00545 |
| 1 | hsa_2564 | D00547 |
| 1 | hsa_2564 | D00548 |
| 1 | hsa_2564 | D00549 |
| 1 | hsa_2564 | D00550 |
| 1 | hsa_2566 | D00225 |
| 1 | hsa_2566 | D00293 |
| 1 | hsa_2566 | D00329 |
| 1 | hsa_2566 | D00499 |
| 1 | hsa_2566 | D00530 |
| 1 | hsa_2566 | D00545 |
| 1 | hsa_2566 | D00547 |
| 1 | hsa_2566 | D00548 |
| 1 | hsa_2566 | D00549 |
| 1 | hsa_2566 | D00550 |
| 1 | hsa_2567 | D00225 |
| 1 | hsa_2567 | D00293 |
| 1 | hsa_2567 | D00329 |
| 1 | hsa_2567 | D00499 |
| 1 | hsa_2567 | D00530 |
| 1 | hsa_2567 | D00545 |
| 1 | hsa_2567 | D00547 |
| 1 | hsa_2567 | D00548 |
| 1 | hsa_2567 | D00549 |
| 1 | hsa_2567 | D00550 |
| 1 | hsa_2569 | D00225 |
| 1 | hsa_2569 | D00293 |
| 1 | hsa_2569 | D00329 |
| 1 | hsa_2569 | D00499 |
| 1 | hsa_2569 | D00530 |
| 1 | hsa_2569 | D00545 |
| 1 | hsa_2569 | D00547 |
| 1 | hsa_2569 | D00548 |
| 1 | hsa_2569 | D00549 |
| 1 | hsa_2569 | D00550 |
| 1 | hsa_2570 | D00293 |
| 1 | hsa_2570 | D00530 |
| 1 | hsa_2570 | D00549 |
| 1 | hsa_26251 | D00294 |
| 1 | hsa_26251 | D02356 |
| 1 | hsa_27012 | D00294 |
| 1 | hsa_27012 | D02356 |
| 1 | hsa_27092 | D00332 |
| 1 | hsa_27092 | D00437 |
| 1 | hsa_27092 | D00512 |
| 1 | hsa_27092 | D00542 |
| 1 | hsa_27092 | D00560 |
| 1 | hsa_27092 | D00618 |
| 1 | hsa_27092 | D00629 |
| 1 | hsa_27092 | D01295 |
| 1 | hsa_27092 | D02356 |
| 1 | hsa_27094 | D00294 |
| 1 | hsa_27094 | D02356 |
| 1 | hsa_27133 | D00294 |
| 1 | hsa_27133 | D00642 |
| 1 | hsa_27133 | D00647 |
| 1 | hsa_27133 | D02272 |
| 1 | hsa_27133 | D02356 |
| 1 | hsa_27345 | D00294 |
| 1 | hsa_27345 | D02356 |
| 1 | hsa_2741 | D00293 |
| 1 | hsa_2741 | D00536 |
| 1 | hsa_2741 | D00542 |
| 1 | hsa_2741 | D00543 |
| 1 | hsa_2741 | D00544 |
| 1 | hsa_2741 | D00545 |
| 1 | hsa_2741 | D00546 |
| 1 | hsa_2741 | D00547 |
| 1 | hsa_2741 | D00549 |
| 1 | hsa_2741 | D02041 |
| 1 | hsa_2742 | D00293 |
| 1 | hsa_2742 | D00536 |
| 1 | hsa_2742 | D00547 |
| 1 | hsa_2742 | D00549 |
| 1 | hsa_2742 | D02041 |
| 1 | hsa_2743 | D00293 |
| 1 | hsa_2743 | D00536 |
| 1 | hsa_2743 | D00547 |
| 1 | hsa_2743 | D00549 |
| 1 | hsa_285242 | D00283 |
| 1 | hsa_285242 | D00451 |
| 1 | hsa_285242 | D00513 |
| 1 | hsa_285242 | D00633 |
| 1 | hsa_285242 | D00677 |
| 1 | hsa_285242 | D00678 |
| 1 | hsa_285242 | D00726 |
| 1 | hsa_285242 | D02041 |
| 1 | hsa_2890 | D00294 |
| 1 | hsa_2890 | D00542 |
| 1 | hsa_2890 | D00543 |
| 1 | hsa_2890 | D00544 |
| 1 | hsa_2890 | D00545 |
| 1 | hsa_2890 | D00546 |
| 1 | hsa_2890 | D00547 |
| 1 | hsa_2890 | D00775 |
| 1 | hsa_2891 | D00294 |
| 1 | hsa_2891 | D00775 |
| 1 | hsa_2891 | D01256 |
| 1 | hsa_2892 | D00294 |
| 1 | hsa_2892 | D00775 |
| 1 | hsa_2893 | D00294 |
| 1 | hsa_2893 | D00775 |
| 1 | hsa_2895 | D00775 |
| 1 | hsa_2897 | D00537 |
| 1 | hsa_2897 | D00775 |
| 1 | hsa_2897 | D02546 |
| 1 | hsa_2898 | D02546 |
| 1 | hsa_2899 | D00775 |
| 1 | hsa_2899 | D02546 |
| 1 | hsa_2900 | D00775 |
| 1 | hsa_2900 | D02546 |
| 1 | hsa_2901 | D00775 |
| 1 | hsa_2901 | D02546 |
| 1 | hsa_2902 | D00136 |
| 1 | hsa_2902 | D00711 |
| 1 | hsa_2902 | D03878 |
| 1 | hsa_2903 | D00136 |
| 1 | hsa_2903 | D00711 |
| 1 | hsa_2903 | D03878 |
| 1 | hsa_2904 | D00136 |
| 1 | hsa_2904 | D00711 |
| 1 | hsa_2904 | D03878 |
| 1 | hsa_2905 | D00136 |
| 1 | hsa_2905 | D00711 |
| 1 | hsa_2905 | D03878 |
| 1 | hsa_2906 | D00136 |
| 1 | hsa_2906 | D00711 |
| 1 | hsa_2906 | D03878 |
| 1 | hsa_3359 | D00283 |
| 1 | hsa_3359 | D00451 |
| 1 | hsa_3359 | D00456 |
| 1 | hsa_3359 | D00513 |
| 1 | hsa_3359 | D00563 |
| 1 | hsa_3359 | D00633 |
| 1 | hsa_3359 | D00677 |
| 1 | hsa_3359 | D00678 |
| 1 | hsa_3359 | D00726 |
| 1 | hsa_3359 | D02041 |
| 1 | hsa_3736 | D00294 |
| 1 | hsa_3736 | D00543 |
| 1 | hsa_3736 | D00544 |
| 1 | hsa_3736 | D00545 |
| 1 | hsa_3736 | D00546 |
| 1 | hsa_3736 | D00547 |
| 1 | hsa_3736 | D00616 |
| 1 | hsa_3736 | D00638 |
| 1 | hsa_3736 | D02356 |
| 1 | hsa_3736 | D03830 |
| 1 | hsa_3737 | D00294 |
| 1 | hsa_3737 | D00616 |
| 1 | hsa_3737 | D00638 |
| 1 | hsa_3737 | D02356 |
| 1 | hsa_3737 | D03830 |
| 1 | hsa_3738 | D00294 |
| 1 | hsa_3738 | D00616 |
| 1 | hsa_3738 | D00619 |
| 1 | hsa_3738 | D02261 |
| 1 | hsa_3738 | D02262 |
| 1 | hsa_3738 | D02356 |
| 1 | hsa_3738 | D03830 |
| 1 | hsa_3739 | D00294 |
| 1 | hsa_3739 | D00617 |
| 1 | hsa_3739 | D00642 |
| 1 | hsa_3739 | D02272 |
| 1 | hsa_3739 | D02356 |
| 1 | hsa_3741 | D00294 |
| 1 | hsa_3741 | D00364 |
| 1 | hsa_3741 | D00616 |
| 1 | hsa_3741 | D00638 |
| 1 | hsa_3741 | D00640 |
| 1 | hsa_3741 | D00642 |
| 1 | hsa_3741 | D01450 |
| 1 | hsa_3741 | D02272 |
| 1 | hsa_3741 | D02356 |
| 1 | hsa_3741 | D03830 |
| 1 | hsa_3742 | D00294 |
| 1 | hsa_3742 | D02356 |
| 1 | hsa_3743 | D00294 |
| 1 | hsa_3743 | D00619 |
| 1 | hsa_3743 | D00636 |
| 1 | hsa_3743 | D00638 |
| 1 | hsa_3743 | D00642 |
| 1 | hsa_3743 | D02272 |
| 1 | hsa_3743 | D02356 |
| 1 | hsa_3744 | D00294 |
| 1 | hsa_3744 | D00351 |
| 1 | hsa_3744 | D00619 |
| 1 | hsa_3744 | D02356 |
| 1 | hsa_3745 | D00294 |
| 1 | hsa_3745 | D02356 |
| 1 | hsa_3746 | D00294 |
| 1 | hsa_3746 | D00616 |
| 1 | hsa_3746 | D00638 |
| 1 | hsa_3746 | D02356 |
| 1 | hsa_3746 | D03830 |
| 1 | hsa_3747 | D00294 |
| 1 | hsa_3747 | D00619 |
| 1 | hsa_3747 | D02356 |
| 1 | hsa_3748 | D00294 |
| 1 | hsa_3748 | D02356 |
| 1 | hsa_3749 | D00294 |
| 1 | hsa_3749 | D02356 |
| 1 | hsa_3750 | D00294 |
| 1 | hsa_3750 | D02356 |
| 1 | hsa_3751 | D00294 |
| 1 | hsa_3751 | D02356 |
| 1 | hsa_3752 | D00294 |
| 1 | hsa_3752 | D01450 |
| 1 | hsa_3752 | D02356 |
| 1 | hsa_3752 | D05156 |
| 1 | hsa_3753 | D00294 |
| 1 | hsa_3753 | D00345 |
| 1 | hsa_3753 | D02356 |
| 1 | hsa_3754 | D00294 |
| 1 | hsa_3754 | D02356 |
| 1 | hsa_3755 | D00294 |
| 1 | hsa_3755 | D02356 |
| 1 | hsa_3756 | D00294 |
| 1 | hsa_3756 | D00642 |
| 1 | hsa_3756 | D02272 |
| 1 | hsa_3756 | D02356 |
| 1 | hsa_3757 | D00234 |
| 1 | hsa_3757 | D00274 |
| 1 | hsa_3757 | D00294 |
| 1 | hsa_3757 | D00351 |
| 1 | hsa_3757 | D00521 |
| 1 | hsa_3757 | D00647 |
| 1 | hsa_3757 | D00648 |
| 1 | hsa_3757 | D02092 |
| 1 | hsa_3757 | D02356 |
| 1 | hsa_3757 | D02485 |
| 1 | hsa_3758 | D00219 |
| 1 | hsa_3758 | D00294 |
| 1 | hsa_3758 | D00335 |
| 1 | hsa_3758 | D00336 |
| 1 | hsa_3758 | D00379 |
| 1 | hsa_3758 | D00380 |
| 1 | hsa_3758 | D00418 |
| 1 | hsa_3758 | D00593 |
| 1 | hsa_3758 | D00594 |
| 1 | hsa_3758 | D01111 |
| 1 | hsa_3758 | D02356 |
| 1 | hsa_3759 | D00294 |
| 1 | hsa_3759 | D02356 |
| 1 | hsa_3760 | D00294 |
| 1 | hsa_3760 | D02356 |
| 1 | hsa_3761 | D00294 |
| 1 | hsa_3761 | D02356 |
| 1 | hsa_3762 | D00294 |
| 1 | hsa_3762 | D02356 |
| 1 | hsa_3763 | D00294 |
| 1 | hsa_3763 | D00326 |
| 1 | hsa_3763 | D00373 |
| 1 | hsa_3763 | D00619 |
| 1 | hsa_3763 | D00798 |
| 1 | hsa_3763 | D00823 |
| 1 | hsa_3763 | D01450 |
| 1 | hsa_3763 | D02356 |
| 1 | hsa_3763 | D03878 |
| 1 | hsa_3764 | D00294 |
| 1 | hsa_3764 | D02356 |
| 1 | hsa_3765 | D00294 |
| 1 | hsa_3765 | D02356 |
| 1 | hsa_3766 | D00294 |
| 1 | hsa_3766 | D02356 |
| 1 | hsa_3767 | D00335 |
| 1 | hsa_3767 | D00379 |
| 1 | hsa_3767 | D00648 |
| 1 | hsa_3767 | D01599 |
| 1 | hsa_3767 | D01799 |
| 1 | hsa_3767 | D01854 |
| 1 | hsa_3768 | D00294 |
| 1 | hsa_3768 | D02356 |
| 1 | hsa_3769 | D00294 |
| 1 | hsa_3769 | D02356 |
| 1 | hsa_3772 | D00294 |
| 1 | hsa_3772 | D02356 |
| 1 | hsa_3773 | D00294 |
| 1 | hsa_3773 | D02356 |
| 1 | hsa_3775 | D00294 |
| 1 | hsa_3775 | D00648 |
| 1 | hsa_3775 | D02356 |
| 1 | hsa_3776 | D00642 |
| 1 | hsa_3776 | D00647 |
| 1 | hsa_3776 | D02272 |
| 1 | hsa_3778 | D00294 |
| 1 | hsa_3778 | D00340 |
| 1 | hsa_3778 | D00519 |
| 1 | hsa_3778 | D00542 |
| 1 | hsa_3778 | D00650 |
| 1 | hsa_3778 | D00651 |
| 1 | hsa_3778 | D00654 |
| 1 | hsa_3778 | D00656 |
| 1 | hsa_3778 | D00658 |
| 1 | hsa_3778 | D00771 |
| 1 | hsa_3778 | D01256 |
| 1 | hsa_3778 | D02356 |
| 1 | hsa_3779 | D00294 |
| 1 | hsa_3779 | D02356 |
| 1 | hsa_3780 | D00294 |
| 1 | hsa_3780 | D00326 |
| 1 | hsa_3780 | D00823 |
| 1 | hsa_3780 | D01575 |
| 1 | hsa_3780 | D02207 |
| 1 | hsa_3780 | D02356 |
| 1 | hsa_3781 | D00294 |
| 1 | hsa_3781 | D00326 |
| 1 | hsa_3781 | D00799 |
| 1 | hsa_3781 | D00809 |
| 1 | hsa_3781 | D00823 |
| 1 | hsa_3781 | D01448 |
| 1 | hsa_3781 | D02207 |
| 1 | hsa_3781 | D02234 |
| 1 | hsa_3781 | D02356 |
| 1 | hsa_3781 | D03274 |
| 1 | hsa_3781 | D04034 |
| 1 | hsa_3782 | D00294 |
| 1 | hsa_3782 | D00326 |
| 1 | hsa_3782 | D00480 |
| 1 | hsa_3782 | D00791 |
| 1 | hsa_3782 | D00809 |
| 1 | hsa_3782 | D00812 |
| 1 | hsa_3782 | D00816 |
| 1 | hsa_3782 | D00823 |
| 1 | hsa_3782 | D01242 |
| 1 | hsa_3782 | D02163 |
| 1 | hsa_3782 | D02356 |
| 1 | hsa_3782 | D03274 |
| 1 | hsa_3782 | D04034 |
| 1 | hsa_3783 | D00227 |
| 1 | hsa_3783 | D00294 |
| 1 | hsa_3783 | D00351 |
| 1 | hsa_3783 | D00542 |
| 1 | hsa_3783 | D01453 |
| 1 | hsa_3783 | D01712 |
| 1 | hsa_3783 | D02356 |
| 1 | hsa_3783 | D02409 |
| 1 | hsa_3783 | D03450 |
| 1 | hsa_3784 | D00294 |
| 1 | hsa_3784 | D00345 |
| 1 | hsa_3784 | D00831 |
| 1 | hsa_3784 | D02356 |
| 1 | hsa_3784 | D03037 |
| 1 | hsa_3785 | D00294 |
| 1 | hsa_3785 | D02356 |
| 1 | hsa_3786 | D00294 |
| 1 | hsa_3786 | D02356 |
| 1 | hsa_3787 | D00294 |
| 1 | hsa_3787 | D02356 |
| 1 | hsa_3788 | D00294 |
| 1 | hsa_3788 | D02356 |
| 1 | hsa_3790 | D00294 |
| 1 | hsa_3790 | D02356 |
| 1 | hsa_40 | D00649 |
| 1 | hsa_41 | D00649 |
| 1 | hsa_5023 | D00528 |
| 1 | hsa_5024 | D00528 |
| 1 | hsa_5025 | D00528 |
| 1 | hsa_5026 | D00528 |
| 1 | hsa_5027 | D00528 |
| 1 | hsa_50801 | D00294 |
| 1 | hsa_50801 | D02356 |
| 1 | hsa_51305 | D00294 |
| 1 | hsa_51305 | D02356 |
| 1 | hsa_51802 | D00252 |
| 1 | hsa_51802 | D00303 |
| 1 | hsa_51802 | D00332 |
| 1 | hsa_51802 | D00512 |
| 1 | hsa_51802 | D00538 |
| 1 | hsa_51802 | D00552 |
| 1 | hsa_51802 | D00649 |
| 1 | hsa_51802 | D00775 |
| 1 | hsa_51802 | D02356 |
| 1 | hsa_54207 | D00294 |
| 1 | hsa_54207 | D00642 |
| 1 | hsa_54207 | D02272 |
| 1 | hsa_54207 | D02356 |
| 1 | hsa_55503 | D00332 |
| 1 | hsa_55503 | D00437 |
| 1 | hsa_55503 | D00512 |
| 1 | hsa_55503 | D00542 |
| 1 | hsa_55503 | D00560 |
| 1 | hsa_55503 | D00618 |
| 1 | hsa_55503 | D00629 |
| 1 | hsa_55503 | D01295 |
| 1 | hsa_55503 | D02356 |
| 1 | hsa_55515 | D00252 |
| 1 | hsa_55515 | D00303 |
| 1 | hsa_55515 | D00332 |
| 1 | hsa_55515 | D00512 |
| 1 | hsa_55515 | D00538 |
| 1 | hsa_55515 | D00552 |
| 1 | hsa_55515 | D00649 |
| 1 | hsa_55515 | D00775 |
| 1 | hsa_55515 | D02356 |
| 1 | hsa_55584 | D00195 |
| 1 | hsa_55584 | D00499 |
| 1 | hsa_55584 | D00524 |
| 1 | hsa_55584 | D00611 |
| 1 | hsa_55584 | D00726 |
| 1 | hsa_55584 | D02101 |
| 1 | hsa_55584 | D02173 |
| 1 | hsa_55584 | D02204 |
| 1 | hsa_55584 | D02207 |
| 1 | hsa_55584 | D03826 |
| 1 | hsa_55584 | D05156 |
| 1 | hsa_55584 | D05453 |
| 1 | hsa_55799 | D00332 |
| 1 | hsa_55799 | D00437 |
| 1 | hsa_55799 | D00512 |
| 1 | hsa_55799 | D00542 |
| 1 | hsa_55799 | D00560 |
| 1 | hsa_55799 | D00618 |
| 1 | hsa_55799 | D00629 |
| 1 | hsa_55799 | D01295 |
| 1 | hsa_55799 | D02356 |
| 1 | hsa_55800 | D00252 |
| 1 | hsa_55800 | D00303 |
| 1 | hsa_55800 | D00332 |
| 1 | hsa_55800 | D00512 |
| 1 | hsa_55800 | D00538 |
| 1 | hsa_55800 | D00552 |
| 1 | hsa_55800 | D00775 |
| 1 | hsa_55800 | D02356 |
| 1 | hsa_55879 | D00225 |
| 1 | hsa_55879 | D00293 |
| 1 | hsa_55879 | D00329 |
| 1 | hsa_55879 | D00499 |
| 1 | hsa_55879 | D00530 |
| 1 | hsa_55879 | D00545 |
| 1 | hsa_55879 | D00547 |
| 1 | hsa_55879 | D00548 |
| 1 | hsa_55879 | D00549 |
| 1 | hsa_55879 | D00550 |
| 1 | hsa_56479 | D00294 |
| 1 | hsa_56479 | D02356 |
| 1 | hsa_56659 | D00294 |
| 1 | hsa_56659 | D00542 |
| 1 | hsa_56659 | D02356 |
| 1 | hsa_56660 | D00542 |
| 1 | hsa_57053 | D00195 |
| 1 | hsa_57053 | D00499 |
| 1 | hsa_57053 | D00524 |
| 1 | hsa_57053 | D00611 |
| 1 | hsa_57053 | D00726 |
| 1 | hsa_57053 | D02101 |
| 1 | hsa_57053 | D02173 |
| 1 | hsa_57053 | D02204 |
| 1 | hsa_57053 | D02207 |
| 1 | hsa_57053 | D03826 |
| 1 | hsa_57053 | D05156 |
| 1 | hsa_57053 | D05453 |
| 1 | hsa_57192 | D00649 |
| 1 | hsa_57657 | D00294 |
| 1 | hsa_57657 | D02356 |
| 1 | hsa_59284 | D00332 |
| 1 | hsa_59284 | D00437 |
| 1 | hsa_59284 | D00512 |
| 1 | hsa_59284 | D00542 |
| 1 | hsa_59284 | D00560 |
| 1 | hsa_59284 | D00618 |
| 1 | hsa_59284 | D00629 |
| 1 | hsa_59284 | D01295 |
| 1 | hsa_59284 | D02356 |
| 1 | hsa_60598 | D00294 |
| 1 | hsa_60598 | D02356 |
| 1 | hsa_6323 | D00354 |
| 1 | hsa_6323 | D00358 |
| 1 | hsa_6323 | D00477 |
| 1 | hsa_6323 | D00504 |
| 1 | hsa_6323 | D00537 |
| 1 | hsa_6323 | D00538 |
| 1 | hsa_6323 | D00638 |
| 1 | hsa_6323 | D00639 |
| 1 | hsa_6323 | D00640 |
| 1 | hsa_6323 | D00642 |
| 1 | hsa_6323 | D00709 |
| 1 | hsa_6323 | D00732 |
| 1 | hsa_6323 | D00735 |
| 1 | hsa_6323 | D00738 |
| 1 | hsa_6323 | D00739 |
| 1 | hsa_6323 | D00740 |
| 1 | hsa_6323 | D00741 |
| 1 | hsa_6323 | D01243 |
| 1 | hsa_6323 | D01287 |
| 1 | hsa_6323 | D01450 |
| 1 | hsa_6323 | D02086 |
| 1 | hsa_6323 | D02087 |
| 1 | hsa_6323 | D02088 |
| 1 | hsa_6323 | D02098 |
| 1 | hsa_6323 | D02272 |
| 1 | hsa_6323 | D03991 |
| 1 | hsa_6324 | D00252 |
| 1 | hsa_6324 | D00303 |
| 1 | hsa_6324 | D00332 |
| 1 | hsa_6324 | D00512 |
| 1 | hsa_6324 | D00538 |
| 1 | hsa_6324 | D00552 |
| 1 | hsa_6324 | D00775 |
| 1 | hsa_6324 | D02356 |
| 1 | hsa_6326 | D00354 |
| 1 | hsa_6326 | D00358 |
| 1 | hsa_6326 | D00477 |
| 1 | hsa_6326 | D00638 |
| 1 | hsa_6326 | D00639 |
| 1 | hsa_6326 | D00640 |
| 1 | hsa_6326 | D00642 |
| 1 | hsa_6326 | D00732 |
| 1 | hsa_6326 | D00735 |
| 1 | hsa_6326 | D00738 |
| 1 | hsa_6326 | D00739 |
| 1 | hsa_6326 | D00740 |
| 1 | hsa_6326 | D00741 |
| 1 | hsa_6326 | D01243 |
| 1 | hsa_6326 | D01287 |
| 1 | hsa_6326 | D01450 |
| 1 | hsa_6326 | D02086 |
| 1 | hsa_6326 | D02087 |
| 1 | hsa_6326 | D02088 |
| 1 | hsa_6326 | D02098 |
| 1 | hsa_6326 | D02272 |
| 1 | hsa_6326 | D03991 |
| 1 | hsa_6328 | D00252 |
| 1 | hsa_6328 | D00303 |
| 1 | hsa_6328 | D00332 |
| 1 | hsa_6328 | D00354 |
| 1 | hsa_6328 | D00358 |
| 1 | hsa_6328 | D00477 |
| 1 | hsa_6328 | D00512 |
| 1 | hsa_6328 | D00538 |
| 1 | hsa_6328 | D00552 |
| 1 | hsa_6328 | D00638 |
| 1 | hsa_6328 | D00639 |
| 1 | hsa_6328 | D00640 |
| 1 | hsa_6328 | D00642 |
| 1 | hsa_6328 | D00732 |
| 1 | hsa_6328 | D00735 |
| 1 | hsa_6328 | D00738 |
| 1 | hsa_6328 | D00739 |
| 1 | hsa_6328 | D00740 |
| 1 | hsa_6328 | D00741 |
| 1 | hsa_6328 | D00775 |
| 1 | hsa_6328 | D01243 |
| 1 | hsa_6328 | D01287 |
| 1 | hsa_6328 | D01450 |
| 1 | hsa_6328 | D02086 |
| 1 | hsa_6328 | D02087 |
| 1 | hsa_6328 | D02088 |
| 1 | hsa_6328 | D02098 |
| 1 | hsa_6328 | D02272 |
| 1 | hsa_6328 | D02356 |
| 1 | hsa_6328 | D03991 |
| 1 | hsa_6329 | D00252 |
| 1 | hsa_6329 | D00303 |
| 1 | hsa_6329 | D00332 |
| 1 | hsa_6329 | D00354 |
| 1 | hsa_6329 | D00358 |
| 1 | hsa_6329 | D00477 |
| 1 | hsa_6329 | D00512 |
| 1 | hsa_6329 | D00538 |
| 1 | hsa_6329 | D00552 |
| 1 | hsa_6329 | D00638 |
| 1 | hsa_6329 | D00639 |
| 1 | hsa_6329 | D00640 |
| 1 | hsa_6329 | D00642 |
| 1 | hsa_6329 | D00732 |
| 1 | hsa_6329 | D00735 |
| 1 | hsa_6329 | D00738 |
| 1 | hsa_6329 | D00739 |
| 1 | hsa_6329 | D00740 |
| 1 | hsa_6329 | D00741 |
| 1 | hsa_6329 | D00775 |
| 1 | hsa_6329 | D01243 |
| 1 | hsa_6329 | D01287 |
| 1 | hsa_6329 | D01450 |
| 1 | hsa_6329 | D02086 |
| 1 | hsa_6329 | D02087 |
| 1 | hsa_6329 | D02088 |
| 1 | hsa_6329 | D02098 |
| 1 | hsa_6329 | D02272 |
| 1 | hsa_6329 | D02356 |
| 1 | hsa_6329 | D03991 |
| 1 | hsa_6330 | D00252 |
| 1 | hsa_6330 | D00303 |
| 1 | hsa_6330 | D00332 |
| 1 | hsa_6330 | D00512 |
| 1 | hsa_6330 | D00538 |
| 1 | hsa_6330 | D00552 |
| 1 | hsa_6330 | D00775 |
| 1 | hsa_6330 | D02356 |
| 1 | hsa_6331 | D00110 |
| 1 | hsa_6331 | D00252 |
| 1 | hsa_6331 | D00303 |
| 1 | hsa_6331 | D00332 |
| 1 | hsa_6331 | D00354 |
| 1 | hsa_6331 | D00358 |
| 1 | hsa_6331 | D00477 |
| 1 | hsa_6331 | D00512 |
| 1 | hsa_6331 | D00533 |
| 1 | hsa_6331 | D00553 |
| 1 | hsa_6331 | D00638 |
| 1 | hsa_6331 | D00639 |
| 1 | hsa_6331 | D00640 |
| 1 | hsa_6331 | D00642 |
| 1 | hsa_6331 | D00708 |
| 1 | hsa_6331 | D00732 |
| 1 | hsa_6331 | D00733 |
| 1 | hsa_6331 | D00735 |
| 1 | hsa_6331 | D00738 |
| 1 | hsa_6331 | D00739 |
| 1 | hsa_6331 | D00740 |
| 1 | hsa_6331 | D00741 |
| 1 | hsa_6331 | D00775 |
| 1 | hsa_6331 | D01243 |
| 1 | hsa_6331 | D01287 |
| 1 | hsa_6331 | D01450 |
| 1 | hsa_6331 | D02086 |
| 1 | hsa_6331 | D02087 |
| 1 | hsa_6331 | D02088 |
| 1 | hsa_6331 | D02098 |
| 1 | hsa_6331 | D02272 |
| 1 | hsa_6331 | D02356 |
| 1 | hsa_6331 | D03991 |
| 1 | hsa_6331 | D05077 |
| 1 | hsa_6331 | D06172 |
| 1 | hsa_6332 | D00252 |
| 1 | hsa_6332 | D00303 |
| 1 | hsa_6332 | D00332 |
| 1 | hsa_6332 | D00512 |
| 1 | hsa_6332 | D00538 |
| 1 | hsa_6332 | D00552 |
| 1 | hsa_6332 | D00775 |
| 1 | hsa_6332 | D02356 |
| 1 | hsa_6334 | D00354 |
| 1 | hsa_6334 | D00358 |
| 1 | hsa_6334 | D00477 |
| 1 | hsa_6334 | D00638 |
| 1 | hsa_6334 | D00639 |
| 1 | hsa_6334 | D00640 |
| 1 | hsa_6334 | D00642 |
| 1 | hsa_6334 | D00732 |
| 1 | hsa_6334 | D00735 |
| 1 | hsa_6334 | D00738 |
| 1 | hsa_6334 | D00739 |
| 1 | hsa_6334 | D00740 |
| 1 | hsa_6334 | D00741 |
| 1 | hsa_6334 | D01243 |
| 1 | hsa_6334 | D01287 |
| 1 | hsa_6334 | D01450 |
| 1 | hsa_6334 | D02086 |
| 1 | hsa_6334 | D02087 |
| 1 | hsa_6334 | D02088 |
| 1 | hsa_6334 | D02098 |
| 1 | hsa_6334 | D02272 |
| 1 | hsa_6334 | D03991 |
| 1 | hsa_6335 | D00354 |
| 1 | hsa_6335 | D00358 |
| 1 | hsa_6335 | D00477 |
| 1 | hsa_6335 | D00638 |
| 1 | hsa_6335 | D00639 |
| 1 | hsa_6335 | D00640 |
| 1 | hsa_6335 | D00642 |
| 1 | hsa_6335 | D00732 |
| 1 | hsa_6335 | D00735 |
| 1 | hsa_6335 | D00738 |
| 1 | hsa_6335 | D00739 |
| 1 | hsa_6335 | D00740 |
| 1 | hsa_6335 | D00741 |
| 1 | hsa_6335 | D01243 |
| 1 | hsa_6335 | D01287 |
| 1 | hsa_6335 | D01450 |
| 1 | hsa_6335 | D02086 |
| 1 | hsa_6335 | D02087 |
| 1 | hsa_6335 | D02088 |
| 1 | hsa_6335 | D02098 |
| 1 | hsa_6335 | D02272 |
| 1 | hsa_6335 | D03991 |
| 1 | hsa_6336 | D00110 |
| 1 | hsa_6336 | D00252 |
| 1 | hsa_6336 | D00303 |
| 1 | hsa_6336 | D00332 |
| 1 | hsa_6336 | D00358 |
| 1 | hsa_6336 | D00512 |
| 1 | hsa_6336 | D00538 |
| 1 | hsa_6336 | D00552 |
| 1 | hsa_6336 | D00732 |
| 1 | hsa_6336 | D00733 |
| 1 | hsa_6336 | D00735 |
| 1 | hsa_6336 | D00738 |
| 1 | hsa_6336 | D00739 |
| 1 | hsa_6336 | D00740 |
| 1 | hsa_6336 | D00741 |
| 1 | hsa_6336 | D00775 |
| 1 | hsa_6336 | D01243 |
| 1 | hsa_6336 | D01287 |
| 1 | hsa_6336 | D01450 |
| 1 | hsa_6336 | D01768 |
| 1 | hsa_6336 | D02086 |
| 1 | hsa_6336 | D02098 |
| 1 | hsa_6336 | D02356 |
| 1 | hsa_6337 | D00252 |
| 1 | hsa_6337 | D00303 |
| 1 | hsa_6337 | D00332 |
| 1 | hsa_6337 | D00512 |
| 1 | hsa_6337 | D00538 |
| 1 | hsa_6337 | D00552 |
| 1 | hsa_6337 | D00649 |
| 1 | hsa_6337 | D00775 |
| 1 | hsa_6337 | D02356 |
| 1 | hsa_6338 | D00252 |
| 1 | hsa_6338 | D00303 |
| 1 | hsa_6338 | D00332 |
| 1 | hsa_6338 | D00512 |
| 1 | hsa_6338 | D00538 |
| 1 | hsa_6338 | D00552 |
| 1 | hsa_6338 | D00649 |
| 1 | hsa_6338 | D00775 |
| 1 | hsa_6338 | D02356 |
| 1 | hsa_6339 | D00252 |
| 1 | hsa_6339 | D00303 |
| 1 | hsa_6339 | D00332 |
| 1 | hsa_6339 | D00512 |
| 1 | hsa_6339 | D00538 |
| 1 | hsa_6339 | D00552 |
| 1 | hsa_6339 | D00649 |
| 1 | hsa_6339 | D00775 |
| 1 | hsa_6339 | D02356 |
| 1 | hsa_6340 | D00252 |
| 1 | hsa_6340 | D00303 |
| 1 | hsa_6340 | D00332 |
| 1 | hsa_6340 | D00512 |
| 1 | hsa_6340 | D00538 |
| 1 | hsa_6340 | D00552 |
| 1 | hsa_6340 | D00649 |
| 1 | hsa_6340 | D00775 |
| 1 | hsa_6340 | D02356 |
| 1 | hsa_6529 | D00110 |
| 1 | hsa_6530 | D00110 |
| 1 | hsa_6530 | D00228 |
| 1 | hsa_6530 | D00367 |
| 1 | hsa_6530 | D00394 |
| 1 | hsa_6530 | D00607 |
| 1 | hsa_6530 | D01603 |
| 1 | hsa_6530 | D02237 |
| 1 | hsa_6530 | D02566 |
| 1 | hsa_6530 | D04999 |
| 1 | hsa_6530 | D05458 |
| 1 | hsa_6531 | D00110 |
| 1 | hsa_6531 | D00367 |
| 1 | hsa_6531 | D04999 |
| 1 | hsa_6531 | D05458 |
| 1 | hsa_6532 | D00110 |
| 1 | hsa_6532 | D00228 |
| 1 | hsa_6532 | D00394 |
| 1 | hsa_6532 | D02360 |
| 1 | hsa_6532 | D02362 |
| 1 | hsa_6532 | D03742 |
| 1 | hsa_6534 | D00035 |
| 1 | hsa_6833 | D00294 |
| 1 | hsa_6833 | D00336 |
| 1 | hsa_6833 | D00380 |
| 1 | hsa_6833 | D01828 |
| 1 | hsa_7225 | D00649 |
| 1 | hsa_773 | D00319 |
| 1 | hsa_773 | D00332 |
| 1 | hsa_773 | D00437 |
| 1 | hsa_773 | D00512 |
| 1 | hsa_773 | D00542 |
| 1 | hsa_773 | D00560 |
| 1 | hsa_773 | D00615 |
| 1 | hsa_773 | D00617 |
| 1 | hsa_773 | D00618 |
| 1 | hsa_773 | D00629 |
| 1 | hsa_773 | D01295 |
| 1 | hsa_773 | D02356 |
| 1 | hsa_773 | D02914 |
| 1 | hsa_773 | D05024 |
| 1 | hsa_774 | D00332 |
| 1 | hsa_774 | D00437 |
| 1 | hsa_774 | D00512 |
| 1 | hsa_774 | D00542 |
| 1 | hsa_774 | D00560 |
| 1 | hsa_774 | D00618 |
| 1 | hsa_774 | D00629 |
| 1 | hsa_774 | D01295 |
| 1 | hsa_774 | D02356 |
| 1 | hsa_775 | D00319 |
| 1 | hsa_775 | D00332 |
| 1 | hsa_775 | D00349 |
| 1 | hsa_775 | D00437 |
| 1 | hsa_775 | D00438 |
| 1 | hsa_775 | D00512 |
| 1 | hsa_775 | D00542 |
| 1 | hsa_775 | D00560 |
| 1 | hsa_775 | D00615 |
| 1 | hsa_775 | D00616 |
| 1 | hsa_775 | D00617 |
| 1 | hsa_775 | D00618 |
| 1 | hsa_775 | D00619 |
| 1 | hsa_775 | D00629 |
| 1 | hsa_775 | D00648 |
| 1 | hsa_775 | D01108 |
| 1 | hsa_775 | D01295 |
| 1 | hsa_775 | D01969 |
| 1 | hsa_775 | D02356 |
| 1 | hsa_775 | D02914 |
| 1 | hsa_775 | D03830 |
| 1 | hsa_776 | D00319 |
| 1 | hsa_776 | D00332 |
| 1 | hsa_776 | D00349 |
| 1 | hsa_776 | D00437 |
| 1 | hsa_776 | D00438 |
| 1 | hsa_776 | D00512 |
| 1 | hsa_776 | D00542 |
| 1 | hsa_776 | D00560 |
| 1 | hsa_776 | D00615 |
| 1 | hsa_776 | D00617 |
| 1 | hsa_776 | D00618 |
| 1 | hsa_776 | D00629 |
| 1 | hsa_776 | D01295 |
| 1 | hsa_776 | D02356 |
| 1 | hsa_776 | D02914 |
| 1 | hsa_777 | D00332 |
| 1 | hsa_777 | D00437 |
| 1 | hsa_777 | D00512 |
| 1 | hsa_777 | D00542 |
| 1 | hsa_777 | D00560 |
| 1 | hsa_777 | D00618 |
| 1 | hsa_777 | D00629 |
| 1 | hsa_777 | D01295 |
| 1 | hsa_777 | D02356 |
| 1 | hsa_777 | D05024 |
| 1 | hsa_778 | D00319 |
| 1 | hsa_778 | D00332 |
| 1 | hsa_778 | D00349 |
| 1 | hsa_778 | D00437 |
| 1 | hsa_778 | D00438 |
| 1 | hsa_778 | D00512 |
| 1 | hsa_778 | D00542 |
| 1 | hsa_778 | D00560 |
| 1 | hsa_778 | D00615 |
| 1 | hsa_778 | D00616 |
| 1 | hsa_778 | D00617 |
| 1 | hsa_778 | D00618 |
| 1 | hsa_778 | D00629 |
| 1 | hsa_778 | D01295 |
| 1 | hsa_778 | D02356 |
| 1 | hsa_778 | D02914 |
| 1 | hsa_778 | D03830 |
| 1 | hsa_779 | D00319 |
| 1 | hsa_779 | D00332 |
| 1 | hsa_779 | D00349 |
| 1 | hsa_779 | D00437 |
| 1 | hsa_779 | D00512 |
| 1 | hsa_779 | D00542 |
| 1 | hsa_779 | D00560 |
| 1 | hsa_779 | D00615 |
| 1 | hsa_779 | D00616 |
| 1 | hsa_779 | D00617 |
| 1 | hsa_779 | D00618 |
| 1 | hsa_779 | D00619 |
| 1 | hsa_779 | D00629 |
| 1 | hsa_779 | D01295 |
| 1 | hsa_779 | D01969 |
| 1 | hsa_779 | D02356 |
| 1 | hsa_779 | D02914 |
| 1 | hsa_779 | D03830 |
| 1 | hsa_781 | D00332 |
| 1 | hsa_781 | D00349 |
| 1 | hsa_781 | D00437 |
| 1 | hsa_781 | D00512 |
| 1 | hsa_781 | D00542 |
| 1 | hsa_781 | D00560 |
| 1 | hsa_781 | D00618 |
| 1 | hsa_781 | D00629 |
| 1 | hsa_781 | D00648 |
| 1 | hsa_781 | D01108 |
| 1 | hsa_781 | D01295 |
| 1 | hsa_781 | D02356 |
| 1 | hsa_782 | D00332 |
| 1 | hsa_782 | D00437 |
| 1 | hsa_782 | D00512 |
| 1 | hsa_782 | D00542 |
| 1 | hsa_782 | D00560 |
| 1 | hsa_782 | D00618 |
| 1 | hsa_782 | D00629 |
| 1 | hsa_782 | D00648 |
| 1 | hsa_782 | D01295 |
| 1 | hsa_782 | D02356 |
| 1 | hsa_783 | D00332 |
| 1 | hsa_783 | D00349 |
| 1 | hsa_783 | D00437 |
| 1 | hsa_783 | D00438 |
| 1 | hsa_783 | D00512 |
| 1 | hsa_783 | D00542 |
| 1 | hsa_783 | D00560 |
| 1 | hsa_783 | D00618 |
| 1 | hsa_783 | D00629 |
| 1 | hsa_783 | D01295 |
| 1 | hsa_783 | D02356 |
| 1 | hsa_784 | D00332 |
| 1 | hsa_784 | D00437 |
| 1 | hsa_784 | D00512 |
| 1 | hsa_784 | D00542 |
| 1 | hsa_784 | D00560 |
| 1 | hsa_784 | D00618 |
| 1 | hsa_784 | D00629 |
| 1 | hsa_784 | D01295 |
| 1 | hsa_784 | D02356 |
| 1 | hsa_785 | D00332 |
| 1 | hsa_785 | D00437 |
| 1 | hsa_785 | D00512 |
| 1 | hsa_785 | D00542 |
| 1 | hsa_785 | D00560 |
| 1 | hsa_785 | D00618 |
| 1 | hsa_785 | D00629 |
| 1 | hsa_785 | D01295 |
| 1 | hsa_785 | D02356 |
| 1 | hsa_786 | D00332 |
| 1 | hsa_786 | D00349 |
| 1 | hsa_786 | D00437 |
| 1 | hsa_786 | D00438 |
| 1 | hsa_786 | D00512 |
| 1 | hsa_786 | D00542 |
| 1 | hsa_786 | D00560 |
| 1 | hsa_786 | D00618 |
| 1 | hsa_786 | D00629 |
| 1 | hsa_786 | D00648 |
| 1 | hsa_786 | D01108 |
| 1 | hsa_786 | D01295 |
| 1 | hsa_786 | D02356 |
| 1 | hsa_7881 | D00294 |
| 1 | hsa_7881 | D02356 |
| 1 | hsa_8001 | D02041 |
| 1 | hsa_81033 | D00294 |
| 1 | hsa_81033 | D00648 |
| 1 | hsa_81033 | D02356 |
| 1 | hsa_83795 | D00294 |
| 1 | hsa_83795 | D00642 |
| 1 | hsa_83795 | D02272 |
| 1 | hsa_83795 | D02356 |
| 1 | hsa_8514 | D00294 |
| 1 | hsa_8514 | D02356 |
| 1 | hsa_8645 | D00294 |
| 1 | hsa_8645 | D00358 |
| 1 | hsa_8645 | D00640 |
| 1 | hsa_8645 | D00642 |
| 1 | hsa_8645 | D00732 |
| 1 | hsa_8645 | D00735 |
| 1 | hsa_8645 | D00738 |
| 1 | hsa_8645 | D00739 |
| 1 | hsa_8645 | D00740 |
| 1 | hsa_8645 | D00741 |
| 1 | hsa_8645 | D01243 |
| 1 | hsa_8645 | D01287 |
| 1 | hsa_8645 | D01450 |
| 1 | hsa_8645 | D02086 |
| 1 | hsa_8645 | D02272 |
| 1 | hsa_8645 | D02356 |
| 1 | hsa_8911 | D00332 |
| 1 | hsa_8911 | D00349 |
| 1 | hsa_8911 | D00437 |
| 1 | hsa_8911 | D00495 |
| 1 | hsa_8911 | D00512 |
| 1 | hsa_8911 | D00538 |
| 1 | hsa_8911 | D00539 |
| 1 | hsa_8911 | D00542 |
| 1 | hsa_8911 | D00560 |
| 1 | hsa_8911 | D00618 |
| 1 | hsa_8911 | D00629 |
| 1 | hsa_8911 | D01295 |
| 1 | hsa_8911 | D02356 |
| 1 | hsa_8911 | D02630 |
| 1 | hsa_8911 | D05024 |
| 1 | hsa_8912 | D00332 |
| 1 | hsa_8912 | D00349 |
| 1 | hsa_8912 | D00437 |
| 1 | hsa_8912 | D00512 |
| 1 | hsa_8912 | D00538 |
| 1 | hsa_8912 | D00539 |
| 1 | hsa_8912 | D00542 |
| 1 | hsa_8912 | D00560 |
| 1 | hsa_8912 | D00618 |
| 1 | hsa_8912 | D00629 |
| 1 | hsa_8912 | D00649 |
| 1 | hsa_8912 | D01295 |
| 1 | hsa_8912 | D02356 |
| 1 | hsa_8912 | D02630 |
| 1 | hsa_8912 | D05024 |
| 1 | hsa_8913 | D00332 |
| 1 | hsa_8913 | D00349 |
| 1 | hsa_8913 | D00392 |
| 1 | hsa_8913 | D00437 |
| 1 | hsa_8913 | D00512 |
| 1 | hsa_8913 | D00538 |
| 1 | hsa_8913 | D00539 |
| 1 | hsa_8913 | D00542 |
| 1 | hsa_8913 | D00560 |
| 1 | hsa_8913 | D00618 |
| 1 | hsa_8913 | D00629 |
| 1 | hsa_8913 | D00649 |
| 1 | hsa_8913 | D00709 |
| 1 | hsa_8913 | D01295 |
| 1 | hsa_8913 | D02356 |
| 1 | hsa_8913 | D02630 |
| 1 | hsa_8913 | D05024 |
| 1 | hsa_8973 | D00195 |
| 1 | hsa_8973 | D00499 |
| 1 | hsa_8973 | D00524 |
| 1 | hsa_8973 | D00611 |
| 1 | hsa_8973 | D00726 |
| 1 | hsa_8973 | D02101 |
| 1 | hsa_8973 | D02173 |
| 1 | hsa_8973 | D02204 |
| 1 | hsa_8973 | D02207 |
| 1 | hsa_8973 | D03826 |
| 1 | hsa_8973 | D05156 |
| 1 | hsa_8973 | D05453 |
| 1 | hsa_89822 | D00294 |
| 1 | hsa_89822 | D02356 |
| 1 | hsa_8989 | D00332 |
| 1 | hsa_8989 | D00437 |
| 1 | hsa_8989 | D00512 |
| 1 | hsa_8989 | D00542 |
| 1 | hsa_8989 | D00560 |
| 1 | hsa_8989 | D00618 |
| 1 | hsa_8989 | D00629 |
| 1 | hsa_8989 | D01295 |
| 1 | hsa_8989 | D02356 |
| 1 | hsa_90134 | D00294 |
| 1 | hsa_90134 | D00648 |
| 1 | hsa_90134 | D02356 |
| 1 | hsa_9127 | D00528 |
| 1 | hsa_9132 | D00294 |
| 1 | hsa_9132 | D00631 |
| 1 | hsa_9132 | D02356 |
| 1 | hsa_9177 | D00283 |
| 1 | hsa_9177 | D00451 |
| 1 | hsa_9177 | D00513 |
| 1 | hsa_9177 | D00633 |
| 1 | hsa_9177 | D00677 |
| 1 | hsa_9177 | D00678 |
| 1 | hsa_9177 | D00726 |
| 1 | hsa_9177 | D02041 |
| 1 | hsa_9254 | D00332 |
| 1 | hsa_9254 | D00437 |
| 1 | hsa_9254 | D00512 |
| 1 | hsa_9254 | D00542 |
| 1 | hsa_9254 | D00560 |
| 1 | hsa_9254 | D00618 |
| 1 | hsa_9254 | D00629 |
| 1 | hsa_9254 | D01295 |
| 1 | hsa_9254 | D02356 |
| 1 | hsa_93107 | D00294 |
| 1 | hsa_93107 | D02356 |
| 1 | hsa_9311 | D00252 |
| 1 | hsa_9311 | D00303 |
| 1 | hsa_9311 | D00332 |
| 1 | hsa_9311 | D00512 |
| 1 | hsa_9311 | D00538 |
| 1 | hsa_9311 | D00552 |
| 1 | hsa_9311 | D00649 |
| 1 | hsa_9311 | D00775 |
| 1 | hsa_9311 | D02356 |
| 1 | hsa_9312 | D00294 |
| 1 | hsa_9312 | D02261 |
| 1 | hsa_9312 | D02262 |
| 1 | hsa_9312 | D02356 |
| 1 | hsa_9312 | D05453 |
| 1 | hsa_93589 | D00332 |
| 1 | hsa_93589 | D00349 |
| 1 | hsa_93589 | D00437 |
| 1 | hsa_93589 | D00438 |
| 1 | hsa_93589 | D00512 |
| 1 | hsa_93589 | D00542 |
| 1 | hsa_93589 | D00560 |
| 1 | hsa_93589 | D00618 |
| 1 | hsa_93589 | D00629 |
| 1 | hsa_93589 | D01295 |
| 1 | hsa_93589 | D02356 |
| 1 | hsa_9424 | D00294 |
| 1 | hsa_9424 | D00642 |
| 1 | hsa_9424 | D00648 |
| 1 | hsa_9424 | D02272 |
| 1 | hsa_9424 | D02356 |
| 1 | hsa_9992 | D00294 |
| 1 | hsa_9992 | D02356 |
| 2 | hsa_10008 | D00274 |
| 2 | hsa_10008 | D00394 |
| 2 | hsa_10008 | D00418 |
| 2 | hsa_10008 | D00538 |
| 2 | hsa_10008 | D00638 |
| 2 | hsa_10008 | D00647 |
| 2 | hsa_10008 | D00738 |
| 2 | hsa_10008 | D00741 |
| 2 | hsa_10008 | D00771 |
| 2 | hsa_10008 | D01108 |
| 2 | hsa_10008 | D01295 |
| 2 | hsa_10008 | D01712 |
| 2 | hsa_10008 | D01828 |
| 2 | hsa_10008 | D02204 |
| 2 | hsa_10008 | D03180 |
| 2 | hsa_10008 | D03274 |
| 2 | hsa_10060 | D00283 |
| 2 | hsa_10060 | D00336 |
| 2 | hsa_10060 | D00338 |
| 2 | hsa_10060 | D00349 |
| 2 | hsa_10060 | D00380 |
| 2 | hsa_10060 | D00615 |
| 2 | hsa_10060 | D00658 |
| 2 | hsa_10060 | D00694 |
| 2 | hsa_10060 | D00697 |
| 2 | hsa_10060 | D00812 |
| 2 | hsa_10369 | D00283 |
| 2 | hsa_10369 | D00326 |
| 2 | hsa_10369 | D00521 |
| 2 | hsa_10369 | D00528 |
| 2 | hsa_10369 | D00543 |
| 2 | hsa_10369 | D00544 |
| 2 | hsa_10369 | D00552 |
| 2 | hsa_10369 | D00616 |
| 2 | hsa_10369 | D00726 |
| 2 | hsa_10369 | D00775 |
| 2 | hsa_10369 | D00964 |
| 2 | hsa_10369 | D01243 |
| 2 | hsa_1080 | D00234 |
| 2 | hsa_1080 | D00283 |
| 2 | hsa_1080 | D00345 |
| 2 | hsa_1080 | D00380 |
| 2 | hsa_1080 | D00477 |
| 2 | hsa_1080 | D00504 |
| 2 | hsa_1080 | D00513 |
| 2 | hsa_1080 | D00611 |
| 2 | hsa_1080 | D00648 |
| 2 | hsa_1080 | D00677 |
| 2 | hsa_1080 | D01071 |
| 2 | hsa_1080 | D01295 |
| 2 | hsa_1080 | D02098 |
| 2 | hsa_1080 | D02630 |
| 2 | hsa_1080 | D02914 |
| 2 | hsa_1080 | D05077 |
| 2 | hsa_1080 | D05458 |
| 2 | hsa_1080 | D06172 |
| 2 | hsa_11254 | D00110 |
| 2 | hsa_11254 | D00524 |
| 2 | hsa_11254 | D00574 |
| 2 | hsa_11254 | D00656 |
| 2 | hsa_11254 | D00775 |
| 2 | hsa_11254 | D00960 |
| 2 | hsa_11254 | D01828 |
| 2 | hsa_11254 | D02630 |
| 2 | hsa_11280 | D00035 |
| 2 | hsa_11280 | D00228 |
| 2 | hsa_11280 | D00340 |
| 2 | hsa_11280 | D00379 |
| 2 | hsa_11280 | D00392 |
| 2 | hsa_11280 | D00394 |
| 2 | hsa_11280 | D00451 |
| 2 | hsa_11280 | D00474 |
| 2 | hsa_11280 | D00544 |
| 2 | hsa_11280 | D00560 |
| 2 | hsa_11280 | D00638 |
| 2 | hsa_11280 | D00639 |
| 2 | hsa_11280 | D00709 |
| 2 | hsa_11280 | D00711 |
| 2 | hsa_11280 | D00760 |
| 2 | hsa_11280 | D00823 |
| 2 | hsa_11280 | D01111 |
| 2 | hsa_11280 | D01295 |
| 2 | hsa_11280 | D01854 |
| 2 | hsa_11280 | D02360 |
| 2 | hsa_1134 | D00228 |
| 2 | hsa_1134 | D00303 |
| 2 | hsa_1134 | D00364 |
| 2 | hsa_1134 | D00618 |
| 2 | hsa_1134 | D00636 |
| 2 | hsa_1134 | D00656 |
| 2 | hsa_1134 | D00709 |
| 2 | hsa_1134 | D00738 |
| 2 | hsa_1134 | D00760 |
| 2 | hsa_1134 | D00816 |
| 2 | hsa_1134 | D01071 |
| 2 | hsa_1134 | D02237 |
| 2 | hsa_1134 | D02261 |
| 2 | hsa_1134 | D02630 |
| 2 | hsa_1134 | D05458 |
| 2 | hsa_1135 | D00035 |
| 2 | hsa_1135 | D00331 |
| 2 | hsa_1135 | D00335 |
| 2 | hsa_1135 | D00530 |
| 2 | hsa_1135 | D00548 |
| 2 | hsa_1135 | D00678 |
| 2 | hsa_1135 | D00732 |
| 2 | hsa_1135 | D01256 |
| 2 | hsa_1135 | D01768 |
| 2 | hsa_1135 | D02041 |
| 2 | hsa_1135 | D02566 |
| 2 | hsa_1135 | D04790 |
| 2 | hsa_1135 | D05461 |
| 2 | hsa_1136 | D00225 |
| 2 | hsa_1136 | D00335 |
| 2 | hsa_1136 | D00418 |
| 2 | hsa_1136 | D00521 |
| 2 | hsa_1136 | D00738 |
| 2 | hsa_1136 | D00760 |
| 2 | hsa_1136 | D01603 |
| 2 | hsa_1136 | D01854 |
| 2 | hsa_1136 | D02086 |
| 2 | hsa_1136 | D02092 |
| 2 | hsa_1136 | D02360 |
| 2 | hsa_1136 | D04790 |
| 2 | hsa_1137 | D00035 |
| 2 | hsa_1137 | D00136 |
| 2 | hsa_1137 | D00351 |
| 2 | hsa_1137 | D00618 |
| 2 | hsa_1137 | D00631 |
| 2 | hsa_1137 | D00638 |
| 2 | hsa_1137 | D00640 |
| 2 | hsa_1137 | D00649 |
| 2 | hsa_1137 | D00651 |
| 2 | hsa_1137 | D00732 |
| 2 | hsa_1137 | D01253 |
| 2 | hsa_1137 | D01712 |
| 2 | hsa_1137 | D02092 |
| 2 | hsa_1137 | D02234 |
| 2 | hsa_1137 | D02461 |
| 2 | hsa_1138 | D00519 |
| 2 | hsa_1138 | D00533 |
| 2 | hsa_1138 | D00548 |
| 2 | hsa_1138 | D00549 |
| 2 | hsa_1138 | D00697 |
| 2 | hsa_1138 | D00739 |
| 2 | hsa_1138 | D00741 |
| 2 | hsa_1138 | D02086 |
| 2 | hsa_1138 | D02092 |
| 2 | hsa_1139 | D00294 |
| 2 | hsa_1139 | D00504 |
| 2 | hsa_1139 | D00530 |
| 2 | hsa_1139 | D00547 |
| 2 | hsa_1139 | D00639 |
| 2 | hsa_1139 | D00640 |
| 2 | hsa_1139 | D00740 |
| 2 | hsa_1139 | D00823 |
| 2 | hsa_1139 | D01242 |
| 2 | hsa_1139 | D02163 |
| 2 | hsa_1139 | D02253 |
| 2 | hsa_1139 | D06106 |
| 2 | hsa_1140 | D00380 |
| 2 | hsa_1140 | D00512 |
| 2 | hsa_1140 | D00543 |
| 2 | hsa_1140 | D00545 |
| 2 | hsa_1140 | D00560 |
| 2 | hsa_1140 | D00638 |
| 2 | hsa_1140 | D00711 |
| 2 | hsa_1140 | D00735 |
| 2 | hsa_1140 | D00823 |
| 2 | hsa_1140 | D01448 |
| 2 | hsa_1140 | D02485 |
| 2 | hsa_1140 | D03830 |
| 2 | hsa_1141 | D00219 |
| 2 | hsa_1141 | D00474 |
| 2 | hsa_1141 | D00548 |
| 2 | hsa_1141 | D00616 |
| 2 | hsa_1141 | D00619 |
| 2 | hsa_1141 | D00640 |
| 2 | hsa_1141 | D01575 |
| 2 | hsa_1141 | D01799 |
| 2 | hsa_1141 | D02098 |
| 2 | hsa_1141 | D02204 |
| 2 | hsa_1141 | D02261 |
| 2 | hsa_1141 | D02272 |
| 2 | hsa_1141 | D04985 |
| 2 | hsa_1141 | D05453 |
| 2 | hsa_1144 | D00274 |
| 2 | hsa_1144 | D00283 |
| 2 | hsa_1144 | D00319 |
| 2 | hsa_1144 | D00367 |
| 2 | hsa_1144 | D00504 |
| 2 | hsa_1144 | D00544 |
| 2 | hsa_1144 | D00553 |
| 2 | hsa_1144 | D00593 |
| 2 | hsa_1144 | D00619 |
| 2 | hsa_1144 | D00735 |
| 2 | hsa_1144 | D00761 |
| 2 | hsa_1144 | D01071 |
| 2 | hsa_1144 | D01111 |
| 2 | hsa_1144 | D01118 |
| 2 | hsa_1144 | D04034 |
| 2 | hsa_1144 | D04790 |
| 2 | hsa_1145 | D00136 |
| 2 | hsa_1145 | D00195 |
| 2 | hsa_1145 | D00219 |
| 2 | hsa_1145 | D00319 |
| 2 | hsa_1145 | D00351 |
| 2 | hsa_1145 | D00379 |
| 2 | hsa_1145 | D00504 |
| 2 | hsa_1145 | D00513 |
| 2 | hsa_1145 | D00521 |
| 2 | hsa_1145 | D00530 |
| 2 | hsa_1145 | D00738 |
| 2 | hsa_1145 | D00760 |
| 2 | hsa_1145 | D01712 |
| 2 | hsa_1145 | D02253 |
| 2 | hsa_1145 | D02461 |
| 2 | hsa_1145 | D02485 |
| 2 | hsa_1145 | D02630 |
| 2 | hsa_1145 | D03037 |
| 2 | hsa_116443 | D00035 |
| 2 | hsa_116443 | D00331 |
| 2 | hsa_116443 | D00545 |
| 2 | hsa_116443 | D00546 |
| 2 | hsa_116443 | D00629 |
| 2 | hsa_116443 | D00677 |
| 2 | hsa_116443 | D00831 |
| 2 | hsa_116443 | D01854 |
| 2 | hsa_116443 | D02098 |
| 2 | hsa_116443 | D02409 |
| 2 | hsa_116443 | D02914 |
| 2 | hsa_116444 | D00326 |
| 2 | hsa_116444 | D00336 |
| 2 | hsa_116444 | D00536 |
| 2 | hsa_116444 | D00648 |
| 2 | hsa_116444 | D00739 |
| 2 | hsa_116444 | D01603 |
| 2 | hsa_116444 | D01768 |
| 2 | hsa_116444 | D01969 |
| 2 | hsa_116444 | D02409 |
| 2 | hsa_1181 | D00228 |
| 2 | hsa_1181 | D00354 |
| 2 | hsa_1181 | D00504 |
| 2 | hsa_1181 | D00546 |
| 2 | hsa_1181 | D00553 |
| 2 | hsa_1181 | D00647 |
| 2 | hsa_1181 | D00708 |
| 2 | hsa_1181 | D00709 |
| 2 | hsa_1181 | D00735 |
| 2 | hsa_1181 | D00798 |
| 2 | hsa_1181 | D02207 |
| 2 | hsa_1181 | D02485 |
| 2 | hsa_1181 | D03742 |
| 2 | hsa_1181 | D03878 |
| 2 | hsa_1181 | D04985 |
| 2 | hsa_1181 | D05458 |
| 2 | hsa_169522 | D00274 |
| 2 | hsa_169522 | D00340 |
| 2 | hsa_169522 | D00418 |
| 2 | hsa_169522 | D00544 |
| 2 | hsa_169522 | D00550 |
| 2 | hsa_169522 | D00740 |
| 2 | hsa_169522 | D00741 |
| 2 | hsa_169522 | D00775 |
| 2 | hsa_169522 | D00798 |
| 2 | hsa_169522 | D01712 |
| 2 | hsa_169522 | D02360 |
| 2 | hsa_169522 | D03450 |
| 2 | hsa_169522 | D05453 |
| 2 | hsa_170572 | D00234 |
| 2 | hsa_170572 | D00252 |
| 2 | hsa_170572 | D00274 |
| 2 | hsa_170572 | D00294 |
| 2 | hsa_170572 | D00326 |
| 2 | hsa_170572 | D00349 |
| 2 | hsa_170572 | D00351 |
| 2 | hsa_170572 | D00364 |
| 2 | hsa_170572 | D00373 |
| 2 | hsa_170572 | D00521 |
| 2 | hsa_170572 | D00593 |
| 2 | hsa_170572 | D00638 |
| 2 | hsa_170572 | D00711 |
| 2 | hsa_170572 | D00726 |
| 2 | hsa_170572 | D00775 |
| 2 | hsa_170572 | D01854 |
| 2 | hsa_170572 | D02253 |
| 2 | hsa_170572 | D03830 |
| 2 | hsa_170572 | D05458 |
| 2 | hsa_170572 | D05461 |
| 2 | hsa_200909 | D00110 |
| 2 | hsa_200909 | D00329 |
| 2 | hsa_200909 | D00480 |
| 2 | hsa_200909 | D00512 |
| 2 | hsa_200909 | D00560 |
| 2 | hsa_200909 | D00654 |
| 2 | hsa_200909 | D00694 |
| 2 | hsa_200909 | D00709 |
| 2 | hsa_200909 | D00711 |
| 2 | hsa_200909 | D00760 |
| 2 | hsa_200909 | D00964 |
| 2 | hsa_200909 | D01111 |
| 2 | hsa_200909 | D02086 |
| 2 | hsa_200909 | D05461 |
| 2 | hsa_22953 | D00480 |
| 2 | hsa_22953 | D00512 |
| 2 | hsa_22953 | D00519 |
| 2 | hsa_22953 | D00544 |
| 2 | hsa_22953 | D00549 |
| 2 | hsa_22953 | D00654 |
| 2 | hsa_22953 | D00726 |
| 2 | hsa_22953 | D02204 |
| 2 | hsa_22953 | D02360 |
| 2 | hsa_22953 | D02485 |
| 2 | hsa_23630 | D00477 |
| 2 | hsa_23630 | D00528 |
| 2 | hsa_23630 | D00638 |
| 2 | hsa_23630 | D00735 |
| 2 | hsa_23630 | D00779 |
| 2 | hsa_23630 | D00823 |
| 2 | hsa_23630 | D02173 |
| 2 | hsa_23630 | D02234 |
| 2 | hsa_23630 | D02485 |
| 2 | hsa_23630 | D05024 |
| 2 | hsa_23704 | D00225 |
| 2 | hsa_23704 | D00252 |
| 2 | hsa_23704 | D00303 |
| 2 | hsa_23704 | D00338 |
| 2 | hsa_23704 | D00480 |
| 2 | hsa_23704 | D00538 |
| 2 | hsa_23704 | D00560 |
| 2 | hsa_23704 | D00656 |
| 2 | hsa_23704 | D00735 |
| 2 | hsa_23704 | D01118 |
| 2 | hsa_23704 | D02485 |
| 2 | hsa_23704 | D03180 |
| 2 | hsa_2554 | D00252 |
| 2 | hsa_2554 | D00349 |
| 2 | hsa_2554 | D00354 |
| 2 | hsa_2554 | D00392 |
| 2 | hsa_2554 | D00495 |
| 2 | hsa_2554 | D00524 |
| 2 | hsa_2554 | D00633 |
| 2 | hsa_2554 | D00639 |
| 2 | hsa_2554 | D00733 |
| 2 | hsa_2554 | D00735 |
| 2 | hsa_2554 | D00816 |
| 2 | hsa_2554 | D01108 |
| 2 | hsa_2554 | D01111 |
| 2 | hsa_2554 | D02041 |
| 2 | hsa_2554 | D02101 |
| 2 | hsa_2554 | D02360 |
| 2 | hsa_2554 | D03450 |
| 2 | hsa_2555 | D00338 |
| 2 | hsa_2555 | D00379 |
| 2 | hsa_2555 | D00504 |
| 2 | hsa_2555 | D00512 |
| 2 | hsa_2555 | D00771 |
| 2 | hsa_2555 | D00809 |
| 2 | hsa_2555 | D00960 |
| 2 | hsa_2555 | D03878 |
| 2 | hsa_2555 | D05077 |
| 2 | hsa_2555 | D05458 |
| 2 | hsa_2556 | D00110 |
| 2 | hsa_2556 | D00227 |
| 2 | hsa_2556 | D00294 |
| 2 | hsa_2556 | D00303 |
| 2 | hsa_2556 | D00340 |
| 2 | hsa_2556 | D00379 |
| 2 | hsa_2556 | D00474 |
| 2 | hsa_2556 | D00524 |
| 2 | hsa_2556 | D00779 |
| 2 | hsa_2556 | D00799 |
| 2 | hsa_2556 | D00823 |
| 2 | hsa_2556 | D01253 |
| 2 | hsa_2556 | D01712 |
| 2 | hsa_2556 | D01969 |
| 2 | hsa_2556 | D02088 |
| 2 | hsa_2556 | D02566 |
| 2 | hsa_2556 | D05453 |
| 2 | hsa_2557 | D00283 |
| 2 | hsa_2557 | D00464 |
| 2 | hsa_2557 | D00504 |
| 2 | hsa_2557 | D00546 |
| 2 | hsa_2557 | D00553 |
| 2 | hsa_2557 | D00560 |
| 2 | hsa_2557 | D00631 |
| 2 | hsa_2557 | D00678 |
| 2 | hsa_2557 | D00697 |
| 2 | hsa_2557 | D06106 |
| 2 | hsa_2558 | D00136 |
| 2 | hsa_2558 | D00335 |
| 2 | hsa_2558 | D00394 |
| 2 | hsa_2558 | D00480 |
| 2 | hsa_2558 | D00618 |
| 2 | hsa_2558 | D00633 |
| 2 | hsa_2558 | D00638 |
| 2 | hsa_2558 | D00639 |
| 2 | hsa_2558 | D00738 |
| 2 | hsa_2558 | D00809 |
| 2 | hsa_2558 | D01108 |
| 2 | hsa_2558 | D02086 |
| 2 | hsa_2558 | D02237 |
| 2 | hsa_2559 | D00340 |
| 2 | hsa_2559 | D00418 |
| 2 | hsa_2559 | D00464 |
| 2 | hsa_2559 | D00474 |
| 2 | hsa_2559 | D00495 |
| 2 | hsa_2559 | D00553 |
| 2 | hsa_2559 | D00636 |
| 2 | hsa_2559 | D00760 |
| 2 | hsa_2559 | D02163 |
| 2 | hsa_2559 | D05024 |
| 2 | hsa_2560 | D00274 |
| 2 | hsa_2560 | D00451 |
| 2 | hsa_2560 | D00464 |
| 2 | hsa_2560 | D00474 |
| 2 | hsa_2560 | D00477 |
| 2 | hsa_2560 | D00546 |
| 2 | hsa_2560 | D00616 |
| 2 | hsa_2560 | D00617 |
| 2 | hsa_2560 | D00619 |
| 2 | hsa_2560 | D00709 |
| 2 | hsa_2560 | D00732 |
| 2 | hsa_2560 | D00735 |
| 2 | hsa_2560 | D00741 |
| 2 | hsa_2560 | D00775 |
| 2 | hsa_2560 | D00960 |
| 2 | hsa_2560 | D01295 |
| 2 | hsa_2560 | D01768 |
| 2 | hsa_2560 | D01828 |
| 2 | hsa_2560 | D02086 |
| 2 | hsa_2560 | D02173 |
| 2 | hsa_2560 | D02237 |
| 2 | hsa_2560 | D02461 |
| 2 | hsa_2560 | D05453 |
| 2 | hsa_2561 | D00294 |
| 2 | hsa_2561 | D00303 |
| 2 | hsa_2561 | D00335 |
| 2 | hsa_2561 | D00338 |
| 2 | hsa_2561 | D00394 |
| 2 | hsa_2561 | D00474 |
| 2 | hsa_2561 | D00563 |
| 2 | hsa_2561 | D00594 |
| 2 | hsa_2561 | D00619 |
| 2 | hsa_2561 | D02272 |
| 2 | hsa_2562 | D00495 |
| 2 | hsa_2562 | D00542 |
| 2 | hsa_2562 | D00544 |
| 2 | hsa_2562 | D00563 |
| 2 | hsa_2562 | D00615 |
| 2 | hsa_2562 | D00704 |
| 2 | hsa_2562 | D02041 |
| 2 | hsa_2562 | D03037 |
| 2 | hsa_2562 | D05024 |
| 2 | hsa_2563 | D00110 |
| 2 | hsa_2563 | D00380 |
| 2 | hsa_2563 | D00474 |
| 2 | hsa_2563 | D00524 |
| 2 | hsa_2563 | D00538 |
| 2 | hsa_2563 | D00633 |
| 2 | hsa_2563 | D00678 |
| 2 | hsa_2563 | D00697 |
| 2 | hsa_2563 | D00740 |
| 2 | hsa_2563 | D00791 |
| 2 | hsa_2563 | D00960 |
| 2 | hsa_2563 | D01448 |
| 2 | hsa_2563 | D02086 |
| 2 | hsa_2563 | D02087 |
| 2 | hsa_2563 | D04985 |
| 2 | hsa_2564 | D00519 |
| 2 | hsa_2564 | D00533 |
| 2 | hsa_2564 | D00544 |
| 2 | hsa_2564 | D00648 |
| 2 | hsa_2564 | D00649 |
| 2 | hsa_2564 | D00740 |
| 2 | hsa_2564 | D00798 |
| 2 | hsa_2564 | D00964 |
| 2 | hsa_2564 | D01243 |
| 2 | hsa_2564 | D02087 |
| 2 | hsa_2564 | D02098 |
| 2 | hsa_2564 | D02262 |
| 2 | hsa_2564 | D02485 |
| 2 | hsa_2564 | D02630 |
| 2 | hsa_2564 | D04034 |
| 2 | hsa_2564 | D04999 |
| 2 | hsa_2566 | D00283 |
| 2 | hsa_2566 | D00294 |
| 2 | hsa_2566 | D00394 |
| 2 | hsa_2566 | D00495 |
| 2 | hsa_2566 | D00521 |
| 2 | hsa_2566 | D00539 |
| 2 | hsa_2566 | D00553 |
| 2 | hsa_2566 | D00735 |
| 2 | hsa_2566 | D01111 |
| 2 | hsa_2566 | D03830 |
| 2 | hsa_2566 | D04034 |
| 2 | hsa_2567 | D00345 |
| 2 | hsa_2567 | D00349 |
| 2 | hsa_2567 | D00358 |
| 2 | hsa_2567 | D00513 |
| 2 | hsa_2567 | D00563 |
| 2 | hsa_2567 | D00607 |
| 2 | hsa_2567 | D00633 |
| 2 | hsa_2567 | D00708 |
| 2 | hsa_2567 | D00759 |
| 2 | hsa_2567 | D01242 |
| 2 | hsa_2567 | D01828 |
| 2 | hsa_2567 | D01969 |
| 2 | hsa_2567 | D02087 |
| 2 | hsa_2567 | D02088 |
| 2 | hsa_2567 | D04034 |
| 2 | hsa_2567 | D04999 |
| 2 | hsa_2567 | D05461 |
| 2 | hsa_2569 | D00219 |
| 2 | hsa_2569 | D00394 |
| 2 | hsa_2569 | D00477 |
| 2 | hsa_2569 | D00513 |
| 2 | hsa_2569 | D00615 |
| 2 | hsa_2569 | D00648 |
| 2 | hsa_2569 | D00791 |
| 2 | hsa_2569 | D00816 |
| 2 | hsa_2569 | D02237 |
| 2 | hsa_2569 | D03830 |
| 2 | hsa_2570 | D00219 |
| 2 | hsa_2570 | D00227 |
| 2 | hsa_2570 | D00234 |
| 2 | hsa_2570 | D00335 |
| 2 | hsa_2570 | D00380 |
| 2 | hsa_2570 | D00438 |
| 2 | hsa_2570 | D00545 |
| 2 | hsa_2570 | D00650 |
| 2 | hsa_2570 | D00654 |
| 2 | hsa_2570 | D00694 |
| 2 | hsa_2570 | D00704 |
| 2 | hsa_2570 | D00760 |
| 2 | hsa_2570 | D01118 |
| 2 | hsa_2570 | D01287 |
| 2 | hsa_2570 | D02101 |
| 2 | hsa_2570 | D02204 |
| 2 | hsa_2570 | D03450 |
| 2 | hsa_26251 | D00225 |
| 2 | hsa_26251 | D00326 |
| 2 | hsa_26251 | D00392 |
| 2 | hsa_26251 | D00474 |
| 2 | hsa_26251 | D00678 |
| 2 | hsa_26251 | D00726 |
| 2 | hsa_26251 | D00740 |
| 2 | hsa_26251 | D01242 |
| 2 | hsa_26251 | D02088 |
| 2 | hsa_26251 | D02101 |
| 2 | hsa_26251 | D02237 |
| 2 | hsa_26251 | D02262 |
| 2 | hsa_26251 | D02461 |
| 2 | hsa_26251 | D02485 |
| 2 | hsa_26251 | D05461 |
| 2 | hsa_27012 | D00227 |
| 2 | hsa_27012 | D00326 |
| 2 | hsa_27012 | D00542 |
| 2 | hsa_27012 | D00617 |
| 2 | hsa_27012 | D00726 |
| 2 | hsa_27012 | D00740 |
| 2 | hsa_27012 | D00761 |
| 2 | hsa_27012 | D01242 |
| 2 | hsa_27012 | D01256 |
| 2 | hsa_27012 | D02092 |
| 2 | hsa_27012 | D02207 |
| 2 | hsa_27012 | D02566 |
| 2 | hsa_27012 | D03826 |
| 2 | hsa_27092 | D00110 |
| 2 | hsa_27092 | D00319 |
| 2 | hsa_27092 | D00544 |
| 2 | hsa_27092 | D00549 |
| 2 | hsa_27092 | D00704 |
| 2 | hsa_27092 | D00738 |
| 2 | hsa_27092 | D00771 |
| 2 | hsa_27092 | D00960 |
| 2 | hsa_27092 | D01111 |
| 2 | hsa_27092 | D02234 |
| 2 | hsa_27092 | D03826 |
| 2 | hsa_27092 | D04034 |
| 2 | hsa_27094 | D00392 |
| 2 | hsa_27094 | D00477 |
| 2 | hsa_27094 | D00539 |
| 2 | hsa_27094 | D00547 |
| 2 | hsa_27094 | D00650 |
| 2 | hsa_27094 | D00658 |
| 2 | hsa_27094 | D00765 |
| 2 | hsa_27094 | D00809 |
| 2 | hsa_27094 | D01575 |
| 2 | hsa_27094 | D01712 |
| 2 | hsa_27094 | D02362 |
| 2 | hsa_27094 | D03742 |
| 2 | hsa_27133 | D00136 |
| 2 | hsa_27133 | D00228 |
| 2 | hsa_27133 | D00252 |
| 2 | hsa_27133 | D00437 |
| 2 | hsa_27133 | D00456 |
| 2 | hsa_27133 | D00480 |
| 2 | hsa_27133 | D00607 |
| 2 | hsa_27133 | D00732 |
| 2 | hsa_27133 | D00759 |
| 2 | hsa_27133 | D01448 |
| 2 | hsa_27133 | D01799 |
| 2 | hsa_27133 | D02262 |
| 2 | hsa_27133 | D02461 |
| 2 | hsa_27133 | D03180 |
| 2 | hsa_27133 | D03991 |
| 2 | hsa_27345 | D00303 |
| 2 | hsa_27345 | D00340 |
| 2 | hsa_27345 | D00379 |
| 2 | hsa_27345 | D00418 |
| 2 | hsa_27345 | D00437 |
| 2 | hsa_27345 | D00524 |
| 2 | hsa_27345 | D00546 |
| 2 | hsa_27345 | D00548 |
| 2 | hsa_27345 | D00593 |
| 2 | hsa_27345 | D00648 |
| 2 | hsa_27345 | D00658 |
| 2 | hsa_27345 | D00697 |
| 2 | hsa_27345 | D00733 |
| 2 | hsa_27345 | D00741 |
| 2 | hsa_27345 | D00771 |
| 2 | hsa_27345 | D01828 |
| 2 | hsa_27345 | D03991 |
| 2 | hsa_2741 | D00332 |
| 2 | hsa_2741 | D00358 |
| 2 | hsa_2741 | D00364 |
| 2 | hsa_2741 | D00477 |
| 2 | hsa_2741 | D00504 |
| 2 | hsa_2741 | D00574 |
| 2 | hsa_2741 | D00616 |
| 2 | hsa_2741 | D00648 |
| 2 | hsa_2741 | D00678 |
| 2 | hsa_2741 | D00740 |
| 2 | hsa_2741 | D00761 |
| 2 | hsa_2741 | D01242 |
| 2 | hsa_2741 | D01243 |
| 2 | hsa_2741 | D02546 |
| 2 | hsa_2741 | D02630 |
| 2 | hsa_2741 | D03180 |
| 2 | hsa_2741 | D05156 |
| 2 | hsa_2742 | D00464 |
| 2 | hsa_2742 | D00563 |
| 2 | hsa_2742 | D00618 |
| 2 | hsa_2742 | D00640 |
| 2 | hsa_2742 | D00654 |
| 2 | hsa_2742 | D00708 |
| 2 | hsa_2742 | D00739 |
| 2 | hsa_2742 | D00740 |
| 2 | hsa_2742 | D00812 |
| 2 | hsa_2742 | D01253 |
| 2 | hsa_2742 | D01256 |
| 2 | hsa_2742 | D01448 |
| 2 | hsa_2742 | D01450 |
| 2 | hsa_2742 | D01969 |
| 2 | hsa_2742 | D02101 |
| 2 | hsa_2742 | D02566 |
| 2 | hsa_2742 | D03826 |
| 2 | hsa_2742 | D05077 |
| 2 | hsa_2743 | D00219 |
| 2 | hsa_2743 | D00225 |
| 2 | hsa_2743 | D00283 |
| 2 | hsa_2743 | D00326 |
| 2 | hsa_2743 | D00345 |
| 2 | hsa_2743 | D00351 |
| 2 | hsa_2743 | D00364 |
| 2 | hsa_2743 | D00499 |
| 2 | hsa_2743 | D00521 |
| 2 | hsa_2743 | D00524 |
| 2 | hsa_2743 | D00553 |
| 2 | hsa_2743 | D00618 |
| 2 | hsa_2743 | D00650 |
| 2 | hsa_2743 | D00960 |
| 2 | hsa_2743 | D00964 |
| 2 | hsa_2743 | D01287 |
| 2 | hsa_2743 | D01599 |
| 2 | hsa_2743 | D01768 |
| 2 | hsa_2743 | D02362 |
| 2 | hsa_2743 | D03037 |
| 2 | hsa_285242 | D00338 |
| 2 | hsa_285242 | D00524 |
| 2 | hsa_285242 | D00533 |
| 2 | hsa_285242 | D00552 |
| 2 | hsa_285242 | D00560 |
| 2 | hsa_285242 | D00638 |
| 2 | hsa_285242 | D00648 |
| 2 | hsa_285242 | D00740 |
| 2 | hsa_285242 | D00760 |
| 2 | hsa_285242 | D00798 |
| 2 | hsa_285242 | D00963 |
| 2 | hsa_285242 | D01854 |
| 2 | hsa_285242 | D05156 |
| 2 | hsa_2890 | D00234 |
| 2 | hsa_2890 | D00340 |
| 2 | hsa_2890 | D00394 |
| 2 | hsa_2890 | D00456 |
| 2 | hsa_2890 | D00521 |
| 2 | hsa_2890 | D00537 |
| 2 | hsa_2890 | D00618 |
| 2 | hsa_2890 | D00678 |
| 2 | hsa_2890 | D00694 |
| 2 | hsa_2890 | D00733 |
| 2 | hsa_2890 | D00740 |
| 2 | hsa_2890 | D00761 |
| 2 | hsa_2890 | D02092 |
| 2 | hsa_2890 | D02101 |
| 2 | hsa_2890 | D02261 |
| 2 | hsa_2891 | D00345 |
| 2 | hsa_2891 | D00364 |
| 2 | hsa_2891 | D00477 |
| 2 | hsa_2891 | D00545 |
| 2 | hsa_2891 | D00552 |
| 2 | hsa_2891 | D00631 |
| 2 | hsa_2891 | D00638 |
| 2 | hsa_2891 | D00726 |
| 2 | hsa_2891 | D01118 |
| 2 | hsa_2891 | D01243 |
| 2 | hsa_2891 | D01448 |
| 2 | hsa_2891 | D02234 |
| 2 | hsa_2891 | D02253 |
| 2 | hsa_2891 | D02356 |
| 2 | hsa_2891 | D02362 |
| 2 | hsa_2891 | D04999 |
| 2 | hsa_2892 | D00136 |
| 2 | hsa_2892 | D00329 |
| 2 | hsa_2892 | D00379 |
| 2 | hsa_2892 | D00528 |
| 2 | hsa_2892 | D00530 |
| 2 | hsa_2892 | D00536 |
| 2 | hsa_2892 | D00543 |
| 2 | hsa_2892 | D00617 |
| 2 | hsa_2892 | D00640 |
| 2 | hsa_2892 | D00647 |
| 2 | hsa_2892 | D00649 |
| 2 | hsa_2892 | D00704 |
| 2 | hsa_2892 | D00760 |
| 2 | hsa_2892 | D02261 |
| 2 | hsa_2892 | D02914 |
| 2 | hsa_2892 | D03830 |
| 2 | hsa_2892 | D06106 |
| 2 | hsa_2893 | D00136 |
| 2 | hsa_2893 | D00335 |
| 2 | hsa_2893 | D00367 |
| 2 | hsa_2893 | D00616 |
| 2 | hsa_2893 | D00761 |
| 2 | hsa_2893 | D00779 |
| 2 | hsa_2893 | D02204 |
| 2 | hsa_2893 | D02207 |
| 2 | hsa_2893 | D02262 |
| 2 | hsa_2893 | D02362 |
| 2 | hsa_2893 | D04790 |
| 2 | hsa_2893 | D06172 |
| 2 | hsa_2895 | D00338 |
| 2 | hsa_2895 | D00367 |
| 2 | hsa_2895 | D00437 |
| 2 | hsa_2895 | D00544 |
| 2 | hsa_2895 | D00553 |
| 2 | hsa_2895 | D00560 |
| 2 | hsa_2895 | D00648 |
| 2 | hsa_2895 | D00649 |
| 2 | hsa_2895 | D00823 |
| 2 | hsa_2895 | D04790 |
| 2 | hsa_2897 | D00329 |
| 2 | hsa_2897 | D00464 |
| 2 | hsa_2897 | D00477 |
| 2 | hsa_2897 | D00642 |
| 2 | hsa_2897 | D00650 |
| 2 | hsa_2897 | D00704 |
| 2 | hsa_2897 | D00759 |
| 2 | hsa_2897 | D01111 |
| 2 | hsa_2897 | D02087 |
| 2 | hsa_2897 | D02237 |
| 2 | hsa_2897 | D03274 |
| 2 | hsa_2897 | D05453 |
| 2 | hsa_2898 | D00195 |
| 2 | hsa_2898 | D00225 |
| 2 | hsa_2898 | D00351 |
| 2 | hsa_2898 | D00495 |
| 2 | hsa_2898 | D00519 |
| 2 | hsa_2898 | D00538 |
| 2 | hsa_2898 | D00633 |
| 2 | hsa_2898 | D00656 |
| 2 | hsa_2898 | D00735 |
| 2 | hsa_2898 | D00759 |
| 2 | hsa_2898 | D00960 |
| 2 | hsa_2898 | D01111 |
| 2 | hsa_2898 | D01243 |
| 2 | hsa_2898 | D01712 |
| 2 | hsa_2899 | D00274 |
| 2 | hsa_2899 | D00345 |
| 2 | hsa_2899 | D00358 |
| 2 | hsa_2899 | D00367 |
| 2 | hsa_2899 | D00480 |
| 2 | hsa_2899 | D00611 |
| 2 | hsa_2899 | D00631 |
| 2 | hsa_2899 | D00649 |
| 2 | hsa_2899 | D00656 |
| 2 | hsa_2899 | D00677 |
| 2 | hsa_2899 | D00739 |
| 2 | hsa_2899 | D00741 |
| 2 | hsa_2899 | D00964 |
| 2 | hsa_2899 | D01108 |
| 2 | hsa_2899 | D01799 |
| 2 | hsa_2899 | D02234 |
| 2 | hsa_2899 | D02485 |
| 2 | hsa_2899 | D02566 |
| 2 | hsa_2899 | D02914 |
| 2 | hsa_2899 | D03826 |
| 2 | hsa_2899 | D04999 |
| 2 | hsa_2900 | D00326 |
| 2 | hsa_2900 | D00329 |
| 2 | hsa_2900 | D00358 |
| 2 | hsa_2900 | D00495 |
| 2 | hsa_2900 | D00513 |
| 2 | hsa_2900 | D00537 |
| 2 | hsa_2900 | D00538 |
| 2 | hsa_2900 | D00539 |
| 2 | hsa_2900 | D00550 |
| 2 | hsa_2900 | D00636 |
| 2 | hsa_2900 | D00760 |
| 2 | hsa_2900 | D00831 |
| 2 | hsa_2900 | D02272 |
| 2 | hsa_2900 | D05077 |
| 2 | hsa_2901 | D00528 |
| 2 | hsa_2901 | D00533 |
| 2 | hsa_2901 | D00537 |
| 2 | hsa_2901 | D00547 |
| 2 | hsa_2901 | D00615 |
| 2 | hsa_2901 | D00631 |
| 2 | hsa_2901 | D00639 |
| 2 | hsa_2901 | D00704 |
| 2 | hsa_2901 | D00726 |
| 2 | hsa_2901 | D00765 |
| 2 | hsa_2901 | D00816 |
| 2 | hsa_2901 | D01603 |
| 2 | hsa_2901 | D01828 |
| 2 | hsa_2901 | D02362 |
| 2 | hsa_2901 | D03991 |
| 2 | hsa_2902 | D00438 |
| 2 | hsa_2902 | D00537 |
| 2 | hsa_2902 | D00545 |
| 2 | hsa_2902 | D00548 |
| 2 | hsa_2902 | D00563 |
| 2 | hsa_2902 | D00594 |
| 2 | hsa_2902 | D00618 |
| 2 | hsa_2902 | D00619 |
| 2 | hsa_2902 | D00735 |
| 2 | hsa_2902 | D00775 |
| 2 | hsa_2902 | D01253 |
| 2 | hsa_2902 | D01287 |
| 2 | hsa_2902 | D01599 |
| 2 | hsa_2902 | D02086 |
| 2 | hsa_2902 | D02207 |
| 2 | hsa_2902 | D02253 |
| 2 | hsa_2902 | D03450 |
| 2 | hsa_2902 | D04790 |
| 2 | hsa_2903 | D00293 |
| 2 | hsa_2903 | D00326 |
| 2 | hsa_2903 | D00332 |
| 2 | hsa_2903 | D00418 |
| 2 | hsa_2903 | D00593 |
| 2 | hsa_2903 | D00656 |
| 2 | hsa_2903 | D00678 |
| 2 | hsa_2903 | D00823 |
| 2 | hsa_2903 | D00963 |
| 2 | hsa_2903 | D01253 |
| 2 | hsa_2903 | D01712 |
| 2 | hsa_2903 | D01768 |
| 2 | hsa_2903 | D02237 |
| 2 | hsa_2903 | D02272 |
| 2 | hsa_2904 | D00303 |
| 2 | hsa_2904 | D00319 |
| 2 | hsa_2904 | D00354 |
| 2 | hsa_2904 | D00364 |
| 2 | hsa_2904 | D00537 |
| 2 | hsa_2904 | D00549 |
| 2 | hsa_2904 | D00550 |
| 2 | hsa_2904 | D00798 |
| 2 | hsa_2904 | D00809 |
| 2 | hsa_2904 | D00812 |
| 2 | hsa_2904 | D01118 |
| 2 | hsa_2904 | D01243 |
| 2 | hsa_2904 | D02207 |
| 2 | hsa_2904 | D04790 |
| 2 | hsa_2904 | D05461 |
| 2 | hsa_2905 | D00283 |
| 2 | hsa_2905 | D00319 |
| 2 | hsa_2905 | D00354 |
| 2 | hsa_2905 | D00364 |
| 2 | hsa_2905 | D00373 |
| 2 | hsa_2905 | D00593 |
| 2 | hsa_2905 | D00615 |
| 2 | hsa_2905 | D00636 |
| 2 | hsa_2905 | D00704 |
| 2 | hsa_2905 | D00733 |
| 2 | hsa_2905 | D00735 |
| 2 | hsa_2905 | D00964 |
| 2 | hsa_2905 | D01828 |
| 2 | hsa_2905 | D02163 |
| 2 | hsa_2905 | D02262 |
| 2 | hsa_2905 | D03742 |
| 2 | hsa_2906 | D00329 |
| 2 | hsa_2906 | D00336 |
| 2 | hsa_2906 | D00456 |
| 2 | hsa_2906 | D00548 |
| 2 | hsa_2906 | D00553 |
| 2 | hsa_2906 | D01599 |
| 2 | hsa_2906 | D01854 |
| 2 | hsa_2906 | D02173 |
| 2 | hsa_2906 | D02409 |
| 2 | hsa_2906 | D03830 |
| 2 | hsa_3359 | D00228 |
| 2 | hsa_3359 | D00274 |
| 2 | hsa_3359 | D00512 |
| 2 | hsa_3359 | D00530 |
| 2 | hsa_3359 | D00536 |
| 2 | hsa_3359 | D00694 |
| 2 | hsa_3359 | D01448 |
| 2 | hsa_3359 | D01453 |
| 2 | hsa_3359 | D01599 |
| 2 | hsa_3359 | D02086 |
| 2 | hsa_3359 | D02237 |
| 2 | hsa_3359 | D02362 |
| 2 | hsa_3359 | D03878 |
| 2 | hsa_3359 | D04985 |
| 2 | hsa_3736 | D00195 |
| 2 | hsa_3736 | D00274 |
| 2 | hsa_3736 | D00293 |
| 2 | hsa_3736 | D00504 |
| 2 | hsa_3736 | D00539 |
| 2 | hsa_3736 | D00548 |
| 2 | hsa_3736 | D00615 |
| 2 | hsa_3736 | D00618 |
| 2 | hsa_3736 | D00633 |
| 2 | hsa_3736 | D00648 |
| 2 | hsa_3736 | D00678 |
| 2 | hsa_3736 | D01243 |
| 2 | hsa_3736 | D01253 |
| 2 | hsa_3736 | D01453 |
| 2 | hsa_3736 | D02207 |
| 2 | hsa_3736 | D02362 |
| 2 | hsa_3736 | D02566 |
| 2 | hsa_3736 | D03037 |
| 2 | hsa_3737 | D00303 |
| 2 | hsa_3737 | D00351 |
| 2 | hsa_3737 | D00358 |
| 2 | hsa_3737 | D00379 |
| 2 | hsa_3737 | D00437 |
| 2 | hsa_3737 | D00538 |
| 2 | hsa_3737 | D00639 |
| 2 | hsa_3737 | D00654 |
| 2 | hsa_3737 | D00694 |
| 2 | hsa_3737 | D00735 |
| 2 | hsa_3737 | D01108 |
| 2 | hsa_3737 | D02485 |
| 2 | hsa_3737 | D03180 |
| 2 | hsa_3737 | D05024 |
| 2 | hsa_3738 | D00136 |
| 2 | hsa_3738 | D00219 |
| 2 | hsa_3738 | D00228 |
| 2 | hsa_3738 | D00418 |
| 2 | hsa_3738 | D00524 |
| 2 | hsa_3738 | D00544 |
| 2 | hsa_3738 | D00552 |
| 2 | hsa_3738 | D00617 |
| 2 | hsa_3738 | D01253 |
| 2 | hsa_3738 | D02041 |
| 2 | hsa_3739 | D00345 |
| 2 | hsa_3739 | D00418 |
| 2 | hsa_3739 | D00437 |
| 2 | hsa_3739 | D00519 |
| 2 | hsa_3739 | D00726 |
| 2 | hsa_3739 | D00779 |
| 2 | hsa_3739 | D00812 |
| 2 | hsa_3739 | D02914 |
| 2 | hsa_3739 | D03450 |
| 2 | hsa_3741 | D00293 |
| 2 | hsa_3741 | D00367 |
| 2 | hsa_3741 | D00418 |
| 2 | hsa_3741 | D00553 |
| 2 | hsa_3741 | D00611 |
| 2 | hsa_3741 | D00647 |
| 2 | hsa_3741 | D00650 |
| 2 | hsa_3741 | D01287 |
| 2 | hsa_3741 | D01603 |
| 2 | hsa_3741 | D02041 |
| 2 | hsa_3741 | D02362 |
| 2 | hsa_3741 | D02566 |
| 2 | hsa_3742 | D00195 |
| 2 | hsa_3742 | D00331 |
| 2 | hsa_3742 | D00480 |
| 2 | hsa_3742 | D00499 |
| 2 | hsa_3742 | D00548 |
| 2 | hsa_3742 | D00654 |
| 2 | hsa_3742 | D00733 |
| 2 | hsa_3742 | D00960 |
| 2 | hsa_3742 | D01575 |
| 2 | hsa_3742 | D02204 |
| 2 | hsa_3742 | D02237 |
| 2 | hsa_3742 | D02362 |
| 2 | hsa_3742 | D05077 |
| 2 | hsa_3742 | D05453 |
| 2 | hsa_3743 | D00252 |
| 2 | hsa_3743 | D00456 |
| 2 | hsa_3743 | D00521 |
| 2 | hsa_3743 | D00560 |
| 2 | hsa_3743 | D00593 |
| 2 | hsa_3743 | D00615 |
| 2 | hsa_3743 | D00617 |
| 2 | hsa_3743 | D00639 |
| 2 | hsa_3743 | D00694 |
| 2 | hsa_3743 | D01242 |
| 2 | hsa_3743 | D01603 |
| 2 | hsa_3743 | D02087 |
| 2 | hsa_3743 | D02092 |
| 2 | hsa_3743 | D02546 |
| 2 | hsa_3743 | D03037 |
| 2 | hsa_3743 | D03274 |
| 2 | hsa_3743 | D04985 |
| 2 | hsa_3743 | D05461 |
| 2 | hsa_3744 | D00349 |
| 2 | hsa_3744 | D00437 |
| 2 | hsa_3744 | D00477 |
| 2 | hsa_3744 | D00536 |
| 2 | hsa_3744 | D00538 |
| 2 | hsa_3744 | D00544 |
| 2 | hsa_3744 | D00545 |
| 2 | hsa_3744 | D00546 |
| 2 | hsa_3744 | D00553 |
| 2 | hsa_3744 | D00563 |
| 2 | hsa_3744 | D00615 |
| 2 | hsa_3744 | D02086 |
| 2 | hsa_3744 | D02207 |
| 2 | hsa_3744 | D02485 |
| 2 | hsa_3744 | D03180 |
| 2 | hsa_3744 | D03830 |
| 2 | hsa_3744 | D05453 |
| 2 | hsa_3745 | D00234 |
| 2 | hsa_3745 | D00392 |
| 2 | hsa_3745 | D00456 |
| 2 | hsa_3745 | D00528 |
| 2 | hsa_3745 | D00536 |
| 2 | hsa_3745 | D00607 |
| 2 | hsa_3745 | D00638 |
| 2 | hsa_3745 | D00711 |
| 2 | hsa_3745 | D00732 |
| 2 | hsa_3745 | D00741 |
| 2 | hsa_3745 | D02546 |
| 2 | hsa_3745 | D06106 |
| 2 | hsa_3746 | D00340 |
| 2 | hsa_3746 | D00394 |
| 2 | hsa_3746 | D00499 |
| 2 | hsa_3746 | D00504 |
| 2 | hsa_3746 | D00607 |
| 2 | hsa_3746 | D01768 |
| 2 | hsa_3746 | D01799 |
| 2 | hsa_3746 | D03450 |
| 2 | hsa_3746 | D03742 |
| 2 | hsa_3746 | D05453 |
| 2 | hsa_3747 | D00225 |
| 2 | hsa_3747 | D00228 |
| 2 | hsa_3747 | D00283 |
| 2 | hsa_3747 | D00349 |
| 2 | hsa_3747 | D00351 |
| 2 | hsa_3747 | D00358 |
| 2 | hsa_3747 | D00550 |
| 2 | hsa_3747 | D00616 |
| 2 | hsa_3747 | D00636 |
| 2 | hsa_3747 | D00739 |
| 2 | hsa_3747 | D01118 |
| 2 | hsa_3747 | D01603 |
| 2 | hsa_3747 | D02087 |
| 2 | hsa_3747 | D02088 |
| 2 | hsa_3747 | D02163 |
| 2 | hsa_3747 | D02630 |
| 2 | hsa_3747 | D05024 |
| 2 | hsa_3748 | D00219 |
| 2 | hsa_3748 | D00274 |
| 2 | hsa_3748 | D00358 |
| 2 | hsa_3748 | D00477 |
| 2 | hsa_3748 | D00545 |
| 2 | hsa_3748 | D00799 |
| 2 | hsa_3748 | D01071 |
| 2 | hsa_3748 | D01118 |
| 2 | hsa_3748 | D02234 |
| 2 | hsa_3748 | D02409 |
| 2 | hsa_3749 | D00225 |
| 2 | hsa_3749 | D00351 |
| 2 | hsa_3749 | D00437 |
| 2 | hsa_3749 | D00438 |
| 2 | hsa_3749 | D00474 |
| 2 | hsa_3749 | D00519 |
| 2 | hsa_3749 | D00528 |
| 2 | hsa_3749 | D00553 |
| 2 | hsa_3749 | D00615 |
| 2 | hsa_3749 | D00694 |
| 2 | hsa_3749 | D00704 |
| 2 | hsa_3749 | D00816 |
| 2 | hsa_3749 | D01768 |
| 2 | hsa_3749 | D02092 |
| 2 | hsa_3749 | D02234 |
| 2 | hsa_3749 | D02566 |
| 2 | hsa_3749 | D04790 |
| 2 | hsa_3749 | D05024 |
| 2 | hsa_3749 | D05458 |
| 2 | hsa_3749 | D06172 |
| 2 | hsa_3750 | D00456 |
| 2 | hsa_3750 | D00618 |
| 2 | hsa_3750 | D00740 |
| 2 | hsa_3750 | D01448 |
| 2 | hsa_3750 | D02253 |
| 2 | hsa_3750 | D02630 |
| 2 | hsa_3750 | D05453 |
| 2 | hsa_3751 | D00195 |
| 2 | hsa_3751 | D00332 |
| 2 | hsa_3751 | D00364 |
| 2 | hsa_3751 | D00392 |
| 2 | hsa_3751 | D00607 |
| 2 | hsa_3751 | D00636 |
| 2 | hsa_3751 | D00656 |
| 2 | hsa_3751 | D00697 |
| 2 | hsa_3751 | D00765 |
| 2 | hsa_3751 | D00960 |
| 2 | hsa_3751 | D01448 |
| 2 | hsa_3751 | D02566 |
| 2 | hsa_3752 | D00438 |
| 2 | hsa_3752 | D00544 |
| 2 | hsa_3752 | D00650 |
| 2 | hsa_3752 | D01253 |
| 2 | hsa_3752 | D01768 |
| 2 | hsa_3752 | D02360 |
| 2 | hsa_3752 | D05461 |
| 2 | hsa_3753 | D00274 |
| 2 | hsa_3753 | D00283 |
| 2 | hsa_3753 | D00340 |
| 2 | hsa_3753 | D00464 |
| 2 | hsa_3753 | D00542 |
| 2 | hsa_3753 | D00547 |
| 2 | hsa_3753 | D00631 |
| 2 | hsa_3753 | D00649 |
| 2 | hsa_3753 | D00775 |
| 2 | hsa_3753 | D00809 |
| 2 | hsa_3753 | D02101 |
| 2 | hsa_3754 | D00219 |
| 2 | hsa_3754 | D00283 |
| 2 | hsa_3754 | D00303 |
| 2 | hsa_3754 | D00329 |
| 2 | hsa_3754 | D00474 |
| 2 | hsa_3754 | D00519 |
| 2 | hsa_3754 | D00521 |
| 2 | hsa_3754 | D00550 |
| 2 | hsa_3754 | D00563 |
| 2 | hsa_3754 | D00574 |
| 2 | hsa_3754 | D00647 |
| 2 | hsa_3754 | D00708 |
| 2 | hsa_3754 | D00733 |
| 2 | hsa_3754 | D00798 |
| 2 | hsa_3754 | D00963 |
| 2 | hsa_3754 | D01071 |
| 2 | hsa_3754 | D01242 |
| 2 | hsa_3754 | D01712 |
| 2 | hsa_3754 | D01854 |
| 2 | hsa_3754 | D02362 |
| 2 | hsa_3754 | D02461 |
| 2 | hsa_3755 | D00293 |
| 2 | hsa_3755 | D00524 |
| 2 | hsa_3755 | D00553 |
| 2 | hsa_3755 | D00629 |
| 2 | hsa_3755 | D00640 |
| 2 | hsa_3755 | D00656 |
| 2 | hsa_3755 | D00733 |
| 2 | hsa_3755 | D00759 |
| 2 | hsa_3755 | D03742 |
| 2 | hsa_3755 | D03878 |
| 2 | hsa_3756 | D00283 |
| 2 | hsa_3756 | D00293 |
| 2 | hsa_3756 | D00332 |
| 2 | hsa_3756 | D00538 |
| 2 | hsa_3756 | D00563 |
| 2 | hsa_3756 | D00593 |
| 2 | hsa_3756 | D00616 |
| 2 | hsa_3756 | D00617 |
| 2 | hsa_3756 | D00633 |
| 2 | hsa_3756 | D00639 |
| 2 | hsa_3756 | D00648 |
| 2 | hsa_3756 | D00654 |
| 2 | hsa_3756 | D00678 |
| 2 | hsa_3756 | D00735 |
| 2 | hsa_3756 | D00739 |
| 2 | hsa_3756 | D00771 |
| 2 | hsa_3756 | D00779 |
| 2 | hsa_3756 | D01599 |
| 2 | hsa_3756 | D01969 |
| 2 | hsa_3756 | D02087 |
| 2 | hsa_3756 | D02207 |
| 2 | hsa_3756 | D02237 |
| 2 | hsa_3756 | D02253 |
| 2 | hsa_3756 | D02914 |
| 2 | hsa_3756 | D03826 |
| 2 | hsa_3756 | D04999 |
| 2 | hsa_3756 | D05156 |
| 2 | hsa_3757 | D00451 |
| 2 | hsa_3757 | D00499 |
| 2 | hsa_3757 | D00512 |
| 2 | hsa_3757 | D00533 |
| 2 | hsa_3757 | D00538 |
| 2 | hsa_3757 | D00607 |
| 2 | hsa_3757 | D00816 |
| 2 | hsa_3757 | D01243 |
| 2 | hsa_3757 | D01253 |
| 2 | hsa_3757 | D02088 |
| 2 | hsa_3757 | D04985 |
| 2 | hsa_3757 | D04999 |
| 2 | hsa_3757 | D05024 |
| 2 | hsa_3757 | D06106 |
| 2 | hsa_3758 | D00195 |
| 2 | hsa_3758 | D00319 |
| 2 | hsa_3758 | D00329 |
| 2 | hsa_3758 | D00351 |
| 2 | hsa_3758 | D00499 |
| 2 | hsa_3758 | D00504 |
| 2 | hsa_3758 | D00519 |
| 2 | hsa_3758 | D00647 |
| 2 | hsa_3758 | D00658 |
| 2 | hsa_3758 | D00771 |
| 2 | hsa_3758 | D00779 |
| 2 | hsa_3758 | D02204 |
| 2 | hsa_3759 | D00499 |
| 2 | hsa_3759 | D00533 |
| 2 | hsa_3759 | D00549 |
| 2 | hsa_3759 | D00636 |
| 2 | hsa_3759 | D01253 |
| 2 | hsa_3759 | D02163 |
| 2 | hsa_3759 | D02566 |
| 2 | hsa_3759 | D02630 |
| 2 | hsa_3759 | D05461 |
| 2 | hsa_3760 | D00136 |
| 2 | hsa_3760 | D00225 |
| 2 | hsa_3760 | D00477 |
| 2 | hsa_3760 | D00513 |
| 2 | hsa_3760 | D00524 |
| 2 | hsa_3760 | D00574 |
| 2 | hsa_3760 | D00618 |
| 2 | hsa_3760 | D00732 |
| 2 | hsa_3760 | D00960 |
| 2 | hsa_3760 | D00964 |
| 2 | hsa_3760 | D01256 |
| 2 | hsa_3760 | D02485 |
| 2 | hsa_3760 | D06106 |
| 2 | hsa_3761 | D00225 |
| 2 | hsa_3761 | D00329 |
| 2 | hsa_3761 | D00332 |
| 2 | hsa_3761 | D00335 |
| 2 | hsa_3761 | D00379 |
| 2 | hsa_3761 | D00456 |
| 2 | hsa_3761 | D00513 |
| 2 | hsa_3761 | D00536 |
| 2 | hsa_3761 | D00553 |
| 2 | hsa_3761 | D00618 |
| 2 | hsa_3761 | D00709 |
| 2 | hsa_3761 | D00791 |
| 2 | hsa_3761 | D01111 |
| 2 | hsa_3761 | D02092 |
| 2 | hsa_3761 | D02173 |
| 2 | hsa_3761 | D02485 |
| 2 | hsa_3761 | D04999 |
| 2 | hsa_3762 | D00110 |
| 2 | hsa_3762 | D00136 |
| 2 | hsa_3762 | D00274 |
| 2 | hsa_3762 | D00464 |
| 2 | hsa_3762 | D00548 |
| 2 | hsa_3762 | D00549 |
| 2 | hsa_3762 | D00594 |
| 2 | hsa_3762 | D00649 |
| 2 | hsa_3762 | D00775 |
| 2 | hsa_3762 | D00960 |
| 2 | hsa_3762 | D02086 |
| 2 | hsa_3762 | D02566 |
| 2 | hsa_3762 | D03037 |
| 2 | hsa_3762 | D03826 |
| 2 | hsa_3762 | D03830 |
| 2 | hsa_3762 | D06172 |
| 2 | hsa_3763 | D00227 |
| 2 | hsa_3763 | D00519 |
| 2 | hsa_3763 | D00537 |
| 2 | hsa_3763 | D00548 |
| 2 | hsa_3763 | D02088 |
| 2 | hsa_3763 | D02546 |
| 2 | hsa_3763 | D02630 |
| 2 | hsa_3763 | D06172 |
| 2 | hsa_3764 | D00110 |
| 2 | hsa_3764 | D00512 |
| 2 | hsa_3764 | D00513 |
| 2 | hsa_3764 | D00550 |
| 2 | hsa_3764 | D00639 |
| 2 | hsa_3764 | D00649 |
| 2 | hsa_3764 | D00964 |
| 2 | hsa_3764 | D01295 |
| 2 | hsa_3764 | D01603 |
| 2 | hsa_3764 | D01969 |
| 2 | hsa_3764 | D02630 |
| 2 | hsa_3764 | D03037 |
| 2 | hsa_3764 | D04790 |
| 2 | hsa_3764 | D04999 |
| 2 | hsa_3764 | D05461 |
| 2 | hsa_3765 | D00335 |
| 2 | hsa_3765 | D00340 |
| 2 | hsa_3765 | D00364 |
| 2 | hsa_3765 | D00521 |
| 2 | hsa_3765 | D00542 |
| 2 | hsa_3765 | D00636 |
| 2 | hsa_3765 | D00639 |
| 2 | hsa_3765 | D01575 |
| 2 | hsa_3765 | D02088 |
| 2 | hsa_3765 | D02237 |
| 2 | hsa_3765 | D02409 |
| 2 | hsa_3765 | D02461 |
| 2 | hsa_3765 | D02630 |
| 2 | hsa_3765 | D04999 |
| 2 | hsa_3765 | D06106 |
| 2 | hsa_3766 | D00136 |
| 2 | hsa_3766 | D00252 |
| 2 | hsa_3766 | D00331 |
| 2 | hsa_3766 | D00547 |
| 2 | hsa_3766 | D00607 |
| 2 | hsa_3766 | D00677 |
| 2 | hsa_3766 | D00963 |
| 2 | hsa_3766 | D04985 |
| 2 | hsa_3767 | D00294 |
| 2 | hsa_3767 | D00331 |
| 2 | hsa_3767 | D00340 |
| 2 | hsa_3767 | D00392 |
| 2 | hsa_3767 | D00524 |
| 2 | hsa_3767 | D00594 |
| 2 | hsa_3767 | D00631 |
| 2 | hsa_3767 | D00636 |
| 2 | hsa_3767 | D00647 |
| 2 | hsa_3767 | D00654 |
| 2 | hsa_3767 | D00677 |
| 2 | hsa_3767 | D00733 |
| 2 | hsa_3767 | D01118 |
| 2 | hsa_3767 | D02546 |
| 2 | hsa_3767 | D05461 |
| 2 | hsa_3768 | D00252 |
| 2 | hsa_3768 | D00303 |
| 2 | hsa_3768 | D00319 |
| 2 | hsa_3768 | D00358 |
| 2 | hsa_3768 | D00394 |
| 2 | hsa_3768 | D00456 |
| 2 | hsa_3768 | D00521 |
| 2 | hsa_3768 | D00533 |
| 2 | hsa_3768 | D00607 |
| 2 | hsa_3768 | D00611 |
| 2 | hsa_3768 | D00633 |
| 2 | hsa_3768 | D00677 |
| 2 | hsa_3768 | D00760 |
| 2 | hsa_3768 | D01854 |
| 2 | hsa_3768 | D02360 |
| 2 | hsa_3768 | D05461 |
| 2 | hsa_3769 | D00110 |
| 2 | hsa_3769 | D00326 |
| 2 | hsa_3769 | D00373 |
| 2 | hsa_3769 | D00379 |
| 2 | hsa_3769 | D00513 |
| 2 | hsa_3769 | D00574 |
| 2 | hsa_3769 | D00633 |
| 2 | hsa_3769 | D00640 |
| 2 | hsa_3769 | D00735 |
| 2 | hsa_3769 | D00739 |
| 2 | hsa_3769 | D01108 |
| 2 | hsa_3769 | D02173 |
| 2 | hsa_3769 | D02207 |
| 2 | hsa_3769 | D02461 |
| 2 | hsa_3769 | D02914 |
| 2 | hsa_3769 | D03878 |
| 2 | hsa_3772 | D00110 |
| 2 | hsa_3772 | D00228 |
| 2 | hsa_3772 | D00437 |
| 2 | hsa_3772 | D00451 |
| 2 | hsa_3772 | D00456 |
| 2 | hsa_3772 | D00480 |
| 2 | hsa_3772 | D00512 |
| 2 | hsa_3772 | D00544 |
| 2 | hsa_3772 | D00765 |
| 2 | hsa_3772 | D01071 |
| 2 | hsa_3772 | D01450 |
| 2 | hsa_3772 | D01599 |
| 2 | hsa_3772 | D01603 |
| 2 | hsa_3772 | D02101 |
| 2 | hsa_3772 | D02163 |
| 2 | hsa_3772 | D02173 |
| 2 | hsa_3772 | D02485 |
| 2 | hsa_3772 | D03037 |
| 2 | hsa_3773 | D00234 |
| 2 | hsa_3773 | D00379 |
| 2 | hsa_3773 | D00495 |
| 2 | hsa_3773 | D00560 |
| 2 | hsa_3773 | D00658 |
| 2 | hsa_3773 | D00739 |
| 2 | hsa_3773 | D01575 |
| 2 | hsa_3773 | D01768 |
| 2 | hsa_3773 | D01854 |
| 2 | hsa_3773 | D02088 |
| 2 | hsa_3773 | D02546 |
| 2 | hsa_3773 | D03450 |
| 2 | hsa_3773 | D04985 |
| 2 | hsa_3773 | D05461 |
| 2 | hsa_3775 | D00319 |
| 2 | hsa_3775 | D00367 |
| 2 | hsa_3775 | D00373 |
| 2 | hsa_3775 | D00594 |
| 2 | hsa_3775 | D00618 |
| 2 | hsa_3775 | D00629 |
| 2 | hsa_3775 | D00633 |
| 2 | hsa_3775 | D00640 |
| 2 | hsa_3775 | D00651 |
| 2 | hsa_3775 | D00960 |
| 2 | hsa_3775 | D01448 |
| 2 | hsa_3775 | D01854 |
| 2 | hsa_3775 | D02253 |
| 2 | hsa_3775 | D02272 |
| 2 | hsa_3775 | D03037 |
| 2 | hsa_3776 | D00552 |
| 2 | hsa_3776 | D00633 |
| 2 | hsa_3776 | D00656 |
| 2 | hsa_3776 | D00711 |
| 2 | hsa_3776 | D00765 |
| 2 | hsa_3776 | D00798 |
| 2 | hsa_3776 | D00964 |
| 2 | hsa_3776 | D01295 |
| 2 | hsa_3776 | D02207 |
| 2 | hsa_3776 | D03830 |
| 2 | hsa_3776 | D04790 |
| 2 | hsa_3776 | D04985 |
| 2 | hsa_3778 | D00035 |
| 2 | hsa_3778 | D00252 |
| 2 | hsa_3778 | D00293 |
| 2 | hsa_3778 | D00354 |
| 2 | hsa_3778 | D00533 |
| 2 | hsa_3778 | D00552 |
| 2 | hsa_3778 | D00649 |
| 2 | hsa_3778 | D00726 |
| 2 | hsa_3778 | D01295 |
| 2 | hsa_3778 | D01603 |
| 2 | hsa_3778 | D01768 |
| 2 | hsa_3778 | D01854 |
| 2 | hsa_3778 | D02261 |
| 2 | hsa_3778 | D04999 |
| 2 | hsa_3778 | D05156 |
| 2 | hsa_3779 | D00110 |
| 2 | hsa_3779 | D00252 |
| 2 | hsa_3779 | D00335 |
| 2 | hsa_3779 | D00340 |
| 2 | hsa_3779 | D00358 |
| 2 | hsa_3779 | D00477 |
| 2 | hsa_3779 | D00504 |
| 2 | hsa_3779 | D00631 |
| 2 | hsa_3779 | D00654 |
| 2 | hsa_3779 | D00656 |
| 2 | hsa_3779 | D01243 |
| 2 | hsa_3779 | D02204 |
| 2 | hsa_3779 | D02237 |
| 2 | hsa_3780 | D00225 |
| 2 | hsa_3780 | D00228 |
| 2 | hsa_3780 | D00336 |
| 2 | hsa_3780 | D00340 |
| 2 | hsa_3780 | D00380 |
| 2 | hsa_3780 | D00521 |
| 2 | hsa_3780 | D00533 |
| 2 | hsa_3780 | D00545 |
| 2 | hsa_3780 | D00574 |
| 2 | hsa_3780 | D00642 |
| 2 | hsa_3780 | D00694 |
| 2 | hsa_3780 | D01295 |
| 2 | hsa_3780 | D01603 |
| 2 | hsa_3780 | D02087 |
| 2 | hsa_3780 | D03826 |
| 2 | hsa_3780 | D06172 |
| 2 | hsa_3781 | D00110 |
| 2 | hsa_3781 | D00225 |
| 2 | hsa_3781 | D00228 |
| 2 | hsa_3781 | D00234 |
| 2 | hsa_3781 | D00283 |
| 2 | hsa_3781 | D00319 |
| 2 | hsa_3781 | D00349 |
| 2 | hsa_3781 | D00367 |
| 2 | hsa_3781 | D00456 |
| 2 | hsa_3781 | D00499 |
| 2 | hsa_3781 | D00512 |
| 2 | hsa_3781 | D00549 |
| 2 | hsa_3781 | D00618 |
| 2 | hsa_3781 | D00640 |
| 2 | hsa_3781 | D00677 |
| 2 | hsa_3781 | D00740 |
| 2 | hsa_3781 | D00759 |
| 2 | hsa_3781 | D00816 |
| 2 | hsa_3781 | D01071 |
| 2 | hsa_3781 | D01768 |
| 2 | hsa_3781 | D02204 |
| 2 | hsa_3781 | D02461 |
| 2 | hsa_3781 | D03830 |
| 2 | hsa_3782 | D00303 |
| 2 | hsa_3782 | D00331 |
| 2 | hsa_3782 | D00335 |
| 2 | hsa_3782 | D00418 |
| 2 | hsa_3782 | D00437 |
| 2 | hsa_3782 | D00548 |
| 2 | hsa_3782 | D00550 |
| 2 | hsa_3782 | D00594 |
| 2 | hsa_3782 | D00618 |
| 2 | hsa_3782 | D00619 |
| 2 | hsa_3782 | D00648 |
| 2 | hsa_3782 | D00694 |
| 2 | hsa_3782 | D00733 |
| 2 | hsa_3782 | D01071 |
| 2 | hsa_3782 | D01799 |
| 2 | hsa_3782 | D04790 |
| 2 | hsa_3783 | D00110 |
| 2 | hsa_3783 | D00332 |
| 2 | hsa_3783 | D00336 |
| 2 | hsa_3783 | D00345 |
| 2 | hsa_3783 | D00358 |
| 2 | hsa_3783 | D00546 |
| 2 | hsa_3783 | D00548 |
| 2 | hsa_3783 | D00553 |
| 2 | hsa_3783 | D02237 |
| 2 | hsa_3784 | D00354 |
| 2 | hsa_3784 | D00456 |
| 2 | hsa_3784 | D00543 |
| 2 | hsa_3784 | D00547 |
| 2 | hsa_3784 | D00640 |
| 2 | hsa_3784 | D00650 |
| 2 | hsa_3784 | D00779 |
| 2 | hsa_3784 | D00816 |
| 2 | hsa_3784 | D01969 |
| 2 | hsa_3784 | D02092 |
| 2 | hsa_3784 | D02360 |
| 2 | hsa_3784 | D02485 |
| 2 | hsa_3784 | D03878 |
| 2 | hsa_3784 | D06172 |
| 2 | hsa_3785 | D00329 |
| 2 | hsa_3785 | D00332 |
| 2 | hsa_3785 | D00373 |
| 2 | hsa_3785 | D00536 |
| 2 | hsa_3785 | D00545 |
| 2 | hsa_3785 | D00563 |
| 2 | hsa_3785 | D00593 |
| 2 | hsa_3785 | D00611 |
| 2 | hsa_3785 | D00650 |
| 2 | hsa_3785 | D00709 |
| 2 | hsa_3785 | D00732 |
| 2 | hsa_3785 | D01242 |
| 2 | hsa_3785 | D01603 |
| 2 | hsa_3785 | D04999 |
| 2 | hsa_3785 | D05156 |
| 2 | hsa_3786 | D00136 |
| 2 | hsa_3786 | D00319 |
| 2 | hsa_3786 | D00351 |
| 2 | hsa_3786 | D00495 |
| 2 | hsa_3786 | D00536 |
| 2 | hsa_3786 | D00544 |
| 2 | hsa_3786 | D00546 |
| 2 | hsa_3786 | D00631 |
| 2 | hsa_3786 | D00709 |
| 2 | hsa_3786 | D00738 |
| 2 | hsa_3786 | D02041 |
| 2 | hsa_3786 | D02092 |
| 2 | hsa_3786 | D05024 |
| 2 | hsa_3787 | D00331 |
| 2 | hsa_3787 | D00335 |
| 2 | hsa_3787 | D00392 |
| 2 | hsa_3787 | D00456 |
| 2 | hsa_3787 | D00474 |
| 2 | hsa_3787 | D00530 |
| 2 | hsa_3787 | D00636 |
| 2 | hsa_3787 | D00648 |
| 2 | hsa_3787 | D00649 |
| 2 | hsa_3787 | D01108 |
| 2 | hsa_3787 | D01243 |
| 2 | hsa_3787 | D01256 |
| 2 | hsa_3787 | D02098 |
| 2 | hsa_3787 | D03274 |
| 2 | hsa_3787 | D03878 |
| 2 | hsa_3787 | D04034 |
| 2 | hsa_3787 | D06106 |
| 2 | hsa_3788 | D00234 |
| 2 | hsa_3788 | D00349 |
| 2 | hsa_3788 | D00537 |
| 2 | hsa_3788 | D00615 |
| 2 | hsa_3788 | D00642 |
| 2 | hsa_3788 | D00650 |
| 2 | hsa_3788 | D00738 |
| 2 | hsa_3788 | D00775 |
| 2 | hsa_3788 | D00823 |
| 2 | hsa_3788 | D01242 |
| 2 | hsa_3788 | D01799 |
| 2 | hsa_3788 | D02086 |
| 2 | hsa_3788 | D04985 |
| 2 | hsa_3788 | D05461 |
| 2 | hsa_3790 | D00340 |
| 2 | hsa_3790 | D00380 |
| 2 | hsa_3790 | D00538 |
| 2 | hsa_3790 | D00543 |
| 2 | hsa_3790 | D00771 |
| 2 | hsa_3790 | D02253 |
| 2 | hsa_3790 | D03450 |
| 2 | hsa_3790 | D04790 |
| 2 | hsa_40 | D00437 |
| 2 | hsa_40 | D00539 |
| 2 | hsa_40 | D00553 |
| 2 | hsa_40 | D00726 |
| 2 | hsa_40 | D02086 |
| 2 | hsa_40 | D02092 |
| 2 | hsa_40 | D02101 |
| 2 | hsa_40 | D02630 |
| 2 | hsa_40 | D03991 |
| 2 | hsa_40 | D05453 |
| 2 | hsa_40 | D06106 |
| 2 | hsa_41 | D00234 |
| 2 | hsa_41 | D00252 |
| 2 | hsa_41 | D00283 |
| 2 | hsa_41 | D00513 |
| 2 | hsa_41 | D00524 |
| 2 | hsa_41 | D00553 |
| 2 | hsa_41 | D00607 |
| 2 | hsa_41 | D00633 |
| 2 | hsa_41 | D00732 |
| 2 | hsa_41 | D02261 |
| 2 | hsa_41 | D05156 |
| 2 | hsa_5023 | D00294 |
| 2 | hsa_5023 | D00303 |
| 2 | hsa_5023 | D00345 |
| 2 | hsa_5023 | D00438 |
| 2 | hsa_5023 | D00480 |
| 2 | hsa_5023 | D00546 |
| 2 | hsa_5023 | D00553 |
| 2 | hsa_5023 | D00615 |
| 2 | hsa_5023 | D00636 |
| 2 | hsa_5023 | D00677 |
| 2 | hsa_5023 | D00711 |
| 2 | hsa_5023 | D01243 |
| 2 | hsa_5023 | D02163 |
| 2 | hsa_5023 | D02204 |
| 2 | hsa_5023 | D03450 |
| 2 | hsa_5023 | D05024 |
| 2 | hsa_5024 | D00228 |
| 2 | hsa_5024 | D00332 |
| 2 | hsa_5024 | D00474 |
| 2 | hsa_5024 | D00512 |
| 2 | hsa_5024 | D00618 |
| 2 | hsa_5024 | D00708 |
| 2 | hsa_5024 | D00760 |
| 2 | hsa_5024 | D01242 |
| 2 | hsa_5024 | D02362 |
| 2 | hsa_5024 | D02409 |
| 2 | hsa_5024 | D03878 |
| 2 | hsa_5025 | D00364 |
| 2 | hsa_5025 | D00451 |
| 2 | hsa_5025 | D00477 |
| 2 | hsa_5025 | D00512 |
| 2 | hsa_5025 | D00530 |
| 2 | hsa_5025 | D00536 |
| 2 | hsa_5025 | D00543 |
| 2 | hsa_5025 | D00548 |
| 2 | hsa_5025 | D00642 |
| 2 | hsa_5025 | D00677 |
| 2 | hsa_5025 | D01071 |
| 2 | hsa_5025 | D01295 |
| 2 | hsa_5025 | D02362 |
| 2 | hsa_5026 | D00338 |
| 2 | hsa_5026 | D00499 |
| 2 | hsa_5026 | D00521 |
| 2 | hsa_5026 | D00533 |
| 2 | hsa_5026 | D00547 |
| 2 | hsa_5026 | D00574 |
| 2 | hsa_5026 | D00649 |
| 2 | hsa_5026 | D00651 |
| 2 | hsa_5026 | D00798 |
| 2 | hsa_5026 | D02098 |
| 2 | hsa_5026 | D02485 |
| 2 | hsa_5026 | D02630 |
| 2 | hsa_5026 | D04985 |
| 2 | hsa_5026 | D04999 |
| 2 | hsa_5026 | D05453 |
| 2 | hsa_5026 | D05461 |
| 2 | hsa_5026 | D06172 |
| 2 | hsa_5027 | D00110 |
| 2 | hsa_5027 | D00319 |
| 2 | hsa_5027 | D00451 |
| 2 | hsa_5027 | D00480 |
| 2 | hsa_5027 | D00539 |
| 2 | hsa_5027 | D00638 |
| 2 | hsa_5027 | D00658 |
| 2 | hsa_5027 | D00708 |
| 2 | hsa_5027 | D00738 |
| 2 | hsa_5027 | D00765 |
| 2 | hsa_5027 | D00771 |
| 2 | hsa_5027 | D01108 |
| 2 | hsa_5027 | D01256 |
| 2 | hsa_5027 | D02163 |
| 2 | hsa_5027 | D03742 |
| 2 | hsa_50801 | D00195 |
| 2 | hsa_50801 | D00227 |
| 2 | hsa_50801 | D00274 |
| 2 | hsa_50801 | D00513 |
| 2 | hsa_50801 | D00542 |
| 2 | hsa_50801 | D00544 |
| 2 | hsa_50801 | D00545 |
| 2 | hsa_50801 | D00547 |
| 2 | hsa_50801 | D00563 |
| 2 | hsa_50801 | D00617 |
| 2 | hsa_50801 | D00697 |
| 2 | hsa_50801 | D00732 |
| 2 | hsa_50801 | D01108 |
| 2 | hsa_50801 | D01450 |
| 2 | hsa_50801 | D02461 |
| 2 | hsa_50801 | D02914 |
| 2 | hsa_50801 | D03830 |
| 2 | hsa_50801 | D03991 |
| 2 | hsa_50801 | D05024 |
| 2 | hsa_51305 | D00136 |
| 2 | hsa_51305 | D00340 |
| 2 | hsa_51305 | D00354 |
| 2 | hsa_51305 | D00373 |
| 2 | hsa_51305 | D00611 |
| 2 | hsa_51305 | D00616 |
| 2 | hsa_51305 | D00629 |
| 2 | hsa_51305 | D00647 |
| 2 | hsa_51305 | D00704 |
| 2 | hsa_51305 | D00726 |
| 2 | hsa_51305 | D00740 |
| 2 | hsa_51305 | D01453 |
| 2 | hsa_51305 | D02088 |
| 2 | hsa_51305 | D02098 |
| 2 | hsa_51305 | D02237 |
| 2 | hsa_51305 | D02253 |
| 2 | hsa_51802 | D00456 |
| 2 | hsa_51802 | D00477 |
| 2 | hsa_51802 | D00530 |
| 2 | hsa_51802 | D00704 |
| 2 | hsa_51802 | D00809 |
| 2 | hsa_51802 | D00963 |
| 2 | hsa_51802 | D02485 |
| 2 | hsa_51802 | D05024 |
| 2 | hsa_51802 | D05156 |
| 2 | hsa_51802 | D05461 |
| 2 | hsa_54207 | D00219 |
| 2 | hsa_54207 | D00225 |
| 2 | hsa_54207 | D00326 |
| 2 | hsa_54207 | D00345 |
| 2 | hsa_54207 | D00364 |
| 2 | hsa_54207 | D00418 |
| 2 | hsa_54207 | D00499 |
| 2 | hsa_54207 | D00544 |
| 2 | hsa_54207 | D00619 |
| 2 | hsa_54207 | D00697 |
| 2 | hsa_54207 | D00779 |
| 2 | hsa_54207 | D00798 |
| 2 | hsa_54207 | D02088 |
| 2 | hsa_54207 | D02092 |
| 2 | hsa_54207 | D02101 |
| 2 | hsa_55503 | D00294 |
| 2 | hsa_55503 | D00513 |
| 2 | hsa_55503 | D00543 |
| 2 | hsa_55503 | D00549 |
| 2 | hsa_55503 | D00593 |
| 2 | hsa_55503 | D00648 |
| 2 | hsa_55503 | D00740 |
| 2 | hsa_55503 | D00741 |
| 2 | hsa_55503 | D01242 |
| 2 | hsa_55503 | D02101 |
| 2 | hsa_55503 | D05077 |
| 2 | hsa_55515 | D00035 |
| 2 | hsa_55515 | D00110 |
| 2 | hsa_55515 | D00195 |
| 2 | hsa_55515 | D00227 |
| 2 | hsa_55515 | D00319 |
| 2 | hsa_55515 | D00336 |
| 2 | hsa_55515 | D00542 |
| 2 | hsa_55515 | D00553 |
| 2 | hsa_55515 | D00560 |
| 2 | hsa_55515 | D00617 |
| 2 | hsa_55515 | D00631 |
| 2 | hsa_55515 | D00650 |
| 2 | hsa_55515 | D00740 |
| 2 | hsa_55515 | D00823 |
| 2 | hsa_55515 | D01108 |
| 2 | hsa_55515 | D02163 |
| 2 | hsa_55515 | D02261 |
| 2 | hsa_55515 | D02262 |
| 2 | hsa_55515 | D03180 |
| 2 | hsa_55515 | D04985 |
| 2 | hsa_55515 | D05458 |
| 2 | hsa_55584 | D00351 |
| 2 | hsa_55584 | D00358 |
| 2 | hsa_55584 | D00379 |
| 2 | hsa_55584 | D00550 |
| 2 | hsa_55584 | D00593 |
| 2 | hsa_55584 | D00615 |
| 2 | hsa_55584 | D00631 |
| 2 | hsa_55584 | D00709 |
| 2 | hsa_55584 | D00735 |
| 2 | hsa_55584 | D00791 |
| 2 | hsa_55584 | D00809 |
| 2 | hsa_55584 | D02261 |
| 2 | hsa_55584 | D05077 |
| 2 | hsa_55584 | D05461 |
| 2 | hsa_55799 | D00326 |
| 2 | hsa_55799 | D00380 |
| 2 | hsa_55799 | D00464 |
| 2 | hsa_55799 | D00524 |
| 2 | hsa_55799 | D00617 |
| 2 | hsa_55799 | D00619 |
| 2 | hsa_55799 | D00648 |
| 2 | hsa_55799 | D00694 |
| 2 | hsa_55799 | D00771 |
| 2 | hsa_55799 | D01242 |
| 2 | hsa_55799 | D01599 |
| 2 | hsa_55800 | D00294 |
| 2 | hsa_55800 | D00364 |
| 2 | hsa_55800 | D00474 |
| 2 | hsa_55800 | D00678 |
| 2 | hsa_55800 | D00694 |
| 2 | hsa_55800 | D00735 |
| 2 | hsa_55800 | D00739 |
| 2 | hsa_55800 | D01448 |
| 2 | hsa_55800 | D02088 |
| 2 | hsa_55800 | D02092 |
| 2 | hsa_55800 | D02261 |
| 2 | hsa_55800 | D03274 |
| 2 | hsa_55800 | D04034 |
| 2 | hsa_55879 | D00367 |
| 2 | hsa_55879 | D00394 |
| 2 | hsa_55879 | D00438 |
| 2 | hsa_55879 | D00616 |
| 2 | hsa_55879 | D00677 |
| 2 | hsa_55879 | D00678 |
| 2 | hsa_55879 | D00733 |
| 2 | hsa_55879 | D01253 |
| 2 | hsa_55879 | D01295 |
| 2 | hsa_55879 | D01575 |
| 2 | hsa_55879 | D02409 |
| 2 | hsa_56479 | D00195 |
| 2 | hsa_56479 | D00331 |
| 2 | hsa_56479 | D00394 |
| 2 | hsa_56479 | D00528 |
| 2 | hsa_56479 | D00530 |
| 2 | hsa_56479 | D00619 |
| 2 | hsa_56479 | D00639 |
| 2 | hsa_56479 | D00651 |
| 2 | hsa_56479 | D01111 |
| 2 | hsa_56659 | D00303 |
| 2 | hsa_56659 | D00504 |
| 2 | hsa_56659 | D00512 |
| 2 | hsa_56659 | D00574 |
| 2 | hsa_56659 | D00611 |
| 2 | hsa_56659 | D00711 |
| 2 | hsa_56659 | D01071 |
| 2 | hsa_56659 | D01969 |
| 2 | hsa_56659 | D02088 |
| 2 | hsa_56659 | D02092 |
| 2 | hsa_56659 | D06106 |
| 2 | hsa_56660 | D00336 |
| 2 | hsa_56660 | D00380 |
| 2 | hsa_56660 | D00456 |
| 2 | hsa_56660 | D01799 |
| 2 | hsa_56660 | D02237 |
| 2 | hsa_56660 | D05461 |
| 2 | hsa_57053 | D00283 |
| 2 | hsa_57053 | D00332 |
| 2 | hsa_57053 | D00513 |
| 2 | hsa_57053 | D00530 |
| 2 | hsa_57053 | D00538 |
| 2 | hsa_57053 | D00553 |
| 2 | hsa_57053 | D00642 |
| 2 | hsa_57053 | D00759 |
| 2 | hsa_57053 | D01448 |
| 2 | hsa_57053 | D01599 |
| 2 | hsa_57053 | D02237 |
| 2 | hsa_57053 | D02360 |
| 2 | hsa_57053 | D02409 |
| 2 | hsa_57053 | D02566 |
| 2 | hsa_57053 | D04034 |
| 2 | hsa_57053 | D06106 |
| 2 | hsa_57192 | D00195 |
| 2 | hsa_57192 | D00464 |
| 2 | hsa_57192 | D00677 |
| 2 | hsa_57192 | D00738 |
| 2 | hsa_57192 | D01253 |
| 2 | hsa_57192 | D01256 |
| 2 | hsa_57192 | D02262 |
| 2 | hsa_57192 | D04034 |
| 2 | hsa_57192 | D04790 |
| 2 | hsa_57192 | D05458 |
| 2 | hsa_57657 | D00225 |
| 2 | hsa_57657 | D00252 |
| 2 | hsa_57657 | D00380 |
| 2 | hsa_57657 | D00438 |
| 2 | hsa_57657 | D00524 |
| 2 | hsa_57657 | D00547 |
| 2 | hsa_57657 | D00615 |
| 2 | hsa_57657 | D00650 |
| 2 | hsa_57657 | D00656 |
| 2 | hsa_57657 | D00733 |
| 2 | hsa_57657 | D00735 |
| 2 | hsa_57657 | D00738 |
| 2 | hsa_57657 | D00760 |
| 2 | hsa_57657 | D00812 |
| 2 | hsa_57657 | D01243 |
| 2 | hsa_57657 | D02098 |
| 2 | hsa_57657 | D06172 |
| 2 | hsa_59284 | D00219 |
| 2 | hsa_59284 | D00364 |
| 2 | hsa_59284 | D00380 |
| 2 | hsa_59284 | D00521 |
| 2 | hsa_59284 | D00546 |
| 2 | hsa_59284 | D00553 |
| 2 | hsa_59284 | D00563 |
| 2 | hsa_59284 | D00593 |
| 2 | hsa_59284 | D00704 |
| 2 | hsa_59284 | D01111 |
| 2 | hsa_59284 | D01256 |
| 2 | hsa_59284 | D06172 |
| 2 | hsa_60598 | D00340 |
| 2 | hsa_60598 | D00367 |
| 2 | hsa_60598 | D00380 |
| 2 | hsa_60598 | D00392 |
| 2 | hsa_60598 | D00418 |
| 2 | hsa_60598 | D00513 |
| 2 | hsa_60598 | D00536 |
| 2 | hsa_60598 | D00539 |
| 2 | hsa_60598 | D00809 |
| 2 | hsa_60598 | D00964 |
| 2 | hsa_60598 | D01118 |
| 2 | hsa_60598 | D01448 |
| 2 | hsa_60598 | D01712 |
| 2 | hsa_60598 | D01828 |
| 2 | hsa_60598 | D02087 |
| 2 | hsa_60598 | D02088 |
| 2 | hsa_60598 | D04790 |
| 2 | hsa_6323 | D00227 |
| 2 | hsa_6323 | D00349 |
| 2 | hsa_6323 | D00351 |
| 2 | hsa_6323 | D00394 |
| 2 | hsa_6323 | D00528 |
| 2 | hsa_6323 | D00617 |
| 2 | hsa_6323 | D00629 |
| 2 | hsa_6323 | D00651 |
| 2 | hsa_6323 | D00771 |
| 2 | hsa_6323 | D00791 |
| 2 | hsa_6323 | D00823 |
| 2 | hsa_6323 | D02237 |
| 2 | hsa_6323 | D02261 |
| 2 | hsa_6323 | D02546 |
| 2 | hsa_6323 | D03742 |
| 2 | hsa_6323 | D04999 |
| 2 | hsa_6324 | D00136 |
| 2 | hsa_6324 | D00524 |
| 2 | hsa_6324 | D00548 |
| 2 | hsa_6324 | D00642 |
| 2 | hsa_6324 | D00648 |
| 2 | hsa_6324 | D00708 |
| 2 | hsa_6324 | D00799 |
| 2 | hsa_6324 | D00963 |
| 2 | hsa_6324 | D03826 |
| 2 | hsa_6324 | D03878 |
| 2 | hsa_6324 | D03991 |
| 2 | hsa_6324 | D04034 |
| 2 | hsa_6326 | D00227 |
| 2 | hsa_6326 | D00293 |
| 2 | hsa_6326 | D00303 |
| 2 | hsa_6326 | D00319 |
| 2 | hsa_6326 | D00326 |
| 2 | hsa_6326 | D00338 |
| 2 | hsa_6326 | D00464 |
| 2 | hsa_6326 | D00513 |
| 2 | hsa_6326 | D00537 |
| 2 | hsa_6326 | D00636 |
| 2 | hsa_6326 | D00694 |
| 2 | hsa_6326 | D00798 |
| 2 | hsa_6326 | D01575 |
| 2 | hsa_6326 | D02237 |
| 2 | hsa_6326 | D02262 |
| 2 | hsa_6326 | D05024 |
| 2 | hsa_6326 | D06106 |
| 2 | hsa_6328 | D00136 |
| 2 | hsa_6328 | D00225 |
| 2 | hsa_6328 | D00319 |
| 2 | hsa_6328 | D00345 |
| 2 | hsa_6328 | D00373 |
| 2 | hsa_6328 | D00543 |
| 2 | hsa_6328 | D00651 |
| 2 | hsa_6328 | D00694 |
| 2 | hsa_6328 | D00726 |
| 2 | hsa_6328 | D00760 |
| 2 | hsa_6328 | D02173 |
| 2 | hsa_6328 | D02253 |
| 2 | hsa_6328 | D02409 |
| 2 | hsa_6328 | D03830 |
| 2 | hsa_6329 | D00294 |
| 2 | hsa_6329 | D00329 |
| 2 | hsa_6329 | D00550 |
| 2 | hsa_6329 | D01108 |
| 2 | hsa_6329 | D01599 |
| 2 | hsa_6329 | D01854 |
| 2 | hsa_6329 | D02262 |
| 2 | hsa_6329 | D02461 |
| 2 | hsa_6329 | D03450 |
| 2 | hsa_6330 | D00331 |
| 2 | hsa_6330 | D00338 |
| 2 | hsa_6330 | D00464 |
| 2 | hsa_6330 | D00521 |
| 2 | hsa_6330 | D00548 |
| 2 | hsa_6330 | D00633 |
| 2 | hsa_6330 | D00694 |
| 2 | hsa_6330 | D00732 |
| 2 | hsa_6330 | D00733 |
| 2 | hsa_6330 | D00771 |
| 2 | hsa_6330 | D00964 |
| 2 | hsa_6330 | D01111 |
| 2 | hsa_6330 | D02409 |
| 2 | hsa_6330 | D04034 |
| 2 | hsa_6331 | D00035 |
| 2 | hsa_6331 | D00136 |
| 2 | hsa_6331 | D00234 |
| 2 | hsa_6331 | D00392 |
| 2 | hsa_6331 | D00647 |
| 2 | hsa_6331 | D02204 |
| 2 | hsa_6331 | D03830 |
| 2 | hsa_6332 | D00234 |
| 2 | hsa_6332 | D00474 |
| 2 | hsa_6332 | D00499 |
| 2 | hsa_6332 | D00537 |
| 2 | hsa_6332 | D00539 |
| 2 | hsa_6332 | D00545 |
| 2 | hsa_6332 | D00550 |
| 2 | hsa_6332 | D00563 |
| 2 | hsa_6332 | D00616 |
| 2 | hsa_6332 | D00636 |
| 2 | hsa_6332 | D00650 |
| 2 | hsa_6332 | D00732 |
| 2 | hsa_6332 | D00738 |
| 2 | hsa_6332 | D00740 |
| 2 | hsa_6332 | D00759 |
| 2 | hsa_6332 | D01071 |
| 2 | hsa_6332 | D01256 |
| 2 | hsa_6332 | D01453 |
| 2 | hsa_6332 | D01599 |
| 2 | hsa_6332 | D02204 |
| 2 | hsa_6332 | D02461 |
| 2 | hsa_6332 | D02630 |
| 2 | hsa_6332 | D03037 |
| 2 | hsa_6332 | D03830 |
| 2 | hsa_6334 | D00326 |
| 2 | hsa_6334 | D00437 |
| 2 | hsa_6334 | D00451 |
| 2 | hsa_6334 | D00547 |
| 2 | hsa_6334 | D00549 |
| 2 | hsa_6334 | D00574 |
| 2 | hsa_6334 | D00697 |
| 2 | hsa_6334 | D01118 |
| 2 | hsa_6334 | D01828 |
| 2 | hsa_6334 | D02461 |
| 2 | hsa_6335 | D00418 |
| 2 | hsa_6335 | D00451 |
| 2 | hsa_6335 | D00539 |
| 2 | hsa_6335 | D00560 |
| 2 | hsa_6335 | D00658 |
| 2 | hsa_6335 | D01253 |
| 2 | hsa_6335 | D02041 |
| 2 | hsa_6335 | D03830 |
| 2 | hsa_6336 | D00351 |
| 2 | hsa_6336 | D00354 |
| 2 | hsa_6336 | D00380 |
| 2 | hsa_6336 | D00438 |
| 2 | hsa_6336 | D00480 |
| 2 | hsa_6336 | D00524 |
| 2 | hsa_6336 | D00618 |
| 2 | hsa_6336 | D00631 |
| 2 | hsa_6336 | D00708 |
| 2 | hsa_6336 | D00709 |
| 2 | hsa_6336 | D00798 |
| 2 | hsa_6336 | D02234 |
| 2 | hsa_6336 | D03878 |
| 2 | hsa_6337 | D00136 |
| 2 | hsa_6337 | D00294 |
| 2 | hsa_6337 | D00418 |
| 2 | hsa_6337 | D00647 |
| 2 | hsa_6337 | D00678 |
| 2 | hsa_6337 | D00708 |
| 2 | hsa_6337 | D00779 |
| 2 | hsa_6337 | D00791 |
| 2 | hsa_6337 | D01287 |
| 2 | hsa_6337 | D01295 |
| 2 | hsa_6337 | D03274 |
| 2 | hsa_6337 | D05458 |
| 2 | hsa_6338 | D00349 |
| 2 | hsa_6338 | D00477 |
| 2 | hsa_6338 | D00560 |
| 2 | hsa_6338 | D00607 |
| 2 | hsa_6338 | D00708 |
| 2 | hsa_6338 | D00726 |
| 2 | hsa_6338 | D00735 |
| 2 | hsa_6338 | D01071 |
| 2 | hsa_6338 | D01108 |
| 2 | hsa_6338 | D01453 |
| 2 | hsa_6338 | D01768 |
| 2 | hsa_6338 | D02272 |
| 2 | hsa_6338 | D02409 |
| 2 | hsa_6338 | D02485 |
| 2 | hsa_6338 | D03830 |
| 2 | hsa_6338 | D04999 |
| 2 | hsa_6338 | D06106 |
| 2 | hsa_6339 | D00537 |
| 2 | hsa_6339 | D00560 |
| 2 | hsa_6339 | D00574 |
| 2 | hsa_6339 | D00617 |
| 2 | hsa_6339 | D00629 |
| 2 | hsa_6339 | D00636 |
| 2 | hsa_6339 | D00640 |
| 2 | hsa_6339 | D00735 |
| 2 | hsa_6339 | D01071 |
| 2 | hsa_6339 | D01108 |
| 2 | hsa_6339 | D02092 |
| 2 | hsa_6339 | D02207 |
| 2 | hsa_6339 | D02261 |
| 2 | hsa_6339 | D02360 |
| 2 | hsa_6340 | D00274 |
| 2 | hsa_6340 | D00345 |
| 2 | hsa_6340 | D00495 |
| 2 | hsa_6340 | D00524 |
| 2 | hsa_6340 | D00536 |
| 2 | hsa_6340 | D00539 |
| 2 | hsa_6340 | D00636 |
| 2 | hsa_6340 | D00678 |
| 2 | hsa_6340 | D00709 |
| 2 | hsa_6340 | D00964 |
| 2 | hsa_6340 | D01111 |
| 2 | hsa_6340 | D01243 |
| 2 | hsa_6340 | D01603 |
| 2 | hsa_6340 | D01712 |
| 2 | hsa_6340 | D02173 |
| 2 | hsa_6340 | D02234 |
| 2 | hsa_6340 | D02630 |
| 2 | hsa_6340 | D03878 |
| 2 | hsa_6340 | D05453 |
| 2 | hsa_6529 | D00225 |
| 2 | hsa_6529 | D00345 |
| 2 | hsa_6529 | D00456 |
| 2 | hsa_6529 | D00477 |
| 2 | hsa_6529 | D00499 |
| 2 | hsa_6529 | D00616 |
| 2 | hsa_6529 | D00771 |
| 2 | hsa_6529 | D01448 |
| 2 | hsa_6529 | D01603 |
| 2 | hsa_6529 | D01969 |
| 2 | hsa_6529 | D02101 |
| 2 | hsa_6529 | D02234 |
| 2 | hsa_6529 | D03037 |
| 2 | hsa_6529 | D03742 |
| 2 | hsa_6530 | D00252 |
| 2 | hsa_6530 | D00294 |
| 2 | hsa_6530 | D00329 |
| 2 | hsa_6530 | D00335 |
| 2 | hsa_6530 | D00499 |
| 2 | hsa_6530 | D00524 |
| 2 | hsa_6530 | D00546 |
| 2 | hsa_6530 | D00616 |
| 2 | hsa_6530 | D00631 |
| 2 | hsa_6530 | D00678 |
| 2 | hsa_6530 | D01854 |
| 2 | hsa_6530 | D01969 |
| 2 | hsa_6530 | D02101 |
| 2 | hsa_6530 | D02234 |
| 2 | hsa_6530 | D02262 |
| 2 | hsa_6530 | D02362 |
| 2 | hsa_6530 | D03450 |
| 2 | hsa_6531 | D00274 |
| 2 | hsa_6531 | D00474 |
| 2 | hsa_6531 | D00519 |
| 2 | hsa_6531 | D00697 |
| 2 | hsa_6531 | D00732 |
| 2 | hsa_6531 | D00741 |
| 2 | hsa_6531 | D00760 |
| 2 | hsa_6531 | D00791 |
| 2 | hsa_6531 | D00964 |
| 2 | hsa_6531 | D02204 |
| 2 | hsa_6531 | D02914 |
| 2 | hsa_6531 | D05077 |
| 2 | hsa_6532 | D00331 |
| 2 | hsa_6532 | D00364 |
| 2 | hsa_6532 | D00438 |
| 2 | hsa_6532 | D00495 |
| 2 | hsa_6532 | D00513 |
| 2 | hsa_6532 | D00543 |
| 2 | hsa_6532 | D00546 |
| 2 | hsa_6532 | D00616 |
| 2 | hsa_6532 | D00633 |
| 2 | hsa_6532 | D00654 |
| 2 | hsa_6532 | D00656 |
| 2 | hsa_6532 | D00733 |
| 2 | hsa_6532 | D01118 |
| 2 | hsa_6532 | D01242 |
| 2 | hsa_6532 | D02086 |
| 2 | hsa_6532 | D03180 |
| 2 | hsa_6534 | D00329 |
| 2 | hsa_6534 | D00351 |
| 2 | hsa_6534 | D00354 |
| 2 | hsa_6534 | D00379 |
| 2 | hsa_6534 | D00499 |
| 2 | hsa_6534 | D00538 |
| 2 | hsa_6534 | D00615 |
| 2 | hsa_6534 | D00638 |
| 2 | hsa_6534 | D00654 |
| 2 | hsa_6534 | D00761 |
| 2 | hsa_6534 | D01287 |
| 2 | hsa_6534 | D02207 |
| 2 | hsa_6534 | D02272 |
| 2 | hsa_6534 | D02461 |
| 2 | hsa_6833 | D00219 |
| 2 | hsa_6833 | D00234 |
| 2 | hsa_6833 | D00513 |
| 2 | hsa_6833 | D00528 |
| 2 | hsa_6833 | D00539 |
| 2 | hsa_6833 | D00552 |
| 2 | hsa_6833 | D00648 |
| 2 | hsa_6833 | D00739 |
| 2 | hsa_6833 | D00964 |
| 2 | hsa_6833 | D01118 |
| 2 | hsa_6833 | D01453 |
| 2 | hsa_6833 | D01603 |
| 2 | hsa_6833 | D02086 |
| 2 | hsa_6833 | D02087 |
| 2 | hsa_6833 | D02546 |
| 2 | hsa_7225 | D00225 |
| 2 | hsa_7225 | D00274 |
| 2 | hsa_7225 | D00303 |
| 2 | hsa_7225 | D00326 |
| 2 | hsa_7225 | D00331 |
| 2 | hsa_7225 | D00332 |
| 2 | hsa_7225 | D00379 |
| 2 | hsa_7225 | D00392 |
| 2 | hsa_7225 | D00524 |
| 2 | hsa_7225 | D00538 |
| 2 | hsa_7225 | D00545 |
| 2 | hsa_7225 | D00548 |
| 2 | hsa_7225 | D00648 |
| 2 | hsa_7225 | D00677 |
| 2 | hsa_7225 | D00791 |
| 2 | hsa_7225 | D00809 |
| 2 | hsa_7225 | D01450 |
| 2 | hsa_7225 | D01712 |
| 2 | hsa_7225 | D02253 |
| 2 | hsa_7225 | D03991 |
| 2 | hsa_7225 | D05024 |
| 2 | hsa_7225 | D05453 |
| 2 | hsa_773 | D00274 |
| 2 | hsa_773 | D00340 |
| 2 | hsa_773 | D00364 |
| 2 | hsa_773 | D00464 |
| 2 | hsa_773 | D00647 |
| 2 | hsa_773 | D00823 |
| 2 | hsa_773 | D01575 |
| 2 | hsa_773 | D02087 |
| 2 | hsa_773 | D02092 |
| 2 | hsa_773 | D02362 |
| 2 | hsa_773 | D05156 |
| 2 | hsa_774 | D00336 |
| 2 | hsa_774 | D00364 |
| 2 | hsa_774 | D00379 |
| 2 | hsa_774 | D00380 |
| 2 | hsa_774 | D00495 |
| 2 | hsa_774 | D00499 |
| 2 | hsa_774 | D00537 |
| 2 | hsa_774 | D00648 |
| 2 | hsa_774 | D00708 |
| 2 | hsa_774 | D00740 |
| 2 | hsa_774 | D00779 |
| 2 | hsa_774 | D01108 |
| 2 | hsa_774 | D03274 |
| 2 | hsa_775 | D00228 |
| 2 | hsa_775 | D00252 |
| 2 | hsa_775 | D00338 |
| 2 | hsa_775 | D00477 |
| 2 | hsa_775 | D00528 |
| 2 | hsa_775 | D00649 |
| 2 | hsa_775 | D00759 |
| 2 | hsa_775 | D00760 |
| 2 | hsa_775 | D00771 |
| 2 | hsa_775 | D00779 |
| 2 | hsa_775 | D00823 |
| 2 | hsa_775 | D02272 |
| 2 | hsa_775 | D02485 |
| 2 | hsa_775 | D03991 |
| 2 | hsa_775 | D05461 |
| 2 | hsa_776 | D00504 |
| 2 | hsa_776 | D00524 |
| 2 | hsa_776 | D00538 |
| 2 | hsa_776 | D00640 |
| 2 | hsa_776 | D00647 |
| 2 | hsa_776 | D00658 |
| 2 | hsa_776 | D00678 |
| 2 | hsa_776 | D00708 |
| 2 | hsa_776 | D00735 |
| 2 | hsa_776 | D01242 |
| 2 | hsa_776 | D01969 |
| 2 | hsa_776 | D02086 |
| 2 | hsa_776 | D02207 |
| 2 | hsa_776 | D03037 |
| 2 | hsa_776 | D03878 |
| 2 | hsa_776 | D04034 |
| 2 | hsa_776 | D05156 |
| 2 | hsa_777 | D00234 |
| 2 | hsa_777 | D00294 |
| 2 | hsa_777 | D00367 |
| 2 | hsa_777 | D00549 |
| 2 | hsa_777 | D00651 |
| 2 | hsa_777 | D00694 |
| 2 | hsa_777 | D00732 |
| 2 | hsa_777 | D00735 |
| 2 | hsa_777 | D01111 |
| 2 | hsa_777 | D01243 |
| 2 | hsa_777 | D01253 |
| 2 | hsa_777 | D01575 |
| 2 | hsa_777 | D02204 |
| 2 | hsa_777 | D02546 |
| 2 | hsa_777 | D04034 |
| 2 | hsa_777 | D06106 |
| 2 | hsa_778 | D00225 |
| 2 | hsa_778 | D00252 |
| 2 | hsa_778 | D00283 |
| 2 | hsa_778 | D00335 |
| 2 | hsa_778 | D00336 |
| 2 | hsa_778 | D00547 |
| 2 | hsa_778 | D00678 |
| 2 | hsa_778 | D00759 |
| 2 | hsa_778 | D01575 |
| 2 | hsa_778 | D02485 |
| 2 | hsa_778 | D05077 |
| 2 | hsa_778 | D05461 |
| 2 | hsa_779 | D00293 |
| 2 | hsa_779 | D00335 |
| 2 | hsa_779 | D00658 |
| 2 | hsa_779 | D00678 |
| 2 | hsa_779 | D00708 |
| 2 | hsa_779 | D01108 |
| 2 | hsa_779 | D01287 |
| 2 | hsa_779 | D01448 |
| 2 | hsa_779 | D02262 |
| 2 | hsa_779 | D05077 |
| 2 | hsa_779 | D06172 |
| 2 | hsa_781 | D00274 |
| 2 | hsa_781 | D00495 |
| 2 | hsa_781 | D00548 |
| 2 | hsa_781 | D00611 |
| 2 | hsa_781 | D00638 |
| 2 | hsa_781 | D00640 |
| 2 | hsa_781 | D00649 |
| 2 | hsa_781 | D00654 |
| 2 | hsa_781 | D00678 |
| 2 | hsa_781 | D00704 |
| 2 | hsa_781 | D01242 |
| 2 | hsa_781 | D02204 |
| 2 | hsa_781 | D02630 |
| 2 | hsa_782 | D00293 |
| 2 | hsa_782 | D00331 |
| 2 | hsa_782 | D00379 |
| 2 | hsa_782 | D00499 |
| 2 | hsa_782 | D00504 |
| 2 | hsa_782 | D00533 |
| 2 | hsa_782 | D00651 |
| 2 | hsa_782 | D00738 |
| 2 | hsa_782 | D00816 |
| 2 | hsa_782 | D01108 |
| 2 | hsa_782 | D01256 |
| 2 | hsa_782 | D02101 |
| 2 | hsa_782 | D02234 |
| 2 | hsa_782 | D02261 |
| 2 | hsa_782 | D02272 |
| 2 | hsa_782 | D02461 |
| 2 | hsa_783 | D00228 |
| 2 | hsa_783 | D00418 |
| 2 | hsa_783 | D00480 |
| 2 | hsa_783 | D00499 |
| 2 | hsa_783 | D00536 |
| 2 | hsa_783 | D00549 |
| 2 | hsa_783 | D00658 |
| 2 | hsa_783 | D00738 |
| 2 | hsa_783 | D00759 |
| 2 | hsa_783 | D00765 |
| 2 | hsa_783 | D01108 |
| 2 | hsa_783 | D01256 |
| 2 | hsa_783 | D01712 |
| 2 | hsa_783 | D02092 |
| 2 | hsa_783 | D02485 |
| 2 | hsa_783 | D02630 |
| 2 | hsa_784 | D00392 |
| 2 | hsa_784 | D00474 |
| 2 | hsa_784 | D00477 |
| 2 | hsa_784 | D00539 |
| 2 | hsa_784 | D00552 |
| 2 | hsa_784 | D00633 |
| 2 | hsa_784 | D00640 |
| 2 | hsa_784 | D00708 |
| 2 | hsa_784 | D00761 |
| 2 | hsa_784 | D00799 |
| 2 | hsa_784 | D00960 |
| 2 | hsa_784 | D00964 |
| 2 | hsa_784 | D01712 |
| 2 | hsa_784 | D01828 |
| 2 | hsa_784 | D02485 |
| 2 | hsa_785 | D00335 |
| 2 | hsa_785 | D00373 |
| 2 | hsa_785 | D00480 |
| 2 | hsa_785 | D00537 |
| 2 | hsa_785 | D00633 |
| 2 | hsa_785 | D00639 |
| 2 | hsa_785 | D00650 |
| 2 | hsa_785 | D02101 |
| 2 | hsa_785 | D02207 |
| 2 | hsa_785 | D02409 |
| 2 | hsa_785 | D02461 |
| 2 | hsa_785 | D02566 |
| 2 | hsa_785 | D05077 |
| 2 | hsa_785 | D06172 |
| 2 | hsa_786 | D00354 |
| 2 | hsa_786 | D00451 |
| 2 | hsa_786 | D00708 |
| 2 | hsa_786 | D00760 |
| 2 | hsa_786 | D02485 |
| 2 | hsa_786 | D03180 |
| 2 | hsa_786 | D06172 |
| 2 | hsa_7881 | D00219 |
| 2 | hsa_7881 | D00234 |
| 2 | hsa_7881 | D00451 |
| 2 | hsa_7881 | D00456 |
| 2 | hsa_7881 | D00512 |
| 2 | hsa_7881 | D00519 |
| 2 | hsa_7881 | D00547 |
| 2 | hsa_7881 | D00574 |
| 2 | hsa_7881 | D00640 |
| 2 | hsa_7881 | D01242 |
| 2 | hsa_7881 | D01256 |
| 2 | hsa_7881 | D01453 |
| 2 | hsa_7881 | D02409 |
| 2 | hsa_7881 | D03742 |
| 2 | hsa_7881 | D03991 |
| 2 | hsa_7881 | D04034 |
| 2 | hsa_7881 | D06106 |
| 2 | hsa_8001 | D00219 |
| 2 | hsa_8001 | D00294 |
| 2 | hsa_8001 | D00499 |
| 2 | hsa_8001 | D00738 |
| 2 | hsa_8001 | D01854 |
| 2 | hsa_8001 | D02204 |
| 2 | hsa_8001 | D03037 |
| 2 | hsa_8001 | D03878 |
| 2 | hsa_8001 | D05077 |
| 2 | hsa_81033 | D00234 |
| 2 | hsa_81033 | D00319 |
| 2 | hsa_81033 | D00345 |
| 2 | hsa_81033 | D00358 |
| 2 | hsa_81033 | D00364 |
| 2 | hsa_81033 | D00379 |
| 2 | hsa_81033 | D00394 |
| 2 | hsa_81033 | D00544 |
| 2 | hsa_81033 | D00640 |
| 2 | hsa_81033 | D00654 |
| 2 | hsa_81033 | D00708 |
| 2 | hsa_81033 | D00711 |
| 2 | hsa_81033 | D00963 |
| 2 | hsa_81033 | D02485 |
| 2 | hsa_83795 | D00283 |
| 2 | hsa_83795 | D00394 |
| 2 | hsa_83795 | D00464 |
| 2 | hsa_83795 | D00544 |
| 2 | hsa_83795 | D00617 |
| 2 | hsa_83795 | D00639 |
| 2 | hsa_83795 | D00678 |
| 2 | hsa_83795 | D00761 |
| 2 | hsa_83795 | D00775 |
| 2 | hsa_83795 | D02253 |
| 2 | hsa_83795 | D02409 |
| 2 | hsa_83795 | D02546 |
| 2 | hsa_83795 | D05156 |
| 2 | hsa_8514 | D00274 |
| 2 | hsa_8514 | D00335 |
| 2 | hsa_8514 | D00418 |
| 2 | hsa_8514 | D00611 |
| 2 | hsa_8514 | D00697 |
| 2 | hsa_8514 | D00726 |
| 2 | hsa_8514 | D00779 |
| 2 | hsa_8514 | D00963 |
| 2 | hsa_8514 | D01243 |
| 2 | hsa_8514 | D01256 |
| 2 | hsa_8514 | D01287 |
| 2 | hsa_8514 | D01854 |
| 2 | hsa_8514 | D02409 |
| 2 | hsa_8514 | D05024 |
| 2 | hsa_8645 | D00035 |
| 2 | hsa_8645 | D00438 |
| 2 | hsa_8645 | D00528 |
| 2 | hsa_8645 | D00615 |
| 2 | hsa_8645 | D01111 |
| 2 | hsa_8645 | D01253 |
| 2 | hsa_8645 | D02092 |
| 2 | hsa_8911 | D00225 |
| 2 | hsa_8911 | D00252 |
| 2 | hsa_8911 | D00319 |
| 2 | hsa_8911 | D00354 |
| 2 | hsa_8911 | D00480 |
| 2 | hsa_8911 | D00519 |
| 2 | hsa_8911 | D00543 |
| 2 | hsa_8911 | D00549 |
| 2 | hsa_8911 | D00593 |
| 2 | hsa_8911 | D00647 |
| 2 | hsa_8911 | D00697 |
| 2 | hsa_8911 | D00735 |
| 2 | hsa_8911 | D00738 |
| 2 | hsa_8911 | D00761 |
| 2 | hsa_8911 | D01448 |
| 2 | hsa_8911 | D01969 |
| 2 | hsa_8911 | D02272 |
| 2 | hsa_8911 | D03830 |
| 2 | hsa_8912 | D00234 |
| 2 | hsa_8912 | D00456 |
| 2 | hsa_8912 | D00464 |
| 2 | hsa_8912 | D00528 |
| 2 | hsa_8912 | D00543 |
| 2 | hsa_8912 | D00546 |
| 2 | hsa_8912 | D00552 |
| 2 | hsa_8912 | D00726 |
| 2 | hsa_8912 | D00740 |
| 2 | hsa_8912 | D01575 |
| 2 | hsa_8912 | D02409 |
| 2 | hsa_8912 | D03037 |
| 2 | hsa_8912 | D04790 |
| 2 | hsa_8913 | D00035 |
| 2 | hsa_8913 | D00274 |
| 2 | hsa_8913 | D00293 |
| 2 | hsa_8913 | D00530 |
| 2 | hsa_8913 | D00640 |
| 2 | hsa_8913 | D00816 |
| 2 | hsa_8913 | D02253 |
| 2 | hsa_8913 | D02546 |
| 2 | hsa_8913 | D02566 |
| 2 | hsa_8913 | D03037 |
| 2 | hsa_8913 | D05156 |
| 2 | hsa_8913 | D05453 |
| 2 | hsa_8973 | D00329 |
| 2 | hsa_8973 | D00574 |
| 2 | hsa_8973 | D00651 |
| 2 | hsa_8973 | D00658 |
| 2 | hsa_8973 | D00709 |
| 2 | hsa_8973 | D00809 |
| 2 | hsa_8973 | D00823 |
| 2 | hsa_8973 | D01118 |
| 2 | hsa_8973 | D01243 |
| 2 | hsa_8973 | D02272 |
| 2 | hsa_89822 | D00252 |
| 2 | hsa_89822 | D00338 |
| 2 | hsa_89822 | D00354 |
| 2 | hsa_89822 | D00418 |
| 2 | hsa_89822 | D00456 |
| 2 | hsa_89822 | D00519 |
| 2 | hsa_89822 | D00544 |
| 2 | hsa_89822 | D00553 |
| 2 | hsa_89822 | D00560 |
| 2 | hsa_89822 | D00648 |
| 2 | hsa_89822 | D00960 |
| 2 | hsa_89822 | D01450 |
| 2 | hsa_89822 | D02092 |
| 2 | hsa_89822 | D02272 |
| 2 | hsa_89822 | D02409 |
| 2 | hsa_89822 | D03830 |
| 2 | hsa_89822 | D05156 |
| 2 | hsa_8989 | D00225 |
| 2 | hsa_8989 | D00319 |
| 2 | hsa_8989 | D00335 |
| 2 | hsa_8989 | D00474 |
| 2 | hsa_8989 | D00521 |
| 2 | hsa_8989 | D00533 |
| 2 | hsa_8989 | D00536 |
| 2 | hsa_8989 | D00550 |
| 2 | hsa_8989 | D00631 |
| 2 | hsa_8989 | D00733 |
| 2 | hsa_8989 | D00740 |
| 2 | hsa_8989 | D00775 |
| 2 | hsa_8989 | D00831 |
| 2 | hsa_8989 | D00963 |
| 2 | hsa_8989 | D01071 |
| 2 | hsa_8989 | D01243 |
| 2 | hsa_8989 | D01253 |
| 2 | hsa_8989 | D01287 |
| 2 | hsa_8989 | D01599 |
| 2 | hsa_8989 | D02207 |
| 2 | hsa_8989 | D03037 |
| 2 | hsa_8989 | D03830 |
| 2 | hsa_90134 | D00219 |
| 2 | hsa_90134 | D00340 |
| 2 | hsa_90134 | D00392 |
| 2 | hsa_90134 | D00524 |
| 2 | hsa_90134 | D00548 |
| 2 | hsa_90134 | D00574 |
| 2 | hsa_90134 | D00812 |
| 2 | hsa_90134 | D00964 |
| 2 | hsa_90134 | D03274 |
| 2 | hsa_9127 | D00195 |
| 2 | hsa_9127 | D00274 |
| 2 | hsa_9127 | D00495 |
| 2 | hsa_9127 | D00545 |
| 2 | hsa_9127 | D00638 |
| 2 | hsa_9127 | D00654 |
| 2 | hsa_9127 | D00677 |
| 2 | hsa_9127 | D00765 |
| 2 | hsa_9127 | D00799 |
| 2 | hsa_9127 | D00823 |
| 2 | hsa_9127 | D00960 |
| 2 | hsa_9127 | D01287 |
| 2 | hsa_9127 | D02163 |
| 2 | hsa_9127 | D02237 |
| 2 | hsa_9127 | D02630 |
| 2 | hsa_9127 | D03037 |
| 2 | hsa_9127 | D03991 |
| 2 | hsa_9127 | D05156 |
| 2 | hsa_9132 | D00349 |
| 2 | hsa_9132 | D00392 |
| 2 | hsa_9132 | D00530 |
| 2 | hsa_9132 | D00547 |
| 2 | hsa_9132 | D00549 |
| 2 | hsa_9132 | D00594 |
| 2 | hsa_9132 | D00619 |
| 2 | hsa_9132 | D00642 |
| 2 | hsa_9132 | D02086 |
| 2 | hsa_9132 | D02204 |
| 2 | hsa_9177 | D00110 |
| 2 | hsa_9177 | D00354 |
| 2 | hsa_9177 | D00373 |
| 2 | hsa_9177 | D00495 |
| 2 | hsa_9177 | D00524 |
| 2 | hsa_9177 | D00543 |
| 2 | hsa_9177 | D00631 |
| 2 | hsa_9177 | D00760 |
| 2 | hsa_9177 | D00798 |
| 2 | hsa_9177 | D01599 |
| 2 | hsa_9177 | D02261 |
| 2 | hsa_9177 | D02546 |
| 2 | hsa_9177 | D03037 |
| 2 | hsa_9177 | D04999 |
| 2 | hsa_9177 | D06172 |
| 2 | hsa_9254 | D00294 |
| 2 | hsa_9254 | D00499 |
| 2 | hsa_9254 | D00537 |
| 2 | hsa_9254 | D00563 |
| 2 | hsa_9254 | D00607 |
| 2 | hsa_9254 | D00650 |
| 2 | hsa_9254 | D00739 |
| 2 | hsa_9254 | D00765 |
| 2 | hsa_9254 | D01071 |
| 2 | hsa_9254 | D01575 |
| 2 | hsa_9254 | D01599 |
| 2 | hsa_9254 | D01712 |
| 2 | hsa_9254 | D03826 |
| 2 | hsa_9254 | D05156 |
| 2 | hsa_93107 | D00542 |
| 2 | hsa_93107 | D00543 |
| 2 | hsa_93107 | D00553 |
| 2 | hsa_93107 | D00649 |
| 2 | hsa_93107 | D00740 |
| 2 | hsa_93107 | D01854 |
| 2 | hsa_93107 | D02087 |
| 2 | hsa_93107 | D02092 |
| 2 | hsa_93107 | D02207 |
| 2 | hsa_93107 | D02362 |
| 2 | hsa_9311 | D00228 |
| 2 | hsa_9311 | D00331 |
| 2 | hsa_9311 | D00537 |
| 2 | hsa_9311 | D00563 |
| 2 | hsa_9311 | D00607 |
| 2 | hsa_9311 | D00629 |
| 2 | hsa_9311 | D00651 |
| 2 | hsa_9311 | D00654 |
| 2 | hsa_9311 | D00816 |
| 2 | hsa_9311 | D01108 |
| 2 | hsa_9311 | D02360 |
| 2 | hsa_9311 | D04985 |
| 2 | hsa_9312 | D00195 |
| 2 | hsa_9312 | D00228 |
| 2 | hsa_9312 | D00329 |
| 2 | hsa_9312 | D00345 |
| 2 | hsa_9312 | D00495 |
| 2 | hsa_9312 | D00629 |
| 2 | hsa_9312 | D00639 |
| 2 | hsa_9312 | D00648 |
| 2 | hsa_9312 | D00709 |
| 2 | hsa_9312 | D00963 |
| 2 | hsa_9312 | D02253 |
| 2 | hsa_9312 | D02360 |
| 2 | hsa_9312 | D05077 |
| 2 | hsa_93589 | D00136 |
| 2 | hsa_93589 | D00219 |
| 2 | hsa_93589 | D00225 |
| 2 | hsa_93589 | D00228 |
| 2 | hsa_93589 | D00234 |
| 2 | hsa_93589 | D00293 |
| 2 | hsa_93589 | D00480 |
| 2 | hsa_93589 | D00538 |
| 2 | hsa_93589 | D00636 |
| 2 | hsa_93589 | D00648 |
| 2 | hsa_93589 | D00740 |
| 2 | hsa_93589 | D00812 |
| 2 | hsa_93589 | D02087 |
| 2 | hsa_93589 | D03826 |
| 2 | hsa_93589 | D03991 |
| 2 | hsa_9424 | D00219 |
| 2 | hsa_9424 | D00338 |
| 2 | hsa_9424 | D00373 |
| 2 | hsa_9424 | D00546 |
| 2 | hsa_9424 | D00616 |
| 2 | hsa_9424 | D00619 |
| 2 | hsa_9424 | D00629 |
| 2 | hsa_9424 | D00631 |
| 2 | hsa_9424 | D00649 |
| 2 | hsa_9424 | D00650 |
| 2 | hsa_9424 | D00779 |
| 2 | hsa_9424 | D00823 |
| 2 | hsa_9424 | D01242 |
| 2 | hsa_9424 | D01295 |
| 2 | hsa_9424 | D01768 |
| 2 | hsa_9424 | D02262 |
| 2 | hsa_9424 | D02409 |
| 2 | hsa_9424 | D03742 |
| 2 | hsa_9992 | D00303 |
| 2 | hsa_9992 | D00319 |
| 2 | hsa_9992 | D00326 |
| 2 | hsa_9992 | D00332 |
| 2 | hsa_9992 | D00358 |
| 2 | hsa_9992 | D00521 |
| 2 | hsa_9992 | D00708 |
| 2 | hsa_9992 | D00709 |
| 2 | hsa_9992 | D00963 |
| 2 | hsa_9992 | D01118 |
| 2 | hsa_9992 | D02092 |
| 2 | hsa_9992 | D02173 |
