# Supplementary material for: Predicting Drug-Target Interaction Networks Based on Functional Groups and Biological Features
Source: PLoS One. 2010 Mar 11;5(3):e9603. doi: 10.1371/journal.pone.0009603 (PMC2836373; doi:10.1371/journal.pone.0009603)
Supplement: Online Supporting Information S3 — The benchmark dataset for the drug-target GPCR interaction system. It contains 1,860 gene-drug pair samples, of which 620 are positive and 1,240 negative. The 1st column of the table indicates the nature of samples with 1 for positive and 2 for negative; the 2nd column shows the code of target gene; and the 3rd column shows the code of drug. All the detailed information for the genes and drugs listed here can be found in KEGG via their codes (see the caption of Online Supporting Information A for further explanation). (1.53 MB DOC) [file pone.0009603.s003.doc]

**Online Supporting Information S3**: The benchmark dataset for the drug-target GPCR interaction system. It contains 1,860 gene-drug pair samples, of which 620 are positive and 1,240 negative. The 1st column of the table indicates the nature of samples with 1 for positive and 2 for negative; the 2nd column shows the code of target gene; and the 3rd column shows the code of drug. All the detailed information for the genes and drugs listed here can be found in KEGG via their codes (see the caption of Online Supporting Information A for further explanation).

| **Group** | **Target Gene** | **Drug** |
| --- | --- | --- |
| 1 | hsa_10161 | D00528 |
| 1 | hsa_10800 | D00411 |
| 1 | hsa_10800 | D01828 |
| 1 | hsa_11255 | D00234 |
| 1 | hsa_11255 | D00300 |
| 1 | hsa_11255 | D00318 |
| 1 | hsa_11255 | D00494 |
| 1 | hsa_11255 | D02566 |
| 1 | hsa_1128 | D00113 |
| 1 | hsa_1128 | D00232 |
| 1 | hsa_1128 | D00274 |
| 1 | hsa_1128 | D00283 |
| 1 | hsa_1128 | D00397 |
| 1 | hsa_1128 | D00454 |
| 1 | hsa_1128 | D00465 |
| 1 | hsa_1128 | D00494 |
| 1 | hsa_1128 | D00524 |
| 1 | hsa_1128 | D00525 |
| 1 | hsa_1128 | D00540 |
| 1 | hsa_1128 | D00646 |
| 1 | hsa_1128 | D00715 |
| 1 | hsa_1128 | D00726 |
| 1 | hsa_1128 | D00779 |
| 1 | hsa_1128 | D01103 |
| 1 | hsa_1128 | D01118 |
| 1 | hsa_1128 | D01269 |
| 1 | hsa_1128 | D01297 |
| 1 | hsa_1128 | D01871 |
| 1 | hsa_1128 | D02070 |
| 1 | hsa_1128 | D02327 |
| 1 | hsa_1128 | D02354 |
| 1 | hsa_1128 | D02356 |
| 1 | hsa_1128 | D02361 |
| 1 | hsa_1128 | D03621 |
| 1 | hsa_1128 | D03858 |
| 1 | hsa_1129 | D00113 |
| 1 | hsa_1129 | D00232 |
| 1 | hsa_1129 | D00274 |
| 1 | hsa_1129 | D00283 |
| 1 | hsa_1129 | D00397 |
| 1 | hsa_1129 | D00454 |
| 1 | hsa_1129 | D00465 |
| 1 | hsa_1129 | D00494 |
| 1 | hsa_1129 | D00524 |
| 1 | hsa_1129 | D00540 |
| 1 | hsa_1129 | D00646 |
| 1 | hsa_1129 | D00760 |
| 1 | hsa_1129 | D00765 |
| 1 | hsa_1129 | D00779 |
| 1 | hsa_1129 | D01871 |
| 1 | hsa_1129 | D02070 |
| 1 | hsa_1129 | D02354 |
| 1 | hsa_1129 | D02356 |
| 1 | hsa_1129 | D02361 |
| 1 | hsa_1129 | D03621 |
| 1 | hsa_1129 | D03858 |
| 1 | hsa_1131 | D00113 |
| 1 | hsa_1131 | D00232 |
| 1 | hsa_1131 | D00454 |
| 1 | hsa_1131 | D00494 |
| 1 | hsa_1131 | D01699 |
| 1 | hsa_1131 | D01871 |
| 1 | hsa_1131 | D02070 |
| 1 | hsa_1131 | D02354 |
| 1 | hsa_1131 | D02361 |
| 1 | hsa_1131 | D03621 |
| 1 | hsa_1131 | D03654 |
| 1 | hsa_1131 | D03858 |
| 1 | hsa_1132 | D00113 |
| 1 | hsa_1132 | D00397 |
| 1 | hsa_1132 | D00454 |
| 1 | hsa_1132 | D00494 |
| 1 | hsa_1132 | D01871 |
| 1 | hsa_1132 | D02070 |
| 1 | hsa_1132 | D02354 |
| 1 | hsa_1132 | D02361 |
| 1 | hsa_1133 | D00113 |
| 1 | hsa_1133 | D00454 |
| 1 | hsa_1133 | D00494 |
| 1 | hsa_1133 | D01871 |
| 1 | hsa_1133 | D02070 |
| 1 | hsa_1133 | D02354 |
| 1 | hsa_1133 | D02361 |
| 1 | hsa_1234 | D03210 |
| 1 | hsa_1241 | D00411 |
| 1 | hsa_1268 | D00306 |
| 1 | hsa_134 | D00227 |
| 1 | hsa_134 | D00332 |
| 1 | hsa_134 | D00528 |
| 1 | hsa_134 | D01712 |
| 1 | hsa_134 | D02884 |
| 1 | hsa_135 | D00227 |
| 1 | hsa_135 | D00528 |
| 1 | hsa_135 | D01712 |
| 1 | hsa_135 | D02884 |
| 1 | hsa_136 | D00371 |
| 1 | hsa_136 | D00528 |
| 1 | hsa_136 | D04006 |
| 1 | hsa_140 | D00227 |
| 1 | hsa_140 | D00528 |
| 1 | hsa_146 | D00255 |
| 1 | hsa_146 | D00281 |
| 1 | hsa_146 | D00283 |
| 1 | hsa_146 | D00426 |
| 1 | hsa_146 | D00437 |
| 1 | hsa_146 | D00454 |
| 1 | hsa_146 | D00509 |
| 1 | hsa_146 | D00513 |
| 1 | hsa_146 | D00604 |
| 1 | hsa_146 | D00607 |
| 1 | hsa_146 | D00609 |
| 1 | hsa_146 | D00996 |
| 1 | hsa_146 | D01020 |
| 1 | hsa_146 | D01022 |
| 1 | hsa_146 | D01024 |
| 1 | hsa_146 | D01051 |
| 1 | hsa_146 | D01358 |
| 1 | hsa_146 | D01603 |
| 1 | hsa_146 | D01713 |
| 1 | hsa_146 | D01965 |
| 1 | hsa_146 | D02149 |
| 1 | hsa_146 | D02234 |
| 1 | hsa_146 | D02237 |
| 1 | hsa_146 | D02356 |
| 1 | hsa_146 | D02361 |
| 1 | hsa_147 | D00255 |
| 1 | hsa_147 | D00281 |
| 1 | hsa_147 | D00283 |
| 1 | hsa_147 | D00426 |
| 1 | hsa_147 | D00437 |
| 1 | hsa_147 | D00454 |
| 1 | hsa_147 | D00509 |
| 1 | hsa_147 | D00513 |
| 1 | hsa_147 | D00607 |
| 1 | hsa_147 | D00609 |
| 1 | hsa_147 | D00996 |
| 1 | hsa_147 | D01020 |
| 1 | hsa_147 | D01022 |
| 1 | hsa_147 | D01024 |
| 1 | hsa_147 | D01051 |
| 1 | hsa_147 | D01358 |
| 1 | hsa_147 | D01603 |
| 1 | hsa_147 | D01713 |
| 1 | hsa_147 | D01965 |
| 1 | hsa_147 | D02149 |
| 1 | hsa_147 | D02234 |
| 1 | hsa_147 | D02237 |
| 1 | hsa_147 | D02356 |
| 1 | hsa_147 | D02361 |
| 1 | hsa_148 | D00095 |
| 1 | hsa_148 | D00255 |
| 1 | hsa_148 | D00281 |
| 1 | hsa_148 | D00283 |
| 1 | hsa_148 | D00426 |
| 1 | hsa_148 | D00437 |
| 1 | hsa_148 | D00454 |
| 1 | hsa_148 | D00494 |
| 1 | hsa_148 | D00503 |
| 1 | hsa_148 | D00509 |
| 1 | hsa_148 | D00513 |
| 1 | hsa_148 | D00607 |
| 1 | hsa_148 | D00609 |
| 1 | hsa_148 | D00954 |
| 1 | hsa_148 | D00965 |
| 1 | hsa_148 | D00996 |
| 1 | hsa_148 | D01020 |
| 1 | hsa_148 | D01022 |
| 1 | hsa_148 | D01024 |
| 1 | hsa_148 | D01051 |
| 1 | hsa_148 | D01358 |
| 1 | hsa_148 | D01603 |
| 1 | hsa_148 | D01692 |
| 1 | hsa_148 | D01713 |
| 1 | hsa_148 | D01965 |
| 1 | hsa_148 | D02149 |
| 1 | hsa_148 | D02234 |
| 1 | hsa_148 | D02237 |
| 1 | hsa_148 | D02356 |
| 1 | hsa_148 | D02361 |
| 1 | hsa_148 | D02566 |
| 1 | hsa_148 | D02910 |
| 1 | hsa_150 | D00136 |
| 1 | hsa_150 | D00255 |
| 1 | hsa_150 | D00270 |
| 1 | hsa_150 | D00281 |
| 1 | hsa_150 | D00283 |
| 1 | hsa_150 | D00332 |
| 1 | hsa_150 | D00437 |
| 1 | hsa_150 | D00454 |
| 1 | hsa_150 | D00509 |
| 1 | hsa_150 | D00513 |
| 1 | hsa_150 | D00514 |
| 1 | hsa_150 | D00563 |
| 1 | hsa_150 | D00604 |
| 1 | hsa_150 | D00606 |
| 1 | hsa_150 | D00607 |
| 1 | hsa_150 | D00609 |
| 1 | hsa_150 | D00613 |
| 1 | hsa_150 | D00996 |
| 1 | hsa_150 | D01022 |
| 1 | hsa_150 | D01603 |
| 1 | hsa_150 | D01713 |
| 1 | hsa_150 | D02076 |
| 1 | hsa_150 | D02149 |
| 1 | hsa_150 | D02237 |
| 1 | hsa_150 | D02349 |
| 1 | hsa_150 | D02356 |
| 1 | hsa_150 | D03274 |
| 1 | hsa_150 | D04034 |
| 1 | hsa_150 | D04375 |
| 1 | hsa_151 | D00136 |
| 1 | hsa_151 | D00255 |
| 1 | hsa_151 | D00270 |
| 1 | hsa_151 | D00281 |
| 1 | hsa_151 | D00283 |
| 1 | hsa_151 | D00437 |
| 1 | hsa_151 | D00454 |
| 1 | hsa_151 | D00509 |
| 1 | hsa_151 | D00513 |
| 1 | hsa_151 | D00563 |
| 1 | hsa_151 | D00604 |
| 1 | hsa_151 | D00606 |
| 1 | hsa_151 | D00607 |
| 1 | hsa_151 | D00609 |
| 1 | hsa_151 | D00613 |
| 1 | hsa_151 | D00996 |
| 1 | hsa_151 | D01022 |
| 1 | hsa_151 | D01603 |
| 1 | hsa_151 | D01713 |
| 1 | hsa_151 | D02076 |
| 1 | hsa_151 | D02149 |
| 1 | hsa_151 | D02237 |
| 1 | hsa_151 | D02356 |
| 1 | hsa_151 | D03274 |
| 1 | hsa_151 | D04034 |
| 1 | hsa_152 | D00281 |
| 1 | hsa_152 | D00509 |
| 1 | hsa_152 | D00604 |
| 1 | hsa_152 | D00606 |
| 1 | hsa_152 | D00607 |
| 1 | hsa_152 | D00609 |
| 1 | hsa_152 | D00613 |
| 1 | hsa_152 | D00996 |
| 1 | hsa_152 | D01022 |
| 1 | hsa_152 | D01603 |
| 1 | hsa_152 | D02076 |
| 1 | hsa_152 | D02149 |
| 1 | hsa_152 | D02237 |
| 1 | hsa_152 | D03274 |
| 1 | hsa_152 | D04034 |
| 1 | hsa_153 | D00095 |
| 1 | hsa_153 | D00235 |
| 1 | hsa_153 | D00255 |
| 1 | hsa_153 | D00432 |
| 1 | hsa_153 | D00437 |
| 1 | hsa_153 | D00454 |
| 1 | hsa_153 | D00483 |
| 1 | hsa_153 | D00513 |
| 1 | hsa_153 | D00598 |
| 1 | hsa_153 | D00601 |
| 1 | hsa_153 | D00632 |
| 1 | hsa_153 | D00635 |
| 1 | hsa_153 | D00645 |
| 1 | hsa_153 | D00996 |
| 1 | hsa_153 | D01390 |
| 1 | hsa_153 | D01454 |
| 1 | hsa_153 | D02066 |
| 1 | hsa_153 | D02149 |
| 1 | hsa_153 | D02150 |
| 1 | hsa_153 | D02338 |
| 1 | hsa_153 | D02342 |
| 1 | hsa_153 | D02358 |
| 1 | hsa_153 | D02374 |
| 1 | hsa_153 | D02614 |
| 1 | hsa_153 | D02910 |
| 1 | hsa_153 | D03415 |
| 1 | hsa_153 | D03490 |
| 1 | hsa_153 | D03879 |
| 1 | hsa_153 | D03880 |
| 1 | hsa_153 | D03881 |
| 1 | hsa_153 | D04625 |
| 1 | hsa_154 | D00095 |
| 1 | hsa_154 | D00235 |
| 1 | hsa_154 | D00255 |
| 1 | hsa_154 | D00432 |
| 1 | hsa_154 | D00437 |
| 1 | hsa_154 | D00454 |
| 1 | hsa_154 | D00483 |
| 1 | hsa_154 | D00513 |
| 1 | hsa_154 | D00598 |
| 1 | hsa_154 | D00601 |
| 1 | hsa_154 | D00632 |
| 1 | hsa_154 | D00635 |
| 1 | hsa_154 | D00645 |
| 1 | hsa_154 | D00683 |
| 1 | hsa_154 | D00684 |
| 1 | hsa_154 | D00687 |
| 1 | hsa_154 | D00688 |
| 1 | hsa_154 | D00996 |
| 1 | hsa_154 | D01386 |
| 1 | hsa_154 | D01390 |
| 1 | hsa_154 | D01454 |
| 1 | hsa_154 | D02066 |
| 1 | hsa_154 | D02147 |
| 1 | hsa_154 | D02149 |
| 1 | hsa_154 | D02150 |
| 1 | hsa_154 | D02338 |
| 1 | hsa_154 | D02342 |
| 1 | hsa_154 | D02359 |
| 1 | hsa_154 | D02374 |
| 1 | hsa_154 | D03415 |
| 1 | hsa_154 | D03490 |
| 1 | hsa_154 | D03879 |
| 1 | hsa_154 | D03880 |
| 1 | hsa_154 | D03881 |
| 1 | hsa_154 | D05792 |
| 1 | hsa_155 | D00255 |
| 1 | hsa_155 | D00432 |
| 1 | hsa_155 | D00437 |
| 1 | hsa_155 | D00454 |
| 1 | hsa_155 | D00483 |
| 1 | hsa_155 | D00513 |
| 1 | hsa_155 | D00996 |
| 1 | hsa_155 | D01390 |
| 1 | hsa_155 | D01454 |
| 1 | hsa_155 | D02066 |
| 1 | hsa_155 | D02149 |
| 1 | hsa_155 | D02150 |
| 1 | hsa_155 | D02338 |
| 1 | hsa_155 | D02374 |
| 1 | hsa_155 | D03415 |
| 1 | hsa_155 | D03879 |
| 1 | hsa_1812 | D00059 |
| 1 | hsa_1812 | D00110 |
| 1 | hsa_1812 | D00270 |
| 1 | hsa_1812 | D00283 |
| 1 | hsa_1812 | D00454 |
| 1 | hsa_1812 | D00493 |
| 1 | hsa_1812 | D00503 |
| 1 | hsa_1812 | D00560 |
| 1 | hsa_1812 | D00613 |
| 1 | hsa_1812 | D00790 |
| 1 | hsa_1812 | D01295 |
| 1 | hsa_1812 | D02354 |
| 1 | hsa_1812 | D02361 |
| 1 | hsa_1812 | D02671 |
| 1 | hsa_1813 | D00059 |
| 1 | hsa_1813 | D00136 |
| 1 | hsa_1813 | D00270 |
| 1 | hsa_1813 | D00283 |
| 1 | hsa_1813 | D00454 |
| 1 | hsa_1813 | D00493 |
| 1 | hsa_1813 | D00494 |
| 1 | hsa_1813 | D00503 |
| 1 | hsa_1813 | D00560 |
| 1 | hsa_1813 | D00726 |
| 1 | hsa_1813 | D00780 |
| 1 | hsa_1813 | D00790 |
| 1 | hsa_1813 | D00987 |
| 1 | hsa_1813 | D01164 |
| 1 | hsa_1813 | D01295 |
| 1 | hsa_1813 | D01462 |
| 1 | hsa_1813 | D01745 |
| 1 | hsa_1813 | D02340 |
| 1 | hsa_1813 | D02354 |
| 1 | hsa_1813 | D02361 |
| 1 | hsa_1813 | D02671 |
| 1 | hsa_1813 | D03165 |
| 1 | hsa_1814 | D00110 |
| 1 | hsa_1814 | D00136 |
| 1 | hsa_1814 | D00270 |
| 1 | hsa_1814 | D00454 |
| 1 | hsa_1814 | D00493 |
| 1 | hsa_1814 | D00503 |
| 1 | hsa_1814 | D00559 |
| 1 | hsa_1814 | D00560 |
| 1 | hsa_1814 | D00726 |
| 1 | hsa_1814 | D01164 |
| 1 | hsa_1814 | D01295 |
| 1 | hsa_1814 | D02340 |
| 1 | hsa_1814 | D02671 |
| 1 | hsa_1815 | D00283 |
| 1 | hsa_1815 | D00454 |
| 1 | hsa_1815 | D02354 |
| 1 | hsa_1815 | D02361 |
| 1 | hsa_1816 | D00270 |
| 1 | hsa_1816 | D00283 |
| 1 | hsa_1816 | D00454 |
| 1 | hsa_1816 | D00493 |
| 1 | hsa_1816 | D00560 |
| 1 | hsa_1816 | D00613 |
| 1 | hsa_1816 | D01295 |
| 1 | hsa_1816 | D02671 |
| 1 | hsa_185 | D00400 |
| 1 | hsa_185 | D00443 |
| 1 | hsa_185 | D00522 |
| 1 | hsa_185 | D00523 |
| 1 | hsa_185 | D00627 |
| 1 | hsa_185 | D02082 |
| 1 | hsa_185 | D04040 |
| 1 | hsa_185 | D05246 |
| 1 | hsa_1909 | D01227 |
| 1 | hsa_1910 | D01227 |
| 1 | hsa_222545 | D01126 |
| 1 | hsa_222545 | D02278 |
| 1 | hsa_222545 | D02279 |
| 1 | hsa_23620 | D01717 |
| 1 | hsa_2550 | D00241 |
| 1 | hsa_2846 | D00528 |
| 1 | hsa_2911 | D00775 |
| 1 | hsa_2912 | D00775 |
| 1 | hsa_2913 | D00775 |
| 1 | hsa_2914 | D00775 |
| 1 | hsa_2915 | D00775 |
| 1 | hsa_2916 | D00775 |
| 1 | hsa_2917 | D00775 |
| 1 | hsa_2918 | D00775 |
| 1 | hsa_2918 | D01346 |
| 1 | hsa_3269 | D00234 |
| 1 | hsa_3269 | D00283 |
| 1 | hsa_3269 | D00300 |
| 1 | hsa_3269 | D00364 |
| 1 | hsa_3269 | D00454 |
| 1 | hsa_3269 | D00480 |
| 1 | hsa_3269 | D00493 |
| 1 | hsa_3269 | D00494 |
| 1 | hsa_3269 | D00520 |
| 1 | hsa_3269 | D00521 |
| 1 | hsa_3269 | D00665 |
| 1 | hsa_3269 | D00666 |
| 1 | hsa_3269 | D01242 |
| 1 | hsa_3269 | D01295 |
| 1 | hsa_3269 | D01324 |
| 1 | hsa_3269 | D01332 |
| 1 | hsa_3269 | D01713 |
| 1 | hsa_3269 | D01717 |
| 1 | hsa_3269 | D01782 |
| 1 | hsa_3269 | D02327 |
| 1 | hsa_3269 | D02354 |
| 1 | hsa_3269 | D02361 |
| 1 | hsa_3269 | D02566 |
| 1 | hsa_3269 | D03621 |
| 1 | hsa_3274 | D00295 |
| 1 | hsa_3274 | D00318 |
| 1 | hsa_3274 | D00422 |
| 1 | hsa_3274 | D00440 |
| 1 | hsa_3274 | D00673 |
| 1 | hsa_3274 | D01713 |
| 1 | hsa_3274 | D03503 |
| 1 | hsa_3350 | D00283 |
| 1 | hsa_3350 | D00451 |
| 1 | hsa_3350 | D00513 |
| 1 | hsa_3350 | D00726 |
| 1 | hsa_3350 | D01051 |
| 1 | hsa_3350 | D01164 |
| 1 | hsa_3350 | D01973 |
| 1 | hsa_3351 | D00283 |
| 1 | hsa_3351 | D00415 |
| 1 | hsa_3351 | D00451 |
| 1 | hsa_3351 | D00513 |
| 1 | hsa_3351 | D00675 |
| 1 | hsa_3351 | D00676 |
| 1 | hsa_3351 | D00726 |
| 1 | hsa_3351 | D01973 |
| 1 | hsa_3351 | D02826 |
| 1 | hsa_3351 | D05740 |
| 1 | hsa_3352 | D00283 |
| 1 | hsa_3352 | D00415 |
| 1 | hsa_3352 | D00451 |
| 1 | hsa_3352 | D00513 |
| 1 | hsa_3352 | D00675 |
| 1 | hsa_3352 | D00676 |
| 1 | hsa_3352 | D00726 |
| 1 | hsa_3352 | D01973 |
| 1 | hsa_3352 | D02826 |
| 1 | hsa_3352 | D05740 |
| 1 | hsa_3354 | D00283 |
| 1 | hsa_3354 | D00451 |
| 1 | hsa_3354 | D00513 |
| 1 | hsa_3354 | D00726 |
| 1 | hsa_3354 | D01973 |
| 1 | hsa_3355 | D00283 |
| 1 | hsa_3355 | D00451 |
| 1 | hsa_3355 | D00513 |
| 1 | hsa_3355 | D00674 |
| 1 | hsa_3355 | D00676 |
| 1 | hsa_3355 | D00726 |
| 1 | hsa_3355 | D01973 |
| 1 | hsa_3356 | D00270 |
| 1 | hsa_3356 | D00283 |
| 1 | hsa_3356 | D00426 |
| 1 | hsa_3356 | D00451 |
| 1 | hsa_3356 | D00454 |
| 1 | hsa_3356 | D00493 |
| 1 | hsa_3356 | D00494 |
| 1 | hsa_3356 | D00513 |
| 1 | hsa_3356 | D00563 |
| 1 | hsa_3356 | D00726 |
| 1 | hsa_3356 | D01051 |
| 1 | hsa_3356 | D01164 |
| 1 | hsa_3356 | D01358 |
| 1 | hsa_3356 | D01713 |
| 1 | hsa_3356 | D02340 |
| 1 | hsa_3356 | D02354 |
| 1 | hsa_3356 | D02357 |
| 1 | hsa_3356 | D02361 |
| 1 | hsa_3356 | D02671 |
| 1 | hsa_3357 | D00283 |
| 1 | hsa_3357 | D00451 |
| 1 | hsa_3357 | D00513 |
| 1 | hsa_3357 | D00726 |
| 1 | hsa_3357 | D01164 |
| 1 | hsa_3357 | D01973 |
| 1 | hsa_3358 | D00283 |
| 1 | hsa_3358 | D00451 |
| 1 | hsa_3358 | D00454 |
| 1 | hsa_3358 | D00513 |
| 1 | hsa_3358 | D00563 |
| 1 | hsa_3358 | D00726 |
| 1 | hsa_3358 | D01164 |
| 1 | hsa_3358 | D02354 |
| 1 | hsa_3358 | D02361 |
| 1 | hsa_3358 | D02578 |
| 1 | hsa_3360 | D00274 |
| 1 | hsa_3360 | D00283 |
| 1 | hsa_3360 | D00451 |
| 1 | hsa_3360 | D00513 |
| 1 | hsa_3360 | D00726 |
| 1 | hsa_3360 | D01994 |
| 1 | hsa_3360 | D06056 |
| 1 | hsa_3360 | D06396 |
| 1 | hsa_3361 | D00283 |
| 1 | hsa_3361 | D00451 |
| 1 | hsa_3361 | D00513 |
| 1 | hsa_3361 | D00726 |
| 1 | hsa_3362 | D00283 |
| 1 | hsa_3362 | D00451 |
| 1 | hsa_3362 | D00454 |
| 1 | hsa_3362 | D00513 |
| 1 | hsa_3362 | D00726 |
| 1 | hsa_3363 | D00283 |
| 1 | hsa_3363 | D01051 |
| 1 | hsa_3363 | D01973 |
| 1 | hsa_338442 | D00049 |
| 1 | hsa_3577 | D00139 |
| 1 | hsa_3577 | D00225 |
| 1 | hsa_3577 | D00380 |
| 1 | hsa_3577 | D00394 |
| 1 | hsa_3577 | D00410 |
| 1 | hsa_3577 | D00437 |
| 1 | hsa_3577 | D00528 |
| 1 | hsa_3577 | D00542 |
| 1 | hsa_3577 | D00574 |
| 1 | hsa_3577 | D01071 |
| 1 | hsa_4543 | D02578 |
| 1 | hsa_4985 | D00560 |
| 1 | hsa_4986 | D00110 |
| 1 | hsa_4986 | D00837 |
| 1 | hsa_4986 | D00838 |
| 1 | hsa_4988 | D00301 |
| 1 | hsa_4988 | D00498 |
| 1 | hsa_4988 | D00837 |
| 1 | hsa_4988 | D00838 |
| 1 | hsa_4988 | D00845 |
| 1 | hsa_4988 | D04716 |
| 1 | hsa_4988 | D05113 |
| 1 | hsa_4988 | D05938 |
| 1 | hsa_5028 | D00528 |
| 1 | hsa_5029 | D00528 |
| 1 | hsa_5030 | D00528 |
| 1 | hsa_5031 | D00528 |
| 1 | hsa_5032 | D00528 |
| 1 | hsa_552 | D01236 |
| 1 | hsa_554 | D01236 |
| 1 | hsa_56413 | D00411 |
| 1 | hsa_57105 | D00411 |
| 1 | hsa_5724 | D01652 |
| 1 | hsa_5731 | D00180 |
| 1 | hsa_5731 | D00682 |
| 1 | hsa_5731 | D02721 |
| 1 | hsa_5731 | D02725 |
| 1 | hsa_5732 | D00180 |
| 1 | hsa_5732 | D00419 |
| 1 | hsa_5732 | D03187 |
| 1 | hsa_5733 | D00419 |
| 1 | hsa_5733 | D01891 |
| 1 | hsa_5733 | D02725 |
| 1 | hsa_5737 | D00356 |
| 1 | hsa_5737 | D01352 |
| 1 | hsa_5737 | D01964 |
| 1 | hsa_5739 | D00079 |
| 1 | hsa_5739 | D00180 |
| 1 | hsa_5739 | D01352 |
| 1 | hsa_5739 | D02721 |
| 1 | hsa_59340 | D00234 |
| 1 | hsa_59340 | D00283 |
| 1 | hsa_59340 | D00300 |
| 1 | hsa_59340 | D00318 |
| 1 | hsa_59340 | D00494 |
| 1 | hsa_59340 | D02566 |
| 1 | hsa_6010 | D05341 |
| 1 | hsa_64805 | D00106 |
| 1 | hsa_64805 | D00528 |
| 1 | hsa_64805 | D00769 |
| 1 | hsa_6751 | D00442 |
| 1 | hsa_6752 | D00442 |
| 1 | hsa_6752 | D02250 |
| 1 | hsa_6753 | D02250 |
| 1 | hsa_6755 | D02250 |
| 1 | hsa_6915 | D00336 |
| 1 | hsa_6915 | D03642 |
| 1 | hsa_7201 | D01925 |
| 1 | hsa_7201 | D02007 |
| 1 | hsa_7201 | D02588 |
| 1 | hsa_8843 | D00499 |
| 1 | hsa_8843 | D00524 |
| 1 | hsa_8843 | D00726 |
| 1 | hsa_9052 | D00094 |
| 1 | hsa_9283 | D01441 |
| 1 | hsa_9934 | D00528 |
| 2 | hsa_10161 | D00235 |
| 2 | hsa_10161 | D00498 |
| 2 | hsa_10161 | D00540 |
| 2 | hsa_10161 | D00559 |
| 2 | hsa_10161 | D00632 |
| 2 | hsa_10161 | D00645 |
| 2 | hsa_10161 | D00666 |
| 2 | hsa_10161 | D00765 |
| 2 | hsa_10161 | D01269 |
| 2 | hsa_10161 | D01324 |
| 2 | hsa_10161 | D03274 |
| 2 | hsa_10800 | D00079 |
| 2 | hsa_10800 | D00106 |
| 2 | hsa_10800 | D00113 |
| 2 | hsa_10800 | D00332 |
| 2 | hsa_10800 | D00380 |
| 2 | hsa_10800 | D00400 |
| 2 | hsa_10800 | D00419 |
| 2 | hsa_10800 | D00480 |
| 2 | hsa_10800 | D00494 |
| 2 | hsa_10800 | D00523 |
| 2 | hsa_10800 | D00673 |
| 2 | hsa_10800 | D01071 |
| 2 | hsa_10800 | D01699 |
| 2 | hsa_10800 | D01712 |
| 2 | hsa_10800 | D02066 |
| 2 | hsa_10800 | D02150 |
| 2 | hsa_10800 | D02359 |
| 2 | hsa_10800 | D05113 |
| 2 | hsa_11255 | D00079 |
| 2 | hsa_11255 | D00255 |
| 2 | hsa_11255 | D00281 |
| 2 | hsa_11255 | D00336 |
| 2 | hsa_11255 | D00364 |
| 2 | hsa_11255 | D00410 |
| 2 | hsa_11255 | D00465 |
| 2 | hsa_11255 | D00503 |
| 2 | hsa_11255 | D00523 |
| 2 | hsa_11255 | D00528 |
| 2 | hsa_11255 | D00682 |
| 2 | hsa_11255 | D00838 |
| 2 | hsa_11255 | D00954 |
| 2 | hsa_11255 | D01269 |
| 2 | hsa_11255 | D01441 |
| 2 | hsa_11255 | D01603 |
| 2 | hsa_11255 | D01994 |
| 2 | hsa_11255 | D03210 |
| 2 | hsa_11255 | D03879 |
| 2 | hsa_11255 | D03880 |
| 2 | hsa_11255 | D06396 |
| 2 | hsa_1128 | D00234 |
| 2 | hsa_1128 | D00255 |
| 2 | hsa_1128 | D00318 |
| 2 | hsa_1128 | D00332 |
| 2 | hsa_1128 | D00371 |
| 2 | hsa_1128 | D00411 |
| 2 | hsa_1128 | D00422 |
| 2 | hsa_1128 | D00509 |
| 2 | hsa_1128 | D01051 |
| 2 | hsa_1128 | D01242 |
| 2 | hsa_1128 | D01346 |
| 2 | hsa_1128 | D01454 |
| 2 | hsa_1128 | D01603 |
| 2 | hsa_1128 | D01712 |
| 2 | hsa_1128 | D02342 |
| 2 | hsa_1128 | D03165 |
| 2 | hsa_1128 | D03880 |
| 2 | hsa_1128 | D05792 |
| 2 | hsa_1128 | D05938 |
| 2 | hsa_1129 | D00079 |
| 2 | hsa_1129 | D00300 |
| 2 | hsa_1129 | D00301 |
| 2 | hsa_1129 | D00306 |
| 2 | hsa_1129 | D00498 |
| 2 | hsa_1129 | D00520 |
| 2 | hsa_1129 | D00560 |
| 2 | hsa_1129 | D00673 |
| 2 | hsa_1129 | D00996 |
| 2 | hsa_1129 | D01051 |
| 2 | hsa_1129 | D01118 |
| 2 | hsa_1129 | D01745 |
| 2 | hsa_1129 | D02007 |
| 2 | hsa_1129 | D02149 |
| 2 | hsa_1129 | D02566 |
| 2 | hsa_1129 | D02910 |
| 2 | hsa_1129 | D03880 |
| 2 | hsa_1131 | D00059 |
| 2 | hsa_1131 | D00095 |
| 2 | hsa_1131 | D00270 |
| 2 | hsa_1131 | D00465 |
| 2 | hsa_1131 | D00493 |
| 2 | hsa_1131 | D00574 |
| 2 | hsa_1131 | D00646 |
| 2 | hsa_1131 | D00987 |
| 2 | hsa_1131 | D00996 |
| 2 | hsa_1131 | D01022 |
| 2 | hsa_1131 | D02082 |
| 2 | hsa_1131 | D02338 |
| 2 | hsa_1131 | D02358 |
| 2 | hsa_1131 | D02588 |
| 2 | hsa_1131 | D03210 |
| 2 | hsa_1131 | D03490 |
| 2 | hsa_1132 | D00095 |
| 2 | hsa_1132 | D00136 |
| 2 | hsa_1132 | D00270 |
| 2 | hsa_1132 | D00318 |
| 2 | hsa_1132 | D00400 |
| 2 | hsa_1132 | D00480 |
| 2 | hsa_1132 | D00523 |
| 2 | hsa_1132 | D00559 |
| 2 | hsa_1132 | D00563 |
| 2 | hsa_1132 | D00598 |
| 2 | hsa_1132 | D00609 |
| 2 | hsa_1132 | D00673 |
| 2 | hsa_1132 | D01352 |
| 2 | hsa_1132 | D01994 |
| 2 | hsa_1132 | D02237 |
| 2 | hsa_1132 | D02342 |
| 2 | hsa_1132 | D02884 |
| 2 | hsa_1132 | D03880 |
| 2 | hsa_1132 | D05113 |
| 2 | hsa_1132 | D05740 |
| 2 | hsa_1133 | D00232 |
| 2 | hsa_1133 | D00380 |
| 2 | hsa_1133 | D00410 |
| 2 | hsa_1133 | D00432 |
| 2 | hsa_1133 | D00483 |
| 2 | hsa_1133 | D00521 |
| 2 | hsa_1133 | D00542 |
| 2 | hsa_1133 | D00666 |
| 2 | hsa_1133 | D00765 |
| 2 | hsa_1133 | D00845 |
| 2 | hsa_1133 | D01118 |
| 2 | hsa_1133 | D02066 |
| 2 | hsa_1133 | D02149 |
| 2 | hsa_1133 | D02327 |
| 2 | hsa_1133 | D02374 |
| 2 | hsa_1133 | D02578 |
| 2 | hsa_1133 | D03274 |
| 2 | hsa_1133 | D03621 |
| 2 | hsa_1133 | D05246 |
| 2 | hsa_1234 | D00426 |
| 2 | hsa_1234 | D01020 |
| 2 | hsa_1234 | D02007 |
| 2 | hsa_1234 | D02234 |
| 2 | hsa_1234 | D02278 |
| 2 | hsa_1234 | D04375 |
| 2 | hsa_1234 | D04625 |
| 2 | hsa_1234 | D05938 |
| 2 | hsa_1241 | D00281 |
| 2 | hsa_1241 | D00480 |
| 2 | hsa_1241 | D00779 |
| 2 | hsa_1241 | D01386 |
| 2 | hsa_1241 | D01462 |
| 2 | hsa_1241 | D02359 |
| 2 | hsa_1241 | D02910 |
| 2 | hsa_1241 | D03490 |
| 2 | hsa_1241 | D03858 |
| 2 | hsa_1268 | D00180 |
| 2 | hsa_1268 | D00283 |
| 2 | hsa_1268 | D00790 |
| 2 | hsa_1268 | D01118 |
| 2 | hsa_1268 | D01462 |
| 2 | hsa_1268 | D01713 |
| 2 | hsa_1268 | D01965 |
| 2 | hsa_1268 | D02150 |
| 2 | hsa_1268 | D02234 |
| 2 | hsa_1268 | D02578 |
| 2 | hsa_1268 | D03880 |
| 2 | hsa_1268 | D04040 |
| 2 | hsa_1268 | D06056 |
| 2 | hsa_134 | D00225 |
| 2 | hsa_134 | D00281 |
| 2 | hsa_134 | D00380 |
| 2 | hsa_134 | D00627 |
| 2 | hsa_134 | D00632 |
| 2 | hsa_134 | D00769 |
| 2 | hsa_134 | D01020 |
| 2 | hsa_134 | D01227 |
| 2 | hsa_134 | D01297 |
| 2 | hsa_134 | D01332 |
| 2 | hsa_134 | D02349 |
| 2 | hsa_134 | D02725 |
| 2 | hsa_135 | D00225 |
| 2 | hsa_135 | D00432 |
| 2 | hsa_135 | D00673 |
| 2 | hsa_135 | D00779 |
| 2 | hsa_135 | D00954 |
| 2 | hsa_135 | D00996 |
| 2 | hsa_135 | D02007 |
| 2 | hsa_135 | D03880 |
| 2 | hsa_136 | D00356 |
| 2 | hsa_136 | D00498 |
| 2 | hsa_136 | D00524 |
| 2 | hsa_136 | D00769 |
| 2 | hsa_136 | D00845 |
| 2 | hsa_136 | D00996 |
| 2 | hsa_136 | D01164 |
| 2 | hsa_136 | D01352 |
| 2 | hsa_136 | D01652 |
| 2 | hsa_136 | D01891 |
| 2 | hsa_136 | D02361 |
| 2 | hsa_136 | D03858 |
| 2 | hsa_140 | D00380 |
| 2 | hsa_140 | D00437 |
| 2 | hsa_140 | D00442 |
| 2 | hsa_140 | D00540 |
| 2 | hsa_140 | D00646 |
| 2 | hsa_140 | D00684 |
| 2 | hsa_140 | D00838 |
| 2 | hsa_140 | D00965 |
| 2 | hsa_140 | D01390 |
| 2 | hsa_140 | D01454 |
| 2 | hsa_140 | D01964 |
| 2 | hsa_140 | D02070 |
| 2 | hsa_140 | D02884 |
| 2 | hsa_140 | D03210 |
| 2 | hsa_146 | D00332 |
| 2 | hsa_146 | D00674 |
| 2 | hsa_146 | D02066 |
| 2 | hsa_146 | D02076 |
| 2 | hsa_146 | D02349 |
| 2 | hsa_146 | D03880 |
| 2 | hsa_146 | D04040 |
| 2 | hsa_147 | D00136 |
| 2 | hsa_147 | D00270 |
| 2 | hsa_147 | D00394 |
| 2 | hsa_147 | D00443 |
| 2 | hsa_147 | D00574 |
| 2 | hsa_147 | D00604 |
| 2 | hsa_147 | D00632 |
| 2 | hsa_147 | D00987 |
| 2 | hsa_147 | D01297 |
| 2 | hsa_147 | D01352 |
| 2 | hsa_147 | D02150 |
| 2 | hsa_147 | D05246 |
| 2 | hsa_148 | D00079 |
| 2 | hsa_148 | D00180 |
| 2 | hsa_148 | D00234 |
| 2 | hsa_148 | D00300 |
| 2 | hsa_148 | D00301 |
| 2 | hsa_148 | D00715 |
| 2 | hsa_148 | D00780 |
| 2 | hsa_148 | D01269 |
| 2 | hsa_148 | D02082 |
| 2 | hsa_148 | D02349 |
| 2 | hsa_148 | D02354 |
| 2 | hsa_148 | D03490 |
| 2 | hsa_148 | D04716 |
| 2 | hsa_148 | D05246 |
| 2 | hsa_150 | D00295 |
| 2 | hsa_150 | D00318 |
| 2 | hsa_150 | D00364 |
| 2 | hsa_150 | D00574 |
| 2 | hsa_150 | D00775 |
| 2 | hsa_150 | D01164 |
| 2 | hsa_150 | D01692 |
| 2 | hsa_150 | D01994 |
| 2 | hsa_150 | D06396 |
| 2 | hsa_151 | D00400 |
| 2 | hsa_151 | D00451 |
| 2 | hsa_151 | D00520 |
| 2 | hsa_151 | D00574 |
| 2 | hsa_151 | D00645 |
| 2 | hsa_151 | D00765 |
| 2 | hsa_151 | D01699 |
| 2 | hsa_151 | D01973 |
| 2 | hsa_151 | D02250 |
| 2 | hsa_151 | D02278 |
| 2 | hsa_151 | D04716 |
| 2 | hsa_151 | D05341 |
| 2 | hsa_152 | D00336 |
| 2 | hsa_152 | D00371 |
| 2 | hsa_152 | D00451 |
| 2 | hsa_152 | D00514 |
| 2 | hsa_152 | D00645 |
| 2 | hsa_152 | D00837 |
| 2 | hsa_152 | D01712 |
| 2 | hsa_152 | D02007 |
| 2 | hsa_152 | D02278 |
| 2 | hsa_152 | D02279 |
| 2 | hsa_152 | D02826 |
| 2 | hsa_152 | D03503 |
| 2 | hsa_152 | D03881 |
| 2 | hsa_153 | D00059 |
| 2 | hsa_153 | D00364 |
| 2 | hsa_153 | D00394 |
| 2 | hsa_153 | D00765 |
| 2 | hsa_153 | D00954 |
| 2 | hsa_153 | D02361 |
| 2 | hsa_153 | D03187 |
| 2 | hsa_153 | D04040 |
| 2 | hsa_154 | D00281 |
| 2 | hsa_154 | D00306 |
| 2 | hsa_154 | D00682 |
| 2 | hsa_154 | D01022 |
| 2 | hsa_154 | D01071 |
| 2 | hsa_154 | D02358 |
| 2 | hsa_154 | D02725 |
| 2 | hsa_155 | D00235 |
| 2 | hsa_155 | D00300 |
| 2 | hsa_155 | D00336 |
| 2 | hsa_155 | D00397 |
| 2 | hsa_155 | D00410 |
| 2 | hsa_155 | D01242 |
| 2 | hsa_155 | D02007 |
| 2 | hsa_155 | D02237 |
| 2 | hsa_155 | D05246 |
| 2 | hsa_155 | D06396 |
| 2 | hsa_1812 | D00234 |
| 2 | hsa_1812 | D00241 |
| 2 | hsa_1812 | D00336 |
| 2 | hsa_1812 | D00410 |
| 2 | hsa_1812 | D00432 |
| 2 | hsa_1812 | D00451 |
| 2 | hsa_1812 | D00542 |
| 2 | hsa_1812 | D00607 |
| 2 | hsa_1812 | D00996 |
| 2 | hsa_1812 | D01071 |
| 2 | hsa_1812 | D01352 |
| 2 | hsa_1812 | D01652 |
| 2 | hsa_1812 | D01828 |
| 2 | hsa_1812 | D02721 |
| 2 | hsa_1812 | D04040 |
| 2 | hsa_1812 | D06396 |
| 2 | hsa_1813 | D00094 |
| 2 | hsa_1813 | D00180 |
| 2 | hsa_1813 | D00442 |
| 2 | hsa_1813 | D00498 |
| 2 | hsa_1813 | D00522 |
| 2 | hsa_1813 | D01346 |
| 2 | hsa_1813 | D01358 |
| 2 | hsa_1813 | D02342 |
| 2 | hsa_1813 | D02357 |
| 2 | hsa_1813 | D02725 |
| 2 | hsa_1813 | D03858 |
| 2 | hsa_1813 | D04716 |
| 2 | hsa_1813 | D05113 |
| 2 | hsa_1813 | D05792 |
| 2 | hsa_1814 | D00113 |
| 2 | hsa_1814 | D00332 |
| 2 | hsa_1814 | D00356 |
| 2 | hsa_1814 | D00451 |
| 2 | hsa_1814 | D00523 |
| 2 | hsa_1814 | D00688 |
| 2 | hsa_1814 | D01713 |
| 2 | hsa_1814 | D01782 |
| 2 | hsa_1814 | D02342 |
| 2 | hsa_1814 | D03415 |
| 2 | hsa_1814 | D03642 |
| 2 | hsa_1814 | D05740 |
| 2 | hsa_1814 | D05938 |
| 2 | hsa_1815 | D00180 |
| 2 | hsa_1815 | D00300 |
| 2 | hsa_1815 | D00371 |
| 2 | hsa_1815 | D00465 |
| 2 | hsa_1815 | D00520 |
| 2 | hsa_1815 | D00574 |
| 2 | hsa_1815 | D00775 |
| 2 | hsa_1815 | D01358 |
| 2 | hsa_1815 | D01994 |
| 2 | hsa_1815 | D03187 |
| 2 | hsa_1815 | D03621 |
| 2 | hsa_1815 | D05341 |
| 2 | hsa_1816 | D00234 |
| 2 | hsa_1816 | D00422 |
| 2 | hsa_1816 | D00432 |
| 2 | hsa_1816 | D00480 |
| 2 | hsa_1816 | D00494 |
| 2 | hsa_1816 | D00574 |
| 2 | hsa_1816 | D00632 |
| 2 | hsa_1816 | D00676 |
| 2 | hsa_1816 | D01051 |
| 2 | hsa_1816 | D01891 |
| 2 | hsa_1816 | D01965 |
| 2 | hsa_1816 | D02349 |
| 2 | hsa_1816 | D02356 |
| 2 | hsa_1816 | D03274 |
| 2 | hsa_1816 | D03490 |
| 2 | hsa_1816 | D05740 |
| 2 | hsa_185 | D00255 |
| 2 | hsa_185 | D00380 |
| 2 | hsa_185 | D00480 |
| 2 | hsa_185 | D00493 |
| 2 | hsa_185 | D00521 |
| 2 | hsa_185 | D00645 |
| 2 | hsa_185 | D00682 |
| 2 | hsa_185 | D00775 |
| 2 | hsa_185 | D00780 |
| 2 | hsa_185 | D00790 |
| 2 | hsa_185 | D01236 |
| 2 | hsa_185 | D01297 |
| 2 | hsa_185 | D01652 |
| 2 | hsa_185 | D01871 |
| 2 | hsa_185 | D02358 |
| 2 | hsa_185 | D02826 |
| 2 | hsa_1909 | D00113 |
| 2 | hsa_1909 | D00274 |
| 2 | hsa_1909 | D00432 |
| 2 | hsa_1909 | D00451 |
| 2 | hsa_1909 | D00542 |
| 2 | hsa_1909 | D00666 |
| 2 | hsa_1909 | D01346 |
| 2 | hsa_1909 | D01441 |
| 2 | hsa_1909 | D01717 |
| 2 | hsa_1909 | D01782 |
| 2 | hsa_1909 | D02354 |
| 2 | hsa_1909 | D02566 |
| 2 | hsa_1909 | D03642 |
| 2 | hsa_1909 | D03654 |
| 2 | hsa_1909 | D04006 |
| 2 | hsa_1909 | D04375 |
| 2 | hsa_1909 | D05740 |
| 2 | hsa_1910 | D00274 |
| 2 | hsa_1910 | D00295 |
| 2 | hsa_1910 | D00364 |
| 2 | hsa_1910 | D00422 |
| 2 | hsa_1910 | D01164 |
| 2 | hsa_1910 | D02237 |
| 2 | hsa_1910 | D05341 |
| 2 | hsa_222545 | D00079 |
| 2 | hsa_222545 | D00139 |
| 2 | hsa_222545 | D00295 |
| 2 | hsa_222545 | D00493 |
| 2 | hsa_222545 | D00521 |
| 2 | hsa_222545 | D00674 |
| 2 | hsa_222545 | D00675 |
| 2 | hsa_222545 | D00765 |
| 2 | hsa_222545 | D00775 |
| 2 | hsa_222545 | D00845 |
| 2 | hsa_222545 | D01103 |
| 2 | hsa_222545 | D01236 |
| 2 | hsa_222545 | D01242 |
| 2 | hsa_222545 | D01454 |
| 2 | hsa_222545 | D02150 |
| 2 | hsa_222545 | D02359 |
| 2 | hsa_222545 | D05938 |
| 2 | hsa_23620 | D00234 |
| 2 | hsa_23620 | D00235 |
| 2 | hsa_23620 | D00559 |
| 2 | hsa_23620 | D00604 |
| 2 | hsa_23620 | D00683 |
| 2 | hsa_23620 | D00837 |
| 2 | hsa_23620 | D01297 |
| 2 | hsa_23620 | D01603 |
| 2 | hsa_23620 | D01692 |
| 2 | hsa_23620 | D01994 |
| 2 | hsa_23620 | D02250 |
| 2 | hsa_23620 | D02566 |
| 2 | hsa_23620 | D03490 |
| 2 | hsa_23620 | D04625 |
| 2 | hsa_23620 | D06056 |
| 2 | hsa_2550 | D00283 |
| 2 | hsa_2550 | D00394 |
| 2 | hsa_2550 | D00632 |
| 2 | hsa_2550 | D00760 |
| 2 | hsa_2550 | D01024 |
| 2 | hsa_2550 | D01699 |
| 2 | hsa_2550 | D02361 |
| 2 | hsa_2550 | D02566 |
| 2 | hsa_2550 | D03621 |
| 2 | hsa_2846 | D00139 |
| 2 | hsa_2846 | D00234 |
| 2 | hsa_2846 | D00270 |
| 2 | hsa_2846 | D00274 |
| 2 | hsa_2846 | D00442 |
| 2 | hsa_2846 | D00480 |
| 2 | hsa_2846 | D00525 |
| 2 | hsa_2846 | D00673 |
| 2 | hsa_2846 | D00845 |
| 2 | hsa_2846 | D00996 |
| 2 | hsa_2846 | D01022 |
| 2 | hsa_2846 | D01891 |
| 2 | hsa_2846 | D02349 |
| 2 | hsa_2846 | D02721 |
| 2 | hsa_2911 | D00301 |
| 2 | hsa_2911 | D00318 |
| 2 | hsa_2911 | D00371 |
| 2 | hsa_2911 | D00480 |
| 2 | hsa_2911 | D00483 |
| 2 | hsa_2911 | D00514 |
| 2 | hsa_2911 | D00542 |
| 2 | hsa_2911 | D00676 |
| 2 | hsa_2911 | D00996 |
| 2 | hsa_2911 | D01297 |
| 2 | hsa_2911 | D01462 |
| 2 | hsa_2911 | D02338 |
| 2 | hsa_2912 | D00480 |
| 2 | hsa_2912 | D00528 |
| 2 | hsa_2912 | D00540 |
| 2 | hsa_2912 | D00627 |
| 2 | hsa_2912 | D00665 |
| 2 | hsa_2912 | D01692 |
| 2 | hsa_2912 | D01699 |
| 2 | hsa_2912 | D02150 |
| 2 | hsa_2912 | D04034 |
| 2 | hsa_2912 | D04716 |
| 2 | hsa_2913 | D00110 |
| 2 | hsa_2913 | D00113 |
| 2 | hsa_2913 | D00380 |
| 2 | hsa_2913 | D00514 |
| 2 | hsa_2913 | D01699 |
| 2 | hsa_2913 | D02374 |
| 2 | hsa_2913 | D02566 |
| 2 | hsa_2913 | D03879 |
| 2 | hsa_2914 | D00180 |
| 2 | hsa_2914 | D00498 |
| 2 | hsa_2914 | D00604 |
| 2 | hsa_2914 | D00682 |
| 2 | hsa_2914 | D01352 |
| 2 | hsa_2914 | D01386 |
| 2 | hsa_2914 | D01712 |
| 2 | hsa_2914 | D01713 |
| 2 | hsa_2914 | D01717 |
| 2 | hsa_2914 | D01973 |
| 2 | hsa_2914 | D02066 |
| 2 | hsa_2914 | D02250 |
| 2 | hsa_2914 | D02340 |
| 2 | hsa_2914 | D02358 |
| 2 | hsa_2914 | D02614 |
| 2 | hsa_2914 | D03654 |
| 2 | hsa_2914 | D03880 |
| 2 | hsa_2915 | D00301 |
| 2 | hsa_2915 | D00443 |
| 2 | hsa_2915 | D00574 |
| 2 | hsa_2915 | D00613 |
| 2 | hsa_2915 | D00726 |
| 2 | hsa_2915 | D01164 |
| 2 | hsa_2915 | D01390 |
| 2 | hsa_2915 | D01603 |
| 2 | hsa_2915 | D01717 |
| 2 | hsa_2915 | D02278 |
| 2 | hsa_2915 | D02349 |
| 2 | hsa_2915 | D03274 |
| 2 | hsa_2916 | D00049 |
| 2 | hsa_2916 | D00095 |
| 2 | hsa_2916 | D00113 |
| 2 | hsa_2916 | D00306 |
| 2 | hsa_2916 | D00503 |
| 2 | hsa_2916 | D00598 |
| 2 | hsa_2916 | D00779 |
| 2 | hsa_2916 | D01324 |
| 2 | hsa_2916 | D01699 |
| 2 | hsa_2916 | D01925 |
| 2 | hsa_2916 | D02150 |
| 2 | hsa_2916 | D02354 |
| 2 | hsa_2916 | D02356 |
| 2 | hsa_2916 | D02361 |
| 2 | hsa_2916 | D02374 |
| 2 | hsa_2916 | D06396 |
| 2 | hsa_2917 | D00180 |
| 2 | hsa_2917 | D00235 |
| 2 | hsa_2917 | D00300 |
| 2 | hsa_2917 | D00332 |
| 2 | hsa_2917 | D00422 |
| 2 | hsa_2917 | D00542 |
| 2 | hsa_2917 | D00779 |
| 2 | hsa_2917 | D01051 |
| 2 | hsa_2917 | D01652 |
| 2 | hsa_2917 | D01717 |
| 2 | hsa_2917 | D01745 |
| 2 | hsa_2917 | D02066 |
| 2 | hsa_2917 | D02342 |
| 2 | hsa_2917 | D02361 |
| 2 | hsa_2917 | D02566 |
| 2 | hsa_2917 | D02884 |
| 2 | hsa_2917 | D03858 |
| 2 | hsa_2918 | D00235 |
| 2 | hsa_2918 | D00241 |
| 2 | hsa_2918 | D00306 |
| 2 | hsa_2918 | D00318 |
| 2 | hsa_2918 | D00454 |
| 2 | hsa_2918 | D00522 |
| 2 | hsa_2918 | D00574 |
| 2 | hsa_2918 | D00688 |
| 2 | hsa_2918 | D01925 |
| 2 | hsa_2918 | D02149 |
| 2 | hsa_2918 | D02349 |
| 2 | hsa_2918 | D03858 |
| 2 | hsa_2918 | D04006 |
| 2 | hsa_2918 | D04034 |
| 2 | hsa_2918 | D06056 |
| 2 | hsa_3269 | D00110 |
| 2 | hsa_3269 | D00225 |
| 2 | hsa_3269 | D00270 |
| 2 | hsa_3269 | D00503 |
| 2 | hsa_3269 | D00514 |
| 2 | hsa_3269 | D00525 |
| 2 | hsa_3269 | D00607 |
| 2 | hsa_3269 | D00635 |
| 2 | hsa_3269 | D00996 |
| 2 | hsa_3269 | D02007 |
| 2 | hsa_3269 | D02234 |
| 2 | hsa_3269 | D02250 |
| 2 | hsa_3269 | D02340 |
| 2 | hsa_3269 | D02578 |
| 2 | hsa_3269 | D06396 |
| 2 | hsa_3274 | D00095 |
| 2 | hsa_3274 | D00540 |
| 2 | hsa_3274 | D00609 |
| 2 | hsa_3274 | D00674 |
| 2 | hsa_3274 | D00775 |
| 2 | hsa_3274 | D01227 |
| 2 | hsa_3274 | D02237 |
| 2 | hsa_3274 | D02250 |
| 2 | hsa_3274 | D02278 |
| 2 | hsa_3274 | D05740 |
| 2 | hsa_3350 | D00394 |
| 2 | hsa_3350 | D00432 |
| 2 | hsa_3350 | D00465 |
| 2 | hsa_3350 | D00574 |
| 2 | hsa_3350 | D00665 |
| 2 | hsa_3350 | D00674 |
| 2 | hsa_3350 | D00675 |
| 2 | hsa_3350 | D00676 |
| 2 | hsa_3350 | D01024 |
| 2 | hsa_3350 | D02082 |
| 2 | hsa_3350 | D02150 |
| 2 | hsa_3350 | D02278 |
| 2 | hsa_3351 | D00079 |
| 2 | hsa_3351 | D00394 |
| 2 | hsa_3351 | D00509 |
| 2 | hsa_3351 | D00523 |
| 2 | hsa_3351 | D00559 |
| 2 | hsa_3351 | D00838 |
| 2 | hsa_3351 | D01925 |
| 2 | hsa_3351 | D02082 |
| 2 | hsa_3351 | D02234 |
| 2 | hsa_3351 | D04625 |
| 2 | hsa_3352 | D00059 |
| 2 | hsa_3352 | D00394 |
| 2 | hsa_3352 | D00426 |
| 2 | hsa_3352 | D00432 |
| 2 | hsa_3352 | D00498 |
| 2 | hsa_3352 | D00521 |
| 2 | hsa_3352 | D00574 |
| 2 | hsa_3352 | D00613 |
| 2 | hsa_3352 | D00682 |
| 2 | hsa_3352 | D01713 |
| 2 | hsa_3352 | D01828 |
| 2 | hsa_3352 | D02340 |
| 2 | hsa_3352 | D03490 |
| 2 | hsa_3354 | D00281 |
| 2 | hsa_3354 | D00397 |
| 2 | hsa_3354 | D00411 |
| 2 | hsa_3354 | D00422 |
| 2 | hsa_3354 | D00665 |
| 2 | hsa_3354 | D00954 |
| 2 | hsa_3354 | D00996 |
| 2 | hsa_3354 | D01020 |
| 2 | hsa_3354 | D01024 |
| 2 | hsa_3354 | D01390 |
| 2 | hsa_3354 | D01652 |
| 2 | hsa_3354 | D01745 |
| 2 | hsa_3354 | D01828 |
| 2 | hsa_3354 | D02671 |
| 2 | hsa_3354 | D04034 |
| 2 | hsa_3355 | D00234 |
| 2 | hsa_3355 | D00235 |
| 2 | hsa_3355 | D00301 |
| 2 | hsa_3355 | D00318 |
| 2 | hsa_3355 | D00397 |
| 2 | hsa_3355 | D00465 |
| 2 | hsa_3355 | D00523 |
| 2 | hsa_3355 | D00560 |
| 2 | hsa_3355 | D00996 |
| 2 | hsa_3355 | D01118 |
| 2 | hsa_3355 | D03621 |
| 2 | hsa_3355 | D03881 |
| 2 | hsa_3356 | D00227 |
| 2 | hsa_3356 | D00232 |
| 2 | hsa_3356 | D00371 |
| 2 | hsa_3356 | D00688 |
| 2 | hsa_3356 | D01126 |
| 2 | hsa_3356 | D01324 |
| 2 | hsa_3356 | D01386 |
| 2 | hsa_3356 | D01441 |
| 2 | hsa_3356 | D02150 |
| 2 | hsa_3356 | D02250 |
| 2 | hsa_3356 | D02342 |
| 2 | hsa_3356 | D02614 |
| 2 | hsa_3356 | D02826 |
| 2 | hsa_3356 | D03858 |
| 2 | hsa_3356 | D03879 |
| 2 | hsa_3356 | D03880 |
| 2 | hsa_3357 | D00227 |
| 2 | hsa_3357 | D00394 |
| 2 | hsa_3357 | D00493 |
| 2 | hsa_3357 | D00514 |
| 2 | hsa_3357 | D00563 |
| 2 | hsa_3357 | D00665 |
| 2 | hsa_3357 | D01227 |
| 2 | hsa_3357 | D01324 |
| 2 | hsa_3357 | D01713 |
| 2 | hsa_3357 | D01891 |
| 2 | hsa_3357 | D02066 |
| 2 | hsa_3357 | D02082 |
| 2 | hsa_3357 | D02358 |
| 2 | hsa_3357 | D02910 |
| 2 | hsa_3357 | D03415 |
| 2 | hsa_3358 | D00106 |
| 2 | hsa_3358 | D00235 |
| 2 | hsa_3358 | D00443 |
| 2 | hsa_3358 | D00676 |
| 2 | hsa_3358 | D00688 |
| 2 | hsa_3358 | D00769 |
| 2 | hsa_3358 | D01352 |
| 2 | hsa_3358 | D01925 |
| 2 | hsa_3358 | D02007 |
| 2 | hsa_3358 | D02070 |
| 2 | hsa_3358 | D02671 |
| 2 | hsa_3358 | D03621 |
| 2 | hsa_3358 | D04040 |
| 2 | hsa_3358 | D04716 |
| 2 | hsa_3360 | D00270 |
| 2 | hsa_3360 | D00295 |
| 2 | hsa_3360 | D00432 |
| 2 | hsa_3360 | D00521 |
| 2 | hsa_3360 | D00540 |
| 2 | hsa_3360 | D00687 |
| 2 | hsa_3360 | D00954 |
| 2 | hsa_3360 | D01051 |
| 2 | hsa_3360 | D01236 |
| 2 | hsa_3360 | D01352 |
| 2 | hsa_3360 | D01441 |
| 2 | hsa_3360 | D01965 |
| 2 | hsa_3360 | D02070 |
| 2 | hsa_3360 | D02338 |
| 2 | hsa_3360 | D03621 |
| 2 | hsa_3360 | D03642 |
| 2 | hsa_3360 | D05938 |
| 2 | hsa_3361 | D00306 |
| 2 | hsa_3361 | D00559 |
| 2 | hsa_3361 | D00604 |
| 2 | hsa_3361 | D00627 |
| 2 | hsa_3361 | D00632 |
| 2 | hsa_3361 | D00675 |
| 2 | hsa_3361 | D02007 |
| 2 | hsa_3361 | D02076 |
| 2 | hsa_3361 | D02234 |
| 2 | hsa_3361 | D02237 |
| 2 | hsa_3361 | D02250 |
| 2 | hsa_3361 | D02356 |
| 2 | hsa_3361 | D02614 |
| 2 | hsa_3362 | D00113 |
| 2 | hsa_3362 | D00234 |
| 2 | hsa_3362 | D00295 |
| 2 | hsa_3362 | D00356 |
| 2 | hsa_3362 | D00415 |
| 2 | hsa_3362 | D00432 |
| 2 | hsa_3362 | D00523 |
| 2 | hsa_3362 | D00560 |
| 2 | hsa_3362 | D00613 |
| 2 | hsa_3362 | D00665 |
| 2 | hsa_3362 | D00684 |
| 2 | hsa_3362 | D00775 |
| 2 | hsa_3362 | D00838 |
| 2 | hsa_3362 | D01051 |
| 2 | hsa_3362 | D01441 |
| 2 | hsa_3362 | D01454 |
| 2 | hsa_3362 | D01891 |
| 2 | hsa_3362 | D02342 |
| 2 | hsa_3362 | D03210 |
| 2 | hsa_3362 | D04625 |
| 2 | hsa_3362 | D05740 |
| 2 | hsa_3363 | D00095 |
| 2 | hsa_3363 | D00295 |
| 2 | hsa_3363 | D00442 |
| 2 | hsa_3363 | D00513 |
| 2 | hsa_3363 | D00525 |
| 2 | hsa_3363 | D00540 |
| 2 | hsa_3363 | D00607 |
| 2 | hsa_3363 | D00675 |
| 2 | hsa_3363 | D00682 |
| 2 | hsa_3363 | D01103 |
| 2 | hsa_3363 | D01441 |
| 2 | hsa_3363 | D01699 |
| 2 | hsa_3363 | D01828 |
| 2 | hsa_3363 | D02566 |
| 2 | hsa_3363 | D02826 |
| 2 | hsa_338442 | D00283 |
| 2 | hsa_338442 | D00437 |
| 2 | hsa_338442 | D00606 |
| 2 | hsa_338442 | D02070 |
| 2 | hsa_338442 | D02076 |
| 2 | hsa_338442 | D02278 |
| 2 | hsa_338442 | D02361 |
| 2 | hsa_338442 | D04034 |
| 2 | hsa_338442 | D05246 |
| 2 | hsa_338442 | D05740 |
| 2 | hsa_3577 | D00301 |
| 2 | hsa_3577 | D00419 |
| 2 | hsa_3577 | D00443 |
| 2 | hsa_3577 | D00524 |
| 2 | hsa_3577 | D01022 |
| 2 | hsa_3577 | D01242 |
| 2 | hsa_3577 | D01324 |
| 2 | hsa_3577 | D01891 |
| 2 | hsa_3577 | D02076 |
| 2 | hsa_3577 | D02578 |
| 2 | hsa_3577 | D02721 |
| 2 | hsa_4543 | D00110 |
| 2 | hsa_4543 | D00306 |
| 2 | hsa_4543 | D00380 |
| 2 | hsa_4543 | D00494 |
| 2 | hsa_4543 | D00498 |
| 2 | hsa_4543 | D00609 |
| 2 | hsa_4543 | D00665 |
| 2 | hsa_4543 | D01126 |
| 2 | hsa_4543 | D01164 |
| 2 | hsa_4543 | D01454 |
| 2 | hsa_4543 | D01745 |
| 2 | hsa_4543 | D01782 |
| 2 | hsa_4543 | D01965 |
| 2 | hsa_4543 | D02614 |
| 2 | hsa_4985 | D00110 |
| 2 | hsa_4985 | D00227 |
| 2 | hsa_4985 | D00509 |
| 2 | hsa_4985 | D00606 |
| 2 | hsa_4985 | D00609 |
| 2 | hsa_4985 | D00688 |
| 2 | hsa_4985 | D01994 |
| 2 | hsa_4985 | D02007 |
| 2 | hsa_4985 | D02588 |
| 2 | hsa_4985 | D02614 |
| 2 | hsa_4985 | D03165 |
| 2 | hsa_4985 | D03642 |
| 2 | hsa_4985 | D04006 |
| 2 | hsa_4986 | D00049 |
| 2 | hsa_4986 | D00281 |
| 2 | hsa_4986 | D00415 |
| 2 | hsa_4986 | D00443 |
| 2 | hsa_4986 | D00465 |
| 2 | hsa_4986 | D00528 |
| 2 | hsa_4986 | D00607 |
| 2 | hsa_4986 | D00726 |
| 2 | hsa_4986 | D00790 |
| 2 | hsa_4986 | D01269 |
| 2 | hsa_4986 | D01297 |
| 2 | hsa_4986 | D01692 |
| 2 | hsa_4986 | D01745 |
| 2 | hsa_4986 | D02070 |
| 2 | hsa_4986 | D02147 |
| 2 | hsa_4986 | D04006 |
| 2 | hsa_4988 | D00095 |
| 2 | hsa_4988 | D00110 |
| 2 | hsa_4988 | D00139 |
| 2 | hsa_4988 | D00437 |
| 2 | hsa_4988 | D00465 |
| 2 | hsa_4988 | D00524 |
| 2 | hsa_4988 | D00560 |
| 2 | hsa_4988 | D00675 |
| 2 | hsa_4988 | D00726 |
| 2 | hsa_4988 | D00790 |
| 2 | hsa_4988 | D01295 |
| 2 | hsa_4988 | D01346 |
| 2 | hsa_4988 | D01386 |
| 2 | hsa_4988 | D02278 |
| 2 | hsa_4988 | D02721 |
| 2 | hsa_4988 | D03415 |
| 2 | hsa_4988 | D03621 |
| 2 | hsa_4988 | D04006 |
| 2 | hsa_5028 | D00225 |
| 2 | hsa_5028 | D00270 |
| 2 | hsa_5028 | D00604 |
| 2 | hsa_5028 | D00779 |
| 2 | hsa_5028 | D00987 |
| 2 | hsa_5028 | D02082 |
| 2 | hsa_5028 | D02349 |
| 2 | hsa_5029 | D00059 |
| 2 | hsa_5029 | D00498 |
| 2 | hsa_5029 | D00521 |
| 2 | hsa_5029 | D00688 |
| 2 | hsa_5029 | D00779 |
| 2 | hsa_5029 | D00790 |
| 2 | hsa_5029 | D01164 |
| 2 | hsa_5029 | D01236 |
| 2 | hsa_5029 | D01441 |
| 2 | hsa_5029 | D02149 |
| 2 | hsa_5029 | D02340 |
| 2 | hsa_5029 | D02725 |
| 2 | hsa_5030 | D00079 |
| 2 | hsa_5030 | D00241 |
| 2 | hsa_5030 | D00364 |
| 2 | hsa_5030 | D00426 |
| 2 | hsa_5030 | D00775 |
| 2 | hsa_5030 | D01227 |
| 2 | hsa_5030 | D01269 |
| 2 | hsa_5030 | D01692 |
| 2 | hsa_5030 | D02342 |
| 2 | hsa_5030 | D02354 |
| 2 | hsa_5030 | D02578 |
| 2 | hsa_5030 | D02725 |
| 2 | hsa_5030 | D03490 |
| 2 | hsa_5031 | D00227 |
| 2 | hsa_5031 | D00274 |
| 2 | hsa_5031 | D00432 |
| 2 | hsa_5031 | D00499 |
| 2 | hsa_5031 | D00522 |
| 2 | hsa_5031 | D00635 |
| 2 | hsa_5031 | D00682 |
| 2 | hsa_5031 | D00684 |
| 2 | hsa_5031 | D00838 |
| 2 | hsa_5031 | D01020 |
| 2 | hsa_5031 | D01024 |
| 2 | hsa_5031 | D01269 |
| 2 | hsa_5031 | D02374 |
| 2 | hsa_5031 | D02578 |
| 2 | hsa_5031 | D03881 |
| 2 | hsa_5031 | D04006 |
| 2 | hsa_5031 | D06396 |
| 2 | hsa_5032 | D00838 |
| 2 | hsa_5032 | D00845 |
| 2 | hsa_5032 | D01071 |
| 2 | hsa_5032 | D01324 |
| 2 | hsa_5032 | D01352 |
| 2 | hsa_5032 | D01462 |
| 2 | hsa_5032 | D01871 |
| 2 | hsa_5032 | D02070 |
| 2 | hsa_5032 | D02147 |
| 2 | hsa_5032 | D02349 |
| 2 | hsa_5032 | D02910 |
| 2 | hsa_552 | D00106 |
| 2 | hsa_552 | D00270 |
| 2 | hsa_552 | D00422 |
| 2 | hsa_552 | D00509 |
| 2 | hsa_552 | D00514 |
| 2 | hsa_552 | D00780 |
| 2 | hsa_552 | D01118 |
| 2 | hsa_552 | D01324 |
| 2 | hsa_552 | D01713 |
| 2 | hsa_552 | D01965 |
| 2 | hsa_552 | D02076 |
| 2 | hsa_552 | D02340 |
| 2 | hsa_552 | D03165 |
| 2 | hsa_552 | D03880 |
| 2 | hsa_554 | D00300 |
| 2 | hsa_554 | D00440 |
| 2 | hsa_554 | D00442 |
| 2 | hsa_554 | D00493 |
| 2 | hsa_554 | D00499 |
| 2 | hsa_554 | D01022 |
| 2 | hsa_554 | D01126 |
| 2 | hsa_554 | D01386 |
| 2 | hsa_554 | D01454 |
| 2 | hsa_554 | D01994 |
| 2 | hsa_554 | D02070 |
| 2 | hsa_554 | D03858 |
| 2 | hsa_554 | D04006 |
| 2 | hsa_554 | D04040 |
| 2 | hsa_554 | D05740 |
| 2 | hsa_554 | D06056 |
| 2 | hsa_56413 | D00059 |
| 2 | hsa_56413 | D00180 |
| 2 | hsa_56413 | D00494 |
| 2 | hsa_56413 | D00499 |
| 2 | hsa_56413 | D00522 |
| 2 | hsa_56413 | D00779 |
| 2 | hsa_56413 | D01103 |
| 2 | hsa_56413 | D01295 |
| 2 | hsa_56413 | D01324 |
| 2 | hsa_56413 | D01462 |
| 2 | hsa_56413 | D01745 |
| 2 | hsa_56413 | D01828 |
| 2 | hsa_56413 | D02354 |
| 2 | hsa_56413 | D02721 |
| 2 | hsa_56413 | D03274 |
| 2 | hsa_56413 | D03490 |
| 2 | hsa_56413 | D04625 |
| 2 | hsa_57105 | D00049 |
| 2 | hsa_57105 | D00139 |
| 2 | hsa_57105 | D00336 |
| 2 | hsa_57105 | D00790 |
| 2 | hsa_57105 | D01051 |
| 2 | hsa_57105 | D01332 |
| 2 | hsa_57105 | D01352 |
| 2 | hsa_57105 | D01712 |
| 2 | hsa_57105 | D01828 |
| 2 | hsa_57105 | D02342 |
| 2 | hsa_57105 | D03654 |
| 2 | hsa_57105 | D03879 |
| 2 | hsa_5724 | D00094 |
| 2 | hsa_5724 | D00234 |
| 2 | hsa_5724 | D00301 |
| 2 | hsa_5724 | D00443 |
| 2 | hsa_5724 | D00480 |
| 2 | hsa_5724 | D00503 |
| 2 | hsa_5724 | D00514 |
| 2 | hsa_5724 | D00574 |
| 2 | hsa_5724 | D00684 |
| 2 | hsa_5724 | D00765 |
| 2 | hsa_5724 | D00775 |
| 2 | hsa_5724 | D00996 |
| 2 | hsa_5724 | D01022 |
| 2 | hsa_5724 | D02578 |
| 2 | hsa_5724 | D03881 |
| 2 | hsa_5731 | D00411 |
| 2 | hsa_5731 | D00454 |
| 2 | hsa_5731 | D00613 |
| 2 | hsa_5731 | D00665 |
| 2 | hsa_5731 | D00769 |
| 2 | hsa_5731 | D00775 |
| 2 | hsa_5731 | D01071 |
| 2 | hsa_5731 | D01324 |
| 2 | hsa_5731 | D01441 |
| 2 | hsa_5731 | D01925 |
| 2 | hsa_5731 | D01964 |
| 2 | hsa_5731 | D02338 |
| 2 | hsa_5731 | D02374 |
| 2 | hsa_5731 | D04006 |
| 2 | hsa_5731 | D05246 |
| 2 | hsa_5732 | D00094 |
| 2 | hsa_5732 | D00106 |
| 2 | hsa_5732 | D00136 |
| 2 | hsa_5732 | D00281 |
| 2 | hsa_5732 | D00364 |
| 2 | hsa_5732 | D00380 |
| 2 | hsa_5732 | D00520 |
| 2 | hsa_5732 | D01242 |
| 2 | hsa_5732 | D02076 |
| 2 | hsa_5732 | D02147 |
| 2 | hsa_5732 | D02279 |
| 2 | hsa_5732 | D02354 |
| 2 | hsa_5732 | D02357 |
| 2 | hsa_5732 | D02671 |
| 2 | hsa_5733 | D00270 |
| 2 | hsa_5733 | D00520 |
| 2 | hsa_5733 | D00522 |
| 2 | hsa_5733 | D00613 |
| 2 | hsa_5733 | D00838 |
| 2 | hsa_5733 | D01652 |
| 2 | hsa_5733 | D01871 |
| 2 | hsa_5733 | D02147 |
| 2 | hsa_5737 | D00095 |
| 2 | hsa_5737 | D00110 |
| 2 | hsa_5737 | D00451 |
| 2 | hsa_5737 | D00454 |
| 2 | hsa_5737 | D00563 |
| 2 | hsa_5737 | D00609 |
| 2 | hsa_5737 | D00682 |
| 2 | hsa_5737 | D00996 |
| 2 | hsa_5737 | D01103 |
| 2 | hsa_5737 | D01227 |
| 2 | hsa_5737 | D01712 |
| 2 | hsa_5737 | D01965 |
| 2 | hsa_5737 | D02354 |
| 2 | hsa_5737 | D02614 |
| 2 | hsa_5737 | D05938 |
| 2 | hsa_5739 | D00281 |
| 2 | hsa_5739 | D00432 |
| 2 | hsa_5739 | D00493 |
| 2 | hsa_5739 | D00676 |
| 2 | hsa_5739 | D01071 |
| 2 | hsa_5739 | D01712 |
| 2 | hsa_5739 | D02327 |
| 2 | hsa_5739 | D02349 |
| 2 | hsa_5739 | D02374 |
| 2 | hsa_5739 | D03881 |
| 2 | hsa_5739 | D05246 |
| 2 | hsa_59340 | D00136 |
| 2 | hsa_59340 | D00380 |
| 2 | hsa_59340 | D00394 |
| 2 | hsa_59340 | D00400 |
| 2 | hsa_59340 | D00483 |
| 2 | hsa_59340 | D00687 |
| 2 | hsa_59340 | D01020 |
| 2 | hsa_59340 | D01332 |
| 2 | hsa_59340 | D02234 |
| 2 | hsa_59340 | D02237 |
| 2 | hsa_59340 | D02278 |
| 2 | hsa_59340 | D02279 |
| 2 | hsa_59340 | D03879 |
| 2 | hsa_6010 | D00106 |
| 2 | hsa_6010 | D00235 |
| 2 | hsa_6010 | D00295 |
| 2 | hsa_6010 | D00318 |
| 2 | hsa_6010 | D00528 |
| 2 | hsa_6010 | D00563 |
| 2 | hsa_6010 | D02250 |
| 2 | hsa_6010 | D02374 |
| 2 | hsa_6010 | D03274 |
| 2 | hsa_6010 | D04375 |
| 2 | hsa_64805 | D00059 |
| 2 | hsa_64805 | D00234 |
| 2 | hsa_64805 | D00503 |
| 2 | hsa_64805 | D00525 |
| 2 | hsa_64805 | D01386 |
| 2 | hsa_64805 | D01603 |
| 2 | hsa_64805 | D02076 |
| 2 | hsa_64805 | D02237 |
| 2 | hsa_64805 | D02279 |
| 2 | hsa_64805 | D02338 |
| 2 | hsa_64805 | D02361 |
| 2 | hsa_64805 | D02578 |
| 2 | hsa_64805 | D02671 |
| 2 | hsa_6751 | D00411 |
| 2 | hsa_6751 | D00419 |
| 2 | hsa_6751 | D00451 |
| 2 | hsa_6751 | D00454 |
| 2 | hsa_6751 | D00480 |
| 2 | hsa_6751 | D00540 |
| 2 | hsa_6751 | D00574 |
| 2 | hsa_6751 | D00607 |
| 2 | hsa_6751 | D00627 |
| 2 | hsa_6751 | D00674 |
| 2 | hsa_6751 | D00780 |
| 2 | hsa_6751 | D00954 |
| 2 | hsa_6751 | D01051 |
| 2 | hsa_6751 | D01713 |
| 2 | hsa_6752 | D00301 |
| 2 | hsa_6752 | D00498 |
| 2 | hsa_6752 | D00522 |
| 2 | hsa_6752 | D00635 |
| 2 | hsa_6752 | D01020 |
| 2 | hsa_6752 | D01603 |
| 2 | hsa_6752 | D02082 |
| 2 | hsa_6752 | D02374 |
| 2 | hsa_6752 | D03880 |
| 2 | hsa_6753 | D00371 |
| 2 | hsa_6753 | D00606 |
| 2 | hsa_6753 | D00607 |
| 2 | hsa_6753 | D00627 |
| 2 | hsa_6753 | D00996 |
| 2 | hsa_6753 | D01022 |
| 2 | hsa_6753 | D01352 |
| 2 | hsa_6753 | D01994 |
| 2 | hsa_6753 | D02082 |
| 2 | hsa_6753 | D02278 |
| 2 | hsa_6753 | D02327 |
| 2 | hsa_6753 | D02349 |
| 2 | hsa_6753 | D02359 |
| 2 | hsa_6753 | D02566 |
| 2 | hsa_6753 | D04006 |
| 2 | hsa_6753 | D04716 |
| 2 | hsa_6755 | D00255 |
| 2 | hsa_6755 | D00356 |
| 2 | hsa_6755 | D00480 |
| 2 | hsa_6755 | D00503 |
| 2 | hsa_6755 | D00509 |
| 2 | hsa_6755 | D00760 |
| 2 | hsa_6755 | D00838 |
| 2 | hsa_6755 | D01024 |
| 2 | hsa_6755 | D01358 |
| 2 | hsa_6755 | D01712 |
| 2 | hsa_6755 | D01891 |
| 2 | hsa_6755 | D02354 |
| 2 | hsa_6755 | D02359 |
| 2 | hsa_6755 | D02374 |
| 2 | hsa_6755 | D02566 |
| 2 | hsa_6755 | D02578 |
| 2 | hsa_6755 | D05246 |
| 2 | hsa_6755 | D05938 |
| 2 | hsa_6915 | D00059 |
| 2 | hsa_6915 | D00274 |
| 2 | hsa_6915 | D00400 |
| 2 | hsa_6915 | D00415 |
| 2 | hsa_6915 | D00480 |
| 2 | hsa_6915 | D00514 |
| 2 | hsa_6915 | D00666 |
| 2 | hsa_6915 | D00674 |
| 2 | hsa_6915 | D00682 |
| 2 | hsa_6915 | D01717 |
| 2 | hsa_6915 | D01925 |
| 2 | hsa_6915 | D02358 |
| 2 | hsa_6915 | D02671 |
| 2 | hsa_6915 | D02725 |
| 2 | hsa_6915 | D03490 |
| 2 | hsa_6915 | D03654 |
| 2 | hsa_6915 | D03858 |
| 2 | hsa_6915 | D03881 |
| 2 | hsa_6915 | D05113 |
| 2 | hsa_6915 | D05341 |
| 2 | hsa_7201 | D00301 |
| 2 | hsa_7201 | D00432 |
| 2 | hsa_7201 | D00520 |
| 2 | hsa_7201 | D00521 |
| 2 | hsa_7201 | D00838 |
| 2 | hsa_7201 | D00987 |
| 2 | hsa_7201 | D01332 |
| 2 | hsa_7201 | D01358 |
| 2 | hsa_7201 | D02066 |
| 2 | hsa_7201 | D02279 |
| 2 | hsa_7201 | D03274 |
| 2 | hsa_7201 | D05246 |
| 2 | hsa_8843 | D00397 |
| 2 | hsa_8843 | D00451 |
| 2 | hsa_8843 | D00503 |
| 2 | hsa_8843 | D00520 |
| 2 | hsa_8843 | D00574 |
| 2 | hsa_8843 | D00606 |
| 2 | hsa_8843 | D00635 |
| 2 | hsa_8843 | D00987 |
| 2 | hsa_8843 | D01022 |
| 2 | hsa_8843 | D01712 |
| 2 | hsa_8843 | D01782 |
| 2 | hsa_8843 | D02278 |
| 2 | hsa_8843 | D02340 |
| 2 | hsa_8843 | D02349 |
| 2 | hsa_8843 | D03642 |
| 2 | hsa_9052 | D00225 |
| 2 | hsa_9052 | D00255 |
| 2 | hsa_9052 | D00419 |
| 2 | hsa_9052 | D00598 |
| 2 | hsa_9052 | D00604 |
| 2 | hsa_9052 | D00845 |
| 2 | hsa_9052 | D01022 |
| 2 | hsa_9052 | D01390 |
| 2 | hsa_9052 | D03415 |
| 2 | hsa_9052 | D03879 |
| 2 | hsa_9052 | D05341 |
| 2 | hsa_9283 | D00094 |
| 2 | hsa_9283 | D00136 |
| 2 | hsa_9283 | D00397 |
| 2 | hsa_9283 | D00442 |
| 2 | hsa_9283 | D00606 |
| 2 | hsa_9283 | D00837 |
| 2 | hsa_9283 | D01020 |
| 2 | hsa_9283 | D01652 |
| 2 | hsa_9283 | D01994 |
| 2 | hsa_9283 | D03858 |
| 2 | hsa_9283 | D04375 |
| 2 | hsa_9283 | D06056 |
| 2 | hsa_9934 | D00136 |
| 2 | hsa_9934 | D00356 |
| 2 | hsa_9934 | D00371 |
| 2 | hsa_9934 | D00410 |
| 2 | hsa_9934 | D00415 |
| 2 | hsa_9934 | D00493 |
| 2 | hsa_9934 | D00509 |
| 2 | hsa_9934 | D00609 |
| 2 | hsa_9934 | D00683 |
| 2 | hsa_9934 | D00684 |
| 2 | hsa_9934 | D00954 |
| 2 | hsa_9934 | D00965 |
| 2 | hsa_9934 | D01782 |
| 2 | hsa_9934 | D01828 |
| 2 | hsa_9934 | D02147 |
| 2 | hsa_9934 | D03880 |
| 2 | hsa_9934 | D03881 |
| 2 | hsa_9934 | D04625 |
