# Supplementary material for: Predicting Drug-Target Interaction Networks Based on Functional Groups and Biological Features
Source: PLoS One. 2010 Mar 11;5(3):e9603. doi: 10.1371/journal.pone.0009603 (PMC2836373; doi:10.1371/journal.pone.0009603)
Supplement: Online Supporting Information S4 — The benchmark dataset for the drug-target nuclear receptor interaction system. It contains 258 gene-drug pair samples, of which 86 are positive and 172 negative. The 1st column of the table indicates the nature of samples with 1 for positive and 2 for negative; the 2nd column shows the code of target gene; and the 3rd column shows the code of drug. All the detailed information for the genes and drugs listed here can be found in KEGG via their codes (see the caption of Online Supporting Information A for further explanation). (0.22 MB DOC) [file pone.0009603.s004.doc]

**Online Supporting Information S4**: The benchmark dataset for the drug-target nuclear receptor interaction system. It contains 258 gene-drug pair samples, of which 86 are positive and 172 negative. The 1st column of the table indicates the nature of samples with 1 for positive and 2 for negative; the 2nd column shows the code of target gene; and the 3rd column shows the code of drug. All the detailed information for the genes and drugs listed here can be found in KEGG via their codes (see the caption of Online Supporting Information A for further explanation).

| **Group** | **Target Gene** | **Drug** |
| --- | --- | --- |
| 1 | hsa_190 | D00094 |
| 1 | hsa_2099 | D00066 |
| 1 | hsa_2099 | D00067 |
| 1 | hsa_2099 | D00105 |
| 1 | hsa_2099 | D00312 |
| 1 | hsa_2099 | D00327 |
| 1 | hsa_2099 | D00554 |
| 1 | hsa_2099 | D00577 |
| 1 | hsa_2099 | D00898 |
| 1 | hsa_2099 | D00950 |
| 1 | hsa_2099 | D00954 |
| 1 | hsa_2099 | D00962 |
| 1 | hsa_2099 | D01161 |
| 1 | hsa_2099 | D01294 |
| 1 | hsa_2099 | D02217 |
| 1 | hsa_2099 | D02367 |
| 1 | hsa_2099 | D04066 |
| 1 | hsa_2100 | D00105 |
| 1 | hsa_2100 | D00577 |
| 1 | hsa_2100 | D01161 |
| 1 | hsa_2100 | D02217 |
| 1 | hsa_2100 | D04066 |
| 1 | hsa_2101 | D00577 |
| 1 | hsa_2103 | D00577 |
| 1 | hsa_2104 | D00577 |
| 1 | hsa_2908 | D00088 |
| 1 | hsa_2908 | D00246 |
| 1 | hsa_2908 | D00585 |
| 1 | hsa_2908 | D01387 |
| 1 | hsa_2908 | D01689 |
| 1 | hsa_3174 | D05341 |
| 1 | hsa_367 | D00075 |
| 1 | hsa_367 | D00327 |
| 1 | hsa_367 | D00462 |
| 1 | hsa_367 | D00585 |
| 1 | hsa_367 | D00586 |
| 1 | hsa_367 | D00956 |
| 1 | hsa_367 | D00961 |
| 1 | hsa_367 | D00965 |
| 1 | hsa_4306 | D00443 |
| 1 | hsa_4306 | D00585 |
| 1 | hsa_4306 | D01115 |
| 1 | hsa_5241 | D00066 |
| 1 | hsa_5241 | D00182 |
| 1 | hsa_5241 | D00585 |
| 1 | hsa_5241 | D00690 |
| 1 | hsa_5241 | D00950 |
| 1 | hsa_5241 | D00951 |
| 1 | hsa_5241 | D00954 |
| 1 | hsa_5241 | D01217 |
| 1 | hsa_5241 | D01294 |
| 1 | hsa_5241 | D02367 |
| 1 | hsa_5465 | D00279 |
| 1 | hsa_5465 | D00565 |
| 1 | hsa_5468 | D00596 |
| 1 | hsa_5468 | D00627 |
| 1 | hsa_5914 | D00094 |
| 1 | hsa_5914 | D00316 |
| 1 | hsa_5914 | D00348 |
| 1 | hsa_5914 | D01132 |
| 1 | hsa_5915 | D00094 |
| 1 | hsa_5915 | D00316 |
| 1 | hsa_5915 | D01132 |
| 1 | hsa_5916 | D00094 |
| 1 | hsa_5916 | D00316 |
| 1 | hsa_5916 | D01132 |
| 1 | hsa_6095 | D00040 |
| 1 | hsa_6095 | D01441 |
| 1 | hsa_6096 | D00094 |
| 1 | hsa_6096 | D01132 |
| 1 | hsa_6097 | D00094 |
| 1 | hsa_6256 | D00094 |
| 1 | hsa_6256 | D00316 |
| 1 | hsa_6256 | D01132 |
| 1 | hsa_6257 | D00094 |
| 1 | hsa_6257 | D00316 |
| 1 | hsa_6257 | D01132 |
| 1 | hsa_7421 | D00187 |
| 1 | hsa_7421 | D00188 |
| 1 | hsa_7421 | D00299 |
| 1 | hsa_7421 | D00930 |
| 1 | hsa_8856 | D00143 |
| 1 | hsa_8856 | D00211 |
| 1 | hsa_8856 | D00730 |
| 1 | hsa_9970 | D00506 |
| 1 | hsa_9971 | D00163 |
| 2 | hsa_190 | D00066 |
| 2 | hsa_190 | D00067 |
| 2 | hsa_190 | D00312 |
| 2 | hsa_190 | D00577 |
| 2 | hsa_190 | D00961 |
| 2 | hsa_190 | D01689 |
| 2 | hsa_2099 | D00188 |
| 2 | hsa_2099 | D00443 |
| 2 | hsa_2099 | D00462 |
| 2 | hsa_2099 | D00506 |
| 2 | hsa_2099 | D00930 |
| 2 | hsa_2099 | D00961 |
| 2 | hsa_2100 | D00163 |
| 2 | hsa_2100 | D00182 |
| 2 | hsa_2100 | D00211 |
| 2 | hsa_2100 | D00327 |
| 2 | hsa_2100 | D00462 |
| 2 | hsa_2100 | D00586 |
| 2 | hsa_2100 | D00596 |
| 2 | hsa_2100 | D01441 |
| 2 | hsa_2101 | D00040 |
| 2 | hsa_2101 | D00143 |
| 2 | hsa_2101 | D00187 |
| 2 | hsa_2101 | D00443 |
| 2 | hsa_2101 | D00950 |
| 2 | hsa_2101 | D02217 |
| 2 | hsa_2103 | D00066 |
| 2 | hsa_2103 | D00246 |
| 2 | hsa_2103 | D00690 |
| 2 | hsa_2103 | D00962 |
| 2 | hsa_2103 | D01217 |
| 2 | hsa_2103 | D01387 |
| 2 | hsa_2104 | D00182 |
| 2 | hsa_2104 | D00316 |
| 2 | hsa_2104 | D00730 |
| 2 | hsa_2104 | D00930 |
| 2 | hsa_2104 | D00954 |
| 2 | hsa_2104 | D01387 |
| 2 | hsa_2908 | D00279 |
| 2 | hsa_2908 | D00312 |
| 2 | hsa_2908 | D00506 |
| 2 | hsa_2908 | D00554 |
| 2 | hsa_2908 | D00690 |
| 2 | hsa_2908 | D01132 |
| 2 | hsa_2908 | D01161 |
| 2 | hsa_3174 | D00143 |
| 2 | hsa_3174 | D00279 |
| 2 | hsa_3174 | D00312 |
| 2 | hsa_3174 | D00586 |
| 2 | hsa_3174 | D01294 |
| 2 | hsa_3174 | D01441 |
| 2 | hsa_3174 | D02217 |
| 2 | hsa_3174 | D02367 |
| 2 | hsa_367 | D00105 |
| 2 | hsa_367 | D00187 |
| 2 | hsa_367 | D00211 |
| 2 | hsa_367 | D00577 |
| 2 | hsa_367 | D00930 |
| 2 | hsa_367 | D01689 |
| 2 | hsa_4306 | D00312 |
| 2 | hsa_4306 | D00316 |
| 2 | hsa_4306 | D01294 |
| 2 | hsa_4306 | D01387 |
| 2 | hsa_4306 | D02367 |
| 2 | hsa_5241 | D00105 |
| 2 | hsa_5241 | D00443 |
| 2 | hsa_5241 | D00596 |
| 2 | hsa_5241 | D00956 |
| 2 | hsa_5241 | D00961 |
| 2 | hsa_5241 | D01161 |
| 2 | hsa_5241 | D01441 |
| 2 | hsa_5241 | D02217 |
| 2 | hsa_5465 | D00506 |
| 2 | hsa_5465 | D00930 |
| 2 | hsa_5465 | D00961 |
| 2 | hsa_5465 | D00962 |
| 2 | hsa_5465 | D02367 |
| 2 | hsa_5468 | D00187 |
| 2 | hsa_5468 | D00279 |
| 2 | hsa_5468 | D00312 |
| 2 | hsa_5468 | D00316 |
| 2 | hsa_5468 | D00348 |
| 2 | hsa_5468 | D00554 |
| 2 | hsa_5468 | D00954 |
| 2 | hsa_5468 | D05341 |
| 2 | hsa_5914 | D00075 |
| 2 | hsa_5914 | D00088 |
| 2 | hsa_5914 | D00143 |
| 2 | hsa_5914 | D00443 |
| 2 | hsa_5914 | D00596 |
| 2 | hsa_5914 | D00730 |
| 2 | hsa_5914 | D00954 |
| 2 | hsa_5914 | D00956 |
| 2 | hsa_5914 | D05341 |
| 2 | hsa_5915 | D00279 |
| 2 | hsa_5915 | D00327 |
| 2 | hsa_5915 | D00586 |
| 2 | hsa_5915 | D00950 |
| 2 | hsa_5915 | D01217 |
| 2 | hsa_5916 | D00075 |
| 2 | hsa_5916 | D00105 |
| 2 | hsa_5916 | D00443 |
| 2 | hsa_5916 | D00554 |
| 2 | hsa_5916 | D00586 |
| 2 | hsa_5916 | D00730 |
| 2 | hsa_5916 | D00965 |
| 2 | hsa_5916 | D01294 |
| 2 | hsa_6095 | D00088 |
| 2 | hsa_6095 | D00143 |
| 2 | hsa_6095 | D00316 |
| 2 | hsa_6095 | D00348 |
| 2 | hsa_6095 | D00961 |
| 2 | hsa_6095 | D00962 |
| 2 | hsa_6096 | D00182 |
| 2 | hsa_6096 | D00327 |
| 2 | hsa_6096 | D00586 |
| 2 | hsa_6096 | D00930 |
| 2 | hsa_6096 | D00962 |
| 2 | hsa_6096 | D00965 |
| 2 | hsa_6096 | D01689 |
| 2 | hsa_6096 | D04066 |
| 2 | hsa_6097 | D00163 |
| 2 | hsa_6097 | D00586 |
| 2 | hsa_6097 | D00627 |
| 2 | hsa_6097 | D01115 |
| 2 | hsa_6097 | D01689 |
| 2 | hsa_6256 | D00075 |
| 2 | hsa_6256 | D00163 |
| 2 | hsa_6256 | D00312 |
| 2 | hsa_6256 | D00565 |
| 2 | hsa_6256 | D00586 |
| 2 | hsa_6256 | D00954 |
| 2 | hsa_6256 | D05341 |
| 2 | hsa_6257 | D00067 |
| 2 | hsa_6257 | D00163 |
| 2 | hsa_6257 | D00182 |
| 2 | hsa_6257 | D00188 |
| 2 | hsa_6257 | D00348 |
| 2 | hsa_6257 | D00462 |
| 2 | hsa_6257 | D00554 |
| 2 | hsa_6257 | D00627 |
| 2 | hsa_6257 | D00956 |
| 2 | hsa_7421 | D00066 |
| 2 | hsa_7421 | D00316 |
| 2 | hsa_7421 | D00443 |
| 2 | hsa_7421 | D00462 |
| 2 | hsa_7421 | D00577 |
| 2 | hsa_7421 | D00586 |
| 2 | hsa_7421 | D01217 |
| 2 | hsa_8856 | D00182 |
| 2 | hsa_8856 | D00565 |
| 2 | hsa_8856 | D00690 |
| 2 | hsa_8856 | D01115 |
| 2 | hsa_8856 | D01689 |
| 2 | hsa_8856 | D02217 |
| 2 | hsa_8856 | D05341 |
| 2 | hsa_9970 | D00163 |
| 2 | hsa_9970 | D00182 |
| 2 | hsa_9970 | D00312 |
| 2 | hsa_9970 | D00327 |
| 2 | hsa_9970 | D00348 |
| 2 | hsa_9970 | D00585 |
| 2 | hsa_9970 | D00961 |
| 2 | hsa_9970 | D01161 |
| 2 | hsa_9970 | D01689 |
| 2 | hsa_9971 | D00075 |
| 2 | hsa_9971 | D00094 |
| 2 | hsa_9971 | D00565 |
| 2 | hsa_9971 | D00577 |
| 2 | hsa_9971 | D00956 |
| 2 | hsa_9971 | D00961 |
| 2 | hsa_9971 | D04066 |
