# Supplementary material for: Predicting Drug-Target Interaction Networks Based on Functional Groups and Biological Features
Source: PLoS One. 2010 Mar 11;5(3):e9603. doi: 10.1371/journal.pone.0009603 (PMC2836373; doi:10.1371/journal.pone.0009603)
Supplement: Online Supporting Information S5 — Output of Maximum Relevancy Minimum Redundancy (mRMR). (1.02 MB DOC) [file pone.0009603.s005.doc]

**Online Supporting Information S5: Output of Maximum Relevancy Minimum Redundancy (mRMR)**

**1. Output of mRMR for the target E (enzyme) class**

| ***** MaxRel features ***** | | | |
| --- | --- | --- | --- |
| Order | Feature | Name | Score |
| 1 | 131 | Target131 | 0.032 |
| 2 | 31 | Target31 | 0.026 |
| 3 | 35 | Target35 | 0.024 |
| 4 | 24 | Target24 | 0.022 |
| 5 | 143 | Drug11 | 0.022 |
| 6 | 92 | Target92 | 0.017 |
| 7 | 148 | Drug16 | 0.014 |
| 8 | 120 | Target120 | 0.014 |
| 9 | 29 | Target29 | 0.013 |
| 10 | 119 | Target119 | 0.012 |
| 11 | 6 | Target6 | 0.01 |
| 12 | 154 | Drug22 | 0.01 |
| 13 | 23 | Target23 | 0.009 |
| 14 | 83 | Target83 | 0.009 |
| 15 | 27 | Target27 | 0.009 |
| 16 | 36 | Target36 | 0.009 |
| 17 | 25 | Target25 | 0.009 |
| 18 | 57 | Target57 | 0.008 |
| 19 | 55 | Target55 | 0.008 |
| 20 | 62 | Target62 | 0.007 |
| 21 | 1 | Target1 | 0.007 |
| 22 | 88 | Target88 | 0.007 |
| 23 | 63 | Target63 | 0.007 |
| 24 | 8 | Target8 | 0.007 |
| 25 | 127 | Target127 | 0.007 |
| 26 | 126 | Target126 | 0.007 |
| 27 | 3 | Target3 | 0.007 |
| 28 | 140 | Drug8 | 0.007 |
| 29 | 105 | Target105 | 0.006 |
| 30 | 99 | Target99 | 0.006 |
| 31 | 141 | Drug9 | 0.006 |
| 32 | 34 | Target34 | 0.006 |
| 33 | 155 | Drug23 | 0.006 |
| 34 | 48 | Target48 | 0.006 |
| 35 | 97 | Target97 | 0.006 |
| 36 | 74 | Target74 | 0.006 |
| 37 | 147 | Drug15 | 0.005 |
| 38 | 50 | Target50 | 0.005 |
| 39 | 158 | Drug26 | 0.005 |
| 40 | 128 | Target128 | 0.005 |
| 41 | 40 | Target40 | 0.005 |
| 42 | 73 | Target73 | 0.005 |
| 43 | 26 | Target26 | 0.005 |
| 44 | 95 | Target95 | 0.004 |
| 45 | 67 | Target67 | 0.004 |
| 46 | 109 | Target109 | 0.004 |
| 47 | 107 | Target107 | 0.004 |
| 48 | 137 | Drug5 | 0.004 |
| 49 | 81 | Target81 | 0.004 |
| 50 | 94 | Target94 | 0.004 |
| 51 | 52 | Target52 | 0.004 |
| 52 | 2 | Target2 | 0.004 |
| 53 | 76 | Target76 | 0.004 |
| 54 | 13 | Target13 | 0.003 |
| 55 | 47 | Target47 | 0.003 |
| 56 | 33 | Target33 | 0.003 |
| 57 | 21 | Target21 | 0.003 |
| 58 | 38 | Target38 | 0.003 |
| 59 | 115 | Target115 | 0.003 |
| 60 | 156 | Drug24 | 0.003 |
| 61 | 117 | Target117 | 0.003 |
| 62 | 71 | Target71 | 0.003 |
| 63 | 44 | Target44 | 0.003 |
| 64 | 65 | Target65 | 0.003 |
| 65 | 51 | Target51 | 0.003 |
| 66 | 93 | Target93 | 0.003 |
| 67 | 138 | Drug6 | 0.003 |
| 68 | 5 | Target5 | 0.002 |
| 69 | 123 | Target123 | 0.002 |
| 70 | 113 | Target113 | 0.002 |
| 71 | 75 | Target75 | 0.002 |
| 72 | 85 | Target85 | 0.002 |
| 73 | 103 | Target103 | 0.002 |
| 74 | 49 | Target49 | 0.002 |
| 75 | 149 | Drug17 | 0.002 |
| 76 | 122 | Target122 | 0.002 |
| 77 | 134 | Drug2 | 0.002 |
| 78 | 17 | Target17 | 0.002 |
| 79 | 108 | Target108 | 0.002 |
| 80 | 77 | Target77 | 0.002 |
| 81 | 28 | Target28 | 0.002 |
| 82 | 66 | Target66 | 0.002 |
| 83 | 20 | Target20 | 0.002 |
| 84 | 116 | Target116 | 0.002 |
| 85 | 30 | Target30 | 0.002 |
| 86 | 22 | Target22 | 0.002 |
| 87 | 106 | Target106 | 0.002 |
| 88 | 89 | Target89 | 0.002 |
| 89 | 80 | Target80 | 0.002 |
| 90 | 46 | Target46 | 0.002 |
| 91 | 39 | Target39 | 0.002 |
| 92 | 59 | Target59 | 0.002 |
| 93 | 139 | Drug7 | 0.002 |
| 94 | 96 | Target96 | 0.002 |
| 95 | 130 | Target130 | 0.001 |
| 96 | 84 | Target84 | 0.001 |
| 97 | 64 | Target64 | 0.001 |
| 98 | 150 | Drug18 | 0.001 |
| 99 | 9 | Target9 | 0.001 |
| 100 | 90 | Target90 | 0.001 |
| 101 | 11 | Target11 | 0.001 |
| 102 | 160 | Drug28 | 0.001 |
| 103 | 78 | Target78 | 0.001 |
| 104 | 136 | Drug4 | 0.001 |
| 105 | 102 | Target102 | 0.001 |
| 106 | 54 | Target54 | 0.001 |
| 107 | 104 | Target104 | 0.001 |
| 108 | 101 | Target101 | 0.001 |
| 109 | 41 | Target41 | 0.001 |
| 110 | 69 | Target69 | 0.001 |
| 111 | 111 | Target111 | 0.001 |
| 112 | 72 | Target72 | 0.001 |
| 113 | 152 | Drug20 | 0.001 |
| 114 | 14 | Target14 | 0.001 |
| 115 | 61 | Target61 | 0.001 |
| 116 | 82 | Target82 | 0.001 |
| 117 | 43 | Target43 | 0.001 |
| 118 | 118 | Target118 | 0.001 |
| 119 | 45 | Target45 | 0.001 |
| 120 | 60 | Target60 | 0.001 |
| 121 | 132 | Target132 | 0.001 |
| 122 | 100 | Target100 | 0.001 |
| 123 | 112 | Target112 | 0.001 |
| 124 | 70 | Target70 | 0.001 |
| 125 | 53 | Target53 | 0.001 |
| 126 | 124 | Target124 | 0.001 |
| 127 | 10 | Target10 | 0.001 |
| 128 | 91 | Target91 | 0.001 |
| 129 | 114 | Target114 | 0.001 |
| 130 | 4 | Target4 | 0.001 |
| 131 | 98 | Target98 | 0.001 |
| 132 | 129 | Target129 | 0 |
| 133 | 125 | Target125 | 0 |
| 134 | 15 | Target15 | 0 |
| 135 | 18 | Target18 | 0 |
| 136 | 37 | Target37 | 0 |
| 137 | 7 | Target7 | 0 |
| 138 | 19 | Target19 | 0 |
| 139 | 16 | Target16 | 0 |
| 140 | 135 | Drug3 | 0 |
| 141 | 56 | Target56 | 0 |
| 142 | 87 | Target87 | 0 |
| 143 | 58 | Target58 | 0 |
| 144 | 68 | Target68 | 0 |
| 145 | 110 | Target110 | 0 |
| 146 | 145 | Drug13 | 0 |
| 147 | 12 | Target12 | 0 |
| 148 | 42 | Target42 | 0 |
| 149 | 146 | Drug14 | 0 |
| 150 | 32 | Target32 | 0 |
| 151 | 79 | Target79 | 0 |
| 152 | 144 | Drug12 | 0 |
| 153 | 121 | Target121 | 0 |
| 154 | 151 | Drug19 | 0 |
| 155 | 159 | Drug27 | 0 |
| 156 | 86 | Target86 | 0 |
| 157 | 133 | Drug1 | 0 |
| 158 | 157 | Drug25 | 0 |
| 159 | 153 | Drug21 | 0 |

*** **mRMR features** ***

| Order | Feature | Name | Score |
| --- | --- | --- | --- |
| 1 | 131 | Target131 | 0.032 |
| 2 | 143 | Drug11 | 0.022 |
| 3 | 154 | Drug22 | 0.008 |
| 4 | 119 | Target119 | 0.006 |
| 5 | 62 | Target62 | 0.005 |
| 6 | 140 | Drug8 | 0.003 |
| 7 | 148 | Drug16 | 0.003 |
| 8 | 31 | Target31 | 0.004 |
| 9 | 155 | Drug23 | 0.003 |
| 10 | 81 | Target81 | 0.003 |
| 11 | 141 | Drug9 | 0.003 |
| 12 | 6 | Target6 | 0.002 |
| 13 | 38 | Target38 | 0.001 |
| 14 | 149 | Drug17 | 0.001 |
| 15 | 138 | Drug6 | 0.001 |
| 16 | 23 | Target23 | 0.001 |
| 17 | 27 | Target27 | 0.001 |
| 18 | 8 | Target8 | 0 |
| 19 | 157 | Drug25 | 0 |
| 20 | 153 | Drug21 | 0 |
| 21 | 142 | Drug10 | 0 |
| 22 | 35 | Target35 | 0 |
| 23 | 137 | Drug5 | 0 |
| 24 | 135 | Drug3 | 0 |
| 25 | 33 | Target33 | 0 |
| 26 | 120 | Target120 | 0 |
| 27 | 126 | Target126 | -0.001 |
| 28 | 32 | Target32 | -0.001 |
| 29 | 29 | Target29 | -0.001 |
| 30 | 150 | Drug18 | -0.001 |
| 31 | 146 | Drug14 | -0.001 |
| 32 | 139 | Drug7 | -0.001 |
| 33 | 147 | Drug15 | -0.001 |
| 34 | 107 | Target107 | -0.001 |
| 35 | 102 | Target102 | -0.001 |
| 36 | 136 | Drug4 | -0.001 |
| 37 | 11 | Target11 | -0.001 |
| 38 | 144 | Drug12 | -0.002 |
| 39 | 117 | Target117 | -0.002 |
| 40 | 44 | Target44 | -0.002 |
| 41 | 83 | Target83 | -0.002 |
| 42 | 156 | Drug24 | -0.002 |
| 43 | 92 | Target92 | -0.002 |
| 44 | 151 | Drug19 | -0.002 |
| 45 | 113 | Target113 | -0.002 |
| 46 | 24 | Target24 | -0.002 |
| 47 | 98 | Target98 | -0.002 |
| 48 | 105 | Target105 | -0.003 |
| 49 | 133 | Drug1 | -0.003 |
| 50 | 159 | Drug27 | -0.003 |
| 51 | 145 | Drug13 | -0.003 |
| 52 | 48 | Target48 | -0.003 |
| 53 | 112 | Target112 | -0.003 |
| 54 | 7 | Target7 | -0.003 |
| 55 | 16 | Target16 | -0.003 |
| 56 | 134 | Drug2 | -0.004 |
| 57 | 67 | Target67 | -0.004 |
| 58 | 122 | Target122 | -0.004 |
| 59 | 34 | Target34 | -0.004 |
| 60 | 124 | Target124 | -0.004 |
| 61 | 123 | Target123 | -0.005 |
| 62 | 103 | Target103 | -0.005 |
| 63 | 160 | Drug28 | -0.005 |
| 64 | 127 | Target127 | -0.005 |
| 65 | 21 | Target21 | -0.005 |
| 66 | 96 | Target96 | -0.005 |
| 67 | 60 | Target60 | -0.006 |
| 68 | 128 | Target128 | -0.006 |
| 69 | 42 | Target42 | -0.006 |
| 70 | 49 | Target49 | -0.006 |
| 71 | 132 | Target132 | -0.006 |
| 72 | 158 | Drug26 | -0.006 |
| 73 | 36 | Target36 | -0.006 |
| 74 | 63 | Target63 | -0.006 |
| 75 | 121 | Target121 | -0.006 |
| 76 | 152 | Drug20 | -0.007 |
| 77 | 91 | Target91 | -0.007 |
| 78 | 116 | Target116 | -0.007 |
| 79 | 45 | Target45 | -0.007 |
| 80 | 47 | Target47 | -0.007 |
| 81 | 125 | Target125 | -0.007 |
| 82 | 101 | Target101 | -0.007 |
| 83 | 130 | Target130 | -0.008 |
| 84 | 65 | Target65 | -0.008 |
| 85 | 37 | Target37 | -0.008 |
| 86 | 88 | Target88 | -0.008 |
| 87 | 115 | Target115 | -0.008 |
| 88 | 95 | Target95 | -0.008 |
| 89 | 99 | Target99 | -0.008 |
| 90 | 82 | Target82 | -0.009 |
| 91 | 118 | Target118 | -0.009 |
| 92 | 69 | Target69 | -0.009 |
| 93 | 87 | Target87 | -0.009 |
| 94 | 86 | Target86 | -0.009 |
| 95 | 10 | Target10 | -0.009 |
| 96 | 93 | Target93 | -0.009 |
| 97 | 129 | Target129 | -0.01 |
| 98 | 46 | Target46 | -0.01 |
| 99 | 70 | Target70 | -0.01 |
| 100 | 76 | Target76 | -0.011 |
| 101 | 30 | Target30 | -0.011 |
| 102 | 61 | Target61 | -0.011 |
| 103 | 80 | Target80 | -0.011 |
| 104 | 55 | Target55 | -0.011 |
| 105 | 104 | Target104 | -0.012 |
| 106 | 9 | Target9 | -0.012 |
| 107 | 106 | Target106 | -0.012 |
| 108 | 25 | Target25 | -0.012 |
| 109 | 43 | Target43 | -0.012 |
| 110 | 56 | Target56 | -0.012 |
| 111 | 53 | Target53 | -0.013 |
| 112 | 40 | Target40 | -0.013 |
| 113 | 57 | Target57 | -0.013 |
| 114 | 54 | Target54 | -0.013 |
| 115 | 1 | Target1 | -0.014 |
| 116 | 59 | Target59 | -0.014 |
| 117 | 68 | Target68 | -0.014 |
| 118 | 26 | Target26 | -0.015 |
| 119 | 12 | Target12 | -0.015 |
| 120 | 114 | Target114 | -0.015 |
| 121 | 100 | Target100 | -0.015 |
| 122 | 51 | Target51 | -0.015 |
| 123 | 97 | Target97 | -0.016 |
| 124 | 18 | Target18 | -0.017 |
| 125 | 74 | Target74 | -0.017 |
| 126 | 64 | Target64 | -0.017 |
| 127 | 41 | Target41 | -0.017 |
| 128 | 4 | Target4 | -0.018 |
| 129 | 14 | Target14 | -0.018 |
| 130 | 22 | Target22 | -0.018 |
| 131 | 109 | Target109 | -0.018 |
| 132 | 90 | Target90 | -0.019 |
| 133 | 20 | Target20 | -0.02 |
| 134 | 89 | Target89 | -0.021 |
| 135 | 3 | Target3 | -0.021 |
| 136 | 85 | Target85 | -0.021 |
| 137 | 5 | Target5 | -0.021 |
| 138 | 13 | Target13 | -0.022 |
| 139 | 111 | Target111 | -0.022 |
| 140 | 94 | Target94 | -0.022 |
| 141 | 39 | Target39 | -0.022 |
| 142 | 78 | Target78 | -0.023 |
| 143 | 17 | Target17 | -0.025 |
| 144 | 58 | Target58 | -0.025 |
| 145 | 75 | Target75 | -0.025 |
| 146 | 19 | Target19 | -0.026 |
| 147 | 110 | Target110 | -0.027 |
| 148 | 15 | Target15 | -0.029 |
| 149 | 73 | Target73 | -0.029 |
| 150 | 71 | Target71 | -0.03 |
| 151 | 108 | Target108 | -0.03 |
| 152 | 84 | Target84 | -0.03 |
| 153 | 50 | Target50 | -0.031 |
| 154 | 79 | Target79 | -0.031 |
| 155 | 52 | Target52 | -0.033 |
| 156 | 77 | Target77 | -0.035 |
| 157 | 2 | Target2 | -0.035 |
| 158 | 28 | Target28 | -0.039 |
| 159 | 72 | Target72 | -0.044 |
| 160 | 66 | Target66 | -0.044 |

**2. Output of mRMR for the target G-protein coupled receptor (GPCR ) class**

*** MaxRel features ***

| Order | Feature | Name | Score |
| --- | --- | --- | --- |
| 1 | 79 | Target79 | 0.055 |
| 2 | 89 | Target89 | 0.054 |
| 3 | 9 | Target9 | 0.051 |
| 4 | 47 | Target47 | 0.05 |
| 5 | 19 | Target19 | 0.05 |
| 6 | 88 | Target88 | 0.046 |
| 7 | 62 | Target62 | 0.044 |
| 8 | 39 | Target39 | 0.044 |
| 9 | 40 | Target40 | 0.043 |
| 10 | 43 | Target43 | 0.043 |
| 11 | 41 | Target41 | 0.043 |
| 12 | 22 | Target22 | 0.038 |
| 13 | 8 | Target8 | 0.035 |
| 14 | 24 | Target24 | 0.035 |
| 15 | 10 | Target10 | 0.034 |
| 16 | 90 | Target90 | 0.032 |
| 17 | 48 | Target48 | 0.032 |
| 18 | 71 | Target71 | 0.032 |
| 19 | 3 | Target3 | 0.028 |
| 20 | 63 | Target63 | 0.028 |
| 21 | 74 | Target74 | 0.028 |
| 22 | 35 | Target35 | 0.028 |
| 23 | 26 | Target26 | 0.026 |
| 24 | 30 | Target30 | 0.026 |
| 25 | 28 | Target28 | 0.025 |
| 26 | 108 | Target108 | 0.025 |
| 27 | 17 | Target17 | 0.025 |
| 28 | 66 | Target66 | 0.025 |
| 29 | 77 | Target77 | 0.025 |
| 30 | 69 | Target69 | 0.025 |
| 31 | 111 | Target111 | 0.025 |
| 32 | 105 | Target105 | 0.024 |
| 33 | 36 | Target36 | 0.023 |
| 34 | 5 | Target5 | 0.022 |
| 35 | 97 | Target97 | 0.021 |
| 36 | 31 | Target31 | 0.021 |
| 37 | 80 | Target80 | 0.021 |
| 38 | 20 | Target20 | 0.02 |
| 39 | 1 | Target1 | 0.02 |
| 40 | 64 | Target64 | 0.019 |
| 41 | 115 | Target115 | 0.019 |
| 42 | 42 | Target42 | 0.019 |
| 43 | 110 | Target110 | 0.018 |
| 44 | 68 | Target68 | 0.018 |
| 45 | 6 | Target6 | 0.018 |
| 46 | 73 | Target73 | 0.017 |
| 47 | 46 | Target46 | 0.017 |
| 48 | 56 | Target56 | 0.017 |
| 49 | 50 | Target50 | 0.016 |
| 50 | 125 | Target125 | 0.016 |
| 51 | 55 | Target55 | 0.016 |
| 52 | 106 | Target106 | 0.016 |
| 53 | 130 | Target130 | 0.015 |
| 54 | 122 | Target122 | 0.015 |
| 55 | 131 | Target131 | 0.015 |
| 56 | 18 | Target18 | 0.014 |
| 57 | 37 | Target37 | 0.014 |
| 58 | 76 | Target76 | 0.014 |
| 59 | 58 | Target58 | 0.013 |
| 60 | 128 | Target128 | 0.013 |
| 61 | 23 | Target23 | 0.013 |
| 62 | 29 | Target29 | 0.012 |
| 63 | 78 | Target78 | 0.012 |
| 64 | 21 | Target21 | 0.012 |
| 65 | 98 | Target98 | 0.012 |
| 66 | 61 | Target61 | 0.011 |
| 67 | 81 | Target81 | 0.011 |
| 68 | 93 | Target93 | 0.01 |
| 69 | 132 | Target132 | 0.01 |
| 70 | 84 | Target84 | 0.01 |
| 71 | 140 | Drug8 | 0.01 |
| 72 | 67 | Target67 | 0.01 |
| 73 | 109 | Target109 | 0.01 |
| 74 | 75 | Target75 | 0.009 |
| 75 | 72 | Target72 | 0.009 |
| 76 | 51 | Target51 | 0.008 |
| 77 | 54 | Target54 | 0.008 |
| 78 | 120 | Target120 | 0.008 |
| 79 | 2 | Target2 | 0.008 |
| 80 | 99 | Target99 | 0.008 |
| 81 | 123 | Target123 | 0.007 |
| 82 | 25 | Target25 | 0.007 |
| 83 | 4 | Target4 | 0.007 |
| 84 | 113 | Target113 | 0.007 |
| 85 | 85 | Target85 | 0.007 |
| 86 | 49 | Target49 | 0.007 |
| 87 | 92 | Target92 | 0.007 |
| 88 | 158 | Drug26 | 0.006 |
| 89 | 104 | Target104 | 0.006 |
| 90 | 114 | Target114 | 0.006 |
| 91 | 116 | Target116 | 0.006 |
| 92 | 127 | Target127 | 0.006 |
| 93 | 129 | Target129 | 0.005 |
| 94 | 57 | Target57 | 0.005 |
| 95 | 14 | Target14 | 0.005 |
| 96 | 117 | Target117 | 0.005 |
| 97 | 44 | Target44 | 0.005 |
| 98 | 15 | Target15 | 0.005 |
| 99 | 155 | Drug23 | 0.004 |
| 100 | 119 | Target119 | 0.004 |
| 101 | 94 | Target94 | 0.004 |
| 102 | 52 | Target52 | 0.004 |
| 103 | 11 | Target11 | 0.004 |
| 104 | 27 | Target27 | 0.004 |
| 105 | 121 | Target121 | 0.004 |
| 106 | 159 | Drug27 | 0.004 |
| 107 | 59 | Target59 | 0.004 |
| 108 | 154 | Drug22 | 0.004 |
| 109 | 124 | Target124 | 0.003 |
| 110 | 91 | Target91 | 0.003 |
| 111 | 150 | Drug18 | 0.003 |
| 112 | 134 | Drug2 | 0.003 |
| 113 | 13 | Target13 | 0.003 |
| 114 | 38 | Target38 | 0.003 |
| 115 | 96 | Target96 | 0.003 |
| 116 | 133 | Drug1 | 0.003 |
| 117 | 95 | Target95 | 0.003 |
| 118 | 101 | Target101 | 0.002 |
| 119 | 152 | Drug20 | 0.002 |
| 120 | 65 | Target65 | 0.002 |
| 121 | 60 | Target60 | 0.002 |
| 122 | 103 | Target103 | 0.002 |
| 123 | 100 | Target100 | 0.002 |
| 124 | 70 | Target70 | 0.002 |
| 125 | 112 | Target112 | 0.002 |
| 126 | 136 | Drug4 | 0.002 |
| 127 | 83 | Target83 | 0.001 |
| 128 | 156 | Drug24 | 0.001 |
| 129 | 53 | Target53 | 0.001 |
| 130 | 102 | Target102 | 0.001 |
| 131 | 126 | Target126 | 0.001 |
| 132 | 141 | Drug9 | 0.001 |
| 133 | 143 | Drug11 | 0.001 |
| 134 | 137 | Drug5 | 0.001 |
| 135 | 33 | Target33 | 0.001 |
| 136 | 160 | Drug28 | 0.001 |
| 137 | 146 | Drug14 | 0.001 |
| 138 | 7 | Target7 | 0 |
| 139 | 87 | Target87 | 0 |
| 140 | 107 | Target107 | 0 |
| 141 | 149 | Drug17 | 0 |
| 142 | 86 | Target86 | 0 |
| 143 | 34 | Target34 | 0 |
| 144 | 118 | Target118 | 0 |
| 145 | 147 | Drug15 | 0 |
| 146 | 148 | Drug16 | 0 |
| 147 | 139 | Drug7 | 0 |
| 148 | 12 | Target12 | 0 |
| 149 | 45 | Target45 | 0 |
| 150 | 144 | Drug12 | 0 |
| 151 | 82 | Target82 | 0 |
| 152 | 145 | Drug13 | 0 |
| 153 | 16 | Target16 | 0 |
| 154 | 151 | Drug19 | 0 |
| 155 | 157 | Drug25 | 0 |
| 156 | 138 | Drug6 | 0 |
| 157 | 135 | Drug3 | 0 |
| 158 | 32 | Target32 | 0 |
| 159 | 153 | Drug21 | 0 |

*** mRMR features ***

| Order | Feature | Name | Score |
| --- | --- | --- | --- |
| 1 | 79 | Target79 | 0.055 |
| 2 | 140 | Drug8 | 0.008 |
| 3 | 117 | Target117 | 0.003 |
| 4 | 150 | Drug18 | 0.002 |
| 5 | 35 | Target35 | 0.006 |
| 6 | 26 | Target26 | 0.003 |
| 7 | 133 | Drug1 | 0.001 |
| 8 | 89 | Target89 | 0.006 |
| 9 | 156 | Drug24 | 0.001 |
| 10 | 136 | Drug4 | 0 |
| 11 | 97 | Target97 | 0.002 |
| 12 | 143 | Drug11 | 0 |
| 13 | 37 | Target37 | 0.001 |
| 14 | 155 | Drug23 | 0 |
| 15 | 88 | Target88 | 0.002 |
| 16 | 157 | Drug25 | 0 |
| 17 | 138 | Drug6 | 0 |
| 18 | 152 | Drug20 | 0 |
| 19 | 135 | Drug3 | 0 |
| 20 | 74 | Target74 | 0.001 |
| 21 | 39 | Target39 | 0.001 |
| 22 | 32 | Target32 | 0 |
| 23 | 153 | Drug21 | 0 |
| 24 | 142 | Drug10 | 0 |
| 25 | 21 | Target21 | 0 |
| 26 | 147 | Drug15 | 0 |
| 27 | 22 | Target22 | 0.001 |
| 28 | 146 | Drug14 | 0 |
| 29 | 9 | Target9 | 0 |
| 30 | 139 | Drug7 | -0.001 |
| 31 | 149 | Drug17 | -0.001 |
| 32 | 62 | Target62 | 0.001 |
| 33 | 137 | Drug5 | -0.002 |
| 34 | 159 | Drug27 | -0.002 |
| 35 | 41 | Target41 | -0.001 |
| 36 | 160 | Drug28 | -0.003 |
| 37 | 144 | Drug12 | -0.003 |
| 38 | 60 | Target60 | -0.003 |
| 39 | 141 | Drug9 | -0.004 |
| 40 | 105 | Target105 | -0.004 |
| 41 | 151 | Drug19 | -0.004 |
| 42 | 134 | Drug2 | -0.004 |
| 43 | 110 | Target110 | -0.004 |
| 44 | 71 | Target71 | -0.005 |
| 45 | 148 | Drug16 | -0.005 |
| 46 | 154 | Drug22 | -0.006 |
| 47 | 47 | Target47 | -0.006 |
| 48 | 145 | Drug13 | -0.007 |
| 49 | 11 | Target11 | -0.007 |
| 50 | 10 | Target10 | -0.006 |
| 51 | 7 | Target7 | -0.008 |
| 52 | 64 | Target64 | -0.008 |
| 53 | 8 | Target8 | -0.008 |
| 54 | 65 | Target65 | -0.01 |
| 55 | 5 | Target5 | -0.01 |
| 56 | 42 | Target42 | -0.01 |
| 57 | 158 | Drug26 | -0.011 |
| 58 | 98 | Target98 | -0.011 |
| 59 | 49 | Target49 | -0.011 |
| 60 | 70 | Target70 | -0.011 |
| 61 | 81 | Target81 | -0.011 |
| 62 | 82 | Target82 | -0.011 |
| 63 | 115 | Target115 | -0.011 |
| 64 | 130 | Target130 | -0.012 |
| 65 | 45 | Target45 | -0.012 |
| 66 | 16 | Target16 | -0.013 |
| 67 | 54 | Target54 | -0.013 |
| 68 | 19 | Target19 | -0.013 |
| 69 | 107 | Target107 | -0.014 |
| 70 | 1 | Target1 | -0.015 |
| 71 | 121 | Target121 | -0.015 |
| 72 | 91 | Target91 | -0.016 |
| 73 | 93 | Target93 | -0.015 |
| 74 | 87 | Target87 | -0.016 |
| 75 | 30 | Target30 | -0.017 |
| 76 | 106 | Target106 | -0.018 |
| 77 | 102 | Target102 | -0.018 |
| 78 | 56 | Target56 | -0.019 |
| 79 | 128 | Target128 | -0.019 |
| 80 | 132 | Target132 | -0.02 |
| 81 | 36 | Target36 | -0.02 |
| 82 | 63 | Target63 | -0.02 |
| 83 | 61 | Target61 | -0.021 |
| 84 | 112 | Target112 | -0.021 |
| 85 | 68 | Target68 | -0.021 |
| 86 | 69 | Target69 | -0.022 |
| 87 | 90 | Target90 | -0.023 |
| 88 | 125 | Target125 | -0.023 |
| 89 | 104 | Target104 | -0.024 |
| 90 | 126 | Target126 | -0.024 |
| 91 | 122 | Target122 | -0.024 |
| 92 | 27 | Target27 | -0.024 |
| 93 | 99 | Target99 | -0.025 |
| 94 | 6 | Target6 | -0.025 |
| 95 | 34 | Target34 | -0.026 |
| 96 | 86 | Target86 | -0.026 |
| 97 | 131 | Target131 | -0.026 |
| 98 | 123 | Target123 | -0.027 |
| 99 | 85 | Target85 | -0.028 |
| 100 | 116 | Target116 | -0.028 |
| 101 | 53 | Target53 | -0.028 |
| 102 | 67 | Target67 | -0.029 |
| 103 | 127 | Target127 | -0.03 |
| 104 | 24 | Target24 | -0.03 |
| 105 | 48 | Target48 | -0.032 |
| 106 | 124 | Target124 | -0.033 |
| 107 | 23 | Target23 | -0.033 |
| 108 | 76 | Target76 | -0.034 |
| 109 | 57 | Target57 | -0.035 |
| 110 | 40 | Target40 | -0.036 |
| 111 | 101 | Target101 | -0.037 |
| 112 | 96 | Target96 | -0.038 |
| 113 | 118 | Target118 | -0.038 |
| 114 | 28 | Target28 | -0.038 |
| 115 | 14 | Target14 | -0.039 |
| 116 | 95 | Target95 | -0.04 |
| 117 | 12 | Target12 | -0.04 |
| 118 | 73 | Target73 | -0.041 |
| 119 | 109 | Target109 | -0.041 |
| 120 | 29 | Target29 | -0.042 |
| 121 | 80 | Target80 | -0.042 |
| 122 | 120 | Target120 | -0.042 |
| 123 | 33 | Target33 | -0.043 |
| 124 | 103 | Target103 | -0.043 |
| 125 | 100 | Target100 | -0.044 |
| 126 | 119 | Target119 | -0.045 |
| 127 | 13 | Target13 | -0.046 |
| 128 | 25 | Target25 | -0.046 |
| 129 | 111 | Target111 | -0.047 |
| 130 | 113 | Target113 | -0.047 |
| 131 | 38 | Target38 | -0.047 |
| 132 | 94 | Target94 | -0.048 |
| 133 | 58 | Target58 | -0.048 |
| 134 | 114 | Target114 | -0.05 |
| 135 | 15 | Target15 | -0.051 |
| 136 | 108 | Target108 | -0.051 |
| 137 | 55 | Target55 | -0.053 |
| 138 | 46 | Target46 | -0.053 |
| 139 | 31 | Target31 | -0.055 |
| 140 | 44 | Target44 | -0.056 |
| 141 | 84 | Target84 | -0.056 |
| 142 | 20 | Target20 | -0.057 |
| 143 | 83 | Target83 | -0.058 |
| 144 | 75 | Target75 | -0.058 |
| 145 | 17 | Target17 | -0.059 |
| 146 | 129 | Target129 | -0.06 |
| 147 | 52 | Target52 | -0.061 |
| 148 | 43 | Target43 | -0.062 |
| 149 | 51 | Target51 | -0.063 |
| 150 | 78 | Target78 | -0.063 |
| 151 | 59 | Target59 | -0.064 |
| 152 | 4 | Target4 | -0.064 |
| 153 | 3 | Target3 | -0.066 |
| 154 | 18 | Target18 | -0.068 |
| 155 | 66 | Target66 | -0.069 |
| 156 | 92 | Target92 | -0.073 |
| 157 | 77 | Target77 | -0.076 |
| 158 | 50 | Target50 | -0.078 |
| 159 | 72 | Target72 | -0.087 |
| 160 | 2 | Target2 | -0.098 |

**3. Output of mRMR for the target Ion Channel) (IC) class**

*** MaxRel features ***

| Order | Feature | Name | Score |
| --- | --- | --- | --- |
| 1 | 23 | Target23 | 0.017 |
| 2 | 145 | Drug13 | 0.015 |
| 3 | 103 | Target103 | 0.011 |
| 4 | 27 | Target27 | 0.011 |
| 5 | 46 | Target46 | 0.01 |
| 6 | 25 | Target25 | 0.009 |
| 7 | 29 | Target29 | 0.009 |
| 8 | 118 | Target118 | 0.009 |
| 9 | 119 | Target119 | 0.008 |
| 10 | 34 | Target34 | 0.008 |
| 11 | 63 | Target63 | 0.007 |
| 12 | 99 | Target99 | 0.007 |
| 13 | 146 | Drug14 | 0.007 |
| 14 | 75 | Target75 | 0.007 |
| 15 | 80 | Target80 | 0.007 |
| 16 | 26 | Target26 | 0.007 |
| 17 | 124 | Target124 | 0.007 |
| 18 | 114 | Target114 | 0.007 |
| 19 | 109 | Target109 | 0.006 |
| 20 | 67 | Target67 | 0.006 |
| 21 | 158 | Drug26 | 0.006 |
| 22 | 20 | Target20 | 0.006 |
| 23 | 2 | Target2 | 0.006 |
| 24 | 128 | Target128 | 0.006 |
| 25 | 35 | Target35 | 0.006 |
| 26 | 17 | Target17 | 0.006 |
| 27 | 66 | Target66 | 0.006 |
| 28 | 28 | Target28 | 0.006 |
| 29 | 108 | Target108 | 0.006 |
| 30 | 77 | Target77 | 0.006 |
| 31 | 10 | Target10 | 0.006 |
| 32 | 97 | Target97 | 0.006 |
| 33 | 40 | Target40 | 0.006 |
| 34 | 130 | Target130 | 0.006 |
| 35 | 62 | Target62 | 0.006 |
| 36 | 44 | Target44 | 0.006 |
| 37 | 55 | Target55 | 0.006 |
| 38 | 82 | Target82 | 0.006 |
| 39 | 38 | Target38 | 0.006 |
| 40 | 41 | Target41 | 0.005 |
| 41 | 126 | Target126 | 0.005 |
| 42 | 88 | Target88 | 0.005 |
| 43 | 33 | Target33 | 0.005 |
| 44 | 58 | Target58 | 0.005 |
| 45 | 8 | Target8 | 0.005 |
| 46 | 53 | Target53 | 0.005 |
| 47 | 117 | Target117 | 0.005 |
| 48 | 39 | Target39 | 0.005 |
| 49 | 57 | Target57 | 0.005 |
| 50 | 12 | Target12 | 0.005 |
| 51 | 19 | Target19 | 0.004 |
| 52 | 56 | Target56 | 0.004 |
| 53 | 72 | Target72 | 0.004 |
| 54 | 64 | Target64 | 0.004 |
| 55 | 104 | Target104 | 0.004 |
| 56 | 50 | Target50 | 0.004 |
| 57 | 30 | Target30 | 0.004 |
| 58 | 98 | Target98 | 0.004 |
| 59 | 9 | Target9 | 0.004 |
| 60 | 22 | Target22 | 0.004 |
| 61 | 89 | Target89 | 0.004 |
| 62 | 136 | Drug4 | 0.004 |
| 63 | 13 | Target13 | 0.004 |
| 64 | 106 | Target106 | 0.004 |
| 65 | 96 | Target96 | 0.004 |
| 66 | 79 | Target79 | 0.004 |
| 67 | 31 | Target31 | 0.004 |
| 68 | 159 | Drug27 | 0.004 |
| 69 | 111 | Target111 | 0.003 |
| 70 | 69 | Target69 | 0.003 |
| 71 | 144 | Drug12 | 0.003 |
| 72 | 43 | Target43 | 0.003 |
| 73 | 90 | Target90 | 0.003 |
| 74 | 120 | Target120 | 0.003 |
| 75 | 49 | Target49 | 0.003 |
| 76 | 122 | Target122 | 0.003 |
| 77 | 5 | Target5 | 0.003 |
| 78 | 92 | Target92 | 0.003 |
| 79 | 115 | Target115 | 0.003 |
| 80 | 59 | Target59 | 0.003 |
| 81 | 155 | Drug23 | 0.003 |
| 82 | 83 | Target83 | 0.003 |
| 83 | 73 | Target73 | 0.002 |
| 84 | 129 | Target129 | 0.002 |
| 85 | 42 | Target42 | 0.002 |
| 86 | 101 | Target101 | 0.002 |
| 87 | 152 | Drug20 | 0.002 |
| 88 | 105 | Target105 | 0.002 |
| 89 | 95 | Target95 | 0.002 |
| 90 | 107 | Target107 | 0.002 |
| 91 | 14 | Target14 | 0.002 |
| 92 | 127 | Target127 | 0.002 |
| 93 | 24 | Target24 | 0.002 |
| 94 | 16 | Target16 | 0.002 |
| 95 | 100 | Target100 | 0.002 |
| 96 | 110 | Target110 | 0.002 |
| 97 | 68 | Target68 | 0.002 |
| 98 | 85 | Target85 | 0.002 |
| 99 | 1 | Target1 | 0.002 |
| 100 | 113 | Target113 | 0.002 |
| 101 | 15 | Target15 | 0.001 |
| 102 | 134 | Drug2 | 0.001 |
| 103 | 6 | Target6 | 0.001 |
| 104 | 51 | Target51 | 0.001 |
| 105 | 18 | Target18 | 0.001 |
| 106 | 36 | Target36 | 0.001 |
| 107 | 52 | Target52 | 0.001 |
| 108 | 94 | Target94 | 0.001 |
| 109 | 65 | Target65 | 0.001 |
| 110 | 147 | Drug15 | 0.001 |
| 111 | 37 | Target37 | 0.001 |
| 112 | 45 | Target45 | 0.001 |
| 113 | 47 | Target47 | 0.001 |
| 114 | 78 | Target78 | 0.001 |
| 115 | 54 | Target54 | 0.001 |
| 116 | 3 | Target3 | 0.001 |
| 117 | 71 | Target71 | 0.001 |
| 118 | 76 | Target76 | 0.001 |
| 119 | 131 | Target131 | 0.001 |
| 120 | 154 | Drug22 | 0.001 |
| 121 | 87 | Target87 | 0.001 |
| 122 | 121 | Target121 | 0.001 |
| 123 | 116 | Target116 | 0.001 |
| 124 | 60 | Target60 | 0.001 |
| 125 | 148 | Drug16 | 0.001 |
| 126 | 132 | Target132 | 0.001 |
| 127 | 4 | Target4 | 0 |
| 128 | 84 | Target84 | 0 |
| 129 | 61 | Target61 | 0 |
| 130 | 112 | Target112 | 0 |
| 131 | 70 | Target70 | 0 |
| 132 | 140 | Drug8 | 0 |
| 133 | 91 | Target91 | 0 |
| 134 | 143 | Drug11 | 0 |
| 135 | 48 | Target48 | 0 |
| 136 | 81 | Target81 | 0 |
| 137 | 156 | Drug24 | 0 |
| 138 | 21 | Target21 | 0 |
| 139 | 141 | Drug9 | 0 |
| 140 | 74 | Target74 | 0 |
| 141 | 123 | Target123 | 0 |
| 142 | 151 | Drug19 | 0 |
| 143 | 7 | Target7 | 0 |
| 144 | 139 | Drug7 | 0 |
| 145 | 125 | Target125 | 0 |
| 146 | 11 | Target11 | 0 |
| 147 | 137 | Drug5 | 0 |
| 148 | 160 | Drug28 | 0 |
| 149 | 102 | Target102 | 0 |
| 150 | 93 | Target93 | 0 |
| 151 | 133 | Drug1 | 0 |
| 152 | 135 | Drug3 | 0 |
| 153 | 157 | Drug25 | 0 |
| 154 | 149 | Drug17 | 0 |
| 155 | 150 | Drug18 | 0 |
| 156 | 153 | Drug21 | 0 |
| 157 | 138 | Drug6 | 0 |
| 158 | 142 | Drug10 | 0 |
| 159 | 32 | Target32 | 0 |

*** mRMR features ***

| Order | Feature | Name | Score |
| --- | --- | --- | --- |
| 1 | 23 | Target23 | 0.017 |
| 2 | 145 | Drug13 | 0.014 |
| 3 | 10 | Target10 | 0.004 |
| 4 | 136 | Drug4 | 0.003 |
| 5 | 118 | Target118 | 0.002 |
| 6 | 155 | Drug23 | 0.001 |
| 7 | 146 | Drug14 | 0.001 |
| 8 | 98 | Target98 | 0.001 |
| 9 | 38 | Target38 | 0 |
| 10 | 144 | Drug12 | 0 |
| 11 | 135 | Drug3 | 0 |
| 12 | 157 | Drug25 | 0 |
| 13 | 65 | Target65 | 0 |
| 14 | 158 | Drug26 | 0 |
| 15 | 27 | Target27 | 0 |
| 16 | 149 | Drug17 | 0 |
| 17 | 150 | Drug18 | 0 |
| 18 | 153 | Drug21 | 0 |
| 19 | 138 | Drug6 | 0 |
| 20 | 142 | Drug10 | 0 |
| 21 | 32 | Target32 | 0 |
| 22 | 46 | Target46 | 0 |
| 23 | 159 | Drug27 | 0 |
| 24 | 16 | Target16 | 0 |
| 25 | 143 | Drug11 | -0.001 |
| 26 | 42 | Target42 | -0.001 |
| 27 | 147 | Drug15 | -0.001 |
| 28 | 140 | Drug8 | -0.001 |
| 29 | 117 | Target117 | -0.001 |
| 30 | 99 | Target99 | -0.001 |
| 31 | 134 | Drug2 | -0.002 |
| 32 | 139 | Drug7 | -0.002 |
| 33 | 26 | Target26 | -0.002 |
| 34 | 152 | Drug20 | -0.002 |
| 35 | 148 | Drug16 | -0.002 |
| 36 | 124 | Target124 | -0.002 |
| 37 | 128 | Target128 | -0.002 |
| 38 | 12 | Target12 | -0.003 |
| 39 | 61 | Target61 | -0.003 |
| 40 | 119 | Target119 | -0.003 |
| 41 | 160 | Drug28 | -0.003 |
| 42 | 107 | Target107 | -0.003 |
| 43 | 137 | Drug5 | -0.004 |
| 44 | 106 | Target106 | -0.003 |
| 45 | 49 | Target49 | -0.004 |
| 46 | 133 | Drug1 | -0.004 |
| 47 | 88 | Target88 | -0.004 |
| 48 | 156 | Drug24 | -0.004 |
| 49 | 130 | Target130 | -0.005 |
| 50 | 53 | Target53 | -0.005 |
| 51 | 81 | Target81 | -0.005 |
| 52 | 141 | Drug9 | -0.005 |
| 53 | 60 | Target60 | -0.006 |
| 54 | 87 | Target87 | -0.006 |
| 55 | 109 | Target109 | -0.006 |
| 56 | 112 | Target112 | -0.006 |
| 57 | 80 | Target80 | -0.006 |
| 58 | 33 | Target33 | -0.007 |
| 59 | 11 | Target11 | -0.007 |
| 60 | 45 | Target45 | -0.008 |
| 61 | 4 | Target4 | -0.008 |
| 62 | 114 | Target114 | -0.008 |
| 63 | 25 | Target25 | -0.008 |
| 64 | 151 | Drug19 | -0.009 |
| 65 | 63 | Target63 | -0.009 |
| 66 | 111 | Target111 | -0.009 |
| 67 | 102 | Target102 | -0.009 |
| 68 | 126 | Target126 | -0.01 |
| 69 | 82 | Target82 | -0.01 |
| 70 | 154 | Drug22 | -0.01 |
| 71 | 29 | Target29 | -0.01 |
| 72 | 37 | Target37 | -0.01 |
| 73 | 121 | Target121 | -0.011 |
| 74 | 95 | Target95 | -0.011 |
| 75 | 7 | Target7 | -0.011 |
| 76 | 21 | Target21 | -0.011 |
| 77 | 122 | Target122 | -0.011 |
| 78 | 34 | Target34 | -0.012 |
| 79 | 86 | Target86 | -0.012 |
| 80 | 91 | Target91 | -0.012 |
| 81 | 127 | Target127 | -0.012 |
| 82 | 120 | Target120 | -0.013 |
| 83 | 70 | Target70 | -0.013 |
| 84 | 103 | Target103 | -0.013 |
| 85 | 15 | Target15 | -0.014 |
| 86 | 56 | Target56 | -0.014 |
| 87 | 113 | Target113 | -0.014 |
| 88 | 57 | Target57 | -0.015 |
| 89 | 96 | Target96 | -0.015 |
| 90 | 6 | Target6 | -0.015 |
| 91 | 97 | Target97 | -0.015 |
| 92 | 110 | Target110 | -0.016 |
| 93 | 48 | Target48 | -0.016 |
| 94 | 30 | Target30 | -0.017 |
| 95 | 8 | Target8 | -0.017 |
| 96 | 101 | Target101 | -0.017 |
| 97 | 44 | Target44 | -0.017 |
| 98 | 123 | Target123 | -0.018 |
| 99 | 104 | Target104 | -0.018 |
| 100 | 43 | Target43 | -0.018 |
| 101 | 14 | Target14 | -0.018 |
| 102 | 132 | Target132 | -0.019 |
| 103 | 116 | Target116 | -0.02 |
| 104 | 129 | Target129 | -0.02 |
| 105 | 35 | Target35 | -0.02 |
| 106 | 125 | Target125 | -0.021 |
| 107 | 92 | Target92 | -0.021 |
| 108 | 13 | Target13 | -0.022 |
| 109 | 22 | Target22 | -0.022 |
| 110 | 19 | Target19 | -0.022 |
| 111 | 75 | Target75 | -0.023 |
| 112 | 36 | Target36 | -0.023 |
| 113 | 100 | Target100 | -0.024 |
| 114 | 115 | Target115 | -0.024 |
| 115 | 131 | Target131 | -0.025 |
| 116 | 90 | Target90 | -0.025 |
| 117 | 76 | Target76 | -0.025 |
| 118 | 31 | Target31 | -0.025 |
| 119 | 54 | Target54 | -0.026 |
| 120 | 67 | Target67 | -0.026 |
| 121 | 62 | Target62 | -0.026 |
| 122 | 89 | Target89 | -0.027 |
| 123 | 105 | Target105 | -0.028 |
| 124 | 2 | Target2 | -0.028 |
| 125 | 74 | Target74 | -0.028 |
| 126 | 78 | Target78 | -0.028 |
| 127 | 68 | Target68 | -0.029 |
| 128 | 17 | Target17 | -0.029 |
| 129 | 47 | Target47 | -0.029 |
| 130 | 69 | Target69 | -0.03 |
| 131 | 93 | Target93 | -0.03 |
| 132 | 85 | Target85 | -0.031 |
| 133 | 55 | Target55 | -0.03 |
| 134 | 40 | Target40 | -0.031 |
| 135 | 73 | Target73 | -0.031 |
| 136 | 83 | Target83 | -0.032 |
| 137 | 64 | Target64 | -0.032 |
| 138 | 41 | Target41 | -0.033 |
| 139 | 52 | Target52 | -0.033 |
| 140 | 51 | Target51 | -0.034 |
| 141 | 24 | Target24 | -0.034 |
| 142 | 66 | Target66 | -0.034 |
| 143 | 84 | Target84 | -0.035 |
| 144 | 3 | Target3 | -0.035 |
| 145 | 18 | Target18 | -0.035 |
| 146 | 20 | Target20 | -0.036 |
| 147 | 9 | Target9 | -0.037 |
| 148 | 58 | Target58 | -0.037 |
| 149 | 1 | Target1 | -0.038 |
| 150 | 28 | Target28 | -0.039 |
| 151 | 50 | Target50 | -0.039 |
| 152 | 59 | Target59 | -0.041 |
| 153 | 94 | Target94 | -0.041 |
| 154 | 39 | Target39 | -0.042 |
| 155 | 108 | Target108 | -0.043 |
| 156 | 71 | Target71 | -0.043 |
| 157 | 5 | Target5 | -0.045 |
| 158 | 79 | Target79 | -0.045 |
| 159 | 72 | Target72 | -0.046 |
| 160 | 77 | Target77 | -0.047 |

**4. Output of mRMR for the target nuclear receptor (NR) class**

*** MaxRel features ***

| Order | Feature | Name | Score |
| --- | --- | --- | --- |
| 1 | 26 | Target26 | 0.055 |
| 2 | 104 | Target104 | 0.051 |
| 3 | 47 | Target47 | 0.044 |
| 4 | 101 | Target101 | 0.044 |
| 5 | 62 | Target62 | 0.038 |
| 6 | 18 | Target18 | 0.038 |
| 7 | 93 | Target93 | 0.036 |
| 8 | 87 | Target87 | 0.035 |
| 9 | 9 | Target9 | 0.032 |
| 10 | 91 | Target91 | 0.029 |
| 11 | 44 | Target44 | 0.028 |
| 12 | 17 | Target17 | 0.028 |
| 13 | 108 | Target108 | 0.028 |
| 14 | 28 | Target28 | 0.028 |
| 15 | 66 | Target66 | 0.028 |
| 16 | 77 | Target77 | 0.028 |
| 17 | 38 | Target38 | 0.027 |
| 18 | 103 | Target103 | 0.025 |
| 19 | 130 | Target130 | 0.025 |
| 20 | 6 | Target6 | 0.025 |
| 21 | 92 | Target92 | 0.024 |
| 22 | 126 | Target126 | 0.024 |
| 23 | 95 | Target95 | 0.024 |
| 24 | 110 | Target110 | 0.023 |
| 25 | 68 | Target68 | 0.023 |
| 26 | 35 | Target35 | 0.023 |
| 27 | 69 | Target69 | 0.022 |
| 28 | 111 | Target111 | 0.022 |
| 29 | 16 | Target16 | 0.022 |
| 30 | 31 | Target31 | 0.022 |
| 31 | 33 | Target33 | 0.022 |
| 32 | 11 | Target11 | 0.021 |
| 33 | 124 | Target124 | 0.021 |
| 34 | 46 | Target46 | 0.02 |
| 35 | 19 | Target19 | 0.02 |
| 36 | 121 | Target121 | 0.02 |
| 37 | 80 | Target80 | 0.02 |
| 38 | 20 | Target20 | 0.02 |
| 39 | 115 | Target115 | 0.019 |
| 40 | 50 | Target50 | 0.019 |
| 41 | 61 | Target61 | 0.019 |
| 42 | 4 | Target4 | 0.018 |
| 43 | 40 | Target40 | 0.018 |
| 44 | 41 | Target41 | 0.018 |
| 45 | 34 | Target34 | 0.018 |
| 46 | 72 | Target72 | 0.018 |
| 47 | 79 | Target79 | 0.018 |
| 48 | 3 | Target3 | 0.018 |
| 49 | 15 | Target15 | 0.017 |
| 50 | 7 | Target7 | 0.017 |
| 51 | 109 | Target109 | 0.017 |
| 52 | 67 | Target67 | 0.017 |
| 53 | 51 | Target51 | 0.017 |
| 54 | 55 | Target55 | 0.017 |
| 55 | 52 | Target52 | 0.016 |
| 56 | 94 | Target94 | 0.016 |
| 57 | 129 | Target129 | 0.016 |
| 58 | 2 | Target2 | 0.016 |
| 59 | 75 | Target75 | 0.015 |
| 60 | 59 | Target59 | 0.015 |
| 61 | 73 | Target73 | 0.015 |
| 62 | 1 | Target1 | 0.015 |
| 63 | 22 | Target22 | 0.015 |
| 64 | 123 | Target123 | 0.015 |
| 65 | 30 | Target30 | 0.015 |
| 66 | 105 | Target105 | 0.014 |
| 67 | 74 | Target74 | 0.014 |
| 68 | 71 | Target71 | 0.014 |
| 69 | 145 | Drug13 | 0.014 |
| 70 | 131 | Target131 | 0.014 |
| 71 | 5 | Target5 | 0.013 |
| 72 | 78 | Target78 | 0.013 |
| 73 | 113 | Target113 | 0.013 |
| 74 | 39 | Target39 | 0.013 |
| 75 | 106 | Target106 | 0.013 |
| 76 | 10 | Target10 | 0.013 |
| 77 | 85 | Target85 | 0.012 |
| 78 | 116 | Target116 | 0.012 |
| 79 | 36 | Target36 | 0.012 |
| 80 | 12 | Target12 | 0.012 |
| 81 | 82 | Target82 | 0.012 |
| 82 | 97 | Target97 | 0.012 |
| 83 | 24 | Target24 | 0.011 |
| 84 | 99 | Target99 | 0.011 |
| 85 | 56 | Target56 | 0.011 |
| 86 | 125 | Target125 | 0.011 |
| 87 | 96 | Target96 | 0.011 |
| 88 | 114 | Target114 | 0.01 |
| 89 | 48 | Target48 | 0.009 |
| 90 | 23 | Target23 | 0.008 |
| 91 | 29 | Target29 | 0.008 |
| 92 | 25 | Target25 | 0.008 |
| 93 | 158 | Drug26 | 0.008 |
| 94 | 58 | Target58 | 0.008 |
| 95 | 120 | Target120 | 0.008 |
| 96 | 86 | Target86 | 0.007 |
| 97 | 90 | Target90 | 0.007 |
| 98 | 76 | Target76 | 0.007 |
| 99 | 63 | Target63 | 0.007 |
| 100 | 159 | Drug27 | 0.007 |
| 101 | 160 | Drug28 | 0.006 |
| 102 | 143 | Drug11 | 0.006 |
| 103 | 136 | Drug4 | 0.005 |
| 104 | 60 | Target60 | 0.005 |
| 105 | 107 | Target107 | 0.005 |
| 106 | 150 | Drug18 | 0.005 |
| 107 | 57 | Target57 | 0.005 |
| 108 | 155 | Drug23 | 0.005 |
| 109 | 64 | Target64 | 0.005 |
| 110 | 146 | Drug14 | 0.005 |
| 111 | 83 | Target83 | 0.005 |
| 112 | 128 | Target128 | 0.005 |
| 113 | 13 | Target13 | 0.005 |
| 114 | 65 | Target65 | 0.005 |
| 115 | 132 | Target132 | 0.005 |
| 116 | 43 | Target43 | 0.005 |
| 117 | 117 | Target117 | 0.004 |
| 118 | 14 | Target14 | 0.004 |
| 119 | 133 | Drug1 | 0.004 |
| 120 | 84 | Target84 | 0.004 |
| 121 | 118 | Target118 | 0.003 |
| 122 | 27 | Target27 | 0.003 |
| 123 | 45 | Target45 | 0.003 |
| 124 | 54 | Target54 | 0.002 |
| 125 | 156 | Drug24 | 0.002 |
| 126 | 100 | Target100 | 0.002 |
| 127 | 42 | Target42 | 0.002 |
| 128 | 134 | Drug2 | 0.002 |
| 129 | 89 | Target89 | 0.002 |
| 130 | 8 | Target8 | 0.001 |
| 131 | 154 | Drug22 | 0.001 |
| 132 | 88 | Target88 | 0.001 |
| 133 | 37 | Target37 | 0.001 |
| 134 | 119 | Target119 | 0.001 |
| 135 | 140 | Drug8 | 0.001 |
| 136 | 141 | Drug9 | 0.001 |
| 137 | 127 | Target127 | 0.001 |
| 138 | 122 | Target122 | 0.001 |
| 139 | 49 | Target49 | 0.001 |
| 140 | 137 | Drug5 | 0 |
| 141 | 147 | Drug15 | 0 |
| 142 | 148 | Drug16 | 0 |
| 143 | 102 | Target102 | 0 |
| 144 | 149 | Drug17 | 0 |
| 145 | 53 | Target53 | 0 |
| 146 | 70 | Target70 | 0 |
| 147 | 112 | Target112 | 0 |
| 148 | 152 | Drug20 | 0 |
| 149 | 98 | Target98 | 0 |
| 150 | 21 | Target21 | 0 |
| 151 | 81 | Target81 | 0 |
| 152 | 144 | Drug12 | 0 |
| 153 | 32 | Target32 | 0 |
| 154 | 138 | Drug6 | 0 |
| 155 | 139 | Drug7 | 0 |
| 156 | 142 | Drug10 | 0 |
| 157 | 157 | Drug25 | 0 |
| 158 | 135 | Drug3 | 0 |
| 159 | 151 | Drug19 | 0 |

*** mRMR features ***

| Order | Feature | Name | Score |
| --- | --- | --- | --- |
| 1 | 26 | Target26 | 0.055 |
| 2 | 150 | Drug18 | 0.005 |
| 3 | 6 | Target6 | 0.001 |
| 4 | 145 | Drug13 | 0.001 |
| 5 | 32 | Target32 | 0 |
| 6 | 143 | Drug11 | 0.001 |
| 7 | 138 | Drug6 | 0 |
| 8 | 139 | Drug7 | 0 |
| 9 | 91 | Target91 | 0.003 |
| 10 | 142 | Drug10 | 0 |
| 11 | 157 | Drug25 | 0 |
| 12 | 146 | Drug14 | 0 |
| 13 | 92 | Target92 | 0 |
| 14 | 135 | Drug3 | 0 |
| 15 | 104 | Target104 | 0.003 |
| 16 | 151 | Drug19 | 0 |
| 17 | 153 | Drug21 | 0 |
| 18 | 149 | Drug17 | -0.001 |
| 19 | 137 | Drug5 | -0.001 |
| 20 | 134 | Drug2 | -0.001 |
| 21 | 141 | Drug9 | -0.002 |
| 22 | 70 | Target70 | -0.004 |
| 23 | 4 | Target4 | -0.004 |
| 24 | 147 | Drug15 | -0.004 |
| 25 | 160 | Drug28 | -0.004 |
| 26 | 144 | Drug12 | -0.005 |
| 27 | 121 | Target121 | -0.005 |
| 28 | 140 | Drug8 | -0.007 |
| 29 | 65 | Target65 | -0.007 |
| 30 | 148 | Drug16 | -0.008 |
| 31 | 101 | Target101 | -0.008 |
| 32 | 16 | Target16 | -0.011 |
| 33 | 112 | Target112 | -0.011 |
| 34 | 136 | Drug4 | -0.012 |
| 35 | 47 | Target47 | -0.012 |
| 36 | 158 | Drug26 | -0.011 |
| 37 | 152 | Drug20 | -0.015 |
| 38 | 86 | Target86 | -0.014 |
| 39 | 107 | Target107 | -0.015 |
| 40 | 49 | Target49 | -0.015 |
| 41 | 45 | Target45 | -0.015 |
| 42 | 133 | Drug1 | -0.016 |
| 43 | 11 | Target11 | -0.018 |
| 44 | 51 | Target51 | -0.018 |
| 45 | 159 | Drug27 | -0.019 |
| 46 | 98 | Target98 | -0.02 |
| 47 | 60 | Target60 | -0.019 |
| 48 | 21 | Target21 | -0.022 |
| 49 | 44 | Target44 | -0.022 |
| 50 | 154 | Drug22 | -0.024 |
| 51 | 62 | Target62 | -0.023 |
| 52 | 116 | Target116 | -0.025 |
| 53 | 156 | Drug24 | -0.025 |
| 54 | 93 | Target93 | -0.026 |
| 55 | 81 | Target81 | -0.027 |
| 56 | 115 | Target115 | -0.029 |
| 57 | 102 | Target102 | -0.031 |
| 58 | 18 | Target18 | -0.031 |
| 59 | 42 | Target42 | -0.034 |
| 60 | 36 | Target36 | -0.034 |
| 61 | 12 | Target12 | -0.037 |
| 62 | 35 | Target35 | -0.041 |
| 63 | 155 | Drug23 | -0.04 |
| 64 | 33 | Target33 | -0.043 |
| 65 | 127 | Target127 | -0.046 |
| 66 | 117 | Target117 | -0.047 |
| 67 | 126 | Target126 | -0.047 |
| 68 | 56 | Target56 | -0.049 |
| 69 | 105 | Target105 | -0.05 |
| 70 | 113 | Target113 | -0.052 |
| 71 | 31 | Target31 | -0.055 |
| 72 | 119 | Target119 | -0.057 |
| 73 | 9 | Target9 | -0.058 |
| 74 | 74 | Target74 | -0.06 |
| 75 | 53 | Target53 | -0.062 |
| 76 | 129 | Target129 | -0.062 |
| 77 | 132 | Target132 | -0.063 |
| 78 | 76 | Target76 | -0.064 |
| 79 | 99 | Target99 | -0.065 |
| 80 | 17 | Target17 | -0.066 |
| 81 | 128 | Target128 | -0.067 |
| 82 | 90 | Target90 | -0.07 |
| 83 | 8 | Target8 | -0.071 |
| 84 | 38 | Target38 | -0.072 |
| 85 | 82 | Target82 | -0.074 |
| 86 | 131 | Target131 | -0.076 |
| 87 | 43 | Target43 | -0.075 |
| 88 | 114 | Target114 | -0.078 |
| 89 | 30 | Target30 | -0.081 |
| 90 | 23 | Target23 | -0.081 |
| 91 | 54 | Target54 | -0.084 |
| 92 | 46 | Target46 | -0.086 |
| 93 | 103 | Target103 | -0.086 |
| 94 | 123 | Target123 | -0.086 |
| 95 | 84 | Target84 | -0.088 |
| 96 | 27 | Target27 | -0.09 |
| 97 | 73 | Target73 | -0.09 |
| 98 | 7 | Target7 | -0.091 |
| 99 | 130 | Target130 | -0.094 |
| 100 | 14 | Target14 | -0.095 |
| 101 | 125 | Target125 | -0.097 |
| 102 | 83 | Target83 | -0.099 |
| 103 | 96 | Target96 | -0.099 |
| 104 | 50 | Target50 | -0.1 |
| 105 | 48 | Target48 | -0.102 |
| 106 | 120 | Target120 | -0.102 |
| 107 | 25 | Target25 | -0.104 |
| 108 | 100 | Target100 | -0.105 |
| 109 | 64 | Target64 | -0.106 |
| 110 | 95 | Target95 | -0.108 |
| 111 | 88 | Target88 | -0.109 |
| 112 | 108 | Target108 | -0.109 |
| 113 | 78 | Target78 | -0.111 |
| 114 | 57 | Target57 | -0.111 |
| 115 | 22 | Target22 | -0.112 |
| 116 | 118 | Target118 | -0.116 |
| 117 | 34 | Target34 | -0.116 |
| 118 | 10 | Target10 | -0.117 |
| 119 | 13 | Target13 | -0.119 |
| 120 | 52 | Target52 | -0.119 |
| 121 | 63 | Target63 | -0.12 |
| 122 | 28 | Target28 | -0.122 |
| 123 | 58 | Target58 | -0.122 |
| 124 | 1 | Target1 | -0.123 |
| 125 | 106 | Target106 | -0.125 |
| 126 | 59 | Target59 | -0.126 |
| 127 | 29 | Target29 | -0.127 |
| 128 | 89 | Target89 | -0.13 |
| 129 | 39 | Target39 | -0.131 |
| 130 | 61 | Target61 | -0.132 |
| 131 | 124 | Target124 | -0.133 |
| 132 | 75 | Target75 | -0.135 |
| 133 | 94 | Target94 | -0.138 |
| 134 | 66 | Target66 | -0.139 |
| 135 | 71 | Target71 | -0.142 |
| 136 | 85 | Target85 | -0.145 |
| 137 | 77 | Target77 | -0.147 |
| 138 | 122 | Target122 | -0.154 |
| 139 | 24 | Target24 | -0.156 |
| 140 | 5 | Target5 | -0.157 |
| 141 | 69 | Target69 | -0.16 |
| 142 | 3 | Target3 | -0.162 |
| 143 | 41 | Target41 | -0.163 |
| 144 | 15 | Target15 | -0.17 |
| 145 | 111 | Target111 | -0.172 |
| 146 | 109 | Target109 | -0.176 |
| 147 | 110 | Target110 | -0.179 |
| 148 | 97 | Target97 | -0.183 |
| 149 | 87 | Target87 | -0.186 |
| 150 | 67 | Target67 | -0.188 |
| 151 | 40 | Target40 | -0.188 |
| 152 | 68 | Target68 | -0.194 |
| 153 | 37 | Target37 | -0.196 |
| 154 | 55 | Target55 | -0.197 |
| 155 | 79 | Target79 | -0.202 |
| 156 | 72 | Target72 | -0.21 |
| 157 | 19 | Target19 | -0.215 |
| 158 | 2 | Target2 | -0.229 |
| 159 | 80 | Target80 | -0.239 |
| 160 | 20 | Target20 | -0.247 |
