# Supplementary material for: Predicting Drug-Target Interaction Networks Based on Functional Groups and Biological Features
Source: PLoS One. 2010 Mar 11;5(3):e9603. doi: 10.1371/journal.pone.0009603 (PMC2836373; doi:10.1371/journal.pone.0009603)
Supplement: Online Supporting Information S6 — The Results of Forward Feature Selection (FFS). (0.12 MB DOC) [file pone.0009603.s006.doc]

**Online Supporting Information S6: The Results of Forward Feature Selection (FFS)**

**1. Selected features with FFS for target E (enzyme) class**

| Feature | Name | Feature Description |
| --- | --- | --- |
| 131 | Target131 | Amino Acids Composition |
| 143 | Drug11 | imine |
| 154 | Drug22 | ar_6c_ring |
| 119 | Target119 | Amino Acids Composition |
| 62 | Target62 | Normalized Van Der Waals volume |
| 140 | Drug8 | methyl |
| 148 | Drug16 | sulfonic acid |
| 31 | Target31 | Secondary structure |
| 155 | Drug23 | non_ar_5c_ring |
| 81 | Target81 | Polarity |
| 141 | Drug9 | ester |
| 6 | Target6 | Hydrophobicity |
| 38 | Target38 | Secondary structure |
| 149 | Drug17 | sulfone |
| 138 | Drug6 | phosphorus |
| 23 | Target23 | Secondary structure |
| 136 | Drug4 | amine |
| 145 | Drug13 | nitro |
| 137 | Drug5 | hydroxamic acid |
| 35 | Target35 | Secondary structure |
| 24 | Target24 | Secondary structure |
| 133 | Drug1 | alcohol |
| 134 | Drug2 | aldehyde |
| 151 | Drug19 | sulfoxide |
| 144 | Drug12 | ketone |
| 146 | Drug14 | halogen |
| 160 | Drug28 | hetero ar_5_ring |
| 135 | Drug3 | amide |
| 139 | Drug7 | carboxylate |
| 157 | Drug25 | hetero ar_6_ring |
| 153 | Drug21 | a_5c_ring |
| 142 | Drug10 | ether |

**2. Selected features with FFS for target GPCR class**

| Feature | Name | Feature Description |
| --- | --- | --- |
| 79 | Target79 | Polarity |
| 140 | Drug8 | methyl |
| 117 | Target117 | Amino Acids Composition |
| 150 | Drug18 | sulfonamide |
| 35 | Target35 | Secondary structure |
| 26 | Target26 | Secondary structure |
| 133 | Drug1 | alcohol |
| 89 | Target89 | Polarity |
| 156 | Drug24 | non_ar_6c_ring |
| 136 | Drug4 | amine |
| 97 | Target97 | Polarizability |
| 143 | Drug11 | imine |
| 37 | Target37 | Secondary structure |
| 155 | Drug23 | non_ar_5c_ring |
| 159 | Drug27 | hetero non_ar_6_ring |
| 158 | Drug26 | hetero non_ar_5_ring |
| 70 | Target70 | Normalized Van Der Waals volume |
| 64 | Target64 | Normalized Van Der Waals volume |
| 134 | Drug2 | aldehyde |
| 160 | Drug28 | hetero ar_5_ring |
| 74 | Target74 | Polarity |
| 130 | Target130 | Amino Acids Composition |
| 42 | Target42 | Secondary structure |
| 71 | Target71 | Polarity |
| 157 | Drug25 | hetero ar_6_ring |
| 138 | Drug6 | phosphorus |
| 135 | Drug3 | amide |
| 153 | Drug21 | a_5c_ring |
| 142 | Drug10 | ether |
| 47 | Target47 | Solvent accessibility |

**3. Selected features with FFS for target iron channel (IC) class**

| Featue | Name | Feature Description |
| --- | --- | --- |
| 23 | Target23 | Secondary structure |
| 145 | Drug13 | nitro |
| 10 | Target10 | Hydrophobicity |
| 136 | Drug4 | amine |
| 118 | Target118 | Amino Acids Composition |
| 155 | Drug23 | non_ar_5c_ring |
| 146 | Drug14 | halogen |
| 98 | Target98 | Polarizability |
| 38 | Target38 | Secondary structure |
| 144 | Drug12 | ketone |
| 135 | Drug3 | amide |
| 157 | Drug25 | hetero ar_6_ring |
| 65 | Target65 | Normalized Van Der Waals volume |
| 158 | Drug26 | hetero non_ar_5_ring |
| 27 | Target27 | Secondary structure |
| 143 | Drug11 | imine |
| 152 | Drug20 | sulfide |
| 159 | Drug27 | hetero non_ar_6_ring |
| 160 | Drug28 | hetero ar_5_ring |
| 137 | Drug5 | hydroxamic acid |
| 133 | Drug1 | alcohol |
| 151 | Drug19 | sulfoxide |
| 80 | Target80 | Polarity |
| 25 | Target25 | Secondary structure |
| 140 | Drug8 | methyl |
| 134 | Drug2 | aldehyde |
| 81 | Target81 | Polarity |
| 141 | Drug9 | ester |
| 156 | Drug24 | non_ar_6c_ring |
| 147 | Drug15 | thiol |
| 149 | Drug17 | sulfone |
| 150 | Drug18 | sulfonamide |
| 153 | Drug21 | a_5c_ring |
| 138 | Drug6 | phosphorus |
| 142 | Drug10 | ether |
| 148 | Drug16 | sulfonic acid |
| 88 | Target88 | Polarity |

**4. Selected features with FFS for target nuclear receptor (NR) class**

| Feature | Name | Feature Description |
| --- | --- | --- |
| 26 | Target26 | Secondary structure |
| 150 | Drug18 | sulfonamide |
| 6 | Target6 | Hydrophobicity |
| 145 | Drug13 | nitro |
| 32 | Target32 | Secondary structure |
| 143 | Drug11 | imine |
| 138 | Drug6 | phosphorus |
| 139 | Drug7 | carboxylate |
| 91 | Target91 | Polarity |
| 142 | Drug10 | ether |
| 157 | Drug25 | hetero ar_6_ring |
| 146 | Drug14 | halogen |
| 92 | Target92 | Polarizability |
| 135 | Drug3 | amide |
| 104 | Target104 | Polarizability |
| 151 | Drug19 | sulfoxide |
| 153 | Drug21 | a_5c_ring |
| 149 | Drug17 | sulfone |
| 137 | Drug5 | hydroxamic acid |
| 156 | Drug24 | non_ar_6c_ring |
| 159 | Drug27 | hetero non_ar_6_ring |
| 86 | Target86 | Polarity |
| 147 | Drug15 | thiol |
| 148 | Drug16 | sulfonic acid |
| 16 | Target16 | Hydrophobicity |
| 35 | Target35 | Secondary structure |
| 98 | Target98 | Polarizability |
| 133 | Drug1 | alcohol |
| 140 | Drug8 | methyl |
| 60 | Target60 | Normalized Van Der Waals volume |
| 44 | Target44 | Solvent accessibility |
| 136 | Drug4 | Amine |
